# Steric‐Adaptive Biocatalysis: Imine Reductase‐Mediated Dynamic Kinetic Resolution for Atroposelective Synthesis of Hindered Biaryl Amines

**DOI:** 10.1002/advs.202510455

**Published:** 2025-09-26

**Authors:** Zhichao Ni, Jingyuan Zhuang, Yadong Gao, Guangsheng Gao, Zhenling Liu, Ping Su, Gui‐Juan Cheng, Li‐Cheng Yang

**Affiliations:** ^1^ State Key Laboratory of Bioactive Substance and Function of Natural Medicines Institute of Materia Medica Chinese Academy of Medical Sciences & Peking Union Medical College Beijing 100050 China; ^2^ Warshel Institute for Computational Biology School of Medicine The Chinese University of Hong Kong Shenzhen Guangdong 518172 China; ^3^ School of Pharmaceutical Engineering Shenyang Pharmaceutical University Shenyang 110016 China; ^4^ State Key Laboratory for Quality Ensurance and Sustainable Use of Dao‐di Herbs National Resource Center for Chinese Materia Medica China Academy of Chinese Medical Sciences Beijing 100700 China

**Keywords:** biaryl amine, borrowing hydrogen cascade, dynamic kinetic resolution, imine reductase, molecular dynamics simulation

## Abstract

As an efficient and environmentally benign strategy, enzymatic catalysis has emerged with high reactivity and selectivity for atropisomeric structures. Atropisomeric biaryl amines are of great importance in drug development and asymmetric catalysis. However, substrates with steric hindrance are often poorly accepted by enzymes, as the immense pockets required are not commonly found in wild‐type enzymes. In this work, an imine reductase (IRED)‐catalyzed dynamic kinetic resolution (DKR) strategy is introduced for the synthesis of atropisomeric biaryl amines, especially those with bulky substitutions on (hetero)aryls, including naphthalene‐ and quinoline‐containing biaryl scaffolds. A broad range of atropisomeric biaryl amines are obtained in up to 99% yield and 99% *ee*, enabled by the steric adaptability of IR‐09. Besides, a borrowing hydrogen cascade system is explored to extend the construction of atropisomeric biaryl amines directly from racemic alcohols. Density functional theory (DFT) calculations and molecular dynamics (MD) simulations are carried out to elucidate the DKR process, selectivity of IR‐09, and interactions between the sterically hindered substrates and residues within the enzyme.

## Graphical abstract

An unusual IRED platform is reported, enabling efficient DKR via reductive amination without any evolutionary engineering. The system accommodates extremely sterically demanding substrates, including naphthyl and quinoline derivatives, delivering naphthyl‐aryl and quinoline‐aryl amine atropisomers with excellent efficiency and stereocontrol (over 80% yield and 90% *ee* for 14 examples out of 33 examples). This methodology significantly expands the accessible chemical space for biocatalytic atroposelective synthesis.

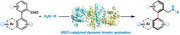

## 1 Introduction

In the field of asymmetric chemistry, the synthesis of chiral amines attracts much attention and enthusiasm.^[^ ^1^ ^]^ While significant advances have been recently recorded in the chemoenzymatic synthesis of various chiral amines, the recognition and selectivity of substrates or intermediates raise the level of difficulty due to the catalytic specificity of the enzyme. Most‐studied enzymes involved in this process include imine reductases (IREDs),^[^ ^2^, ^3^, ^4^ ^]^ transaminases (TAs),^[^ ^5^, ^6^, ^7^ ^]^ monoamine oxidases (MAOs),^[^ ^8^, ^9^ ^]^ and engineered miscellaneous enzymes for direct C─H amination.^[^ ^10^, ^11^, ^12^ ^]^ The family of IREDs, with the ability to generate stereocenter in C─N bonds through asymmetric reduction of imines in a NAD(P)H‐dependent manner, stands out for the synthesis of chiral amines, and garners significant interest from researchers.

**Figure 1:**
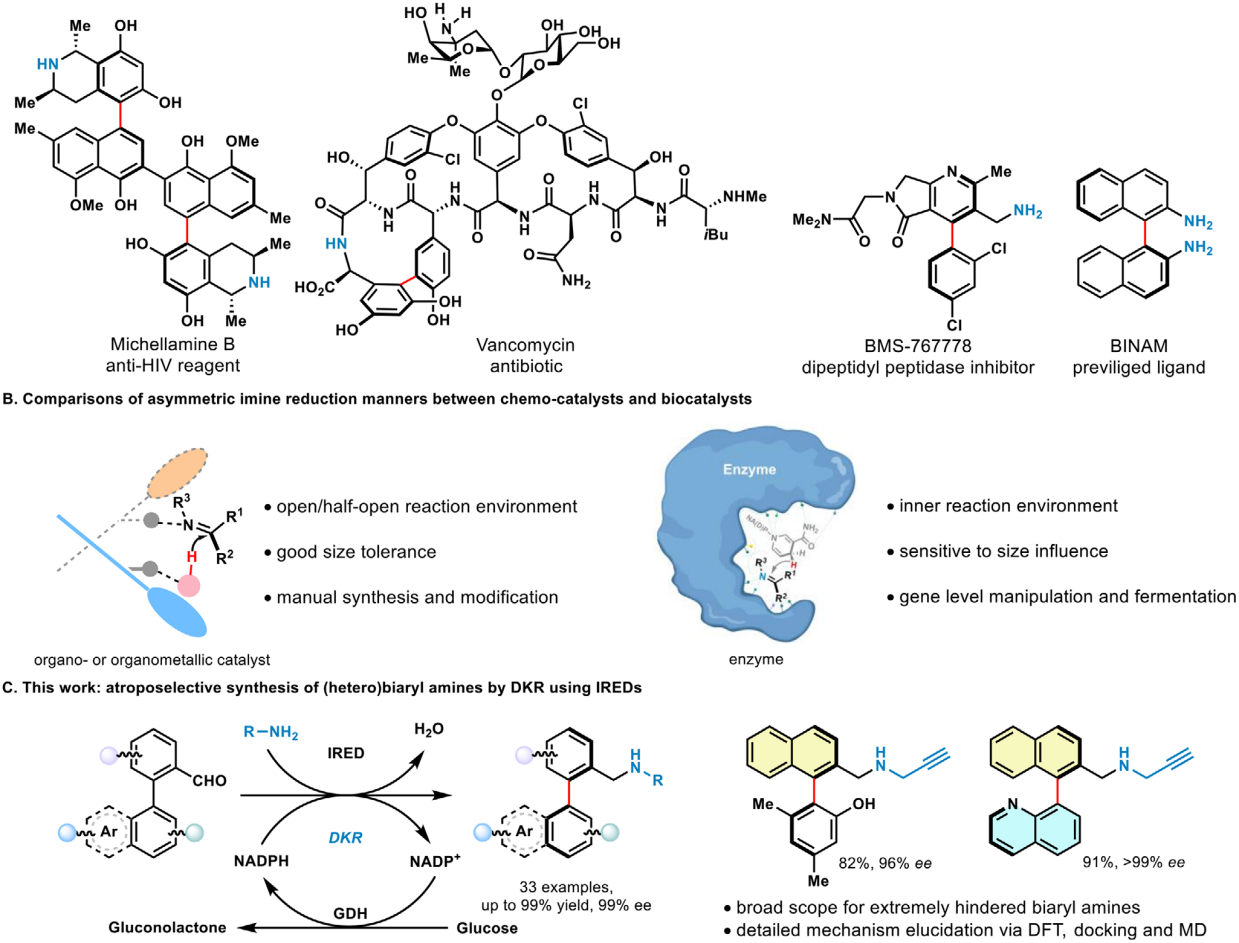
Atroposelective synthesis of axially chiral biaryl amines. A) Exemplified molecules with atropisomeric biaryl amine moiety. B) Comparisons of asymmetric imine reduction manners between chemo‐catalysts and biocatalysts. C) This work: atroposelective synthesis of (hetero)biaryl amines by DKR using IREDs.

Atropisomeric biaryl amines represent a privileged scaffold in many natural products and chiral pharmaceutics.^[^ ^13^, ^14^ ^]^ Type II/III atropisomers, characterized by ortho‐bulky substituents or extended aromatic systems (such as naphthyl groups), exhibit restricted rotation (ΔG^‡^ >20 kcal mol^−1^) with half‐lives ranging from minutes to years.^[^ ^15^ ^]^ This axial chirality plays a vital role in the construction of well‐known drugs and potential molecules, such as anti‐HIV agent michellamine B,^[^ ^16^ ^]^ glycopeptide antibiotic vancomycin,^[^ ^17^ ^]^ anti‐diabetes candidate BMS‐767778^[^ ^18^ ^]^ (**Figure** **1**A). Additionally, atropisomeric biaryl amines act as versatile ligands in asymmetric catalysis, like BINAM and NOBIN derivatives.^[^ ^19^, ^20^, ^21^ ^]^

Given the widespread application of atropisomeric biaryl amines in drug discovery and asymmetric synthesis, the development of efficient approaches through an atroposelective manner remains one of the most demanding and challenging targets. Many metal or organo‐catalysis strategies have been developed to construct the atropisomeric biaryl amines,^[^ ^22^, ^23^, ^24^, ^25^, ^26^, ^27^, ^28^, ^29^ ^]^ including 1) direct formation of the axially chiral bond through cross coupling strategy, 2) cycloaddition of the chiral arene motif via [4+2], [2+2+2] or [3+2], 3) fused ring fragmentation, 4) desymmetrization, 5) KR (kinetic resolution)/DKR (dynamic kinetic resolution) of a prechiral compound. With the rise of green chemistry, enzymatic synthesis represents an environmentally benign and sustainable strategy due to its efficiency, selectivity, variability, and reproducibility.^[^ ^30^, ^31^, ^32^ ^]^ In 2022, Narayan group reported an elegant enzymatic hetero‐cross coupling to access biaryls containing *N*‐heteroarenes, which highlighted the broad substrate promiscuity of the P450 enzymes.^[^ ^33^ ^]^ Similar strategies were subsequently employed by Molinaro and Zhang to prepare the cyclic peptide via Tyrosine–Tyrosine coupling.^[^ ^34^, ^35^ ^]^ Active pharmaceutic ingredients with atropisomeric biaryl amine could also be achieved by lipases through kinetic resolution, such as the asymmetric synthesis toward JNJ‐4355 (a nanomolar potent MCL‐1 inhibitor) through an unprecedented Suzuki–Miyaura/enzymatic process.^[^ ^36^, ^37^ ^]^ Another attractive strategy for the construction of atropisomeric biaryl amines is desymmetrization via engineered IREDs.^[^ ^38^, ^39^ ^]^ In 2024, Fu group reported the first DKR system catalyzed by IRED to access atropisomeric biaryl amines,^[^ ^40^, ^41^ ^]^ in which suitable substrates with fewer restrictions were extensively studied, such as biphenyl, whereas larger frameworks lacked comprehensive explorations.

Chemo‐catalyzed methods for asymmetric imine reduction typically create an open or semi‐open environment (like “chair claw”) to induce the formation of new chiral centers, utilizing organo‐ or organometallic catalysts. Accordingly, the substrates may typically exhibit a wide spectrum of substitutions, from small to large (Figure 1B left). In IRED catalysis, the active site generally creates a shielded or half‐shielded sphere of amino acid residues, which establishes a distinct chiral environment that limiting the imine substrates to adapt the pocket dimension and geometry, thus commonly rejecting bulky or non‐natural substrates (Figure 1B right). Due to structural limitations, enzymes are usually ineffective for other non‐native substrates, especially those with steric hindrance. Extensive enzyme engineering would be necessary to enhance the capacity of enzymes to adapt to desired molecules.^[^ ^42^, ^43^, ^44^, ^45^, ^46^ ^]^

Here, we present a steric‐adaptive biocatalytic DKR strategy for the atroposelective synthesis of biaryl amines through IRED‐catalyzed reductive amination without further evolutionary engineering. This enzyme could achieve steric‐adaptive catalysis through the dynamical accommodation among active center and substrates. Consequently, our enzymatic system effectively produced naphthyl‐aryl and quinoline‐aryl amine atropisomers with remarkable efficiency and enantioselectivity from highly sterically biaryl substrates (Figure 1C). In this system, racemization of the biaryl hydroxy aldehyde occurs via an equilibrium between the biaryl *N,O*‐acetal and corresponding biaryl imine.^[^ ^47^ ^]^ For heterobiaryl substrates, racemization is further facilitated by a transient Lewis acid‐base interaction between quinoline nitrogen and carbonyl group.^[^ ^48^ ^]^ This work may greatly broaden the available chemical space for biocatalytic atroposelective synthesis.

## 2 Results and Discussion

### 2.1 Enzyme Optimization

**Figure 2:**
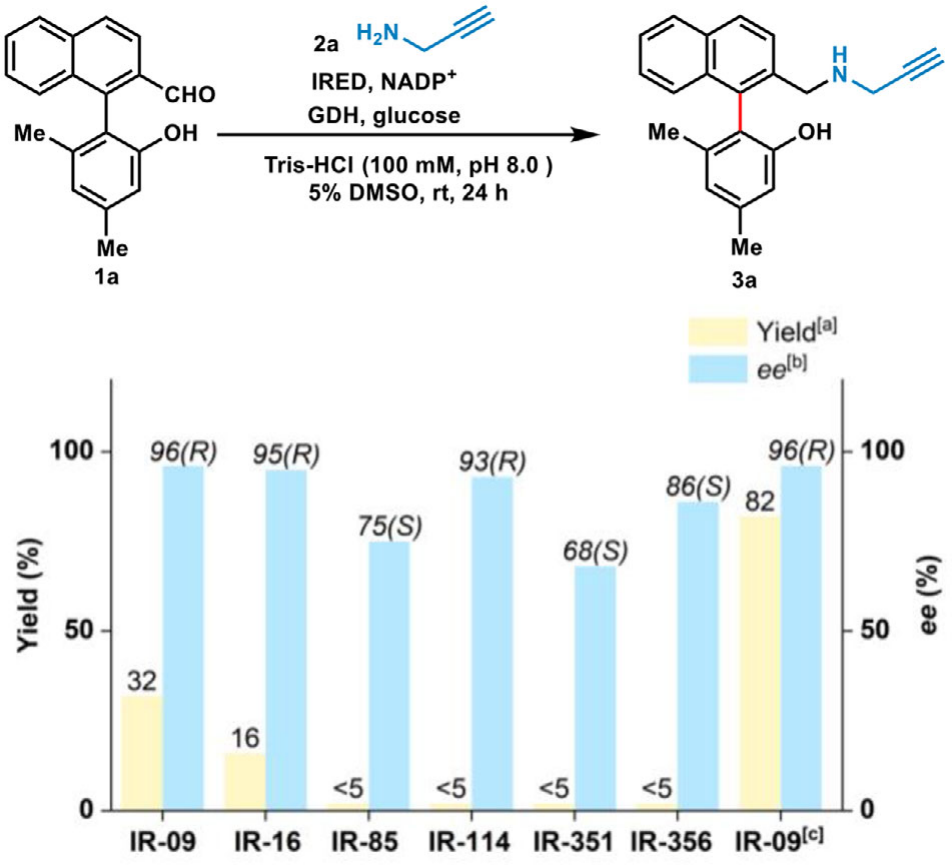
Reaction optimization. Reaction conditions: **1a** (5 µmol, 1 equiv.), **2a** (10 µmol, 2 equiv.), NADP^+^ (0.5 µmol, 10 mol%), glucose (15 µmol, 3 equiv.), GDH (1 mg mL^−1^), lysates of IRED in Tris‐HCl buffer (100 mm, pH 8.0) with 5% DMSO as the cosolvent at rt for 24 h, the final total volume is 1 mL. a) Yield was confirmed by analysis of the crude reaction mixture on HPLC with a prepared standard curve. b) Enantiomeric excess (*ee*) was determined by HPLC on a chiral stationary phase. Chirality was confirmed by comparison with the reported data. c) Reaction in 0.05 mmol scale with 8 equiv. **2a** at 37 °C, isolated yield.

We chose the sterically hindered biaryl aldehyde **1a** as our model substrate, which showed suboptimal performance in previous reports.^[^ ^40^ ^]^ Initial screening was carried out on the basis of 50 collected IREDs under common reaction conditions with propargylamine **2a** and NADPH circulations. The targeted product **3a** was detected only within eight reactions yet in good enantioselectivity (65–96% *ee*, with both *R‐* and *S‐* selectivity) catalyzed by IR‐09, IR‐16, IR‐85, IR‐104, IR‐114, IR‐202, IR‐351, and IR‐356, via TLC and HPLC analysis (Table S1, Supporting Information). Among them, three enzymes IR‐09 (yield 32%), IR‐16 (yield 16%), and IR‐114 (yield <5%)^[^ ^49^, ^50^ ^]^ showed more than 90% *R*‐selectivity (**Figure** **2**). As (*R*)‐atropisomer were not well‐explored through IRED catalysis,^[^ ^40^ ^]^ IR‐09, from *Aspergillus lentulus*, was prioritized for further systematical evaluation, including equivalents of amine, temperature, and pH of the buffer (Table S2, Supporting Information). Under the circulation of NADP^+^/GDH/glucose, the product **3a** was successfully synthesized with 82% yield and 96% *ee* of (*R*)‐enantiomer, using 8 equivalents of propargylamine (**2a**) and purified IR‐09 (0.2 mol%) in Tris‐HCl buffer (100 mm, pH 8.0) at 0.05 mmol scale of substrate **1a** (Figure 2).

### 2.2 Substrate Tolerance Test

**Scheme 1:**
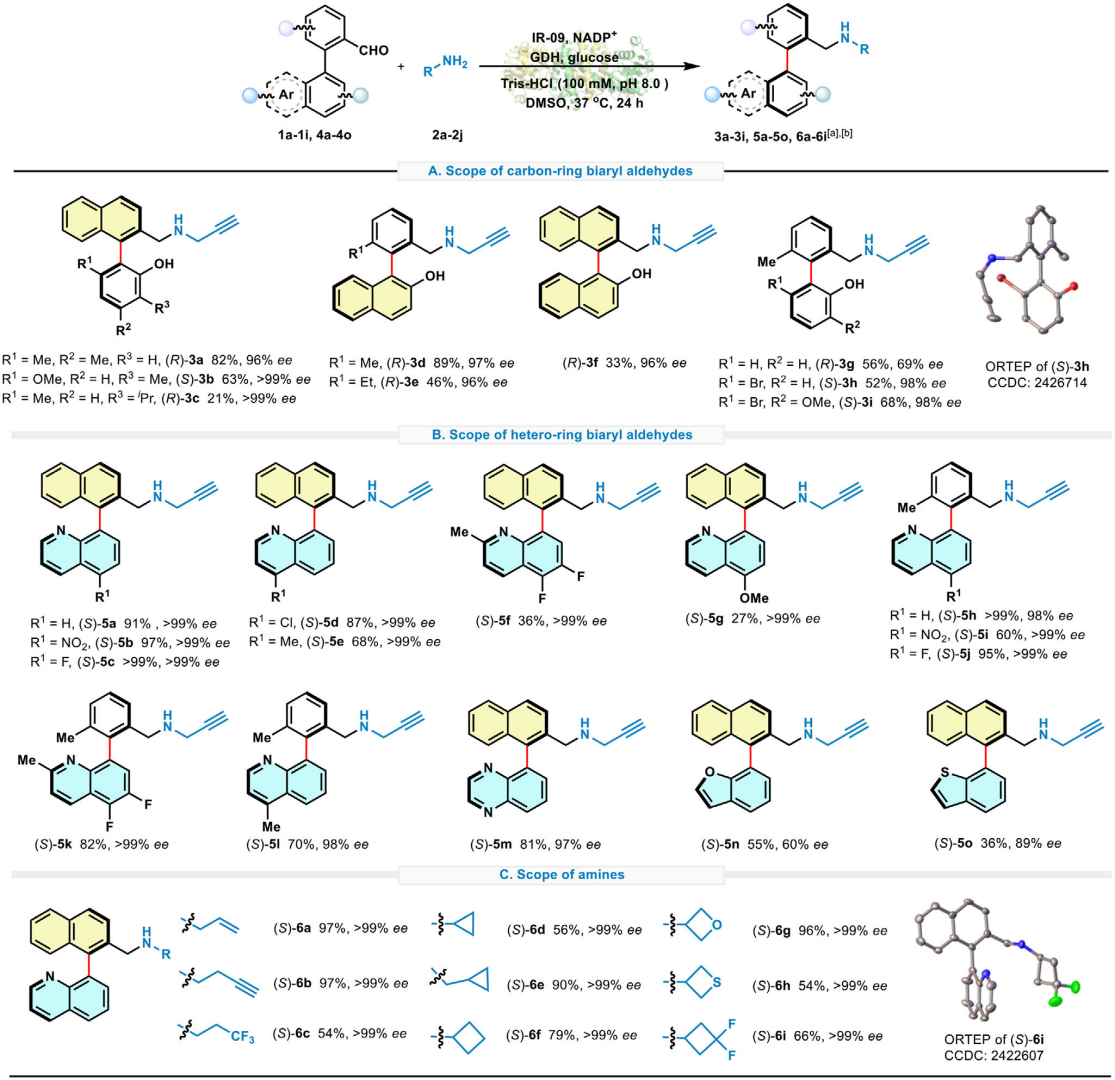
Biaryl amine scope of the IR‐09‐catalyzed DKR reaction. Standard conditions: biaryl aldehyde (**1a**–**i**, **4a**–**o**, 0.05 mmol, 1 equiv.), amine (**2a**–**j**, 0.40 mmol, 8 equiv.), NADP^+^ (0.005 mmol, 10 mol%), glucose (0.15 mol, 3 equiv.), GDH (1 mg mL^−1^), purified IR‐09 (0.2 mol% for biaryl aldehyde scope or 0.8 mol% for amine scope) in Tris‐HCl buffer (100 mm, pH 8.0) with 5% DMSO as the cosolvent at 37 °C for 24 h, the final total volume is 10 mL. a) Isolated yields after silica gel column chromatography purification. b) *ee* was determined by HPLC on a chiral stationary phase.

Under the promotion of the potential of IR‐09, we set out to explore the substrate applicability of this biocatalysis system. First, various sterically hindered biaryl aldehydes bearing naphthalene moieties were evaluated as shown in **Scheme** **1**A. IR‐09 demonstrated robust enantioselectivity for both naphthalene‐phenol or phenyl‐naphthol aldehyde substrates **3a**–**e** (96–>99% *ee*). However, for substrates with an enlarged functional group like **3c** with ‐*^i^* Pr and **3e** with ‐Et, the capacities of IR‐09 were not adequately demonstrated (21% and 46%). As for bis‐naphthalene substrate **3f**, satisfied stereoselectivity (96% *ee*) but further decreased conversion (33%) was observed, which may attribute to heightened rotational barriers (ΔG^‡^ = 26.3 kcal mol^−1^, see Figure S9, Supporting Information). Although excessive steric hindrance may reduce the reactivity, relative steric hindrance is essential for conformational transformation to adapt the enzyme. Product **3g** with less‐hindered biphenyl performed moderate yield and *ee* value, while the analogues **3h**,**i** with bromo‐substituted biphenyl exhibited increased yield and excellent stereoselectivity (98% *ee*). The improvement of yield from **3h** (52%) to **3i** (68%) indicated the importance of enough steric hindrance of the substrates. The product **3g** was identified as (*R*)‐enantiomer according to the reported data,^[^ ^40^ ^]^ while product **3h** was analyzed by X‐ray single crystal diffraction and was determined to be the (*S*)‐enantiomer. According to nomenclature conventions, the opposite chirality comes from the reversed priority sequence of the substitution, which was omitted in the previous studies. These results suggest that there's a considerable pocket within the enzyme to accommodate the aryl groups, as the bulky aromatic rings are more suitable for substrate recognition.

With the good bio‐transformation of aromatic rings, the goal of this research was further expanded to heterocycle aromatic rings and naphthalene‐quinoline substrates, which possessed potential for the discovery of anti‐virus molecules (Scheme 1B).^[^ ^51^ ^]^ With the introduction of heterocyclic aromatic rings, the majority of products showed excellent stereoselectivity from **5a** to **5m**, while the products **5n** (60% *ee*) and **5o** (89% *ee*) were not well‐behaved, possibly due to the disappearance of interactions from *N*‐heteroarene to NADPH. As for the conversion of **5a**–**e**, all products delivered exceptional outcomes with 68–99% yields, especially naphthalene‐quinoline substrates modified by electron‐withdrawing groups like ‐NO_2_ and ‐F. However, the addition of methyl group may obstruct the recognition and interaction between substrate and enzyme, which leads to a modest yield of **5f**. The addition of electron‐donating groups at C‐4 of quinoline also weakens this enzymatic system like **5g**, with only 27% yield. Besides, comparing the substrates with naphthalene (**5a–** **g**), the phenyl derivatives (**5h**–**l**) maintained high yields (60–>99%) and *ee* values (98–>99%), which indicates that naphthalene skeleton may be unnecessary in this system.

**Figure 3:**
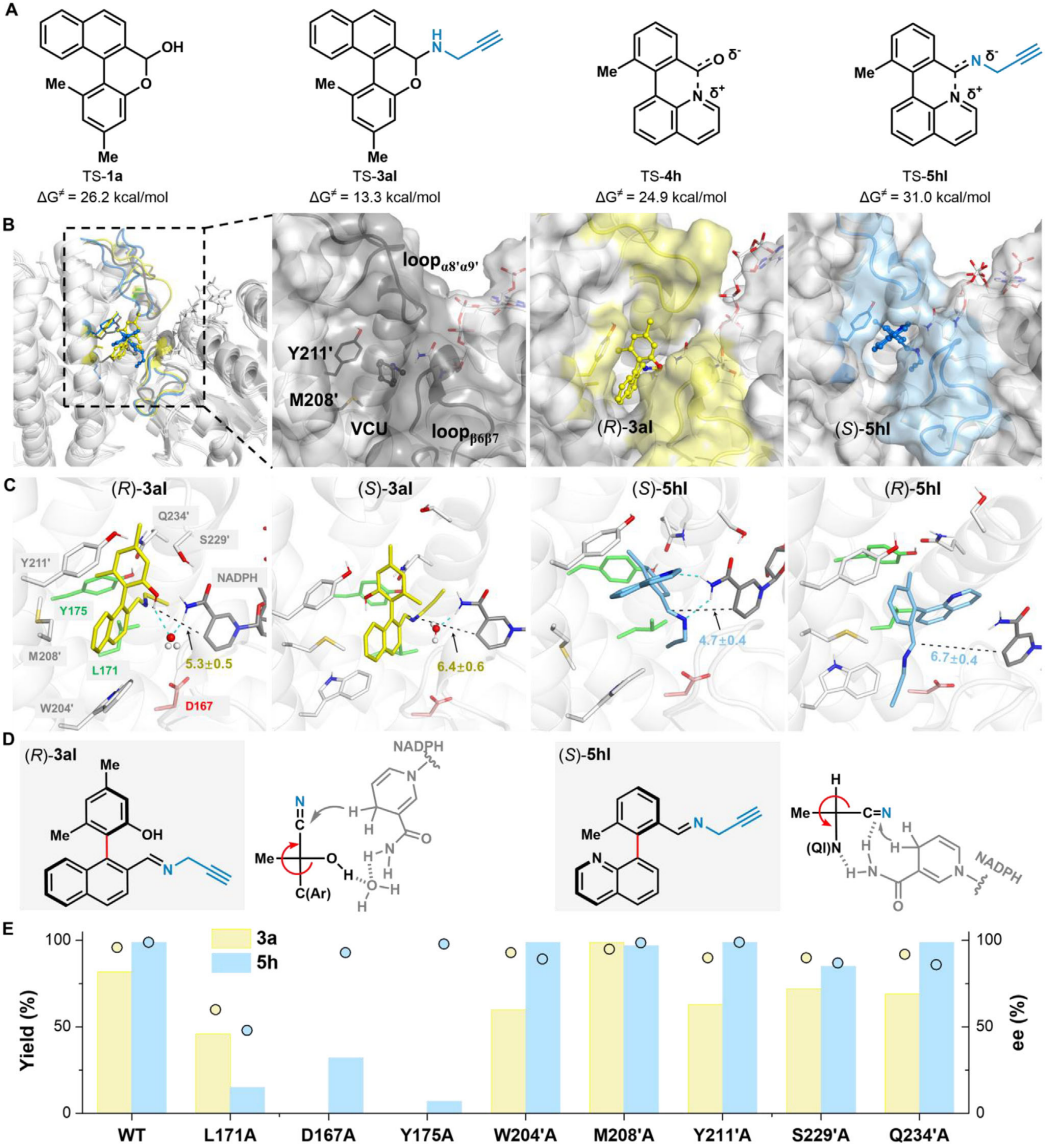
Mechanistic insight for IR‐09‐catalyzed reaction. A) DFT calculation of the rotation barrier energy for DKR. B) Comparison of the IR‐09 active pocket with the original substrate and our two bulky imine intermediates bound. VCU, *N*‐methylcyclohexanamine. C) Representative structures of IR‐09 with (*R*)‐ and (*S*)‐enantiomers of **3aI** and **5hI**, respectively. D) Fischer's Projection Formula of the imine intermediates and the chirality characterization. E) Mutation experiment results. Bar charts depict the yield values, while circles represent the enantiomeric excess (*ee*) values.

Later, we evaluated the compatibility of IR‐09 for different amines (Scheme 1C). Except for the propargylamine (**2a**), linear amines like allylamine (**2b**) and 3‐butynylamine (**2c**) could be efficiently used for the amination process, with 97% yield and >99% *ee* for products **6a**,**b**, while trifluoropropylamine (**2d**) showed limited reactivity (**6c**, 54% yield, >99% *ee*). Cyclic amines like cyclopropyl (**2e**,**f**), cyclobutene (**2g**), and heterocyclobutene variants (**2h**–**j**) delivered **6d**–**i** with excellent *ee* values (>99%), but the yields exhibited varied levels between 54% and 96%. However, bulky aliphatic/aromatic amines were not accepted due to redundant steric hindrance, including benzylamine, phenylamine, cyclohexylamine, and others we tested (Figure S8, Supporting Information). The absolute stereochemistry of (*S*)‐**6i** was determined by X‐ray single crystal diffraction, and that of other products **5a–** **o** and **6a**–**h** were tentatively assigned by analogy (Scheme 1C).

### 2.3 Product Derivatization

**Scheme 2:**
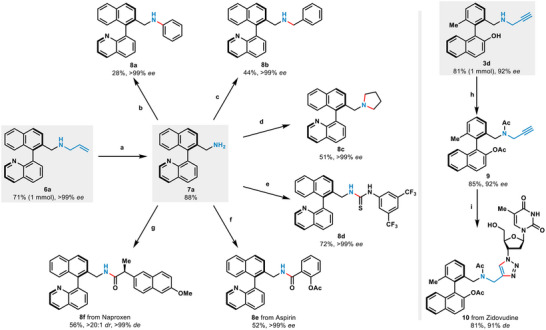
Derivatization of biaryl amines. a) 1,3‐dimethylbarbituric acid, Pd(PPh_3_)_4_, dichloromethane, reflux. b) Phenylboronic acid, *n*‐Bu_4_NOH, *t*‐BuOK, Cu(OAC)_2_, rt. c) Benzyl bromide, potassium carbonate, acetonitrile. d) 1,4‐Dibromobutane, potassium carbonate, acetonitrile. e) 1‐isothiocyanato‐3,5 bis(trifluoromethyl)benzene, dichloromethane. f) Aspirin, DIPEA, HATU. g) Naproxen, DIPEA, HATU. h) acetic anhydride, potassium carbonate, dichloromethane. i) Zidovudine, CuSO_4_·5H_2_O, Vitamin C, THF/water mixture (3:1). See Supporting Information for detailed information.

To expand the application of this enzymatic strategy (**Scheme** **2**), the model reaction between **4a** and **2b** was successfully executed at 1 mmol scale, affording **6a** with satisfied yield (71%) and stereoselectivity (99% *ee*). Subsequent allyl deprotection of **6a** resulted in the generation of primary amine **7a**, which was further diversified through cross‐coupling with phenyl (**8a**), benzyl (**8b**), and aliphatic electrophiles (**8c**) (28–51% yield). This approach enabled the successful synthesis of atropisomeric biaryl amine products that were not compatible with the standard enzymatic reactions. Later, through one‐step transformation, the primary amine **7a** was effectively converted into quinoline‐thiourea structures **8d**, a chiral bifunctional catalyst bearing both Lewis base and hydrogen‐bonding moiety.^[^ ^52^, ^53^ ^]^ Amine **7a** could also be derivatized to amide products **8e**,**f** with Aspirin and Naproxen. Notably, the configurations of all the products were fully maintained (>99% *ee*). Similarly, 1 mmol scale transformation of **1d** to biaryl amine **3d** was achieved with retained stereoselectivity (92% *ee*) and 81% yield. After two steps including acetylation and click reaction with the anti‐HIV nucleoside drug Zidovudine, we successfully obtained the biaryl‐nucleoside conjugate **10** (63% over two steps, 91% *de*).

### 2.4 Investigation of the Borrowing Hydrogen Cascade Reaction

**Scheme 3:**
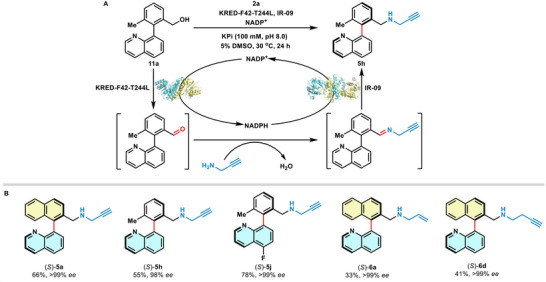
Direct synthesis of biaryl amines from alcohols via borrowing hydrogen cascade. A) Reaction pathway of biocatalytic hydrogen borrowing cascade. B) Scope of biocatalytic borrowing hydrogen cascade. Standard conditions: biaryl alcohols (**11a**–**c**, 0.05 mmol, 1 equiv.), amine (**2a**–**c**, 0.75 mmol, 15 equiv.), NADP^+^ (0.0075 mmol, 15 mol%), purified IR‐09 (2 mol%), and KRED‐F42‐T244L (0.8 mol%) in KPi buffer (100 mm, pH 8.0) with 5% DMSO as the cosolvent at 30 °C for 24 h, the final total volume is 10 mL. Yield was calculated after silica gel column chromatography purification upon the average of three parallel experiments, *ee* was determined by HPLC on a chiral stationary phase.

Building on the fundamental research on IREDs, we further tested the potential of directly transforming the racemic alcohol into chiral amine via a hydrogen‐borrowing cascade system. This reaction is dependent on the migration of the hydride between alcohol and imine through the NAD(P)^+^/NAD(P)H driven by alcohol dehydrogenase (ADH) or ketone reductase (KRED), and IRED, without external GDH and glucose recycle (**Scheme** **3**A).^[^ ^54^, ^55^, ^56^, ^57^ ^]^ As the oxidation of racemic alcohols requires the use of non‐enantioselective ADH or KRED to convert racemic alcohols, broad screening was conducted. Among 40 enzymes, KRED‐F42^[^ ^58^ ^]^ from *Exiguobacterium sp*. MH3 was found to be effective in convertion between aldehyde and racemic alcohol (see Table S5, Supporting Information), but it was not well‐match with IR‐09 according to poor reactivity and substrate tolerance in the cascade system. Thus, enzyme engineering was executed to improve the reactivity of KRED‐F42. Based on the FRISM strategy,^[^ ^59^, ^60^ ^]^ seven residues among 3 Å within the active site of the enzyme were mutated to evaluate the changes of their performance. Out of the mutants, a potential candidate KRED‐F42‐T244L with excellent racemic alcohol production from aldehyde was obtained (see Table S6, Supporting Information). We further optimized the cascade reaction conditions with IR‐09 and F42‐T244L, including enzyme loading, and concentration of amine and NADP^+^. Finally, the model product (*S*)‐**5h** was obtained with 55% yield (the average of three parallel experiments) and 98% *ee*, demonstrating the success of the atropisomeric biaryl amine production through borrowing hydrogen cascade system. Substrate studies showed that several biaryl skeletons and amine partners could be successfully converted into products with moderate yields and excellent *ee* values (Scheme 3B). Further engineering efforts may be beneficial for higher reaction efficiency in the future.

### 2.5 Mechanism Investigation

To elucidate the mechanism of the DKR process, density functional theory (DFT) calculations were performed to evaluate the rotational barriers of various substrates and their corresponding imine intermediates (**Figure** **3A**). The results revealed that different types of substrates undergo distinct racemization pathways. For the phenol‐nathphlene aldehyde substrate **1a**, the rotational free energy barrier of its hemiacetal form **1a** is 26.2 kcal mol^−1^ at ambient temperature, whereas the rotational barrier of *N,O*‐acetal intermediate formed in situ from **1a** and propargylamine **2a** is significantly lower at 13.3 kcal mol^−1^. This indicates that the labile imine intermediate is favored for the IRED‐catalyzed atroposelective DKR process. Similar findings were observed for the more hindered substrate **1f**, where the rotational barrier of the intermediate is lower than that of the starting material (see Figure S9, Supporting Information for detailed information). For quinoline‐containing biaryl substrates such as **4h**, the transient Lewis pair derived from the substrate has a rotational free energy barrier of 24.9 kcal mol^−1^, while the imine intermediate's rotational barrier is higher at 31.0 kcal mol^−1^. This suggests that the atroposelective synthesis catalyzed by IRED occurs through racemization of the substrate itself. The same racemization mechanism was observed for quinoxaline **4m** and benzofuran‐containing biaryl substrates **4n**, where the rotation barrier of the intermediate is higher than that of the starting material (Figure S9, Supporting Information). However, a different scenario applies to the benzothiophene‐containing biaryl substrate **4o**, where both the substrate and imine intermediate exhibit very high rotational barriers (32.6 and 32.8 kcal mol^−1^, respectively (Figure S9, Supporting Information). This high energy requirement makes the DKR process infeasible, resulting in the low yield of product **5o** via KR.

To elucidate the molecular basis of the high enantioselectivity achieved by imine reductase IR‐09, we conducted docking and molecular dynamics (MD) simulations using imine intermediates derived from representative substrates **1a** and **4h**. Our analysis revealed that IR‐09 features a highly flexible binding pocket (Figure 3B), which accommodates bulky substrates through dynamic structural adaptations. Upon binding of the imine intermediates, significant conformational rearrangements occur in the loop regions (loopα8'α9', loopβ6'β7'), accompanied by rotational adjustments of residues (Y211', M208') at the pocket entrance to create space for the imine complexes (Figure 3B). For the **1a**‐derived imine intermediate, the (*R*)‐**3aI** enantiomer stably occupies the binding pocket, with its hydroxyl group forming water‐mediated hydrogen bonds with the amide moiety of NADPH. In contrast, the (*S*)‐**3aI** enantiomer lacks this stabilizing interaction, as its hydroxyl group is oriented away from NADPH. This disparity results in closer proximity of (*R*)‐**3aI** to NADPH (5.3 Å) compared to (*S*)‐**3aI** (6.4 Å), thereby facilitating a more efficient hydride transfer and rationalizing the observed enantioselectivity (Figure 3C). Similarly, the (*S*)‐**5hI** intermediate binds tightly to NADPH (4.7 Å), stabilized by hydrogen bonds between the NADPH amide group and both the quinoline and imine moieties of (*S*)‐**5hI**. Conversely, the (*R*)‐**5hI** enantiomer is positioned farther from NADPH (6.7 Å) due to the absence of these stabilizing interactions (Figure 3C). Collectively, these computational findings demonstrate that the enantioselectivity was controlled by the specific hydrogen‐bonding interactions between the imine intermediates and the amide group of NADPH (Figure 3D). Hydride attacks from the same direction with the polar functional group for both substrates. But based on the sequence of substitution priority rules, the chirality should be *R* and *S* correspondingly.

Further analysis of the binding pocket residues in the IR‐09‐(*R*)‐**3aI** and IR‐09‐(*S*)‐**5hI** complexes revealed that L171 and Y175 were positioned adjacent to the imine group and its attached aryl ring, respectively. These residues may play a critical role in orienting the imine group toward NADPH, as evidenced by dynamic cross‐correlation analysis (DCCA) (Figure S13, Supporting Information), which demonstrates correlated motions between the imine intermediate and L171/Y175 (correlation coefficient >0.55). Notably, alanine substitution of these residues led to a significant reduction in yields (Figure 3E), further corroborating their essential role. Additionally, D167 was found to form a water‐mediated hydrogen bond network with the imine nitrogen (Figure S14, Supporting Information), suggesting its potential role in facilitating proton transfer during the reduction reaction. This mechanistic insight was supported by the markedly diminished activity observed for the D167A variant. Other surrounding residues, W204', M208', Y211', S229', and Q234', exhibited low correlations (correlation coefficient <0.35) with the imine intermediates and their alanine mutations led to minor effects on reactivity and *ee* (Figure 3E).

## 3 Conclusion

In summary, this study developed a novel bio catalytic synthesis of atropisomeric biaryl amines through a DKR process catalyzed by IRED, which could adapt extremely bulky biaryls including naphthalene, quinoline, or related heteroarenes, building the naphthyl‐aryl amine and quinoline‐aryl amine with excellent yields and stereoselectivities. Based on the highly flexible binding pocket, selected substrates were transformed into biaryl products through the DKR process mediated by IR‐09, which were verified by MD simulations. Atropisomeric biaryl amines with excellent enantioselectivity from racemic alcohols were also been successfully explored according to the borrowing hydrogen cascade of KRED‐F42‐T244L and IR‐09. Our research demonstrated that biocatalysts possess the potential to accept challenging non‐native substrates according to specific inherent characteristics and structural flexibility. Further study encompasses the applications and modifications of enzymes, as well as the bioactivity assessment of biaryl amines, with results to be presented in the near future.

## 4 Experimental Section

A screw vial (20 mL) was charged with GDH (400 µL, 25 mg mL^−1^ stock solution in 100 mm Tris‐HCl buffer pH 8.0), glucose (500 µL, 60 mg mL^−1^ stock solution in 100 mm Tris‐HCl buffer pH 8.0), NADP^+^ (100 µL, 38 mg mL^−1^ stock solution in 100 mm Tris‐HCl buffer pH 8.0), IR‐09 protein (0.2 mol% catalyst loading for preparing **3a–i** and **5a–o** or 0.8 mol% catalyst loading for preparing **6a–i**), aldehyde (500 µL, 100 mm stock in DMSO, 0.05 mmol, 1 equiv.) and amine (8 equiv.). Tris‐HCl buffer (100 mm, pH 8.0) was then added to bring the total volume to 10 mL. The vial was sealed and placed on a shaker at 250 rpm at 37 °C for 24 h. Upon completion, the reaction mixture was transferred to a 50 mL centrifuge tube with 10 mL EtOAc added, and the mixture was centrifugated at 4000 rpm for 5 min (×4). The organic phase was collected and dried over Na_2_SO_4_, followed by filtration and vacuum evaporation. The residue was further purified by column chromatography on silica gel to afford the axially chiral biaryl amines.

## Conflict of Interest

The authors declare no conflict of interest.

## Supporting information



Supporting Information

## Data Availability

The data that support the findings of this study are available in the supplementary material of this article.
