# Supplementary material for: Steric‐Adaptive Biocatalysis: Imine Reductase‐Mediated Dynamic Kinetic Resolution for Atroposelective Synthesis of Hindered Biaryl Amines
Source: Adv Sci (Weinh). 2025 Sep 26;12(45):e10455. doi: 10.1002/advs.202510455 (PMC12677705; doi:10.1002/advs.202510455)
Supplement: Supplementary file 1 — Supporting Information [file ADVS-12-e10455-s001.pdf]

Supporting Information

Steric-Adaptive Biocatalysis: Imine Reductase-Mediated Dynamic Kinetic Resolution for Atroposelective Synthesis of Hindered Biaryl Amines

Zhichao Ni,† Jingyuan Zhuang,† Yadong Gao,† Guangsheng Gao, Zhenling Liu, Ping Su, Gui-Juan Cheng,* and Li-Cheng Yang*

Table of Contents

1.General information 3
Protein and DNA Sequence 4
Protein Expression and Purification 6
PCR-based methods for library construction 6
2.Detailed experimental procedures 7
Screening of imine reductases. 7
Reaction optimization. 8
Screening of alcohol dehydrogenase or ketone reductase. 11
Screening of KRED-F42 variants. 12
Optimisation of biocatalytic hydrogen borrowing 13
Standard curve preparation 17
General procedure for the synthesis of biaryl aldehydes and alcohols 19
General procedure for the IRED catalyzed synthesis of biaryl amines 27
General procedure for biocatalytic hydrogen borrowing 53
Derivatization and application of enzymatic products 58
Crystallographic information 68
Density functional theory (DFT) calculations 70
Molecular dynamics (MD) simulations 99
3.NMR Spectra 103
4.References 166

1.General information

General

Unless otherwise noted, all chemicals and reagents for chemical reactions were obtained from commercial suppliers and used as received (Energy-Chemical, Konoscience, Admas, Bidepharm). Flash column chromatography was performed over silica gel (230-400 mesh). NMR spectra were recorded on JEOL (500 MHz) and Quantum-1 (400 MHz) instrument at room temperature, Chemical shifts (δ) were reported in parts per million (ppm) relative to residual solvent peaks rounded to the nearest 0.01 for proton and 0.1 for carbon. Coupling constants (J) were reported in Hz to the nearest 0.1 Hz. Peak multiplicity was indicated as follows s (singlet), d (doublet), t (triplet), q (quartet), m (multiplet) and br (broad). Attribution of peaks was done using the multiplicities and integrals of the peaks. High-resolution mass spectra (HRMS) were obtained on a Thermo Exactive Plus using electrospray ionization time-of-flight (ESI-TOF). Analytical chiral HPLC was carried out using a Shimadzu instrument equipped with ChiralPak AS-H, AD-H, OD-H, IA, IC (4.6 × 250 mm, 5 μm) columns with isopropanol and hexane as the mobile phase.

pET28a (+) was used as a cloning and expression vector for all enzymes described in this study. Genes (codon optimized for expression in *E. coli*) for all imine-reductases (IREDs) and alcohol dehydrogenases (ADHs) were purchased from SynbioB (Tianjin, China). All constructs were cloned directly between the *NdeI* and *XhoI* restriction sites. Cloned plasmids were transformed into *E. coli* DH5α cells for storage, and *E. coli* BL21 (DE3) chemically competent cells for expression. Glucose dehydrogenase (GDH) was purchased from microBioSyn, CAS: 9028-52-9.

Protein and DNA Sequence

IR-09 protein sequence

MSSVSIFGLGAMGTALASRFLEEKYKVAVWNRSPEKASPLLEKGATLSHTALDGINASDLI
VICLLDNAAVQATLNSALEHLRGKTIINLTNGTPDQARKLSDLIVSHGAQYVHGGIMATPS
MIGSPHALVLYSGSPDAFKTAEADLSVLAKCIFLGEDAGSASLHDLALLSGMYGLFSGFLH
ATALVRSSTPAVKFVDLLVPWLGAMTEYTKGMAKQIDEGNYASEGSNLGMQLVAIQNIID
ASAAQQVSADFIRPMKEFMEKAVVAGHGGDDISSLIDFVKST

IR-09 gene sequence

ATGTCGAGCGTAAGTATCTTCGGCTTGGGTGCTATGGGAACGGCCTTGGCTTCGCGCTT
TCTGGAGGAGAAGTACAAAGTTGCCGTGTGGAACCGTAGTCCGGAAAAGGCGTCGCC
GTTACTGGAGAAAGGTGCCACGTTAAGCCATACTGCCTTGGACGGGATCAATGCCTCG
GATTTAATTGTGATTTGTCTTTTAGATAACGCCGCAGTTCAAGCGACCCTGAACAGCGC
ACTGGAACACTTACGTGGTAAAACTATCATCAATCTGACGAATGGCACTCCTGACCAA
GCACGCAAACTTAGCGACTTGATTGTCTCACACGGAGCCCAATACGTGCACGGAGGAA
TTATGGCCACGCCTTCTATGATTGGTTCACCACATGCATTAGTTTTATATAGCGGTTCTCC
AGATGCGTTCAAAACGGCGGAAGCCGACCTTTCAGTGTTGGCAAAATGCATTTTCCTG
GGTGAGGATGCTGGCTCCGCTTCGCTGCATGATCTTGCGCTTCTTAGCGGAATGTATGG
CTTATTCTCTGGATTCCTGCATGCTACAGCGTTAGTGCGCTCGTCAACCCCCGCGGTCA
AATTTGTGGATCTTCTGGTACCTTGGCTTGGAGCAATGACAGAGTACACAAAGGGAAT
GGCTAAGCAAATTGACGAAGGTAATTACGCCTCTGAAGGTTCCAATTTGGGTATGCAAT
TGGTCGCTATCCAGAACATCATCGATGCAAGTGCAGCGCAGCAAGTAAGTGCCGACTT
TATCCGTCCGATGAAAGAGTTTATGGAAAAAGCTGTTGTTGCGGGCCACGGCGGGGAC
GATATTTCGAGCCTTATTGACTTCGTGAAAAGCACCTGA

KRED-F42 protein sequence

MKYTVITGASSGIGYETAKLLAGKGKSLVLVARRTSELEKLRDEVKQISPDSDVILKSVDL
ADNQNVHDLYEGLKELDIETWINNAGFGDFDLVQDIELGKIEKMLRLNIEALTILSSLFVR
DHHDIEGTTLVNISSAGGYRIVPNAVTYCATKFYVSAYTEGLAQELQKGGAKLRAKVLAP
AATETEFADRSRGEAGFDYSKNVKKYHTAAEMAGFLHQLIESDAIVGIVDGETYEFELRG
PLFNYAG

KRED-F42 gene sequence

ATGAAGTACACGGTCATTACAGGAGCAAGTTCAGGAATTGGATATGAGACAGCAAAAC
TACTCGCAGGAAAAGGAAAATCACTCGTCCTCGTCGCACGGCGGACGTCTGAGCTCG
AAAAACTTCGGGATGAAGTCAAACAAATCTCACCAGATAGTGATGTCATCCTCAAGTC
GGTCGATCTCGCAGATAACCAAAATGTCCATGATTTATATGAGGGACTAAAGGAACTCG
ACATCGAGACGTGGATCAACAATGCTGGATTCGGCGATTTTGATCTCGTCCAGGACATT
GAGCTCGGGAAAATCGAGAAAATGCTCCGCTTGAACATCGAGGCGCTGACGATTCTAT
CGAGTCTGTTCGTCCGCGATCATCATGACATCGAAGGAACGACACTCGTCAATATCTCG
TCAGCAGGTGGCTACCGGATCGTTCCGAACGCGGTCACGTATTGCGCGACGAAGTTCT
ATGTCAGTGCCTATACGGAAGGGCTAGCGCAAGAACTGCAAAAAGGCGGGGCAAAAC
TCCGGGCGAAAGTACTGGCACCAGCTGCGACTGAGACAGAGTTTGCGGATCGTTCGC
GCGGCGAAGCAGGGTTCGACTACAGCAAGAACGTCAAAAAGTACCATACGGCGGCTG
AGATGGCAGGCTTCTTGCATCAGTTGATCGAAAGTGACGCGATCGTCGGCATCGTCGA
CGGTGAGACGTATGAGTTCGAATTGCGTGGTCCGTTGTTCAACTACGCAGGATAA

Protein Expression and Purification

Imine reductase from *Aspergillus lentulus* was produced in *E. coli* BL21 with a plasmid encoding IR-09. Ketone reductase from *Exiguobacterium sp.* MH3 was produced in *E. coli* BL21 with a plasmid encoding KRED-F42.Transformed glycerol stocks were used to initiate a 5 mL overnight culture in LuriaBertani (LB) media with kanamycin (50 μg/mL) at 37 °C and 250 rpm. Expression culture (500 mL in a 2 L baffled shake flask) containing kanamycin (50 μg/mL) was inoculated with 5 mL of the overnight culture and grown until the culture reached an OD600 of 0.6 ~ 0.8 (37 °C, 250 rpm). Flasks were chilled on ice and protein expression was induced with 0.2 mM IPTG (20 °C, 20 h, 200 rpm). The cultures were centrifuged at 4,000×*g* and 4 °C for 15 minutes. The cell pellets were decanted, then frozen and stored at –80 °C until further use.

For protein purification, cells were thawed, and re-suspended in potassium phosphate buffer (100 mM, pH 8.0), and then lysed with a JY92-IIDN Ultrasonic Homogenizer (Scientz). Lysate were then centrifuged at 15,000×*g* and 4 °C for 35 minutes to collect supernatant. Proteins were then extracted and purified by immobilized metal affinity chromatography a linear gradient from wash buffer (50 mM Tris-HCl, 20 mM imidazole, 300 mM NaCl, pH 8.0) to elution buffer (50 mM Tris-HCl, 500 mM imidazole, 100 mM NaCl, pH 8.0). Elution fractions containing the enzymes were desalted by Amicon Ultra centrifugal filters with 10k molecular retention (Millipore) into a final buffer solution of 100 mM Tris-HCl buffer (pH 8.0). Then flash-frozen in liquid N_2 and stored at –80 °C until further use. Protein concentrations were determined *via* the Bradford assay (Bio-Rad).

PCR-based methods for library construction

KOD One™ PCR Master MIX was used to construct mutant libraries. 15 μL ddH_2O, 20 μL KOD One™ PCR Master MIX, 2 μL each of 10 μM primers mix and 1 μL (5 -10 ng) template DNA were added make a total volume of 40 μL reaction mixture. The PCR cycles used for generating short fragment were as follows: 98 °C for 30 s, (98 °C for 15 s, Tm-5 °C for 5 s, 72 °C for 1 min) × 25 cycles, 72 °C for 3 min, 4 °C. The PCR products were analyzed by electrophoresis on agarose gel. The digestion was carried out at 37 °C for more than 3 h by adding *DpnI* in PCR reaction mixture. After digestion, the PCR products were used to directly transform *E. coli* BL21 (DE3) chemically competent cells and plated on LB agar plates containing kanamycin (50 μg/mL).

2.Detailed experimental procedures

SI Table 1. Screening of imine reductases.

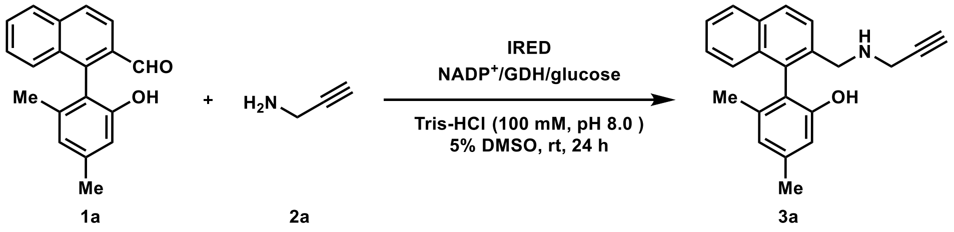

Entry	Enzymes	Accession number	Source	Yield (%)[a]	*ee* (%)[b]
1	IR-09	8QHE_A	*Aspergillus lentulus*	32	96 (*R*)
2	IR-16	XP_043147525.1	*Aspergillus udagawae*	16	95 (*R*)
3	IR-85	WP_003986432.1	*Streptomyces*	<5	75 (*S*)
4	IR-104	WP_124961062.1	*Variovorax beijingensis*	<5	65 (*R*)
5	IR-114	WP_307647705.1	*Variovorax boronicumulans*	<5	93 (*R*)
6	IR-202	KAF4266800.1	*Aspergillus fumigatus*	<5	68 (*R*)
7	IR-351	WP_030601469.1	*Streptomyces rimosus*	<5	68 (*S*)
8	IR-356	WP_030568324.1	*Streptomyces cyaneofuscatus*	<5	86 (*S*)

Reaction conditions: **1a** (5 μmol, 1 equiv.), propargylamine **2a** (10 μmol, 2 equiv.), $NADP^+$ (0.5 μmol, 10 mol%), glucose (15 μmol, 3 equiv.), GDH (1 mg/mL), lysates of IRED in Tris-HCl buffer (100 mM, pH 8.0) with 5% DMSO as cosolvent at rt for 24 h, the final total volume is 1 mL. [a] Yield was determined via HPLC relative to an internal standard 1,3,5-trimethoxybenzene. [b] Enantiomeric excess (*ee*) was determined by HPLC on a chiral stationary phase.

SI Table 2. Reaction optimization.

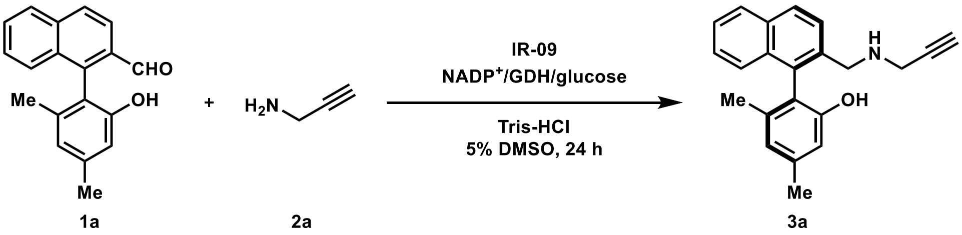

Entry	Amine (equiv.)	pH	Temperature (°C)	Yield (%)[a]	*ee* (%)[b]
1	2	8.0	37	52	96
2	2	8.5	37	34	97
3	2	9.0	37	28	97
4	2	9.5	37	22	98
5	2	8.0	30	26	93
6	4	8.0	37	65	94
7	6	8.0	37	64	94
8	8	8.0	37	70	96
9	10	8.0	37	65	94
10	15	8.0	37	55	93
11	20	8.0	37	57	92
12	30	8.0	37	51	96

Reaction conditions: **1a** (5 μmol, 1 equiv.), propargylamine **2a**, $NADP^+$ (0.5 μmol, 10 mol%), glucose (15 μmol, 3 equiv.), GDH (1 mg/mL), purified IR-09 (1 mol%) in Tris-HCl buffer (100 mM) with 5% DMSO as cosolvent for 24 h, the final total volume is 1 mL. [a] Yield was determined via HPLC relative to an internal standard 1,3,5-trimethoxybenzene. [b] Enantiomeric excess (*ee*) was determined by HPLC on a chiral stationary phase.

SI Table 3. Reductive amination of **1a** with **2a** with IR-09 variants.

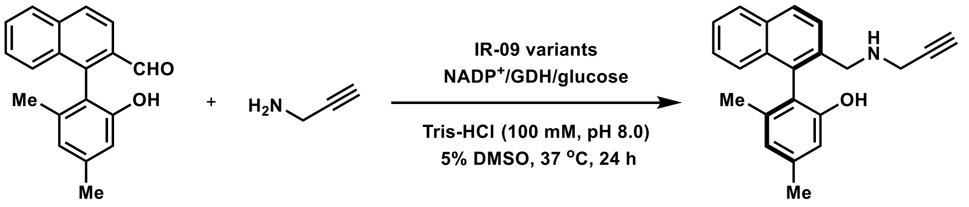

1a 2a 3a

Entry	Variants	Yield (%)[a]	*ee* (%)[b]
1	WT	82	96
2	Y211A	55	93
3	Q234A	69	92
4	D167A	0	n.d.[c]
5	M233A	0	n.d.
6	L171A	46	60
7	S229A	72	90
8	Y175A	0	n.d.
9	D274A	58	84
10	M208A	99	95
11	F178A	78	85
12	W204A	60	93
13	M174A	70	99

Reaction conditions: **1a** (5 μmol, 1 equiv.), propargylamine **2a** (40 μmol, 8 equiv.), $NADP^+$ (0.5 μmol, 10 mol%), glucose (15 μmol, 3 equiv.), GDH (1 mg/mL), lysates of IR-09 variants in Tris-HCl buffer (100 mM, pH 8.0) with 5% DMSO as cosolvent at 37 °C for 24 h, the final total volume is 1 mL. [a] Yield was determined via HPLC relative to an internal standard 1,3,5-trimethoxybenzene. [b] Enantiomeric excess (*ee*) was determined by HPLC on a chiral stationary phase. [c] n.d., not determined.

SI Table 4. Reductive amination of **4h** with **2a** with IR-09 variants.

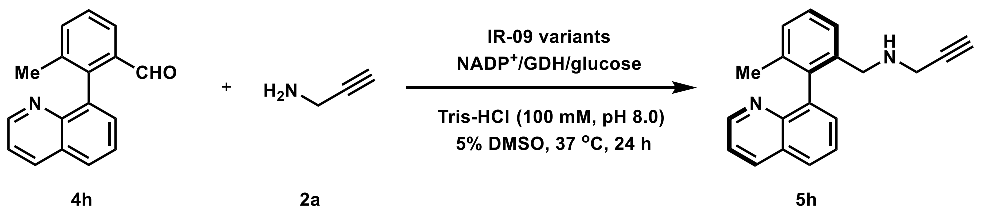

Entry	Variants	Yield (%)[a]	*ee* (%)[b]
1	WT	99	98
2	Y211A	99	99
3	Q234A	99	86
4	D167A	32	93
5	M233A	0	n.d.[c]
6	L171A	15	48
7	S229A	85	87
8	Y175A	7	98
9	D274A	74	97
10	M208A	97	98
11	F178A	99	99
12	W204A	99	89
13	M174A	68	98

Reaction conditions: **4h** (5 μmol, 1 equiv.), propargylamine **2a** (40 μmol, 8 equiv.), $NADP^+$ (0.5 μmol, 10 mol%), glucose (15 μmol, 3 equiv.), GDH (1 mg/mL), lysates of IR-09 variants in Tris-HCl buffer (100 mM, pH 8.0) with 5% DMSO as cosolvent at 37 °C for 24 h, the final total volume is 1 mL. [a] Yield was determined via HPLC relative to an internal standard 1,3,5-trimethoxybenzene. [b] Enantiomeric excess (*ee*) was determined by HPLC on a chiral stationary phase. [c] n.d., not determined.

SI Table 5. Screening of alcohol dehydrogenase or ketone reductase.

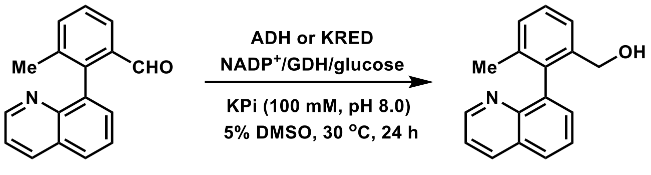

Enzymes	Accession number	Source	Yield (%)[a]	*ee* (%)[b]
YDR368W	NP_010656	*Saccharomyces cerevisiae*	11	18 (*S*)
KRED-F42	WP_023468191.1	*Exiguobacterium sp.* MH3	88	0
KRED-Bt	WP_103592444.1	*Bacillus thuringiensis*	81	78 (*S*)
KmCR2	XP_022675166.1	*Kluyveromyces marxianus CBS4857*	5	31 (*S*)
YAL060W	NP_009341.2	*Saccharomyces cerevisiae*	40	22 (*R*)
ADH-R1	WP_013688875.1	*Burkholderia gladioli*	23	32 (*S*)
ADH-R2	EU485985.1	*Ralstonia sp.*	99	70 (*S*)

Reaction conditions: **4h** (5 μmol, 1 equiv.), $NADP^+$ (0.5 μmol, 10 mol%), glucose (15 μmol, 3 equiv.), GDH (1 mg/mL), lysates of ADH or KRED in KPi buffer (100 mM, pH 8.0) with 5% DMSO as cosolvent at 30 °C for 24 h, the final total volume is 1 mL. [a] Yield was determined via HPLC relative to an internal standard 1,3,5-trimethoxybenzene. [b] Enantiomeric excess (*ee*) was determined by HPLC on a chiral stationary phase.

SI Table 6. Screening of KRED-F42 variants.

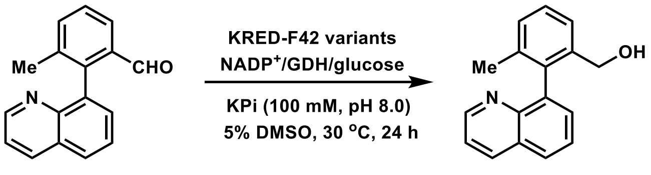

Entry	Variants	Yield (%)[a]	*ee* (%)[b]
1	WT	88	0
2	P216F	97	88 (*R*)
3	Y242F	94	12 (*S*)
4	T244F	91	5 (*S*)
5	M248F	94	17 (*S*)
6	Y270F	95	11 (*R*)
7	A217F	96	23 (*S*)
8	A219F	33	27 (*S*)
9	P216L	43	17 (*S*)
10	Y242L	99	51 (*S*)
11	T244L	99	0
12	M248L	95	12 (*S*)
13	Y270L	84	13 (*R*)
14	A217L	88	22 (*S*)
15	A219L	97	62 (*S*)

Reaction conditions: **4h** (5 μmol, 1 equiv.), $NADP^+$ (0.5 μmol, 10 mol%), glucose (15 μmol, 3 equiv.), GDH (1 mg/mL), lysates of KRED-F42 variants in KPi buffer (100 mM, pH 8.0) with 5% DMSO as cosolvent at 30 °C for 24 h, the final total volume is 1 mL. [a] Yield was determined via HPLC relative to an internal standard 1,3,5-trimethoxybenzene. [b] Enantiomeric excess (*ee*) was determined by HPLC on a chiral stationary phase.

Optimisation of biocatalytic hydrogen borrowing

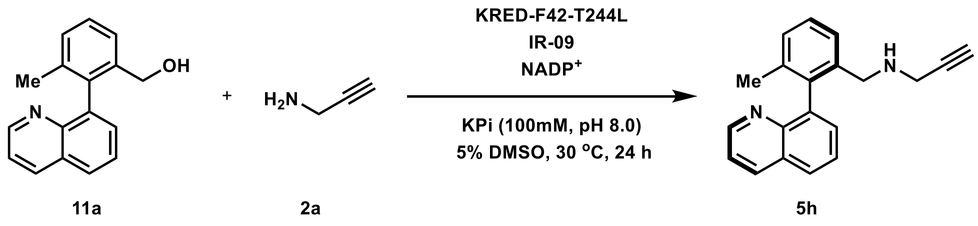

SI Figure 1. IR-09 loading evaluation.

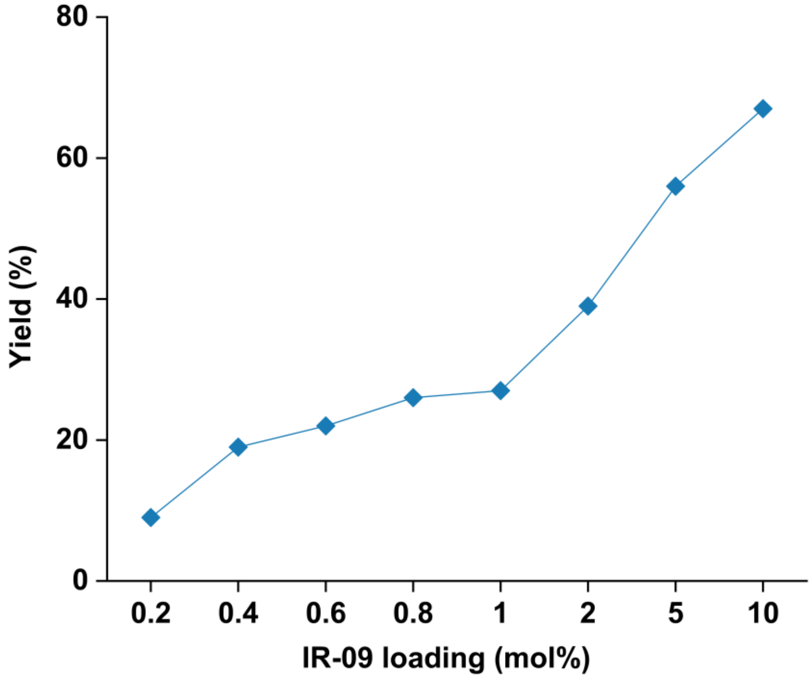

Reaction conditions: **11a** (5 µmol, 1 equiv.), $NADP^+$ (0.5 µmol, 10 mol%), propargylamine **2a** (40 µmol, 8 equiv.), purified KRED-F42-T244L (1 mol%) and IR-09 in KPi buffer (100 mM, pH 8.0) with 5% DMSO as cosolvent at 30 °C for 24 h, the final total volume is 1 mL. Yield was determined via HPLC relative to an internal standard 1,3,5-trimethoxybenzene.

SI Figure 2. KRED-F42-T244L loading evaluation.

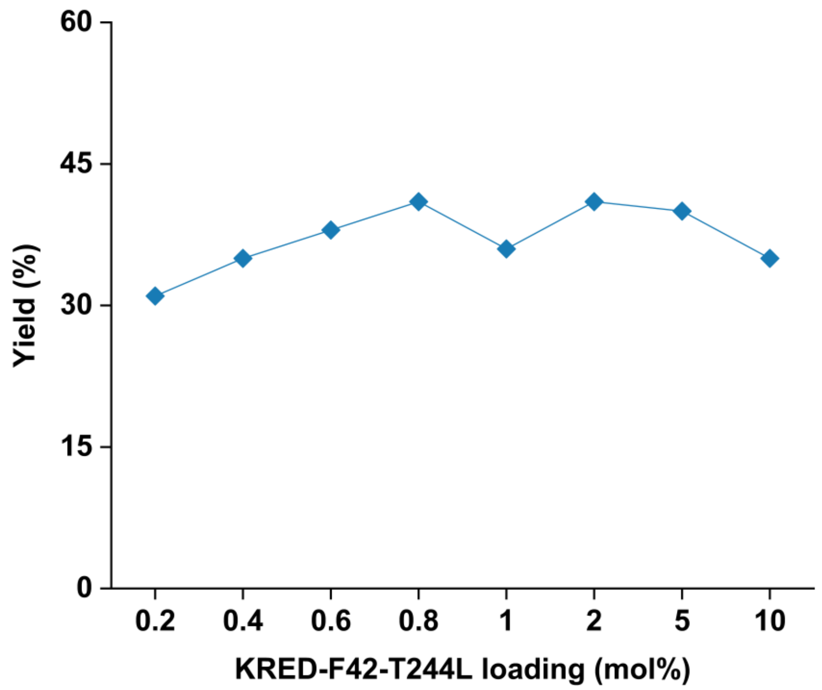

Reaction conditions: **11a** (5 μmol, 1 equiv.), $NADP^+$ (0.5 μmol, 10 mol%), propargylamine **2a** (40 μmol, 8 equiv.), purified KRED-F42-T244L and IR-09 (2 mol%) in KPi buffer (100 mM, pH 8.0) with 5% DMSO as cosolvent at 30 °C for 24 h, the final total volume is 1 mL. Yield was determined via HPLC relative to an internal standard 1,3,5-trimethoxybenzene.

SI Figure 3. Amine concentration.

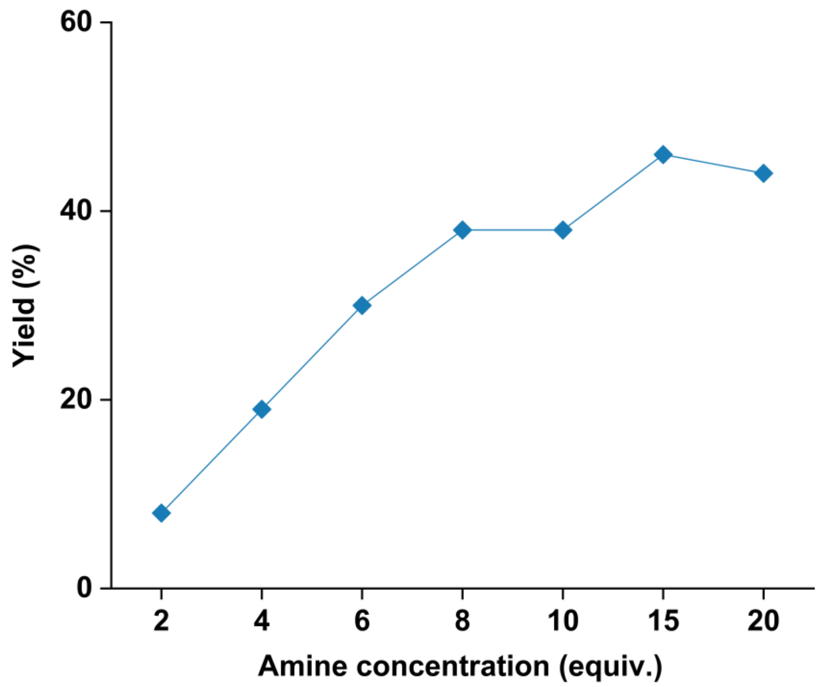

Reaction conditions: **11a** (5 μmol, 1 equiv.), $NADP^+$ (0.5 μmol, 10 mol%), propargylamine **2a**, purified KRED-F42-T244L (0.8 mol%) and IR-09 (2 mol%) in KPi buffer (100 mM, pH 8.0) with 5% DMSO as cosolvent at 30 °C for 24 h, the final total volume is 1 mL. Yield was determined via HPLC relative to an internal standard 1,3,5-trimethoxybenzene.

SI Figure 4. NADP$^+$ concentration.

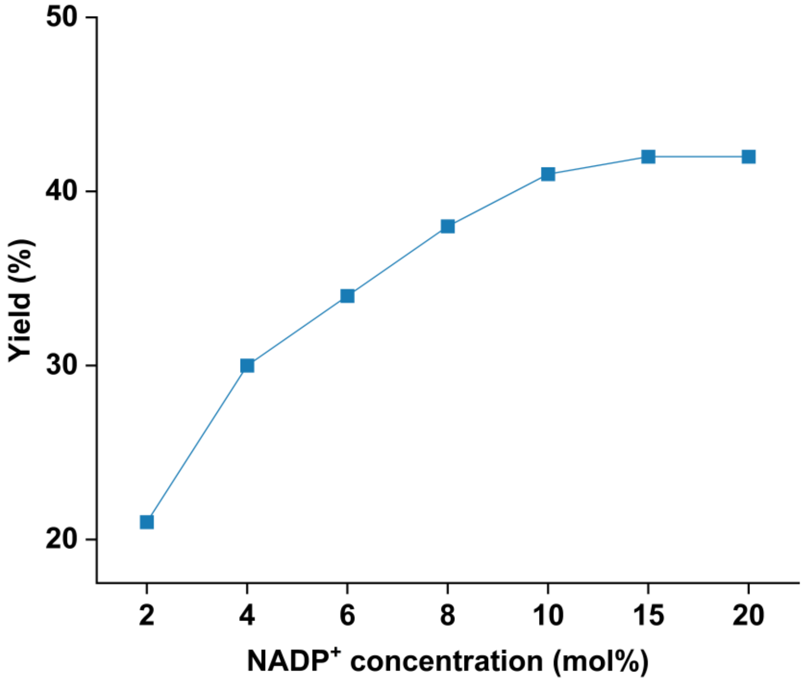

Reaction conditions: **11a** (5 µmol, 1 equiv.), NADP$^+$, propargylamine **2a** (40 µmol, 8 equiv.), purified KRED-F42-T244L (0.8 mol%) and IR-09 (2 mol%) in KPi buffer (100 mM, pH 8.0) with 5% DMSO as cosolvent at 30 °C for 24 h, the final total volume is 1 mL. Yield was determined via HPLC relative to an internal standard 1,3,5-trimethoxybenzene.

Standard curve preparation

SI Figure 5. HPLC calibration curve for **3a**.

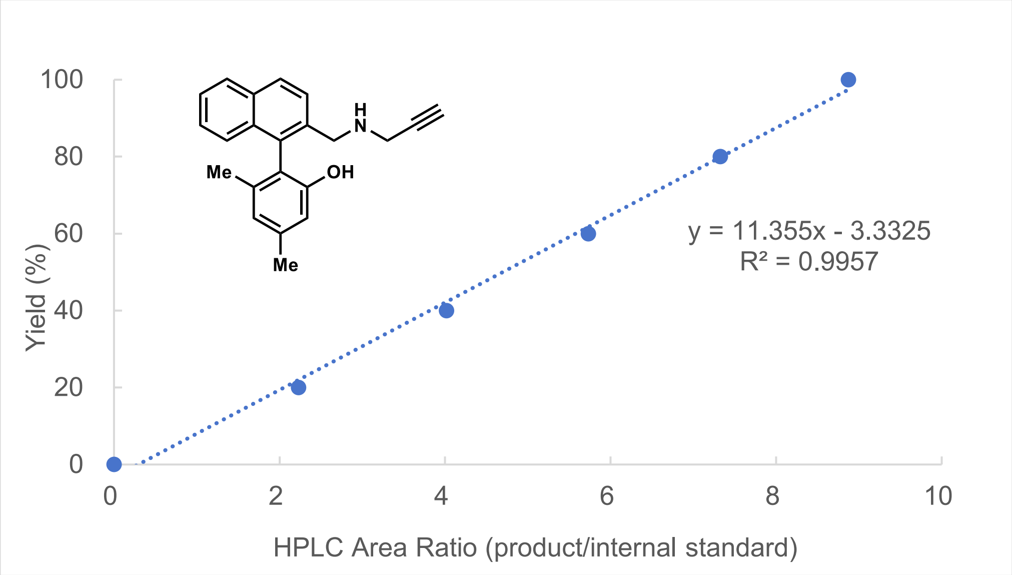

SI Figure 6. HPLC calibration curve for **5h**.

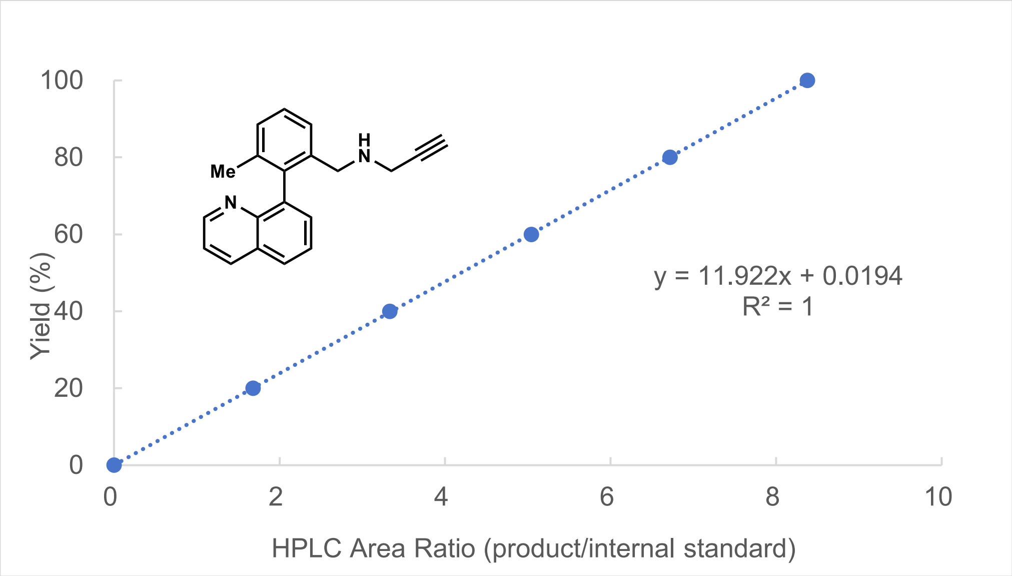

SI Figure 7. HPLC calibration curve for **11a**.

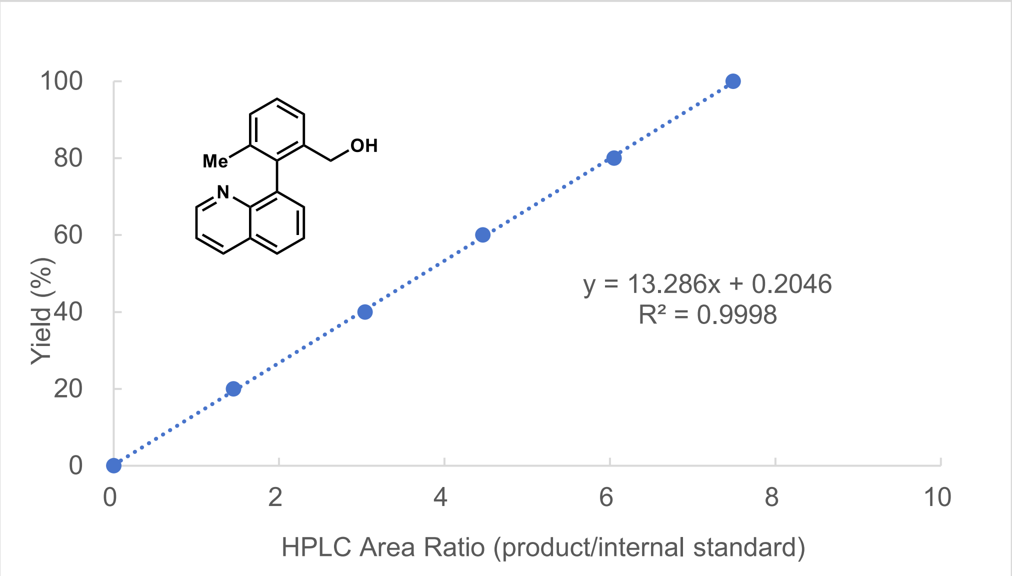

General procedure for the synthesis of biaryl aldehydes and alcohols

List of biaryl aldehydes and alcohols

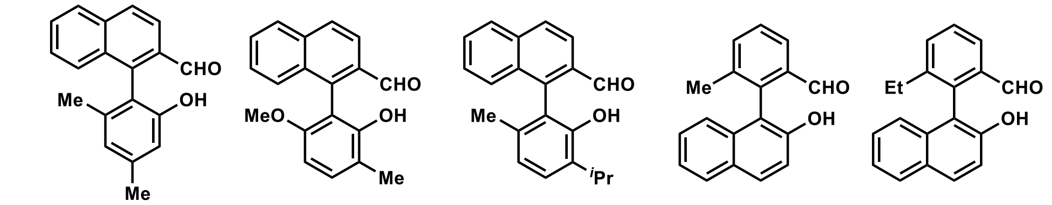

1a **1b** **1c** **1d** **1e**

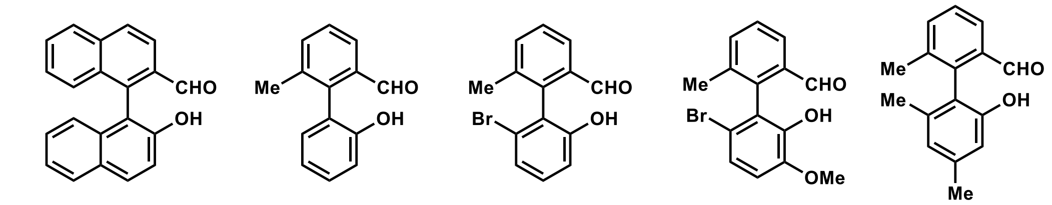

1f **1g** **1h** **1i** **1j**

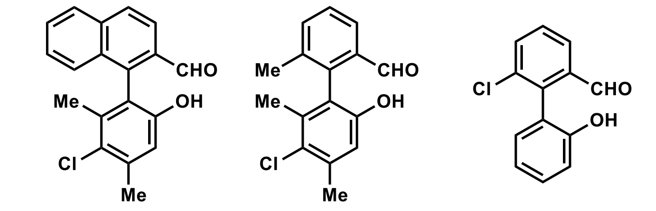

1k **1l** **1m**

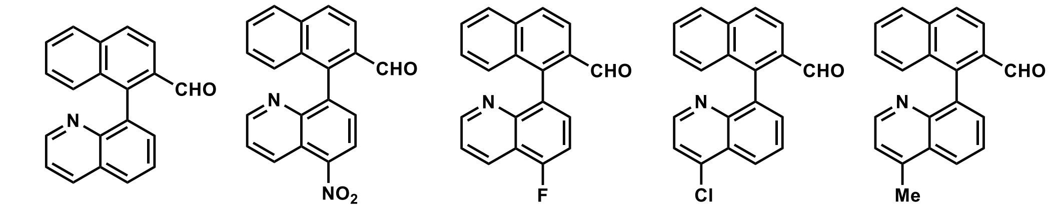

4a **4b** **4c** **4d** **4e**

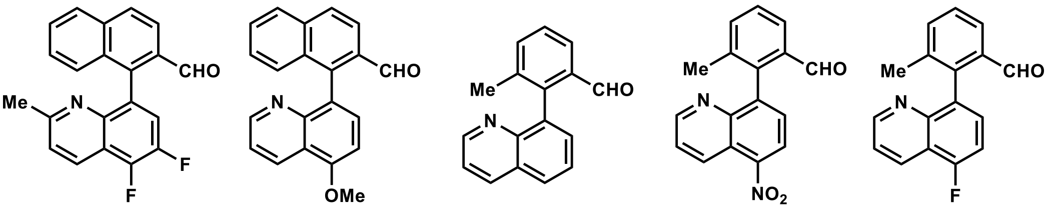

4f **4g** **4h** **4i** **4j**

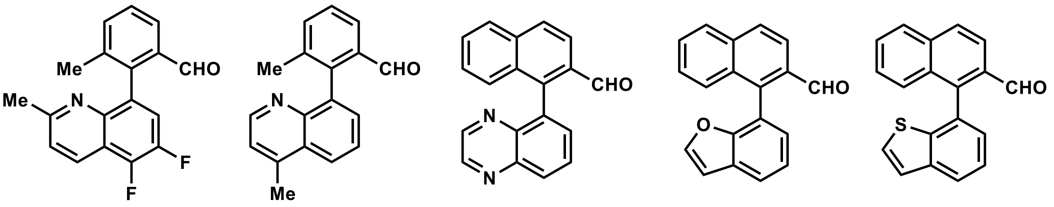

4k **4l** **4m** **4n** **4o**

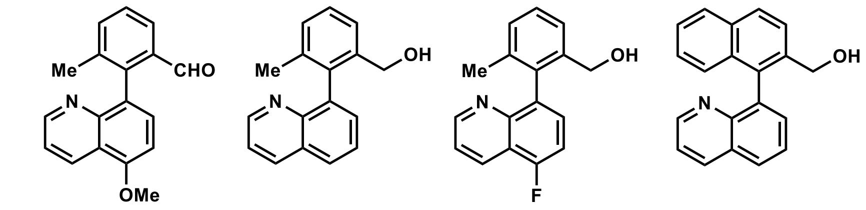

4p **11a** **11b** **11c**

Procedure for the Synthesis of **1a-c**,**1f** and **1j-l**

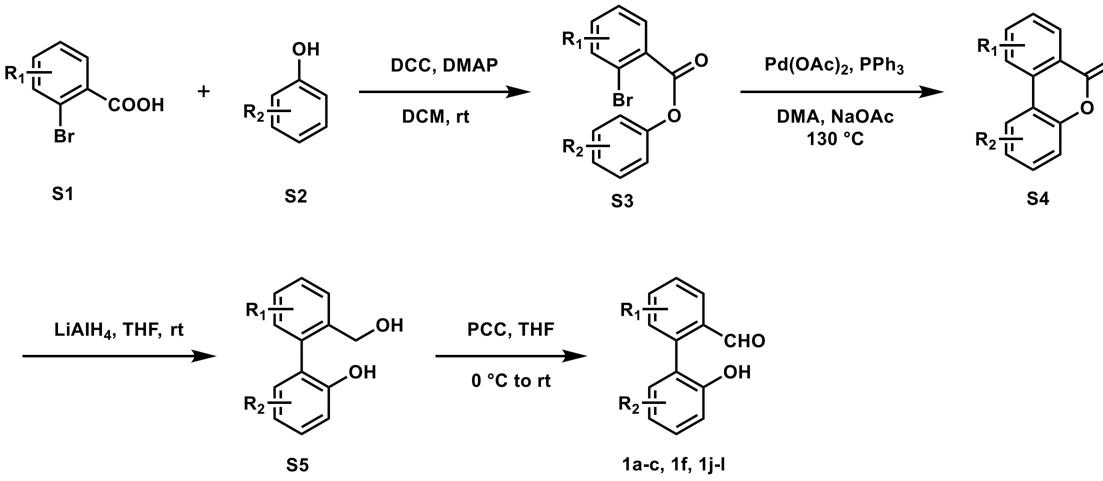

Substrates **1a-c**, **1f** and **1j-l** were synthesized according to the reported procedure[1,2]: To a solution of carboxylic acid **S1** (1 equiv.) in 50 mL dry DCM, phenol **S2** (1.1 equiv.), DMAP (0.1 equiv.) and DCC (3 equiv.) were successively added. The resulting solution was allowed to stir at room temperature for 4 h. The reaction mixture was filtered through celite to remove insoluble solids. The filtrate was evaporated and the residue was subjected to column chromatography on silica gel (typical eluent: PE:EtOAc = 60:1) to afford the ester **S3** as colorless oil.

A mixture of the ester **S3** (1 equiv.), $Pd(OAc)_2$ (0.1 equiv.), PPh_3 (0.2 equiv.), and NaOAc (2 equiv.) was added to DMA under N_2, and the reaction mixture was heated to 130 °C for 18 h. After removal of the solvent in vacuo, the residue was further purified by column chromatography on silica gel (typical eluent: PE:EtOAc = 80:1) to afford the lactones **S4** as slightly yellow solid.

To a solution of the lactone **S4** in THF (5 mL/mmol **S4**), $LiAlH_4$ (2 equiv.) were added in portions at 0 °C. After 2 h of stirring at room temperature, the reaction mixture was quenched carefully with water (5 mL/mmol **S4**), slightly acidified with 2N HCl, extracted with EtOAc (5 mL/mmol **S4**), and dried over Na_2SO_4. The solvent was removed in vacuo and the residue was further purified by column chromatography on silica gel (typical eluent: PE:EtOAc = 3:1) to afford the alcohol **S5**.

To a solution of the alcohol **S5** in THF (5 mL/mmol **S5**), PCC (2.0 equiv.) and 1g of silica gel were added in portions at 0 °C. After stirring at room temperature for 0.5 - 2.0 h, 1g of silica gel was added and the sample was dried by vacuum, and the residue was further purified by column chromatography on silica gel (typical eluent: PE:EtOAc = 10:1) to afford the pure hydroxy aldehyde **1a-c**, **1f** and **1j-l**.

Procedure for the Synthesis of **1d-e** and **1h-i**

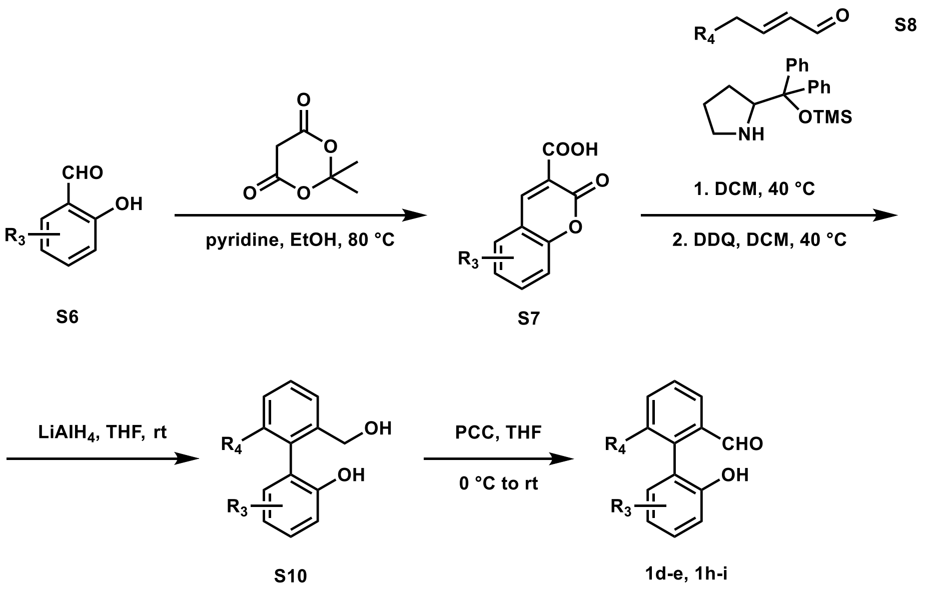

Substrates **1d-e** and **1h-i** were synthesized according to the reported procedure[1,2]: To a solution of Meldrum's acid (1 equiv.) in ethanol, aldehyde **S6** (1 equiv.) and a catalytic amount of pyridine were added. The reaction mixture was stirred at 80 °C for 4 h. The mixture was allowed to cool to room temperature before being stirred at 0 °C for another hour. The solid which precipitated out of solution was filtered off, washed thoroughly with ethanol and dried in vacuo to afford the desired acid **S7**.

To a solution of **S7** (1 equiv.) in DCM, aldehyde **S8** (1.5 equiv.) and catalyst (rac)-2-(diphenyl(trimethylsilyloxy)methyl)pyrrolidine (0.2 equiv.) were added. The reaction mixture was allowed to stir at 40 °C for 24 h, followed by addition of DDQ (2 equiv.). After 12 h, the reaction mixture was cooled to room temperature and filtered through celite to remove insoluble solids. The filtrate was evaporated in vacuo and the residue was subjected to column chromatography on silica gel (typical eluent: PE:EtOAc = 80:1) to afford lactones **S9**.

To a solution of the lactone **S9** in THF (5 mL/mmol **S9**), $LiAlH_4$ (2.0 equiv.) were added in portions at 0 °C. After 2 h of stirring at room temperature, the reaction mixture was quenched carefully with water (5 mL/mmol **S9**), slightly acidified with 2N HCl, extracted with EtOAc (5 mL/mmol **S9**), and dried over Na_2SO_4. The solvent was removed in vacuo and the residue was further purified by column chromatography on silica gel (typical eluent: PE:EtOAc = 3:1) to afford the alcohol **S10**.

To a solution of the alcohol **S10** in THF (5 mL/mmol **S10**), PCC (2.0 equiv.) and 1g of silica gel were added in portions at 0 °C. After stirring at room temperature for 0.5 - 2.0 h, 1g of silica gel was added and the sample was dried by vacuum, and the residue was further purified by column chromatography on silica gel (typical eluent: PE:EtOAc = 10:1) to afford the pure hydroxy aldehyde **1d-e** and **1h-i**.

Procedure for the Synthesis of **1g** and **1m**

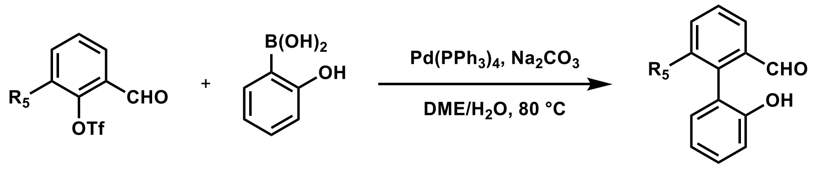

S11 **S12** **1g, 1m**

Substrates **1g** and **1m** were synthesized according to the reported procedure[3,4]: To a solution of **S11** (1 equiv.) in DME/H_2O (4:1 *v/v*) was added boronic acid **S12** (1.5 equiv.), $Pd(PPh_3)_4$ (0.1 equiv.), Na_2CO_3 (3 equiv.), the reaction mixture was heated at 80 °C overnight. After completion, the reaction mixture was diluted with EtOAc and brine, and extracted with EtOAc. The combined organic phases were dried over Na_2SO_4, filtered and removed in vacuo. The residue was further purified by column chromatography on silica gel (PE:EtOAc = 7:1) to afford the hydroxy aldehyde **1g** and **1m**.

Procedure for the Synthesis of **4a-l** and **4p**

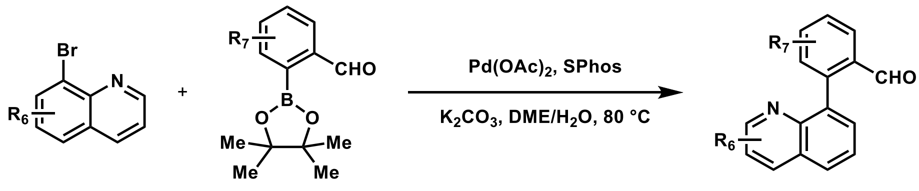

S13 **S14** **4a-l, 4p**

Substrates **4a-l** and **4p** were synthesized according to the reported procedure[5]: To a solution of the corresponding 8-bromoquinoline **S13** (1 equiv.) in DME/H_2O (4:1 *v/v*) was added borate ester **S14** (1.2 equiv.), $Pd(OAc)_2$ (0.05 equiv.), SPhos (0.05 equiv.), K_2CO_3 (4.0 equiv.) under N_2 atmosphere. The reaction mixture was stirred overnight at 80 ºC. After completion, the reaction mixture was diluted with EtOAc and brine, and extracted with EtOAc. The combined organic phases were dried over Na_2SO_4, filtered and removed in vacuo. The residue was further purified by column chromatography on silica gel (PE:EtOAc = 5:1) to afford the desired quinolyl aldehyde **4a-l** and **4p**.

Procedure for the Synthesis of **4n-o**

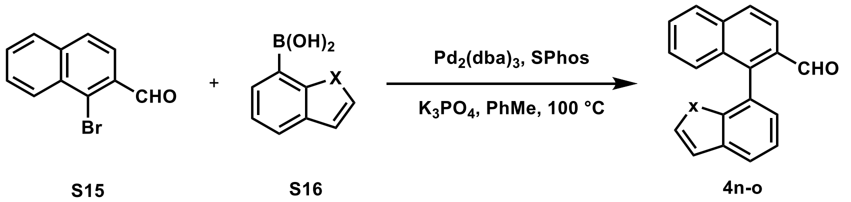

S15 **S16** **4n-o**

To a solution of the 1-bromo-2-naphthaldehyde **S15** (1 equiv.) in PhMe was added boronic acid **S16** (1.2 equiv.), $Pd_2(dba)_3$ (0.05 equiv.), SPhos (0.05 equiv.), K_3PO_4 (4.0 equiv.) under N_2 atmosphere. The reaction mixture is stirred overnight at 100 ºC. After completion, the reaction mixture was diluted with EtOAc and brine, and extracted with EtOAc. The combined organic phases were dried over Na_2SO_4, filtered and removed in vacuo. The residue was further purified by column chromatography on silica gel (PE:EtOAc = 5:1) to afford the desired aldehyde **4n-o**.

Procedure for the Synthesis of **4m**

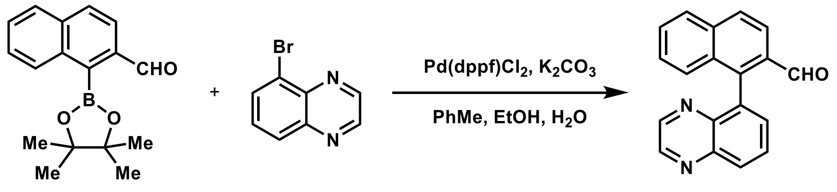

S17 S18 4m

To a solution of the 15-bromoquinoxaline **S18** (1 equiv.) in PhMe/EtOH/H_2O (2:1:1 *v/v*) was added borate ester **S17** (1.2 equiv.), Pd(dppf)Cl_2 (0.05 equiv.), K_2CO_3 (4.0 equiv.) under N_2 atmosphere. The reaction mixture was stirred overnight at 100 ºC. After completion, the reaction mixture was diluted with EtOAc and brine, and extracted with EtOAc. The combined organic phases were dried over Na_2SO_4, filtered and removed in vacuo. The residue was further purified by column chromatography on silica gel (PE:EtOAc = 5:1) to afford the desired aldehyde **4m**.

Procedure for the Synthesis of **11a-c**

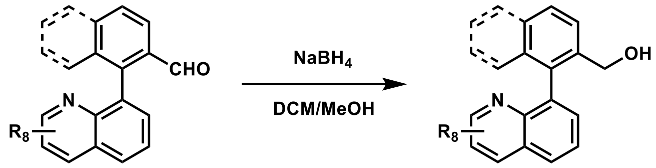

4a, 4h, 4j 11a-c

Substrates **11a-c** were synthesized according to the reported procedure[6]: To a solution of the corresponding aldehyde **4a**, **4h**, **4j** (1 equiv.) in a mixture 1:1 DCM/MeOH, $NaBH_4$ (2 equiv.) was added at 0 ºC. The reaction mixture was stirred at room temperature to the complete consumption of the starting aldehyde (followed by TLC). After completion, the reaction mixture was diluted with EtOAc and brine, and extracted with EtOAc. The combined organic phases were dried over Na_2SO_4, filtered and removed in vacuo. The residue was further purified by column chromatography on silica gel (PE:EtOAc = 3:1) to afford the corresponding racemic alcohols **11a-c**.

Characterization data of unknown biaryl aldehydes substrates

1-(5-nitroquinolin-8-yl)-2-naphthaldehyde (**4b**)

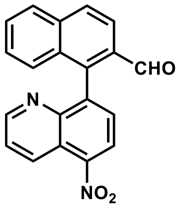

Yellow solid. 90% yield.

^{1}H NMR (400 MHz, CDCl$_3$): δ 9.65 (s, 1H), 9.11 (dd, J = 8.8, 1.7 Hz, 1H), 8.84 (dd, J = 4.2, 1.7 Hz, 1H), 8.52 (d, J = 7.9 Hz, 1H), 8.16 (d, J = 8.6 Hz, 1H), 8.07 (d, J = 8.7 Hz, 1H), 7.98 (d, J = 8.2 Hz, 1H), 7.86 (d, J = 7.9 Hz, 1H), 7.71 – 7.56 (m, 2H), 7.37 (ddd, J = 8.3, 6.8, 1.3 Hz, 1H), 7.30 – 7.21 (m, 1H).

^{13}C NMR (101 MHz, CDCl$_3$): δ 191.4, 152.1, 147.8, 146.0, 143.2, 142.1, 136.2, 132.4, 132.2, 131.7, 130.6, 129.5, 129.0, 128.6, 127.3, 127.2, 124.4, 123.7, 122.7, 121.3.

HRMS (ESI+): m/z calcd for $C_{20}H_{13}N_2O_3$ $[M+H]^+$: 329.0926, found 329.0922.

1-(4-chloroquinolin-8-yl)-2-naphthaldehyde (**4d**)

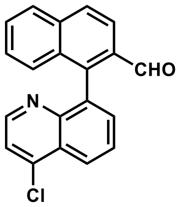

Yellow solid. 16% yield.

^{1}H NMR (400 MHz, CDCl$_3$): δ 9.65 (s, 1H), 8.63 (d, J = 4.6 Hz, 1H), 8.49 (dt, J = 5.9, 3.6 Hz, 1H), 8.16 (d, J = 8.6 Hz, 1H), 8.02 (d, J = 8.6 Hz, 1H), 7.95 (d, J = 8.2 Hz, 1H), 7.86 – 7.78 (m, 2H), 7.58 (ddd, J = 8.2, 6.0, 1.9 Hz, 1H), 7.52 (d, J = 4.6 Hz, 1H), 7.33 (d, J = 6.7 Hz, 2H).

^{13}C NMR (101 MHz, CDCl$_3$): δ 192.4, 150.5, 148.7, 144.0, 143.0, 136.3, 135.7, 133.7, 133.0, 132.0, 128.9, 128.8, 128.5, 127.6, 127.0, 126.9, 126.8, 125.5, 122.3, 121.8.

HRMS (ESI+): m/z calcd for $C_{20}H_{13}ClNO$ $[M+H]^+$: 318.0686, found 318.0689.

1-(5,6-difluoro-2-methylquinolin-8-yl)-2-naphthaldehyde (**4f**)

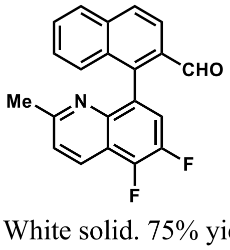

White solid. 75% yield.

^{1}H NMR (400 MHz, CDCl$_3$): δ 9.62 (s, 1H), 8.41 (d, J = 8.7 Hz, 1H), 8.14 (d, J = 8.6 Hz, 1H), 8.02 (d, J = 8.6 Hz, 1H), 7.96 (d, J = 8.2 Hz, 1H), 7.67 – 7.55 (m, 2H), 7.39 (dd, J = 13.8, 7.3 Hz, 3H), 2.44 (s, 3H).

^{13}C NMR (126 MHz, CDCl$_3$): δ 192.4, 160.1 (d, J = 2.6 Hz), 146.3 – 144.1 (m), 144.2 (dd, J = 256.9, 12.9 Hz), 142.2, 136.4, 133.0, 132.2, 131.5 – 131.3 (m), 128.9 (d, J = 6.3 Hz), 128.7, 128.5, 127.3, 127.0, 123.3 (d, J = 3.1 Hz), 122.6, 122.5, 122.3, 118.3 – 118.0 (m), 25.6.

^{19}F NMR (470 MHz, CDCl$_3$): δ -141.13 (dd, J = 20.0, 9.7 Hz), -146.81 (dd, J = 19.7, 8.3 Hz).
HRMS (ESI+): m/z calcd for $C_{21}H_{14}F_2NO$ [M+H]$^+$: 334.1043, found 334.1043.

1-(5-methoxyquinolin-8-yl)-2-naphthaldehyde (**4g**)

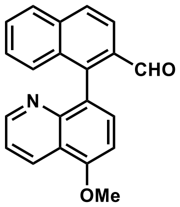

Yellow solid. 62% yield.
^{1}H NMR (400 MHz, CDCl$_3$): δ 9.71 (s, 1H), 8.77 (dd, J = 4.2, 1.8 Hz, 1H), 8.70 (dd, J = 8.5, 1.9 Hz, 1H), 8.14 (d, J = 8.6 Hz, 1H), 7.96 (dd, J = 21.2, 8.5 Hz, 2H), 7.63 (d, J = 7.9 Hz, 1H), 7.56 (ddd, J = 8.2, 6.7, 1.3 Hz, 1H), 7.45 (d, J = 8.6 Hz, 1H), 7.40 (dd, J = 8.4, 4.2 Hz, 1H), 7.33 (ddd, J = 8.4, 6.7, 1.3 Hz, 1H), 7.03 (d, J = 8.1 Hz, 1H), 4.13 (s, 3H).
^{13}C NMR (101 MHz, CDCl$_3$): δ 193.1, 156.0, 151.3, 148.5, 144.8, 136.5, 133.6, 132.8, 132.4, 131.1, 128.6, 128.6, 128.4, 127.9, 126.7, 126.6, 122.3, 120.7, 103.8, 56.1.
HRMS (ESI): m/z calcd for $C_{21}H_{16}NO_2$ [M+H]$^+$: 314.1181, found 314.1175.

2-(5,6-difluoro-2-methylquinolin-8-yl)-3-methylbenzaldehyde (**4k**)

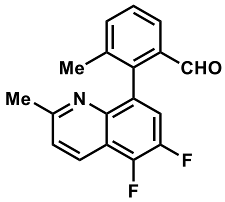

White solid. 83% yield.
^{1}H NMR (400 MHz, CDCl$_3$): δ 9.47 (s, 1H), 8.37 (dd, J = 8.6, 1.3 Hz, 1H), 7.91 (d, J = 7.7 Hz, 1H), 7.57 (d, J = 7.5 Hz, 1H), 7.51 – 7.39 (m, 2H), 7.37 (d, J = 8.6 Hz, 1H), 2.54 (s, 3H), 2.02 (s, 3H).
^{13}C NMR (126 MHz, CDCl$_3$): δ 192.3, 159.7, 145.3 (dd, J = 247.9, 11.5 Hz), 143.8 (dd, J = 256.1, 12.9 Hz), 143.3, 141.3, 138.1, 135.5, 135.3, 133.0 – 132.8 (m), 128.9 (dd, J = 6.4, 3.9 Hz), 128.2, 125.1, 123.0 (d, J = 3.1 Hz), 121.4, 121.3, 118.6 – 117.9 (m), 25.7, 20.2.
^{19}F NMR (470 MHz, CDCl$_3$): δ -141.14 (dd, J = 20.0, 10.7 Hz), -147.76 (dd, J = 19.5, 8.3 Hz).
HRMS (ESI+): m/z calcd for $C_{18}H_{14}F_2NO$ [M+H]$^+$: 298.1043, found 298.1040.

1-(quinoxalin-5-yl)-2-naphthaldehyde (**4m**)

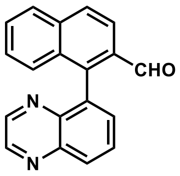

Yellow solid. 77% yield.
^{1}H NMR (400 MHz, CDCl$_3$): δ 9.66 (s, 1H), 8.89 (d, J = 1.8 Hz, 1H), 8.71 (d, J = 1.7 Hz, 1H), 8.34 (dd, J = 8.6, 1.5 Hz, 1H), 8.16 (d, J = 8.6 Hz, 1H), 8.05 (d, J = 8.6 Hz, 1H), 8.00 – 7.92 (m, 2H), 7.85 (dd, J = 7.1, 1.5 Hz, 1H), 7.60 (ddd, J = 8.1, 6.6, 1.4 Hz, 1H), 7.39 – 7.27 (m, 2H).

^{13}C NMR (101 MHz, CDCl$_3$): δ 192.1, 145.6, 145.5, 142.9, 142.9, 142.7, 136.3, 135.6, 133.1, 133.0, 132.2, 130.7, 129.5, 129.2, 128.9, 128.6, 127.5, 127.1, 122.4.
HRMS (ESI+): m/z calcd for $C_{19}H_{13}N_2O$ $[M+H]^+$: 285.1028, found 285.1023.

1-(benzofuran-7-yl)-2-naphthaldehyde (**4n**)

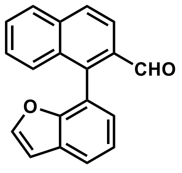

Yellow solid. 85% yield.
^{1}H NMR (400 MHz, CDCl$_3$): δ 9.81 (s, 1H), 8.14 (dd, J = 8.6, 1.5 Hz, 1H), 8.02 (d, J = 8.7 Hz, 1H), 7.97 (d, J = 8.2 Hz, 1H), 7.79 (dt, J = 7.7, 1.4 Hz, 1H), 7.66 – 7.60 (m, 1H), 7.59 – 7.52 (m, 2H), 7.48 – 7.39 (m, 2H), 7.34 (dt, J = 7.3, 1.4 Hz, 1H), 6.90 (t, J = 1.9 Hz, 1H).
^{13}C NMR (101 MHz, CDCl$_3$): δ 192.6, 153.8, 145.9, 141.0, 136.4, 132.4, 131.8, 129.2, 129.0, 128.5, 127.8, 127.5, 127.5, 127.2, 123.0, 122.4, 122.1, 119.1, 107.0.
HRMS (ESI+): m/z calcd for $C_{19}H_{13}O_2$ $[M+H]^+$: 273.0916, found 273.0910.

1-(benzo[b]thiophen-7-yl)-2-naphthaldehyde (**4o**)

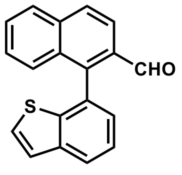

White solid. 81% yield.
^{1}H NMR (400 MHz, CDCl$_3$): δ 9.77 (s, 1H), 8.15 (dd, J = 8.7, 1.6 Hz, 1H), 8.07 – 7.85 (m, 3H), 7.68 – 7.49 (m, 3H), 7.47 (dd, J = 5.5, 1.6 Hz, 1H), 7.43 – 7.36 (m, 3H).
^{13}C NMR (101 MHz, CDCl$_3$): δ 192.3, 144.5, 142.1, 139.9, 136.5, 131.8, 131.2, 130.0, 129.3, 129.2, 128.5, 127.7, 127.2, 127.2, 127.1, 124.5, 124.3, 124.0, 122.3.
HRMS (ESI+): m/z calcd for $C_{19}H_{13}OS$ $[M+H]^+$: 289.0687, found 289.0681.

2-(5-methoxyquinolin-8-yl)-3-methylbenzaldehyde (**4p**)

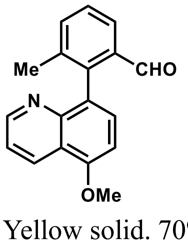

Yellow solid. 70% yield.
^{1}H NMR (400 MHz, CDCl$_3$): δ 9.52 (s, 1H), 8.86 (dd, J = 4.3, 1.8 Hz, 1H), 8.66 (dd, J = 8.6, 1.8 Hz, 1H), 7.92 (d, J = 9.2 Hz, 1H), 7.56 (d, J = 7.0 Hz, 1H), 7.45 (t, J = 7.6 Hz, 2H), 7.40 (dd, J = 8.4, 4.2 Hz, 1H), 6.96 (d, J = 7.9 Hz, 1H), 4.08 (s, 3H), 2.02 (s, 3H).
^{13}C NMR (101 MHz, CDCl$_3$): δ 193.2, 155.6, 151.2, 147.6, 143.6, 138.7, 135.5, 131.6, 131.1, 128.1, 127.9, 124.8, 120.9, 120.5, 103.9, 56.0, 20.1.
HRMS (ESI+): m/z calcd for $C_{18}H_{16}NO_2$ $[M+H]^+$: 278.1181, found 278.1180.

General procedure for the IRED catalyzed synthesis of biaryl amines

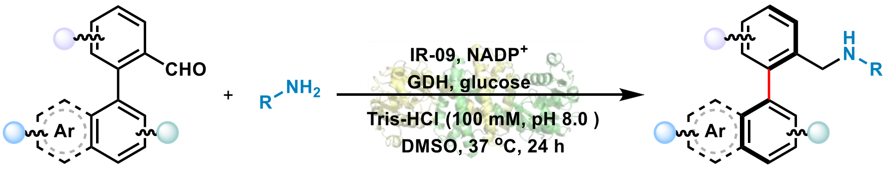

1a-1k, 4a-4p   2a-2j   3a-3k, 5a-5p, 6a-6i

General procedure 1

A screw vial (20 mL) was charged with GDH (400 µL, 25 mg/mL stock solution in 100 mM Tris-HCl buffer pH 8.0), glucose (500 µL, 60 mg/mL stock solution in 100 mM Tris-HCl buffer pH 8.0), $NADP^+$ (100 µL, 38 mg/mL stock solution in 100 mM Tris-HCl buffer pH 8.0), IR-09 protein (0.2 mol% catalyst loading for preparing **3a-k** and **5a-p** or 0.8 mol% catalyst loading for preparing **6a-i**), aldehyde (500 µL, 100 mM stock in DMSO, 0.05 mmol, 1 equiv.) and amine (8 equiv.). Tris-HCl buffer (100 mM, pH 8.0) was added to bring the total volume to 10 mL. The vial was sealed and placed on a shaker at 250 rpm at 37 °C for 24 h. Upon completion, transfer the reaction mixture to a 50 ml centrifuge tube, add 10 ml EtOAc, and centrifuge at 4000 rpm for 5 minutes (×4). Collected organic phases were dried over Na_2SO_4, filtering and solvent was removed under vacuum. The residue was further purified by column chromatography on silica gel to afford the axially chiral biaryl amines.

General procedure 2

A conical flask (1000 mL) was charged with GDH 200 mg, glucose 298 mg (3 equiv.), $NADP^+$ 76 mg (10 mol%), IR-09 lysate 80 ml (OD 40), aldehyde (10 ml, 100 mM stock in DMSO, 1 mmol, 1 equiv.) and amine (8 equiv.). Tris-HCl buffer (100 mM, pH 8.0) was added to bring the total volume to 200 mL. The conical flask was sealed and placed on a shaker at 250 rpm at 37 °C for 24 hours. Upon completion, transfer the reaction mixture to a 50 ml centrifuge tube, add EtOAc, and centrifuge at 4000 rpm for 5 minutes (×5). Collected organic phases were dried over Na_2SO_4, filtering and solvent was removed under vacuum. The residue was further purified by column chromatography on silica gel to afford the axially chiral biaryl amines **3d** and **6a**.

(*R*)-3,5-dimethyl-2-(2-((prop-2-yn-1-ylamino)methyl)naphthalen-1-yl)phenol (**3a**)

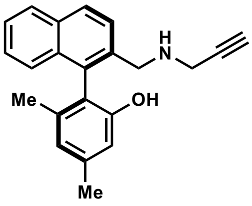

Yellow oil. 82% yield.

^{1}H NMR (400 MHz, $CDCl_3$): δ 7.87 (dd, J = 8.3, 2.9 Hz, 2H), 7.53 (d, J = 8.4 Hz, 1H), 7.49 – 7.41 (m, 1H), 7.34 (dt, J = 11.9, 7.9 Hz, 2H), 6.83 (s, 1H), 6.79 (s, 1H), 5.03 (brs, 1H), 4.01 (d, J = 11.6 Hz, 1H), 3.65 (d, J = 11.7 Hz, 1H), 3.44 (dd, J = 17.3, 2.4 Hz, 1H), 3.33 (dd, J = 17.3, 2.4 Hz, 1H), 2.39 (s, 3H), 2.28 (t, J = 1.6 Hz, 1H), 1.72 (s, 3H).

^{13}C NMR (101 MHz, $CDCl_3$): δ 155.0, 138.9, 138.1, 135.1, 134.6, 133.6, 133.2, 128.3, 128.1, 127.3, 126.7, 126.2, 126.0, 124.5, 123.6, 117.7, 80.7, 72.9, 51.2, 37.1, 21.4, 20.2.

HRMS (ESI+): m/z calcd for $C_{22}H_{22}NO$ $[M+H]^+$: 316.1701, found 316.1699.

Enantioselectivity: 96% *ee*.

Chiral HPLC method: ADH column, 254 nm, 20% isopropanol/hexanes, flow rate 0.8 mL/min, room temperature, t_R (major) = 7.02 min, t_R (minor) = 13.05 min.

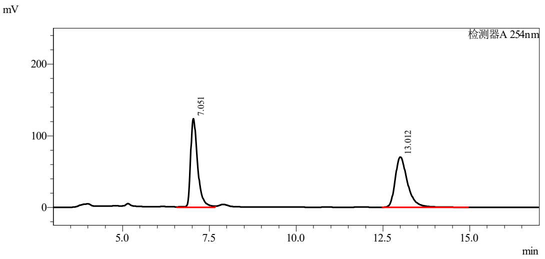

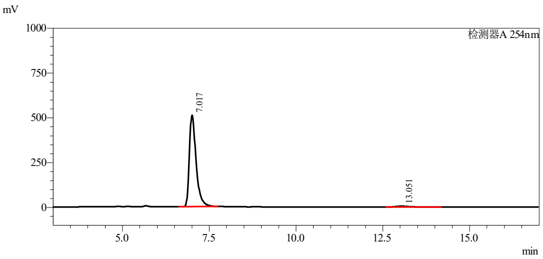

〈峰表〉

检测器A 254nm

峰号	保留时间	面积	高度	浓度
1	7.051	1750240	123697	50.197
2	13.012	1736512	70001	49.803
总计		3486753	193698	

〈峰表〉

检测器A 254nm

峰号	保留时间	面积	高度	浓度
1	7.017	6978392	510710	98.290
2	13.051	121417	4767	1.710
总计		7099810	515477	

English alongside: The HPLC spectra depicted in the SI are as follows.

< Peak table >

Detector A 254nm

Peak number	Retention time	Area	Height	Concentration
1	7.051	1750240	123697	50.197
2	13.012	1736512	70001	49.803
Total		3486753	193698	

Peak number	Retention time	Area	Height	Concentration
1	7.017	6978392	510710	98.290
2	13.051	121417	4767	1.710
Total		7099810	515477	

(*S*)-3-methoxy-6-methyl-2-(2-((prop-2-yn-1-ylamino)methyl)naphthalen-1-yl)phenol (**3b**)

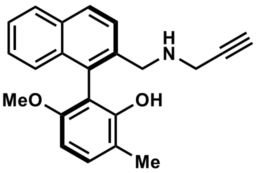

Yellow solid. 63% yield.

^{1}H NMR (400 MHz, CDCl$_3$): δ 7.87 (t, J = 9.0 Hz, 2H), 7.55 (d, J = 8.4 Hz, 1H), 7.44 (dt, J = 8.2, 4.0 Hz, 1H), 7.34 (d, J = 3.7 Hz, 2H), 7.20 (d, J = 8.3 Hz, 1H), 6.57 (d, J = 8.3 Hz, 1H), 4.00 (d, J = 11.6 Hz, 1H), 3.71 (d, J = 11.6 Hz, 1H), 3.54 (s, 3H), 3.44 (dd, J = 17.4, 3.0 Hz, 1H), 3.32 (dd, J = 17.1, 3.3 Hz, 1H), 2.29 (s, 3H), 2.24 (t, J = 2.9 Hz, 1H).

^{13}C NMR (101 MHz, CDCl$_3$): δ 156.5, 154.0, 135.0, 133.4, 132.5, 130.3, 128.5, 128.0, 127.5, 126.4, 126.4, 125.9, 120.6, 115.8, 103.1, 80.6, 72.7, 55.9, 51.4, 37.1, 16.3.

HRMS (ESI+): m/z calcd for $C_{22}H_{22}NO_2$ $[M+H]^+$: 332.1651, found 332.1652.

Enantioselectivity: >99% *ee*.

Chiral HPLC method: ADH column, 254 nm, 10% isopropanol/hexanes, flow rate 0.8 mL/min, room temperature, t_R (major) = 20.98 min, t_R (minor) = 27.82 min.

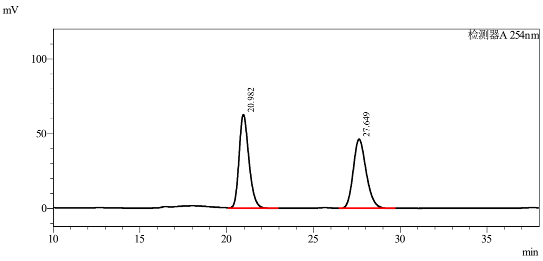

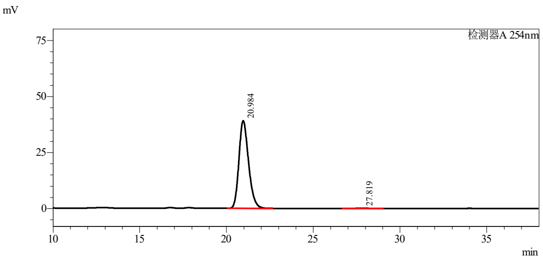

〈峰表〉
检测器A 254nm

峰号	保留时间	面积	高度	浓度
1	20.982	2468284	62668	50.718
2	27.649	2398436	46216	49.282
总计		4866720	108884	

〈峰表〉
检测器A 254nm

峰号	保留时间	面积	高度	浓度
1	20.984	1511555	39053	99.700
2	27.819	4553	83	0.300
总计		1516107	39137	

(*R*)-6-isopropyl-3-methyl-2-(2-((prop-2-yn-1-ylamino)methyl)naphthalen-1-yl)phenol (**3c**)

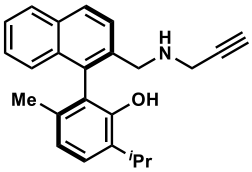

Colorless oil. 21% yield.

^{1}H NMR (400 MHz, CDCl$_3$): δ 7.87 (dd, J = 8.3, 5.7 Hz, 2H), 7.54 (d, J = 8.4 Hz, 1H), 7.45 (t, J = 7.4 Hz, 1H), 7.35 (t, J = 7.6 Hz, 1H), 7.31 – 7.18 (m, 2H), 6.92 (d, J = 7.8 Hz, 1H), 4.00 (d, J = 11.6 Hz, 1H), 3.61 (d, J = 11.6 Hz, 1H), 3.50 – 3.34 (m, 2H), 3.30 (dd, J = 17.2, 2.4 Hz, 1H), 2.26 (t, J = 2.4 Hz, 1H), 1.71 (s, 3H), 1.28 (t, J = 6.4 Hz, 6H).

^{13}C NMR (101 MHz, CDCl$_3$): δ 152.2, 136.0, 135.6, 135.3, 134.6, 133.6, 133.1, 128.3, 128.1, 127.4, 127.3, 126.8, 126.1, 126.1, 125.5, 122.3, 80.7, 72.8, 51.4, 37.3, 27.4, 23.4, 22.7, 20.0.

HRMS (ESI+): m/z calcd for $C_{24}H_{26}NO$ $[M+H]^+$: 334.2014, found 334.2018.

Enantioselectivity: >99% *ee*.

Chiral HPLC method: ADH column, 254 nm, 20% isopropanol/hexanes, flow rate 0.8 mL/min, room temperature, t_R (major) = 6.21 min, t_R (minor) = 14.55 min.

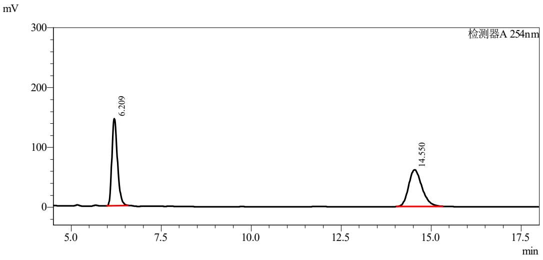

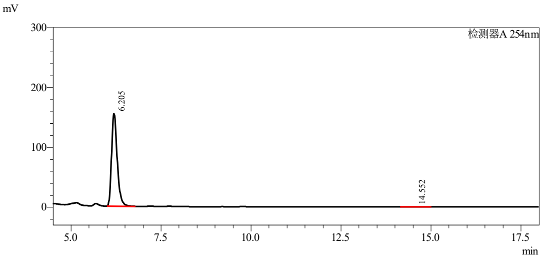

〈峰表〉
检测器A 254nm

峰号	保留时间	面积	高度	浓度
1	6.209	1567909	145371	49.685
2	14.550	1587766	61409	50.315
总计		3155674	206780	

〈峰表〉
检测器A 254nm

峰号	保留时间	面积	高度	浓度
1	6.205	1691908	154341	99.773
2	14.552	3847	168	0.227
总计		1695755	154508	

(*R*)-1-(2-methyl-6-((prop-2-yn-1-ylamino)methyl)phenyl)naphthalen-2-ol (**3d**)

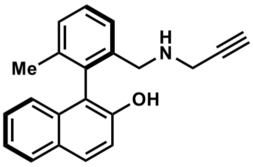

Colorless oil. 89% yield.

^{1}H NMR (400 MHz, CDCl$_3$): δ 7.85 – 7.77 (m, 2H), 7.40 – 7.23 (m, 6H), 7.00 (d, *J* = 8.2 Hz, 1H), 5.21 (brs, 1H), 3.83 (d, *J* = 11.6 Hz, 1H), 3.47 – 3.35 (m, 2H), 3.25 (dd, *J* = 17.2, 2.4 Hz, 1H), 2.21 (t, *J* = 2.4 Hz, 1H), 1.80 (s, 3H).

^{13}C NMR (101 MHz, CDCl$_3$): δ 152.3, 139.6, 138.0, 136.2, 133.5, 130.5, 129.5, 129.3, 128.2, 128.1, 127.4, 126.4, 124.4, 123.5, 122.5, 121.8, 80.3, 72.9, 51.6, 36.8, 20.4.

HRMS (ESI+): m/z calcd for $C_{21}H_{20}NO$ [M+H]$^+$: 302.1545, found 302.1541.

Enantioselectivity: 98% *ee*.

Chiral HPLC method: ASH column, 254 nm, 2% isopropanol/hexanes, flow rate 0.8 mL/min, room temperature, t_R (major) = 19.00 min, t_R (minor) = 15.90 min.

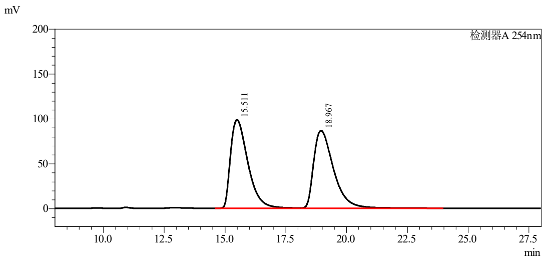

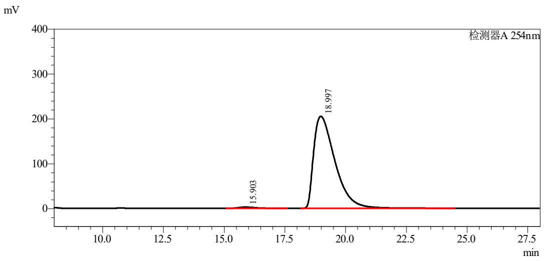

〈峰表〉
检测器A 254nm

峰号	保留时间	面积	高度	浓度
1	15. 511	4925095	98558	49. 919
2	18. 967	4941066	86637	50. 081
总计		9866161	185194	

〈峰表〉
检测器A 254nm

峰号	保留时间	面积	高度	浓度
1	15. 903	134672	2699	1. 062
2	18. 997	12543351	205138	98. 938
总计		12678023	207837	

(*R*)-1-(2-ethyl-6-((prop-2-yn-1-ylamino)methyl)phenyl)naphthalen-2-ol (**3e**)

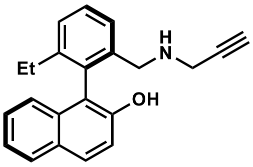

Colorless oil. 46% yield.

^{1}H NMR (400 MHz, CDCl$_3$): δ 7.84 – 7.77 (m, 2H), 7.44 – 7.35 (m, 2H), 7.34 – 7.28 (m, 3H), 7.28 – 7.22 (m, 2H), 7.01 – 6.93 (m, 1H), 3.82 (d, *J* = 11.7 Hz, 1H), 3.43 – 3.31 (m, 2H), 3.24 (dd, *J* = 17.2, 2.4 Hz, 1H), 2.20 (t, *J* = 2.4 Hz, 1H), 2.07 (ddt, *J* = 20.6, 14.5, 7.3 Hz, 2H), 0.91 (t, *J* = 7.6 Hz, 3H).

^{13}C NMR (101 MHz, CDCl$_3$): δ 152.7, 145.5, 138.1, 135.5, 133.8, 129.5, 129.3, 128.7, 128.3, 128.1, 127.3, 126.3, 124.6, 123.5, 122.3, 121.7, 80.4, 72.9, 51.7, 36.9, 26.9, 14.9.

HRMS (ESI+): m/z calcd for $C_{22}H_{22}NO$ [M+H]$^+$: 316.1701, found 316.1701.

Enantioselectivity: 95% *ee*.

Chiral HPLC method: ADH column, 254 nm, 10% isopropanol/hexanes, flow rate 0.8 mL/min, room temperature, t_R (major) = 7.19 min, t_R (minor) = 8.92 min.

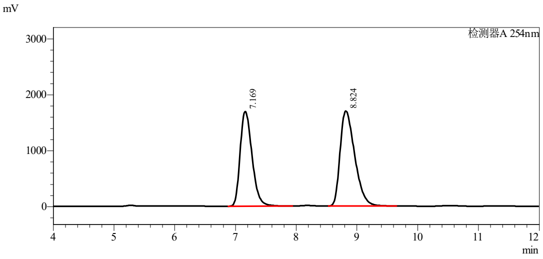

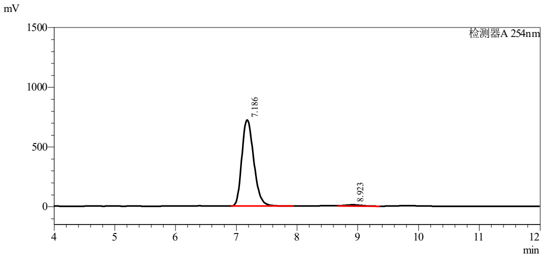

〈峰表〉
检测器A 254nm

峰号	保留时间	面积	高度	浓度
1	7.169	23113256	1691105	45.163
2	8.824	28064466	1689600	54.837
总计		51177723	3380704	

〈峰表〉
检测器A 254nm

峰号	保留时间	面积	高度	浓度
1	7.186	9853493	722654	97.899
2	8.923	211474	11235	2.101
总计		10064967	733888	

(*R*)-2'-((prop-2-yn-1-ylamino)methyl)-[1,1'-binaphthalen]-2-ol (**3f**)

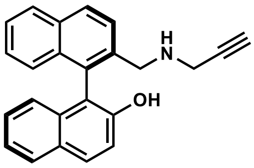

Yellow solid. 33% yield.

^{1}H NMR (400 MHz, CDCl$_3$): δ 7.98 (d, J = 8.3 Hz, 1H), 7.94 – 7.83 (m, 3H), 7.61 (d, J = 8.4 Hz, 1H), 7.50 – 7.37 (m, 2H), 7.33 – 7.27 (m, 1H), 7.17 (dtd, J = 24.3, 7.5, 1.4 Hz, 2H), 7.03 (d, J = 8.6 Hz, 1H), 6.80 (d, J = 8.5 Hz, 1H), 4.98 (brs, 1H), 4.01 (d, J = 11.6 Hz, 1H), 3.62 (d, J = 11.6 Hz, 1H), 3.44 (dd, J = 17.2, 2.4 Hz, 1H), 3.30 (dd, J = 17.2, 2.4 Hz, 1H), 2.23 (t, J = 2.4 Hz, 1H).

^{13}C NMR (101 MHz, CDCl$_3$): δ 153.4, 135.7, 134.4, 133.8, 133.8, 133.6, 129.8, 129.4, 128.9, 128.1, 128.0, 127.4, 127.0, 126.7, 126.4, 126.1, 125.4, 123.5, 121.6, 120.7, 80.4, 73.0, 51.6, 37.1.

HRMS (ESI+): m/z calcd for $C_{24}H_{20}NO$ [M+H]$^+$: 338.1545, found 338.1540.

Enantioselectivity: 96% *ee*.

Chiral HPLC method: ADH column, 254 nm, 10% isopropanol/hexanes, flow rate 0.8 mL/min, room temperature, t_R (major) = 18.11 min, t_R (minor) = 24.64 min.

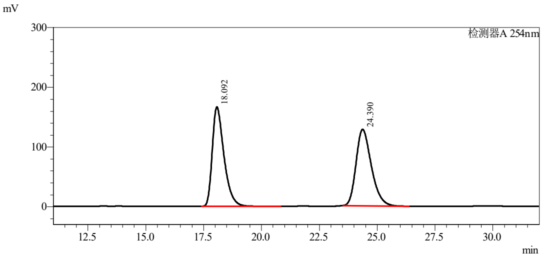

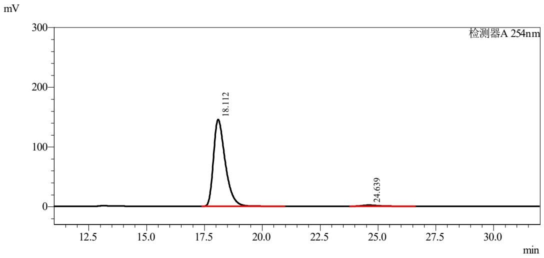

〈峰表〉
检测器A 254nm

峰号	保留时间	面积	高度	浓度
1	18.092	5867544	166401	49.849
2	24.390	5903166	128015	50.151
总计		11770709	294416	

〈峰表〉
检测器A 254nm

峰号	保留时间	面积	高度	浓度
1	18.112	5147276	145399	98.128
2	24.639	98219	1909	1.872
总计		5245495	147308	

(R)-2'-methyl-6'-((prop-2-yn-1-ylamino)methyl)-[1,1'-biphenyl]-2-ol (**3g**)

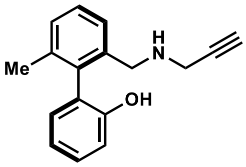

Colorless oil. 56% yield.

^{1}H NMR (400 MHz, CDCl$_3$): δ 7.30 – 7.22 (m, 3H), 7.19 (dd, J = 6.9, 2.1 Hz, 1H), 7.08 (d, J = 8.1 Hz, 1H), 6.97 (d, J = 4.5 Hz, 2H), 3.86 (d, J = 11.5 Hz, 1H), 3.50 (d, J = 11.5 Hz, 1H), 3.43 (dd, J = 17.2, 2.5 Hz, 1H), 3.29 (dd, J = 17.3, 2.4 Hz, 1H), 2.27 (t, J = 2.6 Hz, 1H), 2.02 (s, 3H).

^{13}C NMR (101 MHz, CDCl$_3$): δ 154.7, 138.9, 138.5, 136.6, 131.1, 130.5, 129.6, 129.1, 127.7, 127.1, 120.9, 120.4, 80.3, 73.1, 51.3, 36.7, 21.2.

HRMS (ESI+): m/z calcd for $C_{17}H_{18}NO$ [M+H]$^+$: 252.1388, found 252.1384.

Enantioselectivity: 69% *ee*.

Chiral HPLC method: ODH column, 254 nm, 10% isopropanol/hexanes, flow rate 0.8 mL/min, room temperature, t_R (major) = 7.25 min, t_R (minor) = 9.06 min.

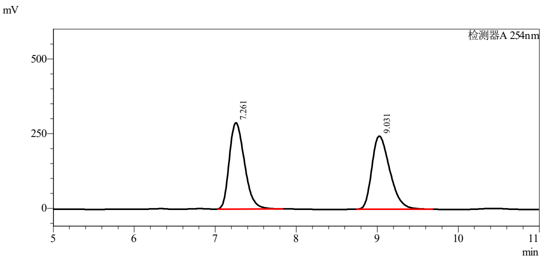

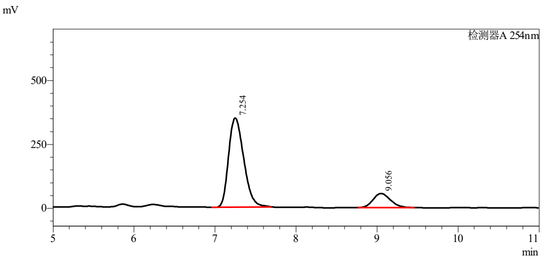

〈峰表〉
检测器A 254nm

峰号	保留时间	面积	高度	浓度
1	7.261	3861659	290131	50.220
2	9.031	3827893	245270	49.780
总计		7689552	535401	

〈峰表〉
检测器A 254nm

峰号	保留时间	面积	高度	浓度
1	7.254	4540456	348278	84.653
2	9.056	823167	54912	15.347
总计		5363624	403190	

The product **3g** was assigned the absolute configuration as R configuration through comparison with the reported data.[4]

Enantioselectivity: 99:1 er. Chiral HPLC method: OD-H column, 210 nm, 10% isopropanol/hexanes, flow rate 1.0 mL/min, room temperature, t_R (minor) = 6.13 min, t_R (major) = 7.45 min.

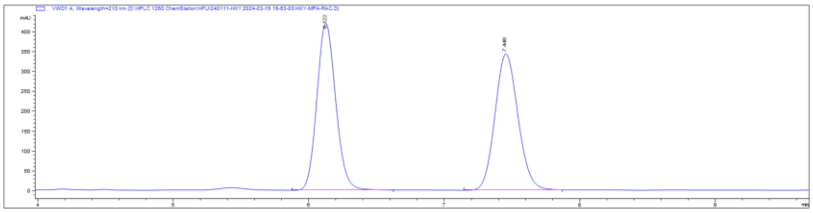

#	Time	Type	Area	Height	Width	Area%	Symmetry
1	6.122	BB	4045.4	419.5	0.1485	49.921	0.857
2	7.448	MF	4058.3	342.2	0.1976	50.079	0.876

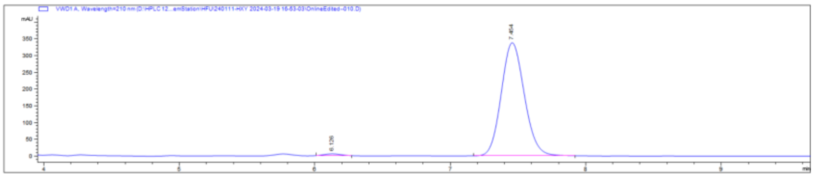

#	Time	Type	Area	Height	Width	Area%	Symmetry
1	6.126	MF	58.2	6.3	0.1545	1.424	0.898
2	7.454	MF	4030.5	338.8	0.1983	98.576	0.872

(*S*)-6-bromo-2'-methyl-6'-((prop-2-yn-1-ylamino)methyl)-[1,1'-biphenyl]-2-ol (**3h**)

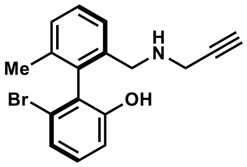

Yellow solid. 52% yield.

^{1}H NMR (400 MHz, CDCl$_3$): δ 7.34 – 7.25 (m, 3H), 7.22 (dd, J = 6.5, 2.0 Hz, 1H), 7.17 – 7.10 (m, 1H), 7.04 (dt, J = 8.1, 1.5 Hz, 1H), 5.35 (brs, 1H), 3.86 (dd, J = 11.7, 1.6 Hz, 1H), 3.47 – 3.34 (m, 2H), 3.26 (dt, J = 17.2, 2.1 Hz, 1H), 2.26 (t, J = 3.3 Hz, 1H), 2.00 (s, 3H).

^{13}C NMR (101 MHz, CDCl$_3$): δ 156.0, 138.8, 137.8, 136.5, 130.9, 130.5, 129.9, 128.4, 127.1, 125.4, 124.6, 119.5, 80.2, 73.1, 51.4, 36.8, 20.2.

HRMS (ESI+): m/z calcd for $C_{17}H_{17}BrNO$ [M+H]$^+$: 330.0494, found 330.0497.

Enantioselectivity: 98% *ee*. Absolute configuration of the enzymatic product **3h** was assigned as *S* by X-ray crystallography.

Chiral HPLC method: ADH column, 254 nm, 10% isopropanol/hexanes, flow rate 0.8 mL/min, room temperature, t_R (major) = 11.20 min, t_R (minor) = 9.10 min.

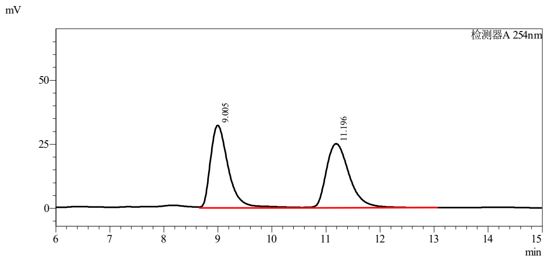

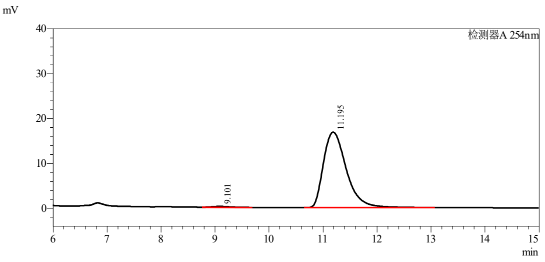

〈峰表〉
检测器A 254nm

峰号	保留时间	面积	高度	浓度
1	9.005	741927	32123	50.053
2	11.196	740358	24968	49.947
总计		1482285	57091	

〈峰表〉
检测器A 254nm

峰号	保留时间	面积	高度	浓度
1	9.101	4312	191	0.858
2	11.195	498375	16831	99.142
总计		502687	17022	

(*S*)-6-bromo-3-methoxy-2'-methyl-6'-((prop-2-yn-1-ylamino)methyl)-[1,1'-biphenyl]-2-ol (**3i**)

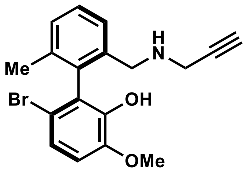

Red solid. 68% yield.

^{1}H NMR (400 MHz, CDCl$_3$): δ 7.34 – 7.23 (m, 4H), 7.21 (d, J = 8.7 Hz, 1H), 6.79 (d, J = 8.7 Hz, 1H), 4.98 (brs, 1H), 3.90 (s, 3H), 3.80 (d, J = 12.1 Hz, 1H), 3.50 (d, J = 12.1 Hz, 1H), 3.37 (dd, J = 17.2, 2.4 Hz, 1H), 3.29 (dd, J = 17.2, 2.4 Hz, 1H), 2.20 (t, J = 2.4 Hz, 1H), 2.01 (s, 3H).

^{13}C NMR (101 MHz, CDCl$_3$): δ 149.5, 144.9, 138.3, 137.4, 136.4, 130.1, 130.0, 128.3, 127.2, 124.2, 115.2, 111.9, 80.6, 72.6, 56.2, 50.8, 36.6, 20.2.

HRMS (ESI+): m/z calcd for $C_{18}H_{19}BrNO_2$ [M+H]$^+$: 360.0599, found 360.0600.

Enantioselectivity: 99% *ee*.

Chiral HPLC method: ODH column, 254 nm, 10% isopropanol/hexanes, flow rate 0.8 mL/min, room temperature, t_R (major) = 19.06 min, t_R (minor) = 22.97 min.

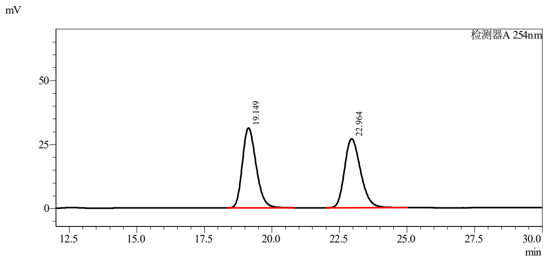

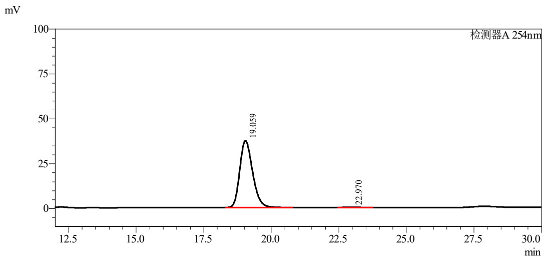

〈峰表〉
检测器A 254nm

峰号	保留时间	面积	高度	浓度
1	19.149	1107888	31202	49.837
2	22.964	1115147	26929	50.163
总计		2223034	58131	

〈峰表〉
检测器A 254nm

峰号	保留时间	面积	高度	浓度
1	19.059	1238815	37279	99.431
2	22.970	7094	208	0.569
总计		1245909	37487	

(*R*)-2',4,6-trimethyl-6'-((prop-2-yn-1-ylamino)methyl)-[1,1'-biphenyl]-2-ol (**3j**)

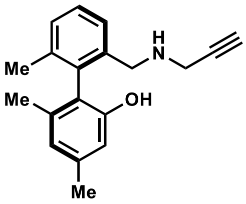

Yellow oil. 22% yield.

^{1}H NMR (400 MHz, CDCl$_3$): δ 7.28 – 7.17 (m, 3H), 6.75 (s, 1H), 6.71 (s, 1H), 3.80 (d, J = 11.6 Hz, 1H), 3.44 – 3.33 (m, 2H), 3.25 (dd, J = 17.3, 2.4 Hz, 1H), 2.32 (s, 3H), 2.24 (t, J = 2.4 Hz, 1H), 1.94 (s, 3H), 1.81 (s, 3H).

^{13}C NMR (101 MHz, CDCl$_3$): δ 154.0, 138.6, 138.4, 137.5, 137.2, 136.9, 130.3, 127.6, 127.1, 126.3, 123.8, 117.9, 80.7, 72.7, 51.2, 36.8, 21.3, 20.2, 19.9.

HRMS (ESI+): m/z calcd for $C_{19}H_{22}NO$ [M+H]$^+$: 280.1701, found 280.1693.

Enantioselectivity: 78% *ee*.

Chiral HPLC method: ODH column, 254 nm, 5% isopropanol/hexanes, flow rate 0.8 mL/min, room temperature, t_R (major) = 6.08 min, t_R (minor) = 7.95 min.

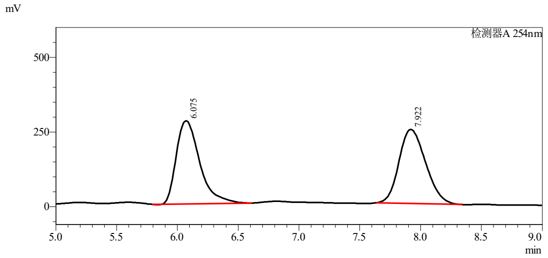

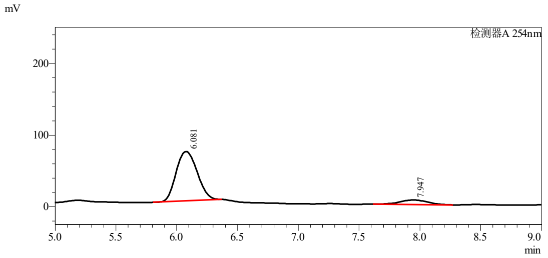

〈峰表〉
检测器A 254nm

峰号	保留时间	面积	高度	浓度
1	6.075	3742532	278396	49.684
2	7.922	3790075	248099	50.316
总计		7532607	526494	

〈峰表〉
检测器A 254nm

峰号	保留时间	面积	高度	浓度
1	6.081	831879	68924	89.120
2	7.947	101556	6378	10.880
总计		933435	75302	

(*R*)-4-chloro-3,5-dimethyl-2-(2-((prop-2-yn-1-ylamino)methyl)naphthalen-1-yl)phenol (**3k**)

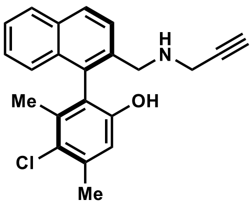

Yellow solid. 6% yield.

^{1}H NMR (400 MHz, CDCl$_3$): δ 7.88 (t, J = 7.4 Hz, 2H), 7.52 (d, J = 8.4 Hz, 1H), 7.46 (t, J = 7.4 Hz, 1H), 7.40 – 7.32 (m, 1H), 7.29 – 7.20 (m, 1H), 6.93 (s, 1H), 4.01 (d, J = 11.6 Hz, 1H), 3.63 (d, J = 11.6 Hz, 1H), 3.44 (dd, J = 17.3, 2.5 Hz, 1H), 3.31 (dd, J = 17.3, 2.5 Hz, 1H), 2.45 (s, 3H), 2.29 (t, J = 2.5 Hz, 1H), 1.80 (s, 3H).

^{13}C NMR (101 MHz, CDCl$_3$): δ 153.3, 137.1, 136.1, 134.9, 134.4, 133.6, 133.1, 128.7, 128.2, 127.3, 127.2, 127.0, 126.8, 126.2, 126.1, 119.8, 80.4, 73.1, 51.3, 37.1, 21.1, 18.5.

HRMS (ESI+): m/z calcd for $C_{22}H_{21}ClNO$ [M+H]$^+$: 350.1312, found 350.1311.

Enantioselectivity: 83% *ee*.

Chiral HPLC method: ADH column, 254 nm, 20% isopropanol/hexanes, flow rate 0.8 mL/min, room temperature, t_R (major) = 6.94 min, t_R (minor) = 11.88 min.

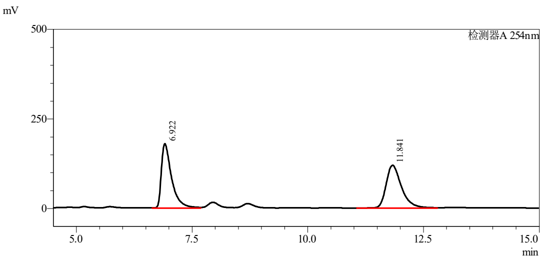

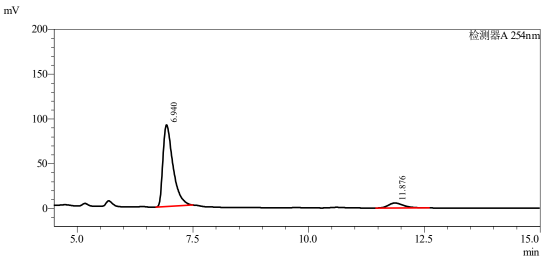

〈峰表〉
检测器A 254nm

峰号	保留时间	面积	高度	浓度
1	6.922	2800838	180283	50.145
2	11.841	2784626	119618	49.855
总计		5585464	299901	

〈峰表〉
检测器A 254nm

峰号	保留时间	面积	高度	浓度
1	6.940	1385283	91364	91.526
2	11.876	128250	5583	8.474
总计		1513533	96946	

(*S*)-N-((1-(quinolin-8-yl)naphthalen-2-yl)methyl)prop-2-yn-1-amine (**5a**)

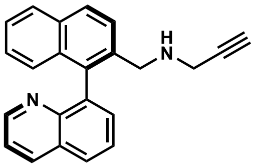

Yellow oil. 91% yield.

^{1}H NMR (400 MHz, CDCl$_3$): δ 8.79 (dt, J = 3.0, 1.5 Hz, 1H), 8.26 (dd, J = 8.2, 1.8 Hz, 1H), 8.00 – 7.93 (m, 2H), 7.90 (d, J = 8.2 Hz, 1H), 7.76 (d, J = 8.4 Hz, 1H), 7.72 – 7.61 (m, 2H), 7.41 (td, J = 8.2, 2.7 Hz, 2H), 7.28 – 7.20 (m, 1H), 7.17 (d, J = 8.5 Hz, 1H), 3.64 (d, J = 12.2 Hz, 1H), 3.56 (d, J = 12.2 Hz, 1H), 3.23 (dd, J = 17.0, 2.4 Hz, 1H), 3.13 (dd, J = 17.0, 2.4 Hz, 1H), 1.75 (t, J = 1.9 Hz, 1H).

^{13}C NMR (101 MHz, CDCl$_3$): δ 150.8, 147.7, 138.3, 136.6, 136.5, 135.2, 133.4, 133.0, 132.0, 128.8, 128.3, 128.1, 128.1, 127.6, 126.6, 126.3, 126.0, 125.5, 121.4, 81.4, 70.9, 51.1, 37.7.

HRMS (ESI+): m/z calcd for $C_{23}H_{19}N_2$ [M+H]$^+$: 323.1548, found 323.1540.

Enantioselectivity: >99% *ee*.

Chiral HPLC method: ADH column, 254 nm, 10% isopropanol/hexanes, flow rate 0.8 mL/min, room temperature, t_R (major) = 17.24 min, t_R (minor) = 19.54 min.

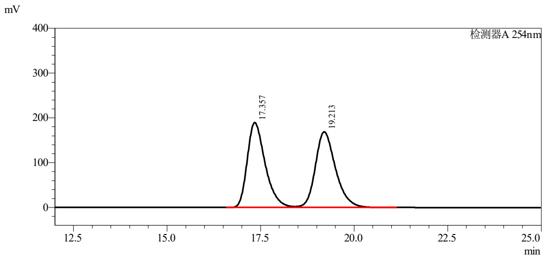

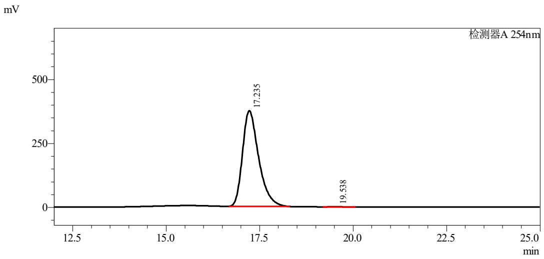

〈峰表〉
检测器A 254nm

峰号	保留时间	面积	高度	浓度
1	17.357	6340827	189582	49.913
2	19.213	6362961	168869	50.087
总计		12703788	358451	

〈峰表〉
检测器A 254nm

峰号	保留时间	面积	高度	浓度
1	17.235	11264484	373689	99.861
2	19.538	15645	663	0.139
总计		11280130	374353	

(*S*)-N-((1-(5-nitroquinolin-8-yl)naphthalen-2-yl)methyl)prop-2-yn-1-amine (**5b**)

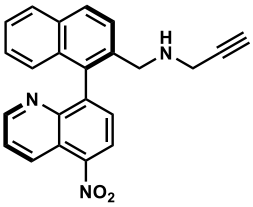

Yellow oil. 97% yield.

^{1}H NMR (400 MHz, CDCl$_3$): δ 9.08 (dt, J = 8.9, 1.6 Hz, 1H), 8.87 (dd, J = 3.7, 1.9 Hz, 1H), 8.49 (dd, J = 7.9, 1.4 Hz, 1H), 8.00 (d, J = 8.4 Hz, 1H), 7.92 (d, J = 8.2 Hz, 1H), 7.77 (dt, J = 8.4, 2.5 Hz, 2H), 7.64 (ddd, J = 8.9, 4.1, 1.3 Hz, 1H), 7.49 – 7.40 (m, 1H), 7.31 – 7.19 (m, 1H), 7.04 (d, J = 8.5 Hz, 1H), 3.61 (d, J = 12.2 Hz, 1H), 3.52 (d, J = 12.3 Hz, 1H), 3.24 (dt, J = 17.2, 1.8 Hz, 1H), 3.14 (dt, J = 17.1, 1.9 Hz, 1H), 1.73 (t, J = 1.6 Hz, 1H).

^{13}C NMR (101 MHz, CDCl$_3$): δ 151.8, 147.6, 146.3, 145.5, 135.2, 135.0, 132.9, 132.7, 132.2, 130.3, 129.0, 128.3, 127.5, 126.4, 126.1, 125.8, 124.1, 124.1, 121.6, 81.6, 70.9, 50.7, 37.6.

HRMS (ESI+): m/z calcd for $C_{23}H_{18}N_3O_2$ $[M+H]^+$: 368.1399, found 368.1400.

Enantioselectivity: >99% *ee*.

Chiral HPLC method: IA column, 254 nm, 10% isopropanol/hexanes, flow rate 0.8 mL/min, room temperature, t_R (major) = 24.88 min, t_R (minor) = 26.93 min.

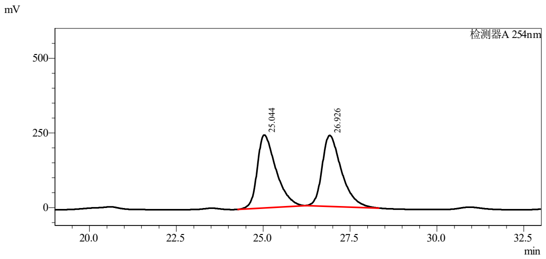

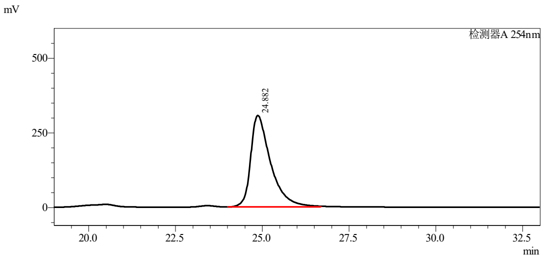

〈峰表〉
检测器A 254nm

峰号	保留时间	面积	高度	浓度
1	25.044	9206395	243932	49.938
2	26.926	9229376	237549	50.062
总计		18435771	481481	

〈峰表〉
检测器A 254nm

峰号	保留时间	面积	高度	浓度
1	24.882	12438927	306539	100.000
总计		12438927	306539	

(*S*)-N-((1-(5-fluoroquinolin-8-yl)naphthalen-2-yl)methyl)prop-2-yn-1-amine (**5c**)

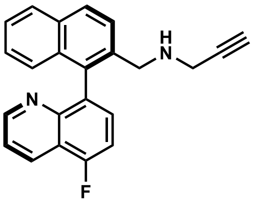

Colorless oil. >99% yield.

^{1}H NMR (400 MHz, CDCl$_3$): δ 8.82 (dd, J = 4.3, 1.8 Hz, 1H), 8.54 (dd, J = 8.5, 1.9 Hz, 1H), 7.96 (d, J = 8.5 Hz, 1H), 7.90 (d, J = 8.2 Hz, 1H), 7.75 (d, J = 8.5 Hz, 1H), 7.59 (dd, J = 8.0, 6.1 Hz, 1H), 7.50 – 7.32 (m, 3H), 7.29 – 7.20 (m, 2H), 7.15 (d, J = 8.5 Hz, 1H), 3.64 (d, J = 12.1 Hz, 1H), 3.55 (d, J = 12.2 Hz, 1H), 3.24 (dd, J = 17.1, 2.4 Hz, 1H), 3.14 (dd, J = 17.0, 2.4 Hz, 1H), 1.76 (t, J = 2.4 Hz, 1H).

^{13}C NMR (126 MHz, CDCl$_3$): δ 157.7 (d, J = 255.6 Hz), 151.5, 148.1 (d, J = 2.9 Hz), 135.8 (d, J = 2.5 Hz), 134.3 (d, J = 4.7 Hz), 133.5, 133.0, 131.3 (d, J = 8.6 Hz), 129.5 (d, J = 4.9 Hz), 128.4, 128.2, 127.6, 126.5, 126.0, 125.5, 121.5 (d, J = 2.8 Hz), 119.4 (d, J = 16.1 Hz), 110.0, 109.9, 81.8, 70.7, 51.0, 37.7.

^{19}F NMR (470 MHz, CDCl$_3$): δ -122.92.

HRMS (ESI+): m/z calcd for $C_{23}H_{18}FN_2$ $[M+H]^+$: 341.1454, found 341.1451.

Enantioselectivity: >99% *ee*.

Chiral HPLC method: IA column, 254 nm, 5% isopropanol/hexanes, flow rate 0.8 mL/min, room temperature, t_R (major) = 20.80 min, t_R (minor) = 22.96 min.

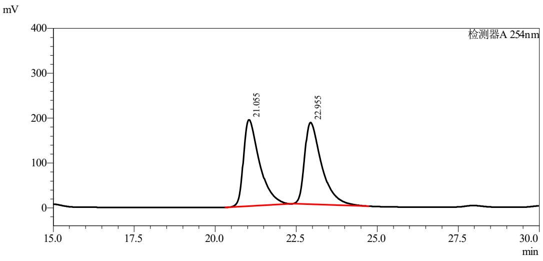

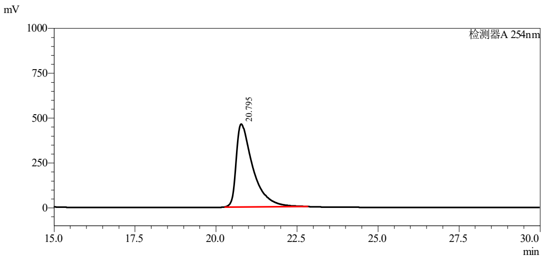

〈峰表〉
检测器A 254nm

峰号	保留时间	面积	高度	浓度
1	21.055	6703084	192479	49.971
2	22.955	6710741	182116	50.029
总计		13413824	374595	

〈峰表〉
检测器A 254nm

峰号	保留时间	面积	高度	浓度
1	20.795	16597329	461754	100.000
总计		16597329	461754	

(*S*)-N-((1-(4-chloroquinolin-8-yl)naphthalen-2-yl)methyl)prop-2-yn-1-amine (**5d**)

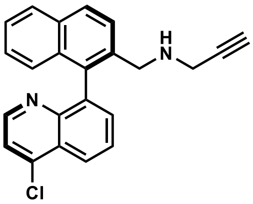

Yellow oil. 87% yield.

^{1}H NMR (400 MHz, CDCl$_3$): δ 8.63 (d, J = 4.6 Hz, 1H), 8.41 (dd, J = 8.4, 1.5 Hz, 1H), 7.96 (d, J = 8.5 Hz, 1H), 7.90 (d, J = 8.2 Hz, 1H), 7.83 – 7.68 (m, 3H), 7.50 (d, J = 4.6 Hz, 1H), 7.42 (ddd, J = 8.1, 6.8, 1.2 Hz, 1H), 7.27 – 7.21 (m, 1H), 7.11 (d, J = 8.5 Hz, 1H), 3.62 (d, J = 12.2 Hz, 1H),

3.54 (d, J = 12.2 Hz, 1H), 3.23 (dd, J = 17.0, 2.4 Hz, 1H), 3.13 (dd, J = 17.0, 2.4 Hz, 1H), 1.74 (t, J = 2.4 Hz, 1H).

^{13}C NMR (101 MHz, CDCl$_3$): δ 150.1, 148.6, 142.9, 138.9, 136.1, 135.5, 133.3, 133.0, 132.9, 128.4, 128.2, 127.5, 127.3, 127.1, 126.5, 126.1, 125.5, 124.4, 121.6, 81.7, 70.6, 51.0, 37.7.

HRMS (ESI+): m/z calcd for $C_{23}H_{18}ClN_2$ $[M+H]^+$: 357.1159, found 357.1154.

Enantioselectivity: >99% *ee*.

Chiral HPLC method: IA column, 254 nm, 10% isopropanol/hexanes, flow rate 0.8 mL/min, room temperature, t_R (major) = 13.18 min, t_R (minor) = 15.78 min.

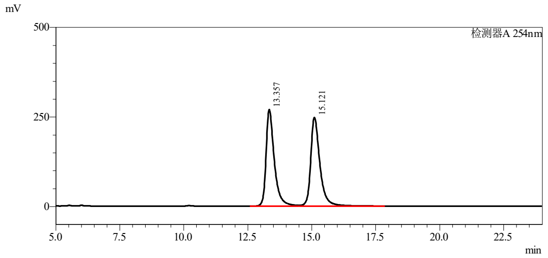

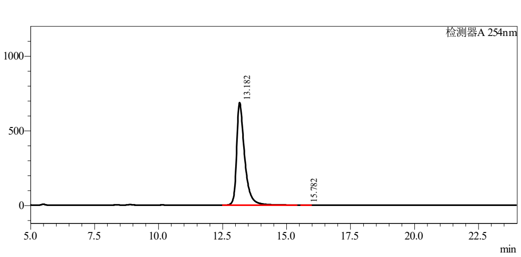

〈峰表〉
检测器A 254nm

峰号	保留时间	面积	高度	浓度
1	13.357	6071674	269499	49.579
2	15.121	6174717	247775	50.421
总计		12246391	517274	

〈峰表〉
检测器A 254nm

峰号	保留时间	面积	高度	浓度
1	13.182	15032500	687407	99.965
2	15.782	5206	291	0.035
总计		15037706	687698	

(*S*)-N-((1-(4-methylquinolin-8-yl)naphthalen-2-yl)methyl)prop-2-yn-1-amine (**5e**)

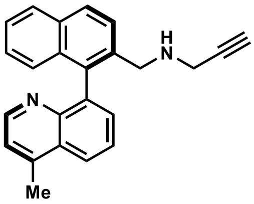

Yellow oil. 68% yield.

^{1}H NMR (400 MHz, CDCl$_3$): δ 8.63 (dd, J = 4.3, 1.5 Hz, 1H), 8.15 (dt, J = 8.5, 1.6 Hz, 1H), 7.94 (d, J = 8.4 Hz, 1H), 7.88 (d, J = 8.2 Hz, 1H), 7.77 – 7.66 (m, 2H), 7.62 (dt, J = 6.9, 1.7 Hz, 1H), 7.44 – 7.33 (m, 1H), 7.23 (td, J = 8.9, 3.9 Hz, 2H), 7.14 (d, J = 8.5 Hz, 1H), 3.62 (dd, J = 12.3, 1.5 Hz, 1H), 3.55 (dd, J = 13.0, 1.7 Hz, 1H), 3.20 (dt, J = 16.8, 2.0 Hz, 1H), 3.12 (dt, J = 16.9, 2.1 Hz, 1H), 2.80 (s, 3H), 1.77 (t, J = 2.2 Hz, 1H).

^{13}C NMR (101 MHz, CDCl$_3$): δ 150.4, 147.5, 144.4, 138.8, 137.0, 135.3, 133.4, 133.0, 131.7, 128.8, 128.1, 128.1, 127.6, 126.7, 126.0, 125.9, 125.4, 124.0, 122.2, 81.8, 70.7, 51.3, 37.7, 19.2.

HRMS (ESI+): m/z calcd for $C_{24}H_{21}N_2$ $[M+H]^+$: 337.1705, found 337.1696.

Enantioselectivity: >99% *ee*.

Chiral HPLC method: ODH column, 254 nm, 10% isopropanol/hexanes, flow rate 0.8 mL/min, room temperature, t_R (major) = 11.12 min, t_R (minor) = 13.49 min.

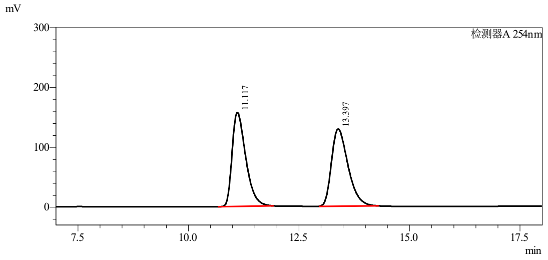

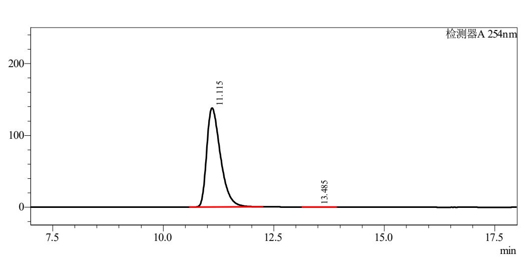

<〈峰表〉
检测器A 254nm

峰号	保留时间	面积	高度	浓度
1	11.117	3386736	157192	50.124
2	13.397	3370028	129071	49.876
总计		6756764	286263	

〈峰表〉
检测器A 254nm

峰号	保留时间	面积	高度	浓度
1	11.115	3001750	138030	99.945
2	13.485	1648	111	0.055
总计		3003399	138141	

(*S*)-N-((1-(5,6-difluoro-2-methylquinolin-8-yl)naphthalen-2-yl)methyl)prop-2-yn-1-amine (**5f**)

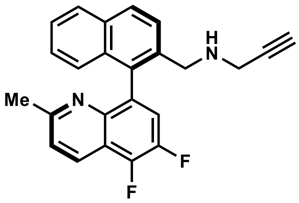

White solid. 36% yield.

^{1}H NMR (400 MHz, CDCl$_3$): δ 8.39 (d, J = 8.7 Hz, 1H), 7.96 (d, J = 8.5 Hz, 1H), 7.90 (d, J = 8.2 Hz, 1H), 7.73 (d, J = 8.5 Hz, 1H), 7.51 – 7.39 (m, 2H), 7.36 (d, J = 8.6 Hz, 1H), 7.31 – 7.23 (m, 1H), 7.17 (d, J = 8.5 Hz, 1H), 3.58 (d, J = 11.8 Hz, 1H), 3.52 (d, J = 11.8 Hz, 1H), 3.27 (dd, J = 17.0, 2.4 Hz, 1H), 3.11 (dd, J = 17.0, 2.4 Hz, 1H), 2.48 (s, 3H), 1.71 (t, J = 2.4 Hz, 1H).

^{13}C NMR (126 MHz, CDCl$_3$): δ 159.4 (d, J = 2.7 Hz), 145.4 (dd, J = 247.8, 11.4 Hz),143.9,143.5 (dd, J = 255.0, 13.0 Hz), 135.9, 134.8 (dd, J = 6.3, 5.1 Hz), 134.7, 133.4, 133.0, 128.9 (dd, J = 6.5, 4.1 Hz), 128.4, 128.1, 127.8, 126.4, 126.1, 125.5, 123.0 (d, J = 3.0 Hz), 122.0 (d, J = 20.3 Hz), 118.4 (dd, J = 12.4, 2.4 Hz), 81.6, 70.6, 51.3, 37.8, 25.5.

^{19}F NMR (470 MHz, CDCl$_3$): δ -141.34 (dd, J = 19.4, 9.4 Hz), -149.01 (dd, J = 20.8, 9.4 Hz).

HRMS (ESI+): m/z calcd for $C_{24}H_{19}F_2N_2$ [M+H]$^+$: 373.1516, found 373.1508.

Enantioselectivity: >99% *ee*.

Chiral HPLC method: ODH column, 254 nm, 10% isopropanol/hexanes, flow rate 0.8 mL/min, room temperature, t_R (major) = 7.72 min, t_R (minor) = 8.84 min.

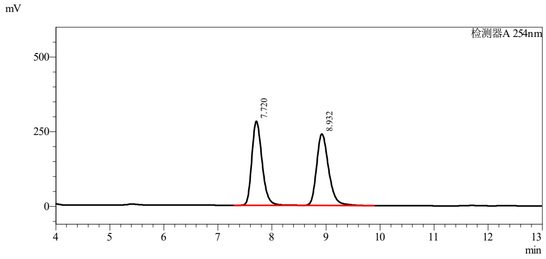

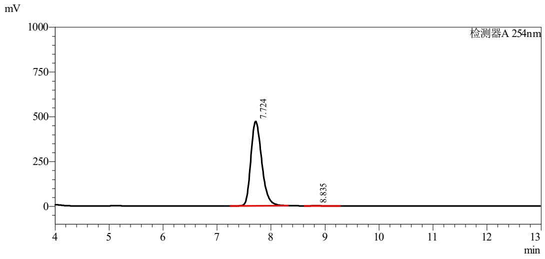

〈峰表〉
检测器A 254nm

峰号	保留时间	面积	高度	浓度
1	7.720	3714876	282014	50.029
2	8.932	3710522	239816	49.971
总计		7425398	521831	

〈峰表〉
检测器A 254nm

峰号	保留时间	面积	高度	浓度
1	7.724	6630400	471614	99.806
2	8.835	12863	758	0.194
总计		6643262	472372	

(S)-N-((1-(5-methoxyquinolin-8-yl)naphthalen-2-yl)methyl)prop-2-yn-1-amine (**5g**)

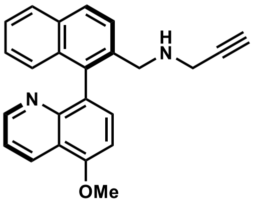

Yellow oil. 27% yield.

^{1}H NMR (400 MHz, CDCl$_3$): δ 8.77 (dd, J = 4.2, 1.8 Hz, 1H), 8.69 (dd, J = 8.5, 1.8 Hz, 1H), 7.92 (dd, J = 21.5, 8.3 Hz, 2H), 7.75 (d, J = 8.5 Hz, 1H), 7.53 (d, J = 7.9 Hz, 1H), 7.44 – 7.33 (m, 2H), 7.28 – 7.19 (m, 2H), 7.01 (d, J = 7.9 Hz, 1H), 4.10 (s, 3H), 3.67 (d, J = 12.1 Hz, 1H), 3.58 (d, J = 12.1 Hz, 1H), 3.23 (dd, J = 16.9, 2.4 Hz, 1H), 3.14 (dd, J = 17.0, 2.4 Hz, 1H), 1.78 (t, J = 2.4 Hz, 1H).

^{13}C NMR (101 MHz, CDCl$_3$): δ 155.1, 150.9, 148.2, 136.7, 135.9, 133.8, 133.0, 131.7, 131.0, 130.0, 128.1, 128.0, 127.6, 126.7, 125.8, 125.3, 121.1, 120.4, 104.0, 81.9, 70.7, 55.9, 51.3, 37.7.

HRMS (ESI+): m/z calcd for $C_{24}H_{21}N_2O$ [M+H]$^+$: 353.1654, found 353.1646.

Enantioselectivity: >99% *ee*.

Chiral HPLC method: IA column, 254 nm, 10% isopropanol/hexanes, flow rate 0.8 mL/min, room temperature, t_R (major) = 16.32 min, t_R (minor) = 23.16 min.

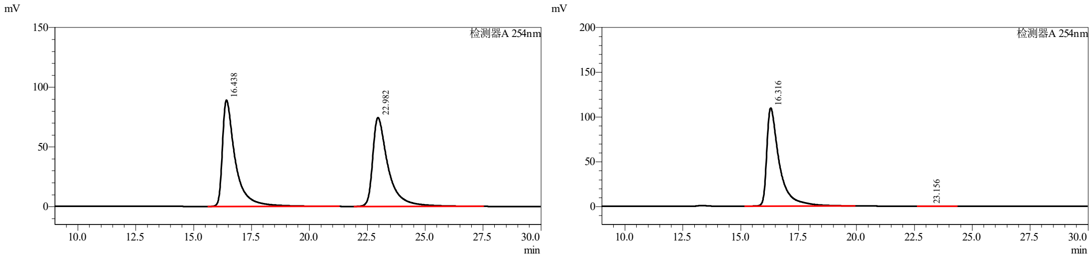

〈峰表〉
检测器A 254nm

峰号	保留时间	面积	高度	浓度
1	16.438	3475973	89063	50.105
2	22.982	3461434	74348	49.895
总计		6937406	163411	

〈峰表〉
检测器A 254nm

峰号	保留时间	面积	高度	浓度
1	16.316	4187821	109668	99.787
2	23.156	8919	184	0.213
总计		4196740	109852	

(S)-N-(3-methyl-2-(quinolin-8-yl)benzyl)prop-2-yn-1-amine (**5h**)

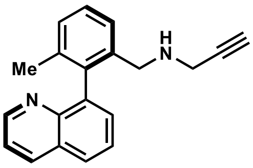

Yellow oil. >99% yield.

^{1}H NMR (400 MHz, CDCl$_3$): δ 8.91 – 8.80 (m, 1H), 8.21 (dt, J = 8.3, 1.4 Hz, 1H), 7.91 – 7.83 (m, 1H), 7.62 (t, J = 7.6 Hz, 1H), 7.54 (dt, J = 7.1, 1.4 Hz, 1H), 7.40 (dd, J = 7.9, 5.1 Hz, 2H), 7.34 (t, J = 7.5 Hz, 1H), 7.29 – 7.23 (m, 1H), 3.47 (d, J = 12.5 Hz, 1H), 3.39 (d, J = 12.5 Hz, 1H), 3.14 (dd, J = 16.4, 1.9 Hz, 1H), 3.07 (dd, J = 16.9, 1.9 Hz, 1H), 1.91 (s, 3H), 1.76 (t, J = 2.8 Hz, 1H).

^{13}C NMR (101 MHz, CDCl$_3$): δ 150.7, 147.0, 139.5, 139.5, 137.8, 137.0, 136.4, 130.7, 128.9, 128.8, 127.9, 127.7, 127.0, 126.4, 121.3, 81.7, 70.7, 50.9, 37.4, 20.8.

HRMS (ESI+): m/z calcd for $C_{20}H_{19}N_2$ [M+H]$^+$: 287.1548, found 287.1539.

Enantioselectivity: 98% *ee*.

Chiral HPLC method: ODH column, 254 nm, 10% isopropanol/hexanes, flow rate 0.8 mL/min, room temperature, t_R (major) = 8.71 min, t_R (minor) = 10.17 min.

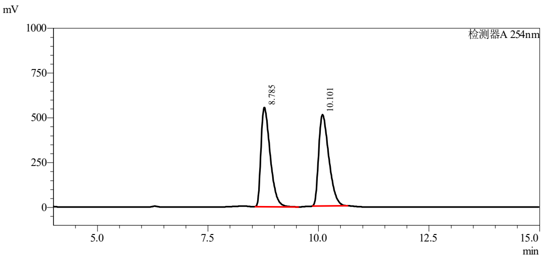

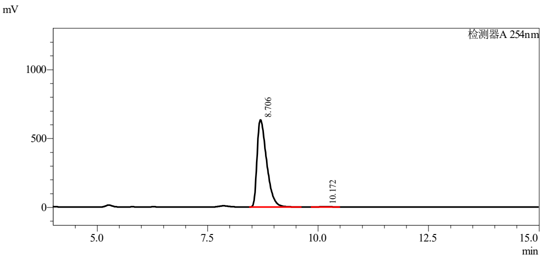

〈峰表〉
检测器A 254nm

峰号	保留时间	面积	高度	浓度
1	8. 785	8073253	554358	49. 645
2	10. 101	8188762	510367	50. 355
总计		16262016	1064725	

〈峰表〉
检测器A 254nm

峰号	保留时间	面积	高度	浓度
1	8. 706	9613683	633053	99. 301
2	10. 172	67656	3943	0. 699
总计		9681339	636996	

(*S*)-N-(3-methyl-2-(5-nitroquinolin-8-yl)benzyl)prop-2-yn-1-amine (**5i**)

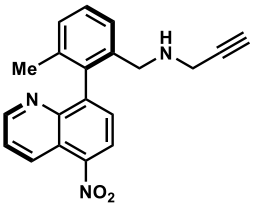

Yellow oil. 60% yield.

^{1}H NMR (400 MHz, CDCl$_3$): δ 9.04 (dd, J = 8.8, 1.7 Hz, 1H), 8.96 (dd, J = 4.1, 1.6 Hz, 1H), 8.43 (d, J = 7.9 Hz, 1H), 7.71 – 7.60 (m, 2H), 7.41 (dt, J = 15.0, 7.6 Hz, 2H), 7.32 – 7.24 (m, 2H), 3.44 (d, J = 12.4 Hz, 1H), 3.34 (d, J = 12.4 Hz, 1H), 3.16 (dd, J = 17.1, 2.4 Hz, 1H), 3.09 (dd, J = 17.2, 2.4 Hz, 1H), 1.89 (s, 3H), 1.75 (t, J = 2.4 Hz, 1H).

^{13}C NMR (101 MHz, CDCl$_3$): δ 151.7, 147.4, 146.9, 145.2, 138.1, 137.4, 136.3, 132.2, 129.1, 129.0, 128.7, 127.0, 124.3, 124.0, 121.7, 81.7, 70.8, 50.6, 37.4, 20.7.

HRMS (ESI+): m/z calcd for $C_{20}H_{18}N_3O_2$ $[M+H]^+$: 332.1399, found 332.1395.

Enantioselectivity: >99% *ee*.

Chiral HPLC method: ODH column, 254 nm, 10% isopropanol/hexanes, flow rate 0.8 mL/min, room temperature, t_R (major) = 10.51 min, t_R (minor) = 16.82 min.

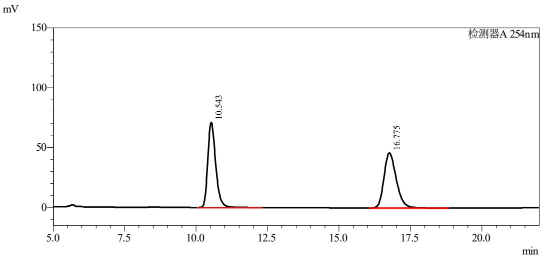

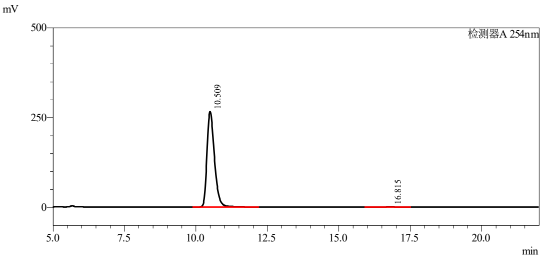

〈峰表〉
检测器A 254nm

峰号	保留时间	面积	高度	浓度
1	10. 543	1375357	71169	50. 060
2	16. 775	1372041	45904	49. 940
总计		2747398	117073	

〈峰表〉
检测器A 254nm

峰号	保留时间	面积	高度	浓度
1	10. 509	4862445	265909	99. 517
2	16. 815	23617	733	0. 483
总计		4886062	266642	

(S)-N-(2-(5-fluoroquinolin-8-yl)-3-methylbenzyl)prop-2-yn-1-amine (**5j**)

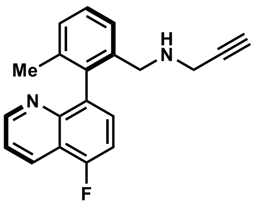

Colorless oil. 95% yield.

^{1}H NMR (400 MHz, CDCl$_3$): δ 8.90 (dd, J = 4.3, 1.8 Hz, 1H), 8.50 (dd, J = 8.4, 1.8 Hz, 1H), 7.47 (ddd, J = 8.4, 5.2, 3.0 Hz, 2H), 7.42 (d, J = 7.6 Hz, 1H), 7.38 – 7.23 (m, 4H), 3.47 (d, J = 12.4 Hz, 1H), 3.38 (d, J = 12.5 Hz, 1H), 3.17 (dd, J = 17.0, 2.4 Hz, 1H), 3.09 (dd, J = 17.0, 2.4 Hz, 1H), 1.90 (s, 3H), 1.77 (t, J = 2.4 Hz, 1H).

^{13}C NMR (126 MHz, CDCl$_3$): δ 157.4 (d, J = 255.2 Hz), 151.5, 147.3 (d, J = 2.9 Hz), 138.7, 138.0, 137.3, 135.4 (d, J = 4.7 Hz), 130.1 (d, J = 8.4 Hz), 129.6 (d, J = 4.9 Hz), 129.0, 128.1, 127.0, 121.4 (d, J = 2.9 Hz), 119.5 (d, J = 16.1 Hz), 110.0 (d, J = 19.1 Hz), 81.6, 70.8, 50.9, 37.5, 20.8.

^{19}F NMR (470 MHz, CDCl$_3$): δ -123.45.

HRMS (ESI+): m/z calcd for $C_{20}H_{18}FN_2$ $[M+H]^+$: 305.1454, found 305.1451.

Enantioselectivity: >99% *ee*.

Chiral HPLC method: ODH column, 254 nm, 10% isopropanol/hexanes, flow rate 0.8 mL/min, room temperature, t_R (major) = 8.01 min, t_R (minor) = 10.91 min.

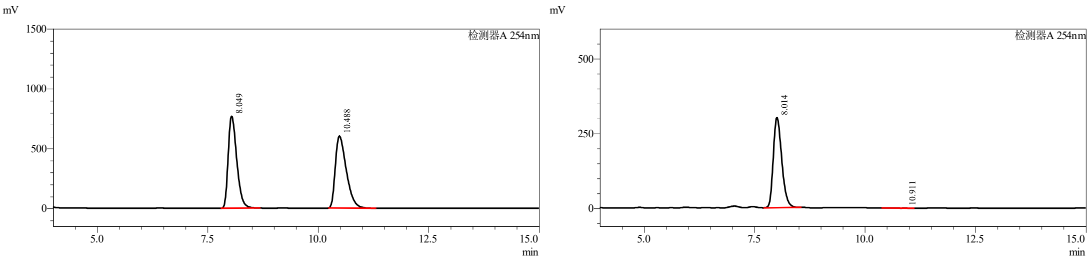

〈峰表〉
检测器A 254nm

峰号	保留时间	面积	高度	浓度
1	8.049	10396095	769273	49.955
2	10.488	10414837	601484	50.045
总计		20810932	1370757	

〈峰表〉
检测器A 254nm

峰号	保留时间	面积	高度	浓度
1	8.014	4028039	301708	99.938
2	10.911	2511	54	0.062
总计		4030549	301762	

(S)-N-(2-(5,6-difluoro-2-methylquinolin-8-yl)-3-methylbenzyl)prop-2-yn-1-amine (**5k**)

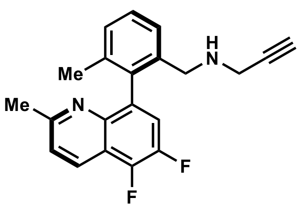

Colorless oil. 82% yield.

^{1}H NMR (400 MHz, CDCl$_3$): δ 8.35 (d, J = 8.6 Hz, 1H), 7.41 – 7.30 (m, 4H), 7.24 (d, J = 7.3 Hz, 1H), 3.43 (d, J = 12.1 Hz, 1H), 3.36 (d, J = 12.1 Hz, 1H), 3.17 (dd, J = 16.9, 2.4 Hz, 1H), 3.04 (dd, J = 16.9, 2.4 Hz, 1H), 2.57 (s, 3H), 1.90 (s, 3H), 1.75 (t, J = 2.4 Hz, 1H).

^{13}C NMR (126 MHz, CDCl$_3$): δ 159.2 (d, J = 2.7 Hz), 145.5 (dd, J = 247.4, 11.4 Hz), 143.3 (dd, J = 254.4, 13.0 Hz), 143.2, 138.1, 138.0, 137.1, 136.1 (dd, J = 6.4, 4.8 Hz), 128.9 (dd, J = 6.6, 4.2

Hz), 128.8, 127.9, 127.1, 122.8 (d, J = 3.1 Hz), 120.7 (d, J = 20.4 Hz), 118.4 (dd, J = 12.2, 2.5 Hz), 81.8, 70.5, 51.1, 37.5, 25.6, 20.9.

^{19}F NMR (470 MHz, CDCl$_3$): δ -141.34 (dd, J = 20.4, 10.1 Hz), -149.64 (dd, J = 19.7, 9.9 Hz).

HRMS (ESI+): m/z calcd for $C_{21}H_{19}F_2N_2$ $[M+H]^+$: 337.1516, found 337.1517.

Enantioselectivity: >99% *ee.*

Chiral HPLC method: ODH column, 254 nm, 5% isopropanol/hexanes, flow rate 0.8 mL/min, room temperature, t_R (major) = 7.51 min, t_R (minor) = 10.27 min.

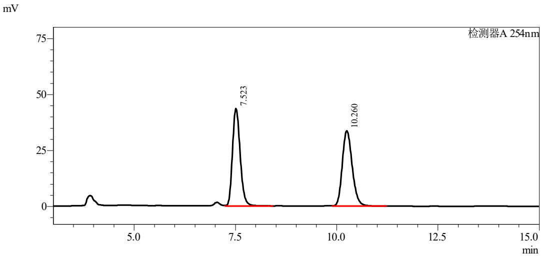

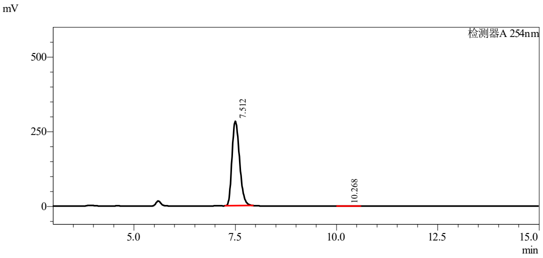

〈峰表〉
检测器A 254nm

峰号	保留时间	面积	高度	浓度
1	7.523	575355	43459	50.034
2	10.260	574585	33692	49.966
总计		1149940	77151	

〈峰表〉
检测器A 254nm

峰号	保留时间	面积	高度	浓度
1	7.512	3697271	282968	99.890
2	10.268	4073	248	0.110
总计		3701344	283216	

(S)-N-(3-methyl-2-(4-methylquinolin-8-yl)benzyl)prop-2-yn-1-amine (**5l**)

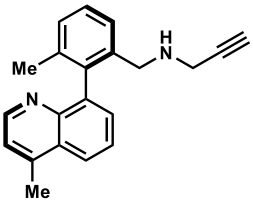

Yellow oil. 70% yield.

^{1}H NMR (400 MHz, CDCl$_3$): δ 8.70 (dd, J = 4.3, 2.0 Hz, 1H), 8.06 (dt, J = 8.4, 1.8 Hz, 1H), 7.63 (ddd, J = 8.9, 7.0, 2.0 Hz, 1H), 7.51 (dd, J = 7.0, 1.5 Hz, 1H), 7.39 (d, J = 7.6 Hz, 1H), 7.33 (td, J = 7.5, 2.0 Hz, 1H), 7.27 – 7.20 (m, 2H), 3.45 (dd, J = 12.4, 2.0 Hz, 1H), 3.37 (dd, J = 12.5, 2.1 Hz, 1H), 3.13 (dd, J = 16.9, 2.3 Hz, 1H), 3.06 (dd, J = 16.9, 2.3 Hz, 1H), 2.76 (s, 3H), 1.90 (s, 3H), 1.78 (t, J = 2.4 Hz, 1H).

^{13}C NMR (101 MHz, CDCl$_3$): δ 150.3, 146.7, 144.4, 140.0, 140.0, 137.8, 136.9, 130.4, 128.9, 128.8, 127.8, 126.8, 126.1, 123.7, 122.1, 82.0, 70.6, 51.1, 37.5, 20.8, 19.1.

HRMS (ESI+): m/z calcd for $C_{21}H_{21}N_2$ $[M+H]^+$: 301.1705, found 301.1698.

Enantioselectivity: 98% *ee.*

Chiral HPLC method: ODH column, 254 nm, 10% isopropanol/hexanes, flow rate 0.8 mL/min, room temperature, t_R (major) = 8.48 min, t_R (minor) = 11.30 min.

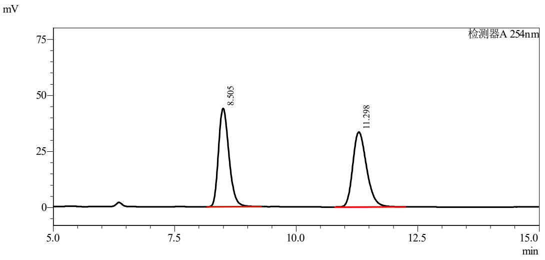

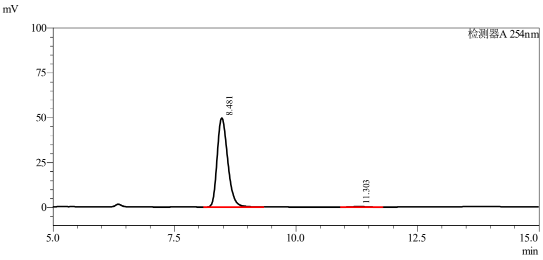

〈峰表〉
检测器A 254nm

峰号	保留时间	面积	高度	浓度
1	8.505	660973	43827	49.956
2	11.298	662130	33371	50.044
总计		1323103	77198	

〈峰表〉
检测器A 254nm

峰号	保留时间	面积	高度	浓度
1	8.481	751725	49634	99.212
2	11.303	5971	309	0.788
总计		757696	49942	

(*S*)-N-((1-(quinoxalin-5-yl)naphthalen-2-yl)methyl)prop-2-yn-1-amine (**5m**)

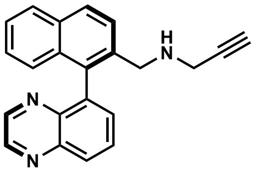

Yellow oil. 81% yield.

^{1}H NMR (400 MHz, CDCl$_3$): *δ* 8.85 (d, *J* = 1.8 Hz, 1H), 8.71 (d, *J* = 1.8 Hz, 1H), 8.26 (dt, *J* = 8.5, 1.6 Hz, 1H), 8.02 – 7.86 (m, 3H), 7.75 (ddd, *J* = 8.6, 6.6, 1.7 Hz, 2H), 7.43 (td, *J* = 7.7, 2.1 Hz, 1H), 7.29 – 7.20 (m, 2H), 7.11 (d, *J* = 8.4 Hz, 1H), 3.62 (dd, *J* = 12.2, 1.9 Hz, 1H), 3.54 (dd, *J* = 12.2, 1.9 Hz, 1H), 3.23 (dt, *J* = 17.1, 2.1 Hz, 1H), 3.14 (dt, *J* = 17.1, 2.1 Hz, 1H), 1.68 (t, *J* = 2.3 Hz, 1H).

^{13}C NMR (101 MHz, CDCl$_3$): *δ* 145.3, 145.2, 143.4, 142.7, 138.8, 135.8, 135.1, 133.3, 132.9, 132.5, 129.8, 129.6, 128.7, 128.2, 127.5, 126.4, 126.2, 125.7, 81.4, 70.8, 50.7, 37.6.

HRMS (ESI+): m/z calcd for $C_{22}H_{18}N_3$ [M+H]$^+$: 324.1501, found 324.1493.

Enantioselectivity: 97% *ee*.

Chiral HPLC method: ODH column, 254 nm, 10% isopropanol/hexanes, flow rate 0.8 mL/min, room temperature, t_R (major) = 16.52 min, t_R (minor) = 15.02 min.

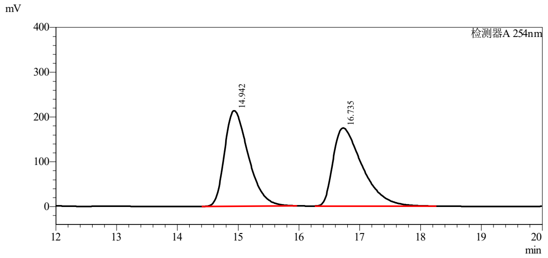

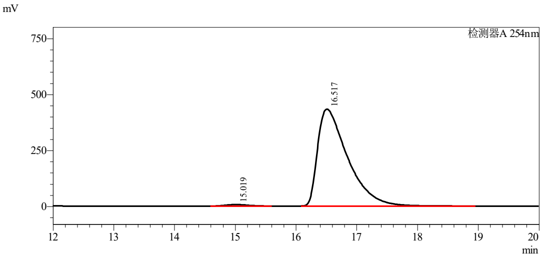

〈峰表〉
检测器A 254nm

峰号	保留时间	面积	高度	浓度
1	14.942	5837637	213228	49.969
2	16.735	5844916	174204	50.031
总计		11682553	387432	

〈峰表〉
检测器A 254nm

峰号	保留时间	面积	高度	浓度
1	15.019	174757	6811	1.158
2	16.517	14922839	432460	98.842
总计		15097597	439271	

(*S*)-N-((1-(benzofuran-7-yl)naphthalen-2-yl)methyl)prop-2-yn-1-amine (**5n**)

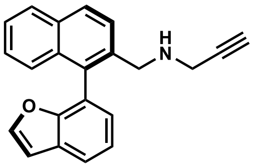

Colorless oil. 55% yield.

^{1}H NMR (400 MHz, CDCl$_3$): *δ* 7.92 (d, *J* = 8.5 Hz, 1H), 7.87 (d, *J* = 8.2 Hz, 1H), 7.70 (dd, *J* = 11.8, 8.1 Hz, 2H), 7.49 (d, *J* = 2.2 Hz, 1H), 7.42 (ddd, *J* = 8.1, 5.6, 2.2 Hz, 1H), 7.36 (t, *J* = 7.6 Hz, 1H), 7.32 – 7.24 (m, 2H), 7.24 – 7.17 (m, 1H), 6.83 (d, *J* = 2.2 Hz, 1H), 3.74 (d, *J* = 12.7 Hz, 1H), 3.66 (d, *J* = 12.8 Hz, 1H), 3.21 (t, *J* = 2.5 Hz, 2H), 1.91 (t, *J* = 2.4 Hz, 1H).

^{13}C NMR (101 MHz, CDCl$_3$): δ 153.5, 145.4, 135.9, 133.1, 132.9, 132.9, 128.6, 128.1, 127.8, 127.4, 126.7, 126.3, 126.3, 125.7, 123.1, 122.5, 120.9, 106.9, 81.8, 71.1, 50.6, 37.7.

HRMS (ESI+): m/z calcd for $C_{22}H_{18}NO$ [M+H]$^+$: 312.1388, found 312.1380.

Enantioselectivity: 60% *ee*.

Chiral HPLC method: IA column, 254 nm, 5% isopropanol/hexanes, flow rate 0.8 mL/min, room temperature, t_R (major) = 17.73 min, t_R (minor) = 18.86 min.

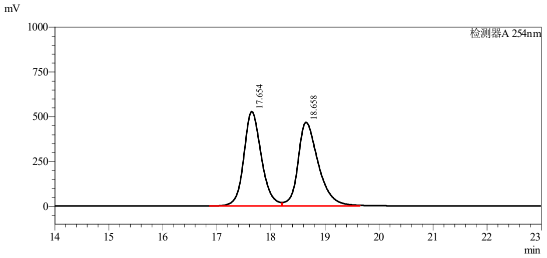

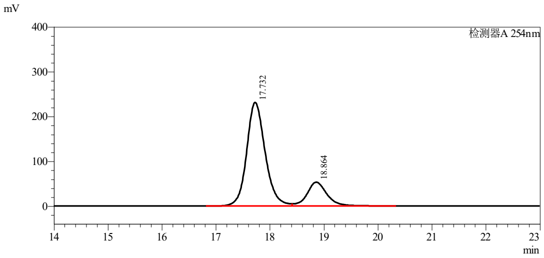

〈峰表〉
检测器A 254nm

峰号	保留时间	面积	高度	浓度
1	17.654	11929228	526398	49.094
2	18.658	12369514	466779	50.906
总计		24298742	993177	

〈峰表〉
检测器A 254nm

峰号	保留时间	面积	高度	浓度
1	17.732	5523393	231381	80.018
2	18.864	1379316	53232	19.982
总计		6902709	284613	

(*S*)-N-((1-(benzo[b]thiophen-7-yl)naphthalen-2-yl)methyl)prop-2-yn-1-amine (**5o**)

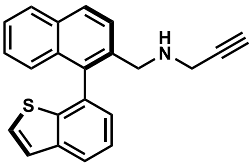

Colorless oil. 36% yield.

^{1}H NMR (400 MHz, CDCl$_3$): δ 7.99 – 7.87 (m, 3H), 7.76 (d, J = 8.5 Hz, 1H), 7.53 (t, J = 7.6 Hz, 1H), 7.45 (dd, J = 8.7, 5.7 Hz, 2H), 7.38 (d, J = 5.4 Hz, 1H), 7.28 (t, J = 7.3 Hz, 3H), 3.76 (d, J = 12.9 Hz, 1H), 3.67 (d, J = 12.9 Hz, 1H), 3.25 (t, J = 1.9 Hz, 2H), 1.96 (t, J = 2.4 Hz, 1H).

^{13}C NMR (101 MHz, CDCl$_3$): δ 141.5, 139.9, 136.6, 135.4, 133.4, 133.0, 132.2, 128.7, 128.1, 127.3, 127.1, 126.3, 126.2, 126.1, 125.8, 124.7, 124.3, 123.0, 81.9, 71.2, 50.4, 37.8.

HRMS (ESI+): m/z calcd for $C_{22}H_{18}NS$ [M+H]$^+$: 328.1160, found 328.1154.

Enantioselectivity: 89% *ee*.

Chiral HPLC method: ODH column, 254 nm, 10% isopropanol/hexanes, flow rate 0.8 mL/min, room temperature, t_R (major) = 9.74 min, t_R (minor) = 11.25 min.

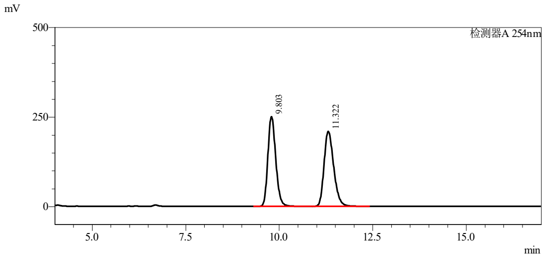

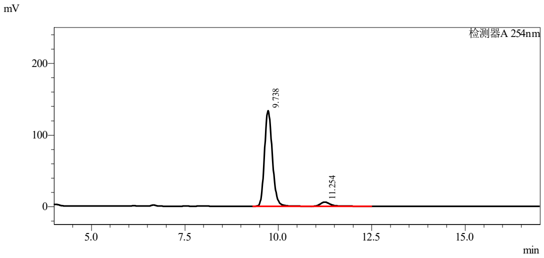

〈峰表〉
检测器A 254nm

峰号	保留时间	面积	高度	浓度
1	9.803	3681131	250561	49.408
2	11.322	3769416	209403	50.592
总计		7450547	459965	

〈峰表〉
检测器A 254nm

峰号	保留时间	面积	高度	浓度
1	9.738	1951921	133289	94.542
2	11.254	112683	5771	5.458
总计		2064604	139060	

(*S*)-N-(2-(5-methoxyquinolin-8-yl)-3-methylbenzyl)prop-2-yn-1-amine (**5p**)

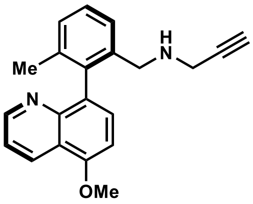

Yellow oil. 26% yield.

^{1}H NMR (400 MHz, CDCl$_3$): δ 8.84 (dt, J = 4.2, 1.4 Hz, 1H), 8.63 (dt, J = 8.5, 1.4 Hz, 1H), 7.45 – 7.28 (m, 4H), 7.28 – 7.22 (m, 1H), 6.94 (d, J = 7.9 Hz, 1H), 4.06 (s, 3H), 3.49 (d, J = 12.4 Hz, 1H), 3.39 (d, J = 12.4 Hz, 1H), 3.14 (dd, J = 16.9, 2.4 Hz, 1H), 3.06 (dd, J = 16.8, 2.4 Hz, 1H), 1.92 (s, 3H), 1.77 (t, J = 2.5 Hz, 1H).

^{13}C NMR (101 MHz, CDCl$_3$): δ 154.8, 150.9, 147.5, 139.5, 138.4, 137.5, 131.3, 130.9, 130.3, 128.8, 127.7, 126.9, 121.1, 120.3, 104.1, 81.9, 70.6, 55.9, 51.2, 37.5, 20.8.

HRMS (ESI+): m/z calcd for $C_{21}H_{21}N_2O$ [M+H]$^+$: 317.1654, found 317.1650.

Enantioselectivity: 89% *ee*.

Chiral HPLC method: ODH column, 254 nm, 10% isopropanol/hexanes, flow rate 0.8 mL/min, room temperature, t_R (major) = 9.31 min, t_R (minor) = 10.42 min.

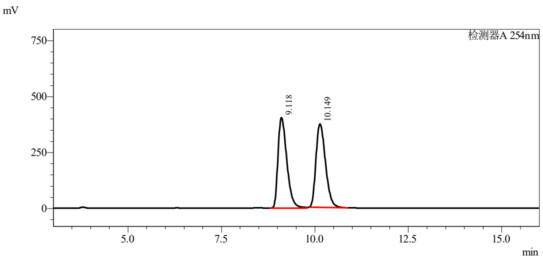

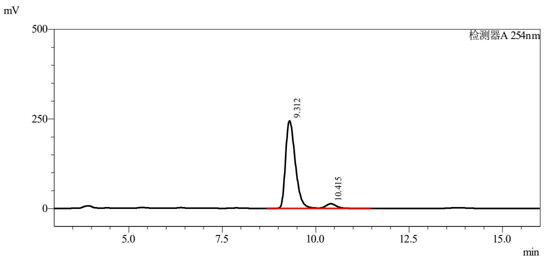

〈峰表〉
检测器A 254nm

峰号	保留时间	面积	高度	浓度
1	9.118	6699804	404068	50.305
2	10.149	6618497	372566	49.695
总计		13318300	776634	

〈峰表〉
检测器A 254nm

峰号	保留时间	面积	高度	浓度
1	9.312	4448442	244428	94.502
2	10.415	258780	12897	5.498
总计		4707222	257325	

(*S*)-N-((1-(quinolin-8-yl)naphthalen-2-yl)methyl)prop-2-en-1-amine (**6a**)

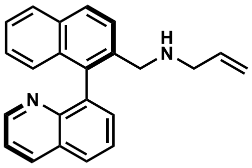

Yellow oil. 97% yield.

^{1}H NMR (400 MHz, CDCl$_3$): δ 8.80 – 8.71 (m, 1H), 8.27 (dd, J = 8.3, 1.8 Hz, 1H), 7.96 (t, J = 7.8 Hz, 2H), 7.90 (d, J = 8.2 Hz, 1H), 7.78 – 7.61 (m, 3H), 7.40 (dt, J = 8.6, 4.6 Hz, 2H), 7.28 – 7.20 (m, 1H), 7.15 (d, J = 8.5 Hz, 1H), 5.57 (ddt, J = 16.6, 11.2, 6.0 Hz, 1H), 4.97 – 4.80 (m, 2H), 3.53 (d, J = 2.4 Hz, 2H), 3.01 (dd, J = 14.1, 5.8 Hz, 1H), 2.89 (dd, J = 14.1, 6.2 Hz, 1H).

^{13}C NMR (126 MHz, CDCl$_3$): δ 150.7, 147.7, 138.5, 136.6, 136.5, 136.3, 136.1, 133.5, 132.9, 131.9, 128.7, 128.1, 128.1, 128.1, 127.5, 126.6, 126.3, 125.9, 125.3, 121.3, 115.7, 51.9.

HRMS (ESI+): m/z calcd for $C_{23}H_{21}N_2$ [M+H]$^+$: 325.1705, found 325.1714.

Enantioselectivity: >99% *ee*.

Chiral HPLC method: ASH column, 254 nm, 2% isopropanol/hexanes, flow rate 0.5 mL/min, room temperature, t_R (major) = 21.45 min, t_R (minor) = 17.63 min.

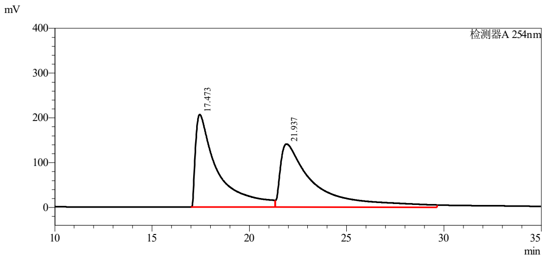

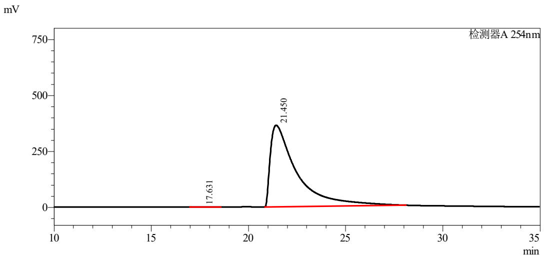

〈峰表〉
检测器A 254nm

峰号	保留时间	面积	高度	浓度
1	17.473	16390557	206437	48.954
2	21.937	17091015	140666	51.046
总计		33481572	347103	

〈峰表〉
检测器A 254nm

峰号	保留时间	面积	高度	浓度
1	17.631	9190	194	0.027
2	21.450	34669169	364036	99.973
总计		34678359	364230	

(*S*)-N-((1-(quinolin-8-yl)naphthalen-2-yl)methyl)but-3-yn-1-amine (**6b**)

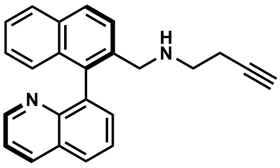

Yellow oil. 97% yield.

^{1}H NMR (400 MHz, $CDCl_3$): δ 8.79 (dd, J = 4.2, 1.8 Hz, 1H), 8.27 (dd, J = 8.3, 1.8 Hz, 1H), 7.96 (dd, J = 9.0, 7.3 Hz, 2H), 7.89 (d, J = 8.2 Hz, 1H), 7.76 – 7.62 (m, 3H), 7.40 (dd, J = 8.0, 4.6 Hz, 2H), 7.27 – 7.19 (m, 1H), 7.13 (d, J = 8.5 Hz, 1H), 3.59 (d, J = 13.0 Hz, 1H), 3.53 (d, J = 13.0 Hz, 1H), 2.52 (ddt, J = 18.3, 11.8, 5.8 Hz, 2H), 2.09 (td, J = 6.8, 2.7 Hz, 2H), 1.86 (t, J = 2.6 Hz, 1H).

^{13}C NMR (101 MHz, $CDCl_3$): δ 150.8, 147.6, 138.4, 136.5, 136.2, 136.0, 133.4, 132.9, 131.9, 128.7, 128.2, 128.1, 127.2, 126.6, 126.3, 125.9, 125.3, 121.4, 82.7, 69.2, 51.8, 47.5, 19.5.

HRMS (ESI+): m/z calcd for $C_{24}H_{21}N_2$ $[M+H]^+$: 337.1705, found 337.1714.

Enantioselectivity: >99% *ee*.

Chiral HPLC method: IA column, 254 nm, 10% isopropanol/hexanes, flow rate 0.8 mL/min, room temperature, t_R (major) = 8.43 min, t_R (minor) = 9.77 min.

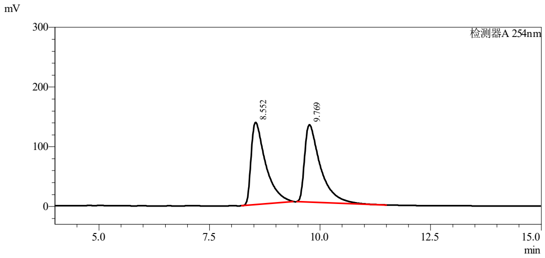

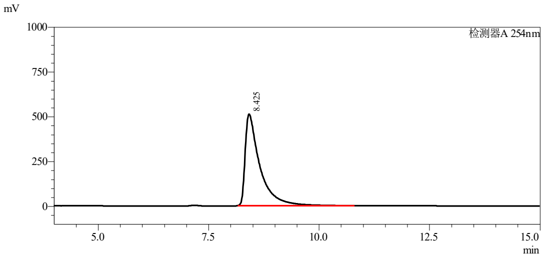

〈峰表〉
检测器A 254nm

峰号	保留时间	面积	高度	浓度
1	8.552	3173385	137885	49.521
2	9.769	3234746	129666	50.479
总计		6408131	267550	

〈峰表〉
检测器A 254nm

峰号	保留时间	面积	高度	浓度
1	8.425	11799646	511001	100.000
总计		11799646	511001	

(*S*)-3,3,3-trifluoro-N-((1-(quinolin-8-yl)naphthalen-2-yl)methyl)propan-1-amine (**6c**)

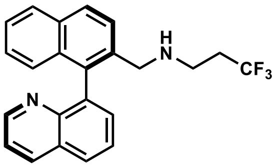

Yellow solid. 54% yield.

^{1}H NMR (500 MHz, CDCl$_3$): δ 8.78 (dd, J = 4.2, 1.8 Hz, 1H), 8.28 (dd, J = 8.3, 1.8 Hz, 1H), 7.97 (dd, J = 10.2, 7.8 Hz, 2H), 7.90 (d, J = 8.2 Hz, 1H), 7.75 – 7.66 (m, 2H), 7.64 (dd, J = 7.0, 1.6 Hz, 1H), 7.45 – 7.36 (m, 2H), 7.29 – 7.22 (m, 1H), 7.15 (d, J = 8.5 Hz, 1H), 3.55 (s, 2H), 2.60 (dt, J = 12.3, 7.2 Hz, 1H), 2.51 (dt, J = 12.3, 7.2 Hz, 1H), 2.05 – 1.92 (m, 2H).

^{13}C NMR (126 MHz, CDCl$_3$): δ 150.8, 147.6, 138.4, 136.5, 136.3, 135.6, 133.5, 133.0, 131.9, 128.7, 128.3, 128.1, 127.2, 126.7 (d, J = 276.7 Hz), 126.6, 126.3, 126.0, 125.5, 121.4, 52.3, 42.1 (q, J = 3.3 Hz), 34.4 (q, J = 27.5 Hz).

^{19}F NMR (470 MHz, CDCl$_3$): δ -65.00.

HRMS (ESI+): m/z calcd for $C_{23}H_{20}F_3N_2$ [M+H]$^+$: 381.1579, found 381.1576.

Enantioselectivity: >99% *ee*.

Chiral HPLC method: ODH column, 254 nm, 10% isopropanol/hexanes, flow rate 0.8 mL/min, room temperature, t_R (major) = 8.53 min, t_R (minor) = 9.68 min.

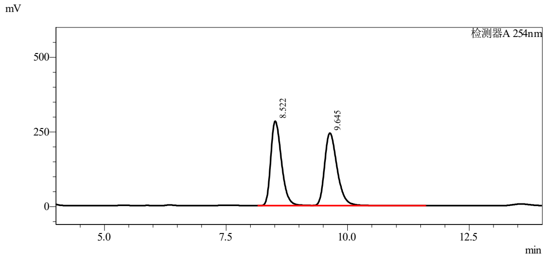

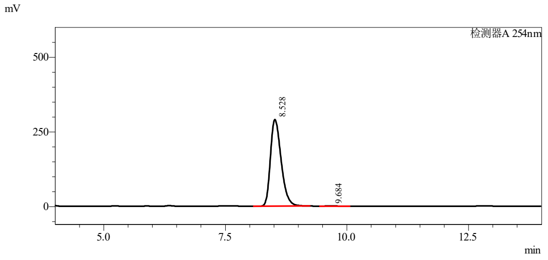

〈峰表〉
检测器A 254nm

峰号	保留时间	面积	高度	浓度
1	8. 522	4350377	283304	49. 804
2	9. 645	4384547	242493	50. 196
总计		8734924	525796	

〈峰表〉
检测器A 254nm

峰号	保留时间	面积	高度	浓度
1	8. 528	4463579	290257	99. 816
2	9. 684	8232	551	0. 184
总计		4471812	290808	

(*S*)-N-((1-(quinolin-8-yl)naphthalen-2-yl)methyl)cyclopropanamine (**6d**)

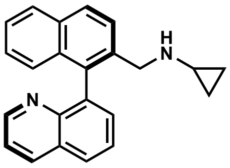

Yellow oil. 54% yield.

^{1}H NMR (400 MHz, CDCl$_3$): δ 8.79 (dd, J = 4.2, 1.8 Hz, 1H), 8.27 (dd, J = 8.2, 1.8 Hz, 1H), 8.01 – 7.85 (m, 3H), 7.75 – 7.59 (m, 3H), 7.45 – 7.36 (m, 2H), 7.26 – 7.09 (m, 2H), 3.58 (d, J = 2.7 Hz, 2H), 1.89 (tt, J = 6.9, 3.6 Hz, 1H), 0.26 – 0.15 (m, 2H), 0.09 (dt, J = 10.4, 3.6 Hz, 1H), 0.06 – -0.04 (m, 1H).

^{13}C NMR (101 MHz, CDCl$_3$): δ 150.7, 147.7, 138.6, 136.4, 136.2, 133.5, 132.8, 131.9, 128.7, 128.1, 128.1, 128.0, 127.7, 126.6, 126.3, 125.8, 125.3, 121.3, 52.2, 30.1, 6.5, 6.2.

HRMS (ESI+): m/z calcd for $C_{23}H_{21}N_2$ [M+H]$^+$: 325.1705, found 325.1715.

Enantioselectivity: >99% *ee*.

Chiral HPLC method: IC column, 254 nm, 5% isopropanol/hexanes, flow rate 0.8 mL/min, room temperature, t_R (major) = 18.93 min, t_R (minor) = 23.15 min.

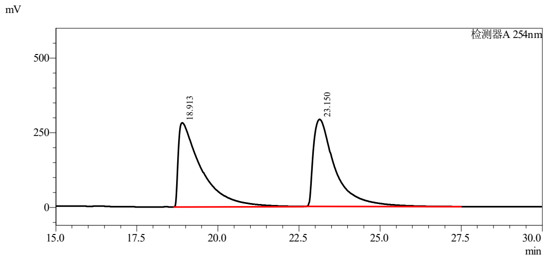

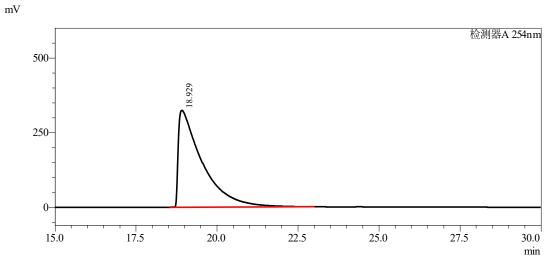

〈峰表〉

检测器A 254nm

峰号	保留时间	面积	高度	浓度
1	18.913	14068117	281585	49.814
2	23.150	14173082	293202	50.186
总计		28241199	574787	

〈峰表〉

检测器A 254nm

峰号	保留时间	面积	高度	浓度
1	18.929	16631116	324033	100.000
总计		16631116	324033	

(*S*)-1-cyclopropyl-N-((1-(quinolin-8-yl)naphthalen-2-yl)methyl)methanamine (**6e**)

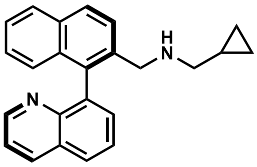

Yellow oil. 90% yield.

^{1}H NMR (400 MHz, CDCl$_3$): δ 8.79 (dd, J = 4.2, 1.7 Hz, 1H), 8.27 (dd, J = 8.4, 1.8 Hz, 1H), 7.96 (dd, J = 8.8, 6.7 Hz, 2H), 7.89 (d, J = 8.2 Hz, 1H), 7.76 – 7.61 (m, 3H), 7.44 – 7.35 (m, 2H), 7.27 – 7.18 (m, 1H), 7.12 (d, J = 8.5 Hz, 1H), 3.58 (s, 2H), 2.29 (dd, J = 12.1, 6.7 Hz, 1H), 2.10 (dd, J = 12.1, 7.1 Hz, 1H), 0.66 (qq, J = 7.4, 3.8 Hz, 1H), 0.24 (qq, J = 8.5, 4.3 Hz, 2H), -0.16 (dq, J = 9.0, 4.0 Hz, 1H), -0.24 (dq, J = 8.1, 4.1 Hz, 1H).

^{13}C NMR (101 MHz, CDCl$_3$): δ 150.8, 147.7, 138.6, 136.5, 136.2, 136.2, 133.5, 132.9, 131.9, 128.7, 128.2, 128.1, 128.1, 127.5, 126.6, 126.3, 125.9, 125.3, 121.4, 54.6, 52.5, 11.1, 3.4, 3.1.

HRMS (ESI+): m/z calcd for $C_{24}H_{23}N_2$ [M+H]$^+$: 339.1861, found 339.1858.

Enantioselectivity: >99% *ee*.

Chiral HPLC method: ODH column, 254 nm, 2% isopropanol/hexanes, flow rate 0.5 mL/min, room temperature, t_R (major) = 42.52 min, t_R (minor) = 46.01 min.

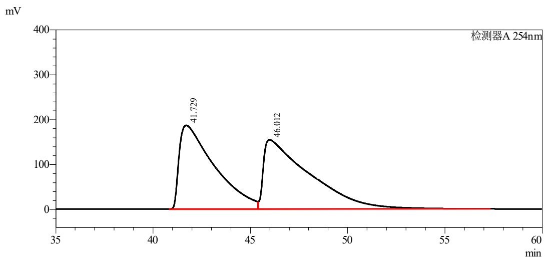

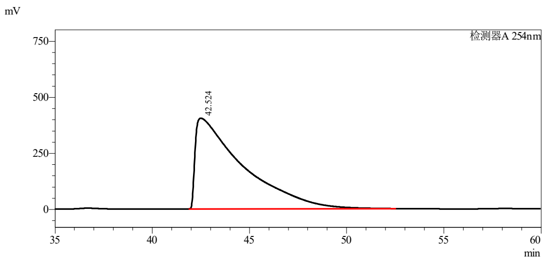

〈峰表〉

检测器A 254nm

峰号	保留时间	面积	高度	浓度
1	41.729	23522135	186340	48.023
2	46.012	25458492	154084	51.977
总计		48980628	340424	

〈峰表〉

检测器A 254nm

峰号	保留时间	面积	高度	浓度
1	42.524	69737232	404935	100.000
总计		69737232	404935	

(*S*)-N-((1-(quinolin-8-yl)naphthalen-2-yl)methyl)cyclobutanamine (**6f**)

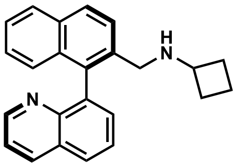

Colorless oil. 79% yield.

^{1}H NMR (400 MHz, CDCl$_3$): δ 8.81 (dd, J = 4.2, 1.8 Hz, 1H), 8.28 (dd, J = 8.3, 1.8 Hz, 1H), 8.01 – 7.92 (m, 2H), 7.89 (d, J = 8.2 Hz, 1H), 7.78 – 7.62 (m, 3H), 7.46 – 7.36 (m, 2H), 7.23 (dd, J = 6.8, 1.4 Hz, 1H), 7.19 – 7.11 (m, 1H), 3.43 (s, 2H), 3.05 (p, J = 7.4 Hz, 1H), 1.95 – 1.85 (m, 1H), 1.84 – 1.74 (m, 1H), 1.42 (pd, J = 6.5, 4.0 Hz, 2H), 1.37 – 1.26 (m, 1H), 0.93 – 0.77 (m, 1H).

^{13}C NMR (101 MHz, CDCl$_3$): δ 150.6, 147.6, 138.4, 136.6, 136.2, 135.8, 133.4, 132.9, 132.0, 128.7, 128.2, 128.1, 128.1, 127.8, 126.5, 126.3, 125.9, 125.3, 121.4, 53.6, 49.5, 30.5, 30.0, 14.6.

HRMS (ESI+): m/z calcd for $C_{24}H_{23}N_2$ [M+H]$^+$: 339.1861, found 339.1857.

Enantioselectivity: >99% *ee*.

Chiral HPLC method: ASH column, 254 nm, 1% isopropanol/hexanes, flow rate 0.8 mL/min, room temperature, t_R (major) = 18.50 min, t_R (minor) = 13.53 min.

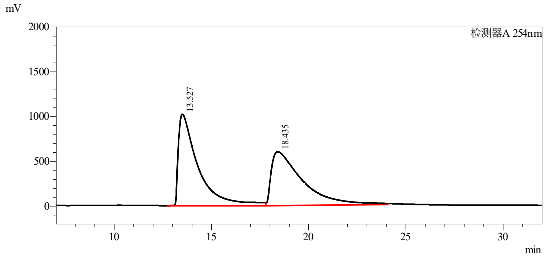

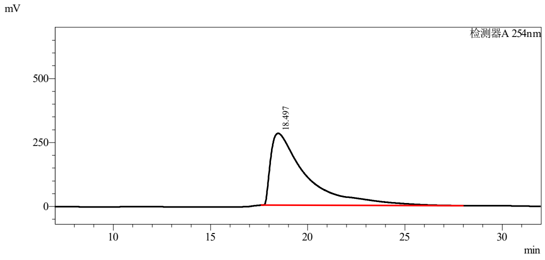

〈峰表〉
检测器A 254nm

峰号	保留时间	面积	高度	浓度
1	13.527	72268597	1022589	49.608
2	18.435	73411723	601708	50.392
总计		145680320	1624297	

〈峰表〉
检测器A 254nm

峰号	保留时间	面积	高度	浓度
1	18.497	36104715	280782	100.000
总计		36104715	280782	

(*S*)-N-((1-(quinolin-8-yl)naphthalen-2-yl)methyl)oxetan-3-amine (**6g**)

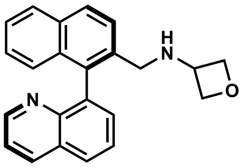

Colorless oil. 96% yield.

^{1}H NMR (400 MHz, CDCl$_3$): δ 8.84 (dd, J = 4.2, 1.8 Hz, 1H), 8.30 (dd, J = 8.3, 1.8 Hz, 1H), 8.02 – 7.93 (m, 2H), 7.90 (d, J = 8.2 Hz, 1H), 7.74 – 7.67 (m, 2H), 7.64 (dd, J = 7.0, 1.6 Hz, 1H), 7.49 – 7.37 (m, 2H), 7.28 – 7.21 (m, 1H), 7.15 (d, J = 8.5 Hz, 1H), 4.49 (t, J = 6.8 Hz, 1H), 4.43 (t, J = 6.6 Hz, 1H), 4.04 (t, J = 6.3 Hz, 1H), 3.77 (p, J = 6.5 Hz, 1H), 3.57 (t, J = 6.2 Hz, 1H), 3.44 (s, 2H).

^{13}C NMR (101 MHz, CDCl$_3$): δ 150.9, 147.6, 138.3, 136.6, 136.4, 135.5, 133.4, 133.0, 132.0, 128.7, 128.4, 128.3, 128.1, 127.4, 126.6, 126.4, 126.1, 125.6, 121.6, 79.4, 53.2, 50.0.

HRMS (ESI+): m/z calcd for $C_{23}H_{21}N_2O$ [M+H]$^+$: 341.1654, found 341.1649.

Enantioselectivity: >99% *ee*.

Chiral HPLC method: IA column, 254 nm, 10% isopropanol/hexanes, flow rate 0.8 mL/min, room temperature, t_R (major) = 11.32 min, t_R (minor) = 15.26 min.

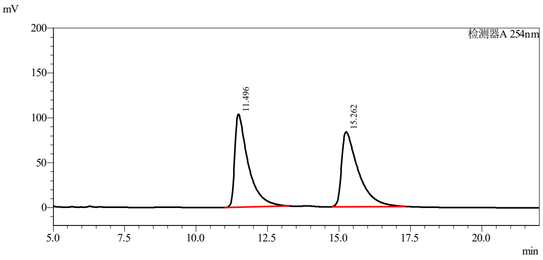

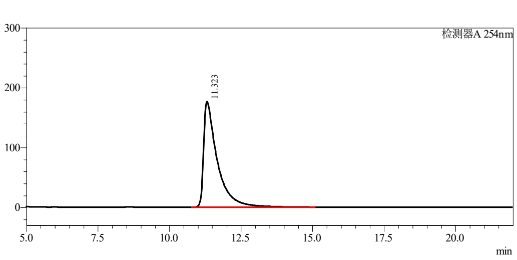

〈峰表〉
检测器A 254nm

峰号	保留时间	面积	高度	浓度
1	11.496	3423574	103586	50.035
2	15.262	3418730	83390	49.965
总计		6842304	186976	

〈峰表〉
检测器A 254nm

峰号	保留时间	面积	高度	浓度
1	11.323	5891201	176522	100.000
总计		5891201	176522	

(*S*)-N-((1-(quinolin-8-yl)naphthalen-2-yl)methyl)thietan-3-amine (**6h**)

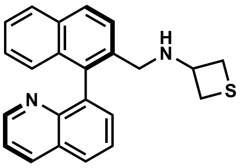

Yellow oil. 54% yield.

^{1}H NMR (400 MHz, CDCl$_3$): δ 8.85 (dd, J = 4.2, 2.0 Hz, 1H), 8.30 (dd, J = 8.4, 2.0 Hz, 1H), 8.02 – 7.92 (m, 2H), 7.89 (d, J = 8.2 Hz, 1H), 7.70 (td, J = 6.6, 3.4 Hz, 2H), 7.64 (dd, J = 7.0, 1.9 Hz, 1H), 7.53 – 7.36 (m, 2H), 7.29 – 7.19 (m, 1H), 7.15 (d, J = 8.5 Hz, 1H), 3.86 (p, J = 7.8 Hz, 1H), 3.43 (s, 2H), 2.97 – 2.85 (m, 1H), 2.79 (qd, J = 7.9, 3.6 Hz, 2H), 2.27 (td, J = 8.6, 1.7 Hz, 1H).

^{13}C NMR (101 MHz, CDCl$_3$): δ 150.8, 147.6, 138.5, 136.5, 136.2, 136.0, 133.4, 132.9, 131.9, 128.7, 128.2, 128.1, 127.2, 126.6, 126.3, 125.9, 125.3, 121.4, 82.8, 69.2, 51.8, 47.6, 19.5.

HRMS (ESI+): m/z calcd for $C_{23}H_{21}N_2S$ [M+H]$^+$: 357.1425, found 357.1420.

Enantioselectivity: >99% *ee*.

Chiral HPLC method: ODH column, 254 nm, 5% isopropanol/hexanes, flow rate 0.8 mL/min, room temperature, t_R (major) = 18.90 min, t_R (minor) = 20.20 min.

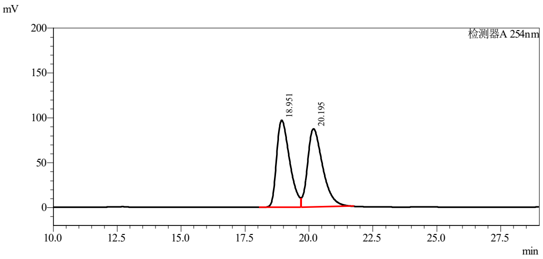

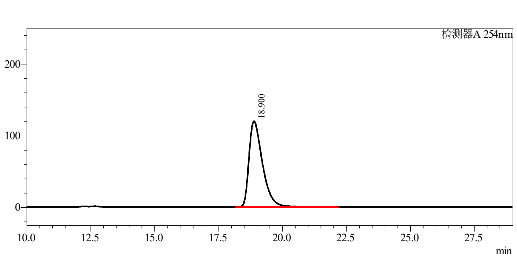

〈峰表〉
检测器A 254nm

峰号	保留时间	面积	高度	浓度
1	18.951	3437206	96886	49.157
2	20.195	3555089	86936	50.843
总计		6992295	183823	

〈峰表〉
检测器A 254nm

峰号	保留时间	面积	高度	浓度
1	18.900	4411164	119748	100.000
总计		4411164	119748	

(*S*)-3,3-difluoro-N-((1-(quinolin-8-yl)naphthalen-2-yl)methyl)cyclobutan-1-amine (**6i**)

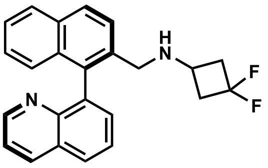

White solid. 66% yield.

^{1}H NMR (400 MHz, CDCl$_3$): δ 8.80 (dd, J = 4.2, 1.8 Hz, 1H), 8.30 (dd, J = 8.3, 1.9 Hz, 1H), 8.03 – 7.94 (m, 2H), 7.91 (d, J = 8.2 Hz, 1H), 7.71 (dt, J = 7.8, 3.3 Hz, 2H), 7.65 (dd, J = 7.1, 1.6 Hz, 1H), 7.48 – 7.39 (m, 2H), 7.31 – 7.21 (m, 1H), 7.16 (d, J = 8.5 Hz, 1H), 3.48 (d, J = 12.0 Hz, 1H), 3.44 (d, J = 12.0 Hz, 1H), 3.01 (hd, J = 6.9, 3.1 Hz, 1H), 2.43 (dtq, J = 32.3, 12.8, 6.0 Hz, 2H), 2.00 – 1.80 (m, 1H), 1.36 (tddd, J = 13.8, 9.5, 7.1, 2.7 Hz, 1H).

^{13}C NMR (126 MHz, CDCl$_3$): δ 150.8, 147.6, 138.3, 136.6, 136.4, 135.3, 133.4, 133.0, 132.0, 128.7, 128.4, 128.3, 128.1, 127.6, 126.6, 126.4, 126.0, 125.5, 121.6, 119.2 (dd, J = 283.9, 270.8 Hz), 50.4, 42.5 (dd, J = 23.2, 2.1 Hz), 42.5 (d, J = 81.8 Hz).42.3 – 42.0 (m).

^{19}F NMR (470 MHz, CDCl$_3$): δ -82.25 (ddd, J = 196.8, 19.0, 10.2 Hz), -96.60 (dp, J = 194.1, 14.6 Hz).

HRMS (ESI+): m/z calcd for $C_{24}H_{21}F_2N_2$ [M+H]$^+$: 375.1673, found 375.1665.

Enantioselectivity: >99% *ee*. Absolute configuration of the enzymatic product **6i** was assigned as *S* by X-ray crystallography.

Chiral HPLC method: ODH column, 254 nm, 10% isopropanol/hexanes, flow rate 0.8 mL/min, room temperature, t_R (major) = 9.69 min, t_R (minor) = 10.64 min.

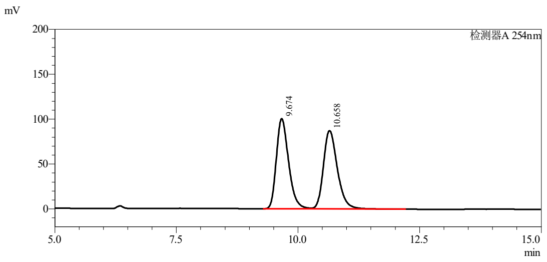

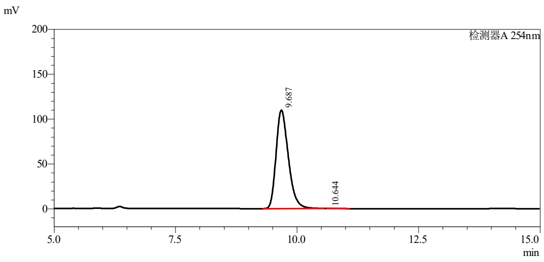

〈峰表〉

检测器A 254nm

峰号	保留时间	面积	高度	浓度
1	9.674	1764430	100428	50.220
2	10.658	1748970	87231	49.780
总计		3513400	187659	

〈峰表〉

检测器A 254nm

峰号	保留时间	面积	高度	浓度
1	9.687	1934057	109691	99.990
2	10.644	190	24	0.010
总计		1934247	109715	

General procedure for biocatalytic hydrogen borrowing

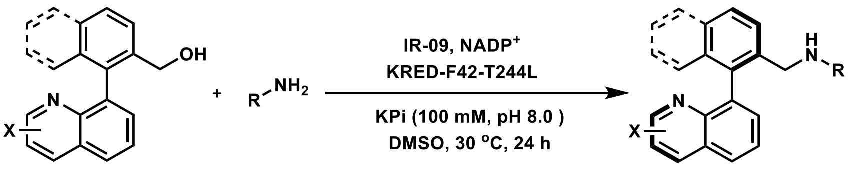

A screw vial (20 mL) was charged with $NADP^+$ (300 μL, 19 mg/mL stock solution in 100 mM KPi buffer pH 8.0), alcohols **11a-c** (500 μL, 100 mM stock in DMSO, 0.05 mmol, 1 equiv.) and amine **2a-c** (15 equiv.), purified IR-09 (2 mol%) and KRED-F42-T244L (0.8 mol%). KPi buffer (100 mM, pH 8.0) was added to bring the total volume to 10 mL. The vial was sealed and placed on a shaker at 250 rpm at 30 °C for 24 h. Upon completion, the reaction mixture was transferred to a 50 ml centrifuge tube, and added 10 ml EtOAc, and centrifuged at 4000 rpm for 5 minutes (×4). Collected organic phases were dried over Na_2SO_4, filtering and solvent was removed under vacuum. The residue was further purified by column chromatography on silica gel to afford the axially chiral biaryl amines **5a**, **5h**, **5j** and **6a-b**.

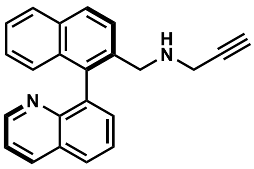

5a was isolated in 66% yield (average of three runs: 69%, 63%, 67%) with enantioselectivity (>99% ee).

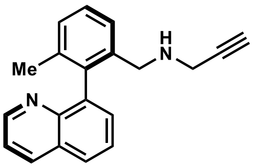

5h was isolated in 55% yield (average of three runs: 52%, 57%, 56%) with enantioselectivity (98% ee).

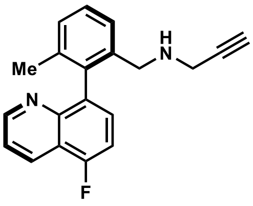

5j was isolated in 78% yield (average of three runs: 77%, 75%, 83%) with enantioselectivity (>99% ee).

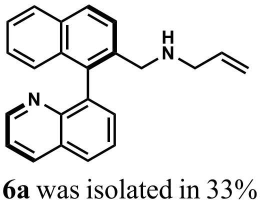

6a was isolated in 33% yield (average of three runs: 35%, 30%, 34%) with enantioselectivity (>99% ee).

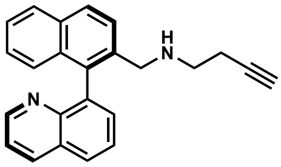

6b was isolated in 41% yield (average of three runs: 42%, 39%, 43%) with enantioselectivity (>99% ee).

Limitations

A Substrates scope

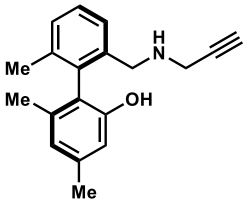

(*R*)-**3j**
22%, 78% *ee*

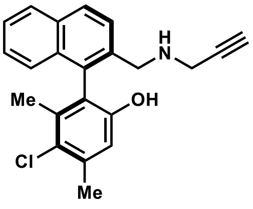

(*R*)-**3k**
6%, 83% *ee*

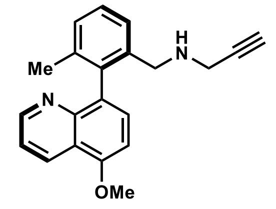

(*S*)-**5p**
26%, 89% *ee*

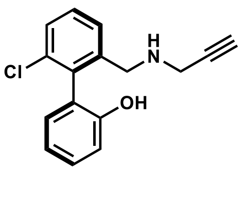

<5%[a], 57% *ee*[b]

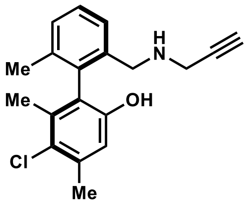

0%

B Amines scope

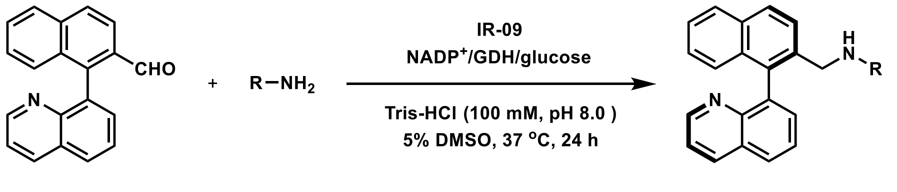

4a

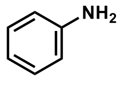

0%

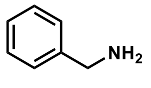

0%

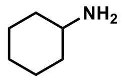

0%

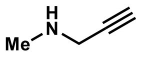

0%

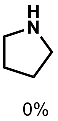

0%

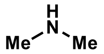

0%

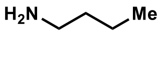

0%

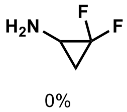

0%

SI Figure 8. (**A**) Biaryl aldehydes or (**B**) Amines not well accepted by IR-09. Reaction conditions: aldehyde (5 µmol, 1 equiv), amine (15 µmol, 3 equiv), $NADP^+$ (0.5 µmol, 10 mol%), glucose (15 µmol, 3 equiv), GDH (1 mg/mL), purified IR-09 (1 mol%) in Tris-HCl buffer (100 mM, pH 8.0) with 5% DMSO as cosolvent at 37 ºC for 24 h, the final total volume is 1 mL. [a] Yield was determined via LCMS. [b] Enantiomeric excess (*ee*) was determined by HPLC on a chiral stationary phase.

The *ee* of remaining substrates

A screw vial (4 mL) was charged with GDH (40 µL, 25 mg/mL stock solution in 100 mM Tris-HCl buffer pH 8.0), glucose (50 µL, 60 mg/mL stock solution in 100 mM Tris-HCl buffer pH 8.0), $NADP^+$ (10 µL, 38 mg/mL stock solution in 100 mM Tris-HCl buffer pH 8.0), IR-09 protein (0.2 mol%), aldehyde (50 µL, 100 mM stock in DMSO, 0.05 mmol, 1 equiv.) and amine (8 equiv.). Tris-HCl buffer (100 mM, pH 8.0) was added to bring the total volume to 1 mL. The vial was sealed and placed on a shaker at 250 rpm at 37 °C for 24 h. Upon completion, transfer the reaction mixture to a 2 ml centrifuge tube, add 500 µL EtOAc, and centrifuge at 12000 rpm for 5 minutes. Collected organic phases were dried over Na_2SO_4, filtering and solvent was removed under vacuum. The crudes were purified and then dissolved in isopropanol for chiral HPLC analysis.

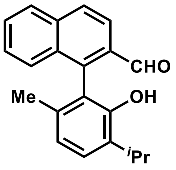

The recovered material **1c** had an enantiomeric excess (*ee*) of 11%.
Chiral HPLC method: ADH column, 254 nm, 5% isopropanol/hexanes, flow rate 0.6 mL/min, room temperature.

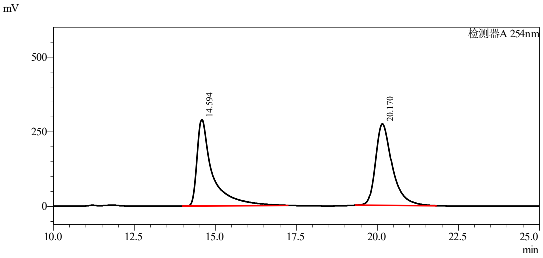

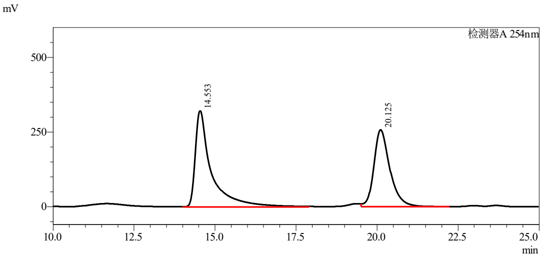

〈峰表〉
检测器A 254nm

峰号	保留时间	面积	高度	浓度
1	14.594	9617157	289522	49.328
2	20.170	9879277	273040	50.672
总计		19496434	562562	

〈峰表〉
检测器A 254nm

峰号	保留时间	面积	高度	浓度
1	14.553	11170306	321676	55.481
2	20.125	8963157	257625	44.519
总计		20133463	579302	

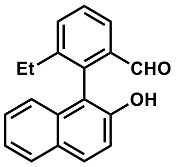

The recovered material **1e** had an enantiomeric excess (*ee*) of 21%.
Chiral HPLC method: ADH column, 254 nm, 10% isopropanol/hexanes, flow rate 0.8 mL/min, room temperature.

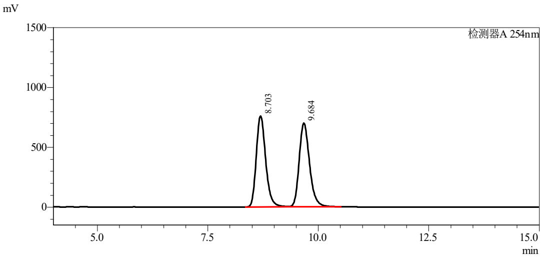

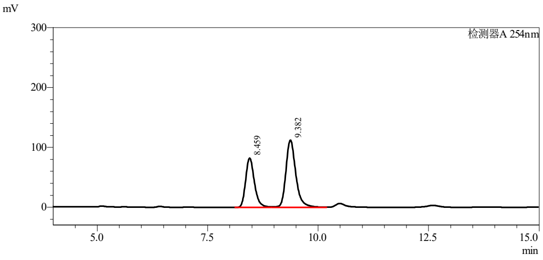

〈峰表〉

检测器A 254nm

峰号	保留时间	面积	高度	浓度
1	8. 703	11383373	761241	49. 808
2	9. 684	11471127	699897	50. 192
总计		22854500	1461138	

〈峰表〉

检测器A 254nm

峰号	保留时间	面积	高度	浓度
1	8. 459	1143235	82357	39. 310
2	9. 382	1765004	112475	60. 690
总计		2908239	194832	

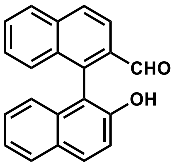

The recovered material **1f** had an enantiomeric excess (*ee*) of 37%.

Chiral HPLC method: ADH column, 254 nm, 5% isopropanol/hexanes, flow rate 0.8 mL/min, room temperature.

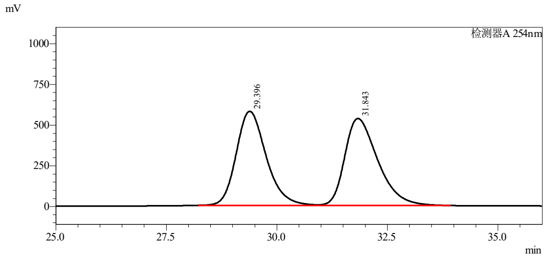

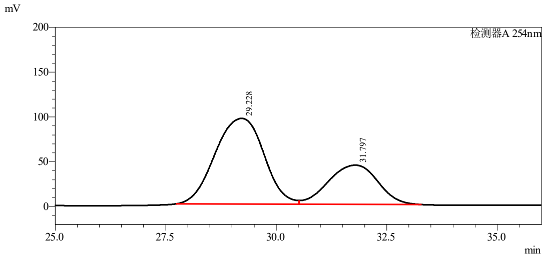

〈峰表〉

检测器A 254nm

峰号	保留时间	面积	高度	浓度
1	29. 396	27322902	577955	49. 530
2	31. 843	27841024	534013	50. 470
总计		55163926	1111968	

〈峰表〉

检测器A 254nm

峰号	保留时间	面积	高度	浓度
1	29. 228	7346909	95615	68. 337
2	31. 797	3404029	43772	31. 663
总计		10750938	139387	

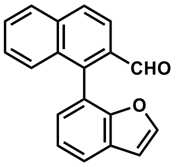

The recovered material **4n** had an enantiomeric excess (*ee*) of 0%.

Chiral HPLC method: ODH column, 254 nm, 10% isopropanol/hexanes, flow rate 0.8 mL/min, room temperature.

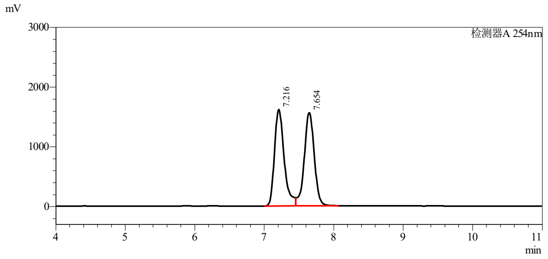

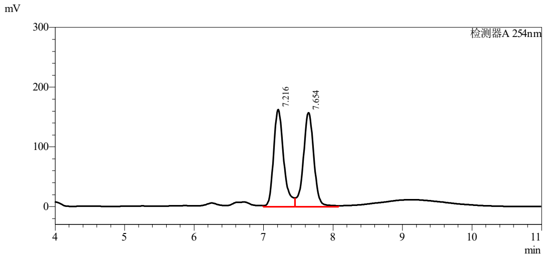

〈峰表〉

检测器A 254nm

峰号	保留时间	面积	高度	浓度
1	7. 216	15846207	1617628	49. 997
2	7. 654	15848072	1568953	50. 003
总计		31694280	3186580	

〈峰表〉

检测器A 254nm

峰号	保留时间	面积	高度	浓度
1	7. 216	1661259	162904	50. 065
2	7. 654	1656951	157503	49. 935
总计		3318210	320407	

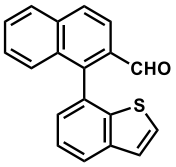

The recovered material **4o** had an enantiomeric excess (*ee*) of 53%.

Chiral HPLC method: ADH column, 254 nm, 5% isopropanol/hexanes, flow rate 0.8 mL/min, room temperature.

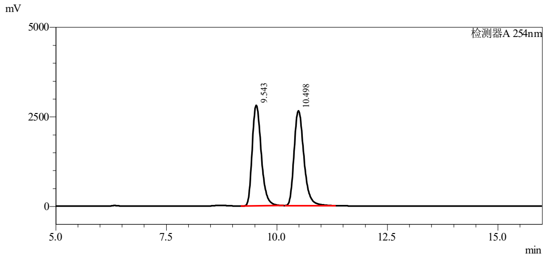

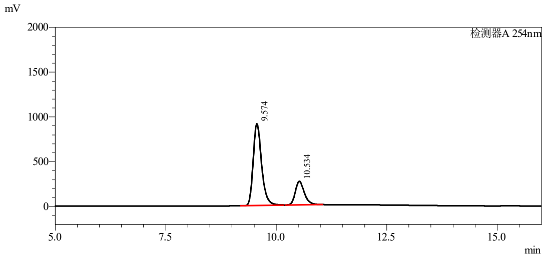

〈峰表〉
检测器A 254nm

峰号	保留时间	面积	高度	浓度
1	9.543	40266602	2808398	49.439
2	10.498	41180942	2645833	50.561
总计		81447544	5454232	

〈峰表〉
检测器A 254nm

峰号	保留时间	面积	高度	浓度
1	9.574	12106868	911811	76.394
2	10.534	3741052	263070	23.606
总计		15847920	1174881	

Derivatization and application of enzymatic products

Procedure for the Synthesis of **7a**

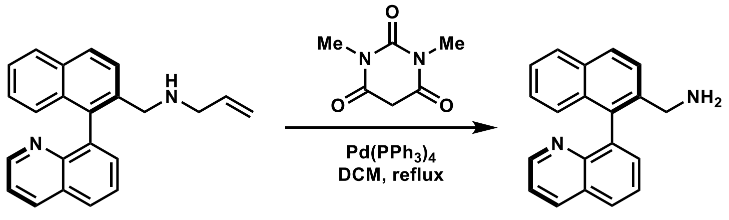

(S)-**6a** >99% *ee*   (S)-**7a** >99% *ee*

Substrates **7a** were synthesized according to the reported procedure[7]: To a stirred solution of (*S*)-**6a** (140 mg, 0.43 mmol, 1equiv.) in anhydrous DCM (5 mL), 1,3-dimethylbarbituric acid (672 mg, 4.3 mmol, 10 equiv.) and $Pd(PPh_3)_4$ (25 mg, 0.0215 mmol, 0.05 equiv.) were added and the reaction mixture was refluxed for 12 h. After completion, 10 mL of 2M NaOH aqueous solution were added and the mixture was extracted with EtOAc (3×10 mL). The combined organic phases were dried over Na_2SO_4, filtered and removed in vacuo. The residue was further purified by column chromatography on silica gel (DCM: MeOH = 10:1) to afford the (*S*)-**7a**.

Procedure for the Synthesis of **8a**

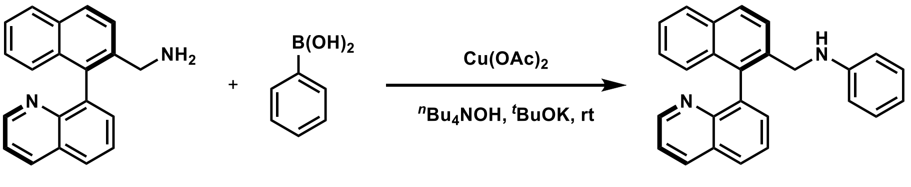

(S)-**7a** >99% *ee*   (S)-**8a** >99% *ee*

Substrates **8a** were synthesized according to the reported procedure[8]: To a stirred solution of the amine (*S*)-**7a** (28.4 mg, 0.1 mmol, 1equiv.) in 20% aqueous solution of *n*-Bu_4NOH, $Cu(OAC)_2$ (3.6 mg, 0.02 mmol, 0.2 equiv.), *t*-BuOK (22 mg, 0.2 mmol, 2equiv.) and phenylboronic acid (18 mg, 0.15 mmol, 1.5equiv.) were added. The reaction mixture was stirred at rt for 24 h. After completion, the mixture was extracted with EtOAc. The combined organic phases were dried over Na_2SO_4, filtered and removed in vacuo. The residue was further purified by column chromatography on silica gel (PE: EtOAc = 10:1) to afford the (*S*)-**8a**.

Procedure for the Synthesis of **8b-c**

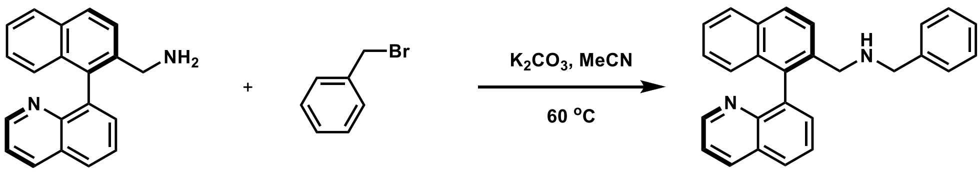

(*S*)-**7a** >99% *ee* (*S*)-**8b** >99% *ee*

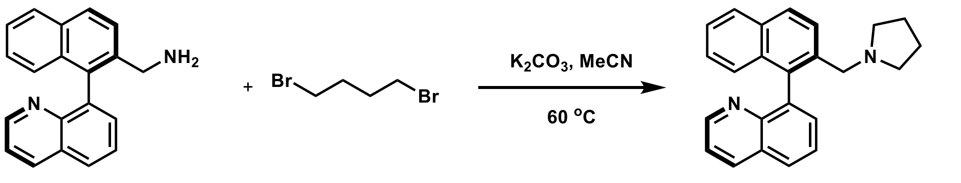

(*S*)-**7a** >99% *ee* (*S*)-**8c** >99% *ee*

To a stirred solution of the amine (*S*)-**7a** (20mg, 0.07mmol, 1equiv.) in anhydrous MeCN (1 mL), corresponding bromide (1.1 equiv.) and K_2CO_3 (20 mg, 0.14 mmol, 2 equiv.) were added and the reaction mixture was stirred overnight at 60 ºC. After completion, the reaction mixture was diluted with EtOAc and brine, and extracted with EtOAc. The combined organic phases were dried over Na_2SO_4, filtered and removed in vacuo. The residue was further purified by column chromatography on silica gel to afford the (*S*)-**8b-c**.

Procedure for the Synthesis of **8d**

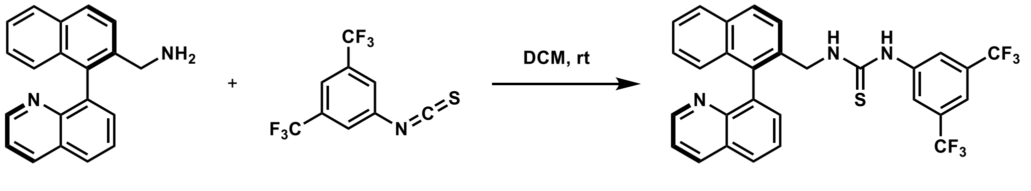

(*S*)-**7a** >99% *ee* (*S*)-**8d** >99% *ee*

Substrates **8d** were synthesized according to the reported procedure[7]: To a stirred solution of the amine (*S*)-**7a** (60 mg, 0.21 mmol, 1 equiv.) in anhydrous DCM, 1-isothiocyanato-3,5 bis(trifluoromethyl)benzenewas (65 mg, 0.25 mmol, 1.2 equiv.) was added and the reaction mixture was stirred at rt for 12 h. After completion, the solvent was removed removed in vacuo and the residue was further purified by column chromatography on silica gel to afford (*S*)-**8d**.

Procedure for the Synthesis of **8e-f**

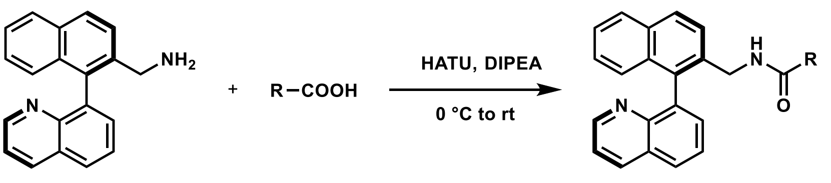

(*S*)-**7a** >99% *ee* (*S*)-**8e-f** >99% *ee*

To a stirred solution of the corresponding acid (1.2 equiv.) in anhydrous DCM, HATU (46 mg, 0.12 mmol, 1.2 equiv.) and DIPEA (31 mg, 0.24 mmol, 2 equiv.) were added at 0 ºC. The reaction mixture was stirred at 0 ºC for 30 min before the addition of the (*S*)-**7a** (28.4 mg, 0.1 mmol, 1 equiv.) and DIPEA (31 mg, 0.24 mmol, 2 equiv.). The reaction mixture was stirred at rt for 12 h. After

completion, the reaction mixture was diluted with DCM and brine, and extracted with DCM. The combined organic phases were dried over Na_2SO_4, filtered and removed in vacuo. The residue was further purified by column chromatography on silica gel to afford the (*S*)-**8e-f**.

Procedure for the Synthesis of **9**

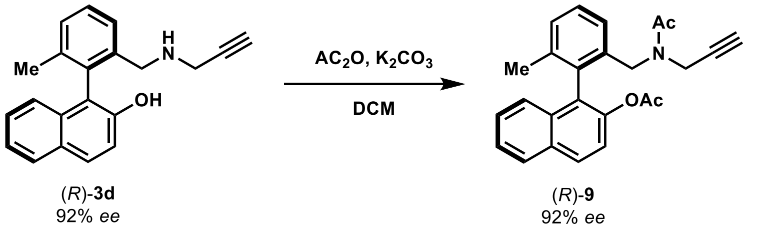

Substrates **9** were synthesized according to the reported procedure[9]: To a stirred solution of the (*R*)-**3d** (43.3 mg, 0.14 mmol, 1 equiv.) in anhydrous DCM, acetic anhydride (72 mg, 0.7 mmol, 5 equiv.) and K_2CO_3 (193 mg, 1.4 mmol, 10 equiv.) were added and the reaction mixture was refluxed for 12 h. After completion, the reaction mixture was diluted with EtOAc and brine, and extracted with EtOAc. The combined organic phases were dried over Na_2SO_4, filtered and removed in vacuo. The residue was further purified by column chromatography on silica gel to afford the (*R*)-**9**.

Procedure for the Synthesis of **10**

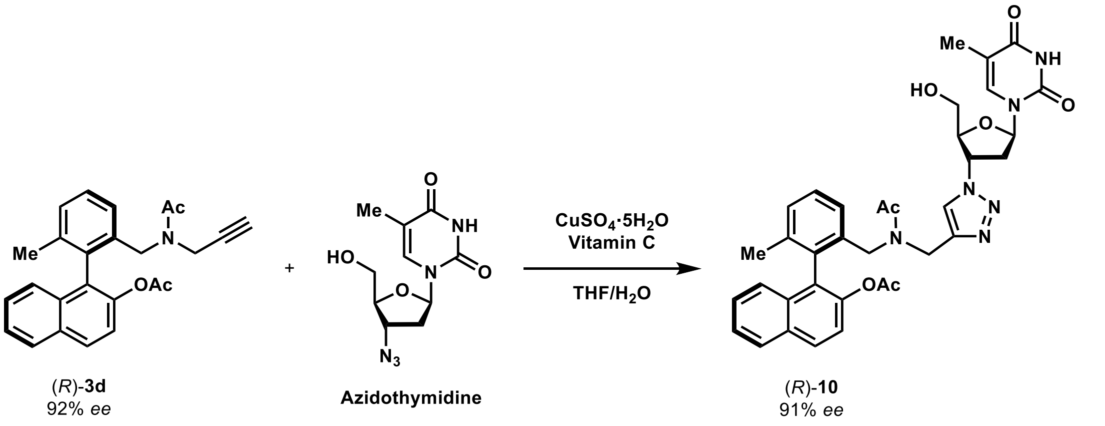

Substrates **10** were synthesized according to the reported procedure[10]: To a stirred solution of the (*R*)-**3d** (26 mg, 0. 067 mmol, 1 equiv.) in THF/H_2O (3:1 *v/v*) were added azidothymidine (18 mg, 0.067 mmol, 1 equiv.), 1 M solution of vitamin C (0.1 equiv.) in water and 1 M solution of $CuSO_4·5H_2O$ (0.06 equiv.) in water. The reaction mixture was stirred at rt for 12 h. After completion, the reaction mixture was diluted with EtOAc and brine, and extracted with EtOAc. The combined organic phases were dried over Na_2SO_4, filtered and removed in vacuo. The residue was further purified by column chromatography on silica gel to afford the (*R*)-**10**.

(*S*)-(1-(quinolin-8-yl)naphthalen-2-yl)methanamine (**7a**)

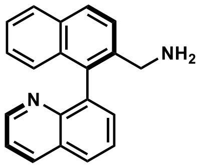

Yellow solid. 88% yield.

^{1}H NMR (400 MHz, CDCl$_3$): δ 8.75 (dd, J = 4.2, 1.8 Hz, 1H), 8.27 (dd, J = 8.3, 1.8 Hz, 1H), 8.00 – 7.91 (m, 2H), 7.88 (d, J = 8.2 Hz, 1H), 7.67 (d, J = 8.0 Hz, 2H), 7.62 (dd, J = 7.0, 1.6 Hz, 1H), 7.40 (ddd, J = 12.5, 7.6, 2.7 Hz, 2H), 7.28 – 7.19 (m, 1H), 7.12 (d, J = 8.5 Hz, 1H), 3.60 (q, J = 14.1 Hz, 2H), 2.93 (s, 2H).

^{13}C NMR (101 MHz, CDCl$_3$): δ 150.9, 147.5, 138.1, 137.8, 136.7, 135.5, 133.4, 132.9, 132.2, 128.8, 128.3, 128.1, 126.6, 126.5, 126.4, 126.1, 125.5, 121.5, 44.9.

HRMS (ESI+): m/z calcd for $C_{20}H_{17}N_2$ [M+H]$^+$: 285.1392, found 285.1383 .

The enantiomeric excess (*ee*) was not determined due to the compound's high polarity.

(*S*)-N-((1-(quinolin-8-yl)naphthalen-2-yl)methyl)aniline (**8a**)

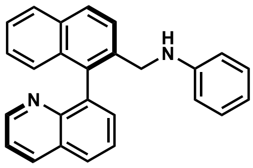

Yellow solid. 28% yield.

^{1}H NMR (400 MHz, CDCl$_3$): δ 8.86 (dd, J = 4.0, 1.8 Hz, 1H), 8.24 (dd, J = 8.3, 1.8 Hz, 1H), 7.94 (td, J = 9.4, 7.0 Hz, 3H), 7.75 (d, J = 8.5 Hz, 1H), 7.68 (d, J = 4.8 Hz, 2H), 7.47 – 7.35 (m, 2H), 7.31 – 7.22 (m, 1H), 7.18 (d, J = 8.5 Hz, 1H), 7.06 (t, J = 7.7 Hz, 2H), 6.61 (t, J = 7.3 Hz, 1H), 6.43 (d, J = 7.9 Hz, 2H), 4.14 (d, J = 13.4 Hz, 1H), 3.98 (d, J = 13.3 Hz, 1H).

^{13}C NMR (101 MHz, CDCl$_3$): δ 150.7, 148.4, 147.5, 138.1, 136.6, 136.2, 135.2, 133.5, 133.1, 131.7, 129.1, 128.8, 128.4, 128.3, 128.2, 126.6, 126.5, 126.4, 126.0, 125.5, 121.4, 117.1, 112.7, 47.4.

HRMS (ESI+): m/z calcd for $C_{26}H_{21}N_2$ [M+H]$^+$: 361.1705, found 361.1706 .

Enantioselectivity: >99% *ee*.

Chiral HPLC method: ODH column, 254 nm, 5% isopropanol/hexanes, flow rate 0.8 mL/min, room temperature, t_R (major) = 18.17 min, t_R (minor) = 23.98 min.

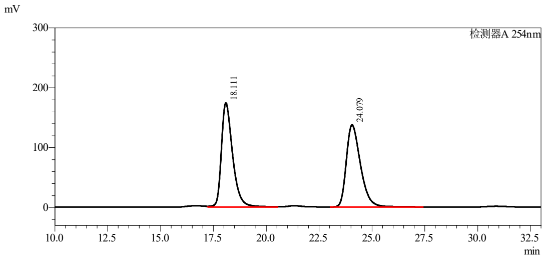

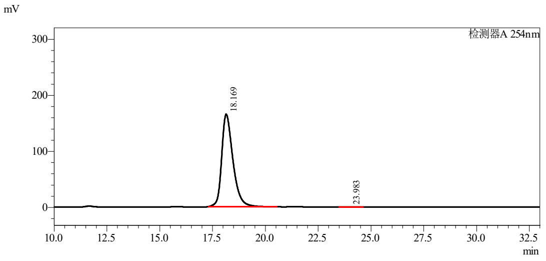

〈峰表〉
检测器A 254nm

峰号	保留时间	面积	高度	浓度
1	18.111	6455078	174044	50.275
2	24.079	6384377	137210	49.725
总计		12839455	311254	

〈峰表〉
检测器A 254nm

峰号	保留时间	面积	高度	浓度
1	18.169	6126625	164991	99.936
2	23.983	3911	103	0.064
总计		6130537	165094	

(S)-N-benzyl-1-(1-(quinolin-8-yl)naphthalen-2-yl)methanamine (**8b**)

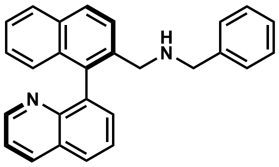

Yellow oil. 44% yield.

^{1}H NMR (400 MHz, CDCl$_3$): δ 8.71 (dd, J = 4.2, 1.8 Hz, 1H), 8.26 (dd, J = 8.3, 1.8 Hz, 1H), 7.95 (dt, J = 19.2, 8.4 Hz, 3H), 7.82 (d, J = 8.5 Hz, 1H), 7.70 – 7.59 (m, 2H), 7.46 – 7.34 (m, 2H), 7.28 – 7.09 (m, 5H), 7.03 (dd, J = 7.0, 2.4 Hz, 2H), 3.62 (d, J = 13.3 Hz, 1H), 3.62 (s, 2H), 3.52 (d, J = 13.3 Hz, 1H).

^{13}C NMR (101 MHz, CDCl$_3$): δ 150.7, 147.6, 139.8, 138.3, 136.5, 136.4, 135.7, 133.5, 133.0, 131.9, 128.6, 128.3, 128.2, 128.2, 128.1, 128.0, 127.6, 126.8, 126.6, 126.3, 125.9, 125.4, 121.3, 53.2, 51.9.

HRMS (ESI+): m/z calcd for $C_{27}H_{23}N_2$ [M+H]$^+$: 375.1861, found 375.1857.

Enantioselectivity: >99% *ee*.

Chiral HPLC method: ODH column, 254 nm, 5% isopropanol/hexanes, flow rate 0.8 mL/min, room temperature, t_R (major) = 21.04 min, t_R (minor) = 22.76 min.

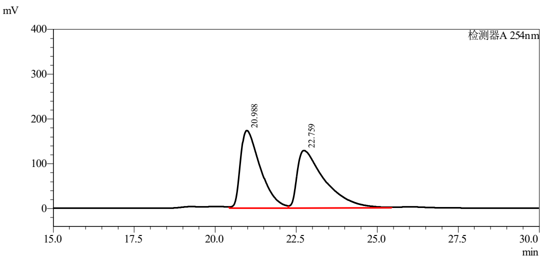

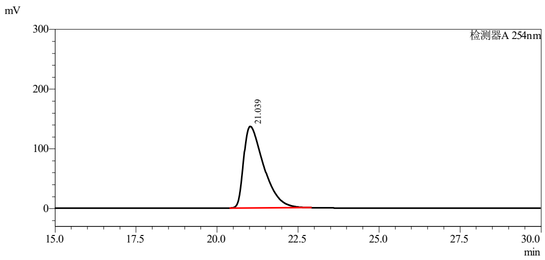

〈峰表〉
检测器A 254nm

峰号	保留时间	面积	高度	浓度
1	20.988	7464114	173392	49.289
2	22.759	7679560	128563	50.711
总计		15143675	301955	

〈峰表〉
检测器A 254nm

峰号	保留时间	面积	高度	浓度
1	21.039	6032176	137043	100.000
总计		6032176	137043	

(S)-8-(2-(pyrrolidin-1-ylmethyl)naphthalen-1-yl)quinoline (**8c**)

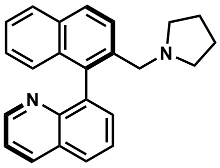

Yellow solid. 51% yield.

^{1}H NMR (400 MHz, CDCl$_3$): δ 8.79 (dd, J = 4.1, 1.8 Hz, 1H), 8.26 (dd, J = 8.3, 1.8 Hz, 1H), 7.99 – 7.93 (m, 3H), 7.89 (d, J = 8.1 Hz, 1H), 7.68 (t, J = 7.5 Hz, 1H), 7.62 (dd, J = 7.0, 1.6 Hz, 1H), 7.44 – 7.35 (m, 2H), 7.21 (ddd, J = 8.2, 6.7, 1.3 Hz, 1H), 7.12 (d, J = 8.5 Hz, 1H), 3.52 (d, J = 13.6 Hz, 1H), 3.39 (d, J = 13.7 Hz, 1H), 2.41 (s, 4H), 1.66 (d, J = 6.3 Hz, 4H).

^{13}C NMR (101 MHz, CDCl$_3$): δ 150.6, 147.6, 138.5, 136.3, 136.0, 133.3, 132.8, 132.1, 128.5, 128.1, 128.1, 128.0, 127.0, 126.7, 126.2, 125.8, 125.3, 121.2, 57.8, 54.1, 23.6.

HRMS (ESI+): m/z calcd for $C_{23}H_{24}N_2$ [M+H]$^+$: 339.1861, found 339.1858.

Enantioselectivity: >99% *ee*.

Chiral HPLC method: ODH column, 254 nm, 5% isopropanol/hexanes, flow rate 0.8 mL/min, room temperature, t_R (major) = 7.93 min, t_R (minor) = 6.85 min.

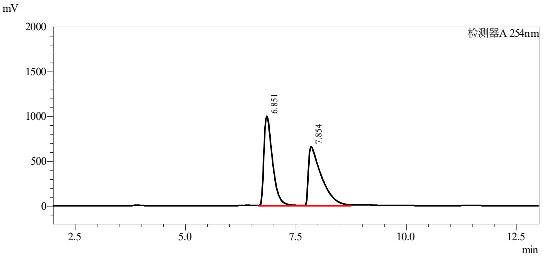

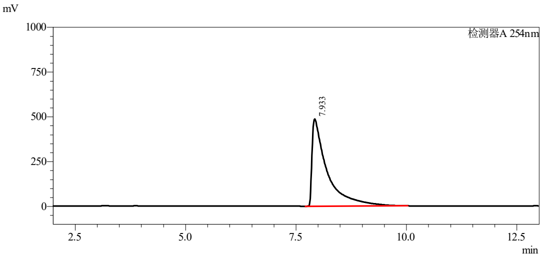

〈峰表〉
检测器A 254nm

峰号	保留时间	面积	高度	浓度
1	6.851	13634912	999940	49.955
2	7.854	13659618	658998	50.045
总计		27294531	1658938	

〈峰表〉
检测器A 254nm

峰号	保留时间	面积	高度	浓度
1	7.933	11757175	487387	100.000
总计		11757175	487387	

(*S*)-1-(3,5-bis(trifluoromethyl)phenyl)-3-((1-(quinolin-8-yl)naphthalen-2-yl)methyl)thiourea (**8d**)

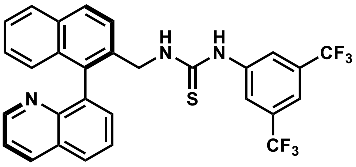

White solid. 72% yield.

^{1}H NMR (500 MHz, DMSO-*d$_6$*): δ 9.28 (s, 1H), 8.72 (dd, *J* = 4.1, 1.8 Hz, 1H), 8.48 (dd, *J* = 8.3, 1.8 Hz, 1H), 8.14 (dd, *J* = 8.2, 1.5 Hz, 1H), 8.05 – 7.93 (m, 4H), 7.79 (dd, *J* = 8.2, 7.0 Hz, 1H), 7.71 (dd, *J* = 7.0, 1.5 Hz, 1H), 7.66 (d, *J* = 8.6 Hz, 1H), 7.56 – 7.49 (m, 2H), 7.44 (ddd, *J* = 8.1, 6.7, 1.2 Hz, 1H), 7.26 (ddd, *J* = 8.3, 6.7, 1.3 Hz, 1H), 7.00 (dd, *J* = 8.5, 1.1 Hz, 1H), 6.70 (t, *J* = 5.7 Hz, 1H), 4.08 (dd, *J* = 15.2, 5.8 Hz, 1H), 3.99 (dd, *J* = 15.2, 5.7 Hz, 1H).

^{13}C NMR (126 MHz, DMSO-*d$_6$*): δ 151.0, 146.9, 143.0, 138.8, 133.3, 133.0, 132.0, 131.4, 129.2, 128.6, 127.8, 127.2 (d, *J* = 32.4 Hz), 126.7 (d, *J* = 32.4 Hz), 124.9, 124.6, 124.2, 123.9, 122.8, 122.4 (d, *J* = 9.8 Hz), 121.9 (d, *J* = 272.7 Hz), 121.7 (d, *J* = 9.2 Hz), 117.9, 117.6 (d, *J* = 272.6 Hz), 113.6 (d, *J* = 3.0 Hz), 110.9 – 109.3 (m), 37.9.

^{19}F NMR (470 MHz, DMSO-*d$_6$*): δ -61.65.

HRMS (ESI+): m/z calcd for $C_{29}H_{20}F_6N_3S$ $[M+H]^+$: 556.1282, found 556.1482.

Enantioselectivity: >99% *ee*.

Chiral HPLC method: IA column, 254 nm, 5% isopropanol/hexanes, flow rate 0.5 mL/min, room temperature, t_R (major) = 18.19 min, t_R (minor) = 21.45 min.

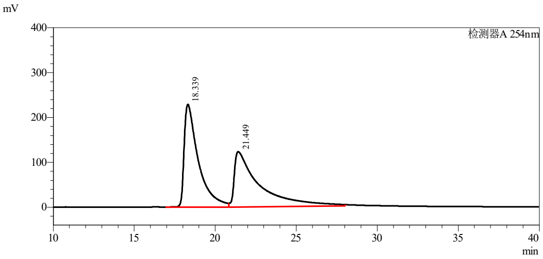

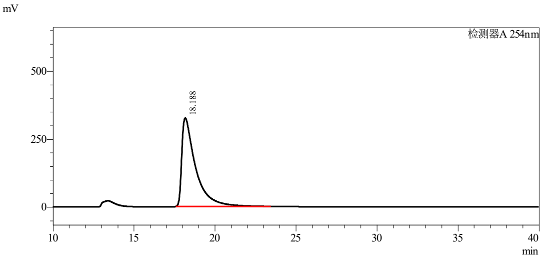

〈峰表〉
检测器A 254nm

峰号	保留时间	面积	高度	浓度
1	18.339	13953915	228756	48.339
2	21.449	14913147	123245	51.661
总计		28867062	352001	

〈峰表〉
检测器A 254nm

峰号	保留时间	面积	高度	浓度
1	18.188	19961146	327035	100.000
总计		19961146	327035	

(S)-2-(((1-(quinolin-8-yl)naphthalen-2-yl)methyl)carbamoyl)phenyl acetate **(8e)**

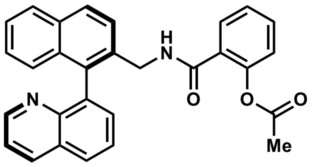

White solid. 52% yield.

^{1}H NMR (400 MHz, CDCl$_3$): δ 8.70 (dd, J = 4.1, 1.9 Hz, 1H), 8.25 (dd, J = 8.3, 1.9 Hz, 1H), 7.98 (dd, J = 7.6, 2.7 Hz, 2H), 7.91 (d, J = 8.2 Hz, 1H), 7.75 – 7.63 (m, 4H), 7.48 – 7.38 (m, 2H), 7.34 (dd, J = 8.3, 4.2 Hz, 1H), 7.28 – 7.21 (m, 3H), 7.16 (d, J = 8.5 Hz, 1H), 7.05 (d, J = 8.1 Hz, 1H), 4.94 (dd, J = 13.7, 8.2 Hz, 1H), 3.88 (dd, J = 13.7, 2.6 Hz, 1H), 1.80 (s, 3H).

^{13}C NMR (101 MHz, CDCl$_3$): δ 169.3, 164.6, 150.7, 148.0, 147.5, 137.6, 136.9, 136.5, 134.3, 133.4, 133.2, 132.5, 131.8, 130.4, 128.9, 128.8, 128.5, 128.2, 128.1, 127.4, 126.5, 126.3, 125.8, 123.2, 121.6, 42.8, 20.4.

HRMS (ESI+): m/z calcd for $C_{29}H_{23}N_2O_3$ $[M+H]^+$: 447.1709, found 447.1718.

Enantioselectivity: >99% *ee*.

Chiral HPLC method: ODH column, 254 nm, 10% isopropanol/hexanes, flow rate 0.8 mL/min, room temperature, t_R (major) = 29.17 min, t_R (minor) = 33.04 min.

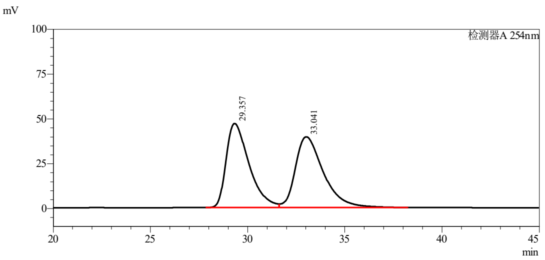

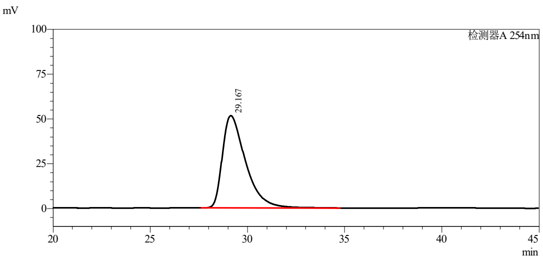

<峰表>
检测器A 254nm

峰号	保留时间	面积	高度	浓度
1	29.357	3864319	46904	49.315
2	33.041	3971731	39430	50.685
总计		7836049	86334	

<峰表>
检测器A 254nm

峰号	保留时间	面积	高度	浓度
1	29.167	4290472	51493	100.000
总计		4290472	51493	

(S)-2-(6-methoxynaphthalen-2-yl)-N-(((S)-1-(quinolin-8-yl)naphthalen-2-yl)methyl)propanamide (**8f**)

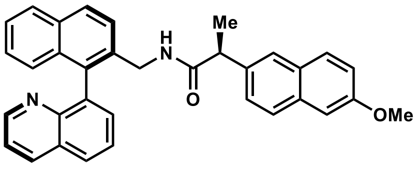

Colorless oil. 56% yield.

^{1}H NMR (400 MHz, CDCl$_3$): δ 8.35 (dd, J = 4.1, 1.9 Hz, 1H), 8.27 – 8.16 (m, 1H), 7.88 (ddd, J = 13.3, 7.5, 3.7 Hz, 3H), 7.66 (dd, J = 8.6, 6.2 Hz, 2H), 7.52 (t, J = 5.5 Hz, 4H), 7.40 (t, J = 7.5 Hz, 1H), 7.25 – 7.04 (m, 6H), 5.96 (s, 1H), 4.48 (dd, J = 14.0, 6.8 Hz, 1H), 3.93 (s, 3H), 3.92 – 3.86 (m, 1H), 3.41 (q, J = 7.2 Hz, 1H), 1.44 (d, J = 7.1 Hz, 3H).

^{13}C NMR (101 MHz, CDCl$_3$): δ 173.8, 157.8, 150.6, 147.3, 137.6, 136.7, 136.4, 136.4, 134.1, 133.8, 133.2, 133.1, 132.1, 129.4, 129.1, 128.6, 128.3, 128.1, 127.5, 127.1, 126.5, 126.5, 126.4, 126.1, 126.1, 125.7, 121.3, 119.1, 105.7, 55.4, 47.0, 42.9, 18.5.

HRMS (ESI+): m/z calcd for $C_{34}H_{29}N_2O_2$ $[M+H]^+$: 497.2229, found 497.2238.

Enantioselectivity: >20:1 *dr*, >99% *ee*.

Chiral HPLC method: ODH column, 254 nm, 10% isopropanol/hexanes, flow rate 0.8 mL/min, room temperature, t_R (major) = 20.89 min, t_R (minor) = 18.09 min.

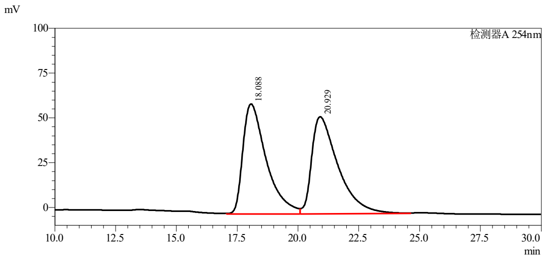

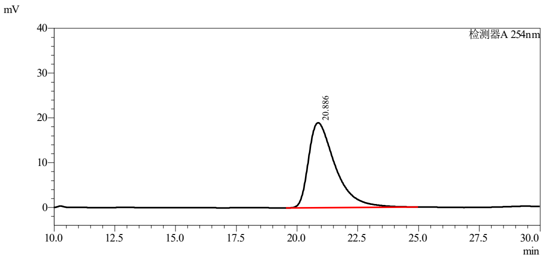

〈峰表〉
检测器A 254nm

峰号	保留时间	面积	高度	浓度
1	18.088	4125116	61399	50.050
2	20.929	4116889	54199	49.950
总计		8242005	115599	

〈峰表〉
检测器A 254nm

峰号	保留时间	面积	高度	浓度
1	20.886	1456209	18979	100.000
总计		1456209	18979	

(*R*)-1-(2-methyl-6-((N-(prop-2-yn-1-yl)acetamido)methyl)phenyl)naphthalen-2-yl acetate (**9**)

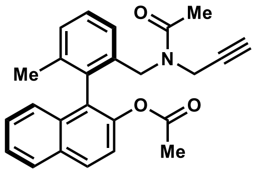

Colorless oil. 85% yield.

^{1}H NMR (400 MHz, CDCl$_3$): δ 7.94 (t, J = 8.1 Hz, 2H), 7.91 – 7.86 (m, 0.55H*), 7.48 (dt, J = 15.1, 7.5 Hz, 1.31H*), 7.43 – 7.30 (m, 4H), 7.29 – 7.19 (m, 3H), 7.14 (d, J = 7.6 Hz, 0.91H*), 4.50 (d, J = 15.4 Hz, 0.32H*), 4.30 (d, J = 17.5 Hz, 1H), 4.19 (dd, J = 17.2, 2.5 Hz, 1H), 4.12 (d, J = 16.4 Hz, 1H), 4.07 – 3.97 (m, 1H), 3.67 – 3.50 (m, 0.66H), 2.03 (d, J = 2.4 Hz, 0.42H*), 1.99 – 1.96 (m, 1H), 1.95 (s, 1H*)，1.94 (s, 3H) 1.92 (s, 3H), 1.90 (s, 1.22H*), 1.82 (s, 1.17H*), 1.76 (s, 3H). *Indicates the peaks of rotamer (3:1).

^{13}C NMR (101 MHz, CDCl$_3$): δ 170.9, 169.3, 145.8, 138.6, 135.2, 133.0, 132.1, 132.0, 129.6, 129.4, 128.5, 128.5, 127.3, 126.1, 124.6, 123.5, 121.9, 78.8, 71.6, 49.6, 34.4, 21.0, 20.5, 19.7.

HRMS (ESI+): m/z calcd for $C_{25}H_{24}NO_3$ $[M+H]^+$: 386.1756, found 386.1761.

Enantioselectivity: 92% *ee*.

Chiral HPLC method: ODH column, 254 nm, 10% isopropanol/hexanes, flow rate 0.8 mL/min, room temperature, t_R (major) = 12.41 min, t_R (minor) = 14.02 min.

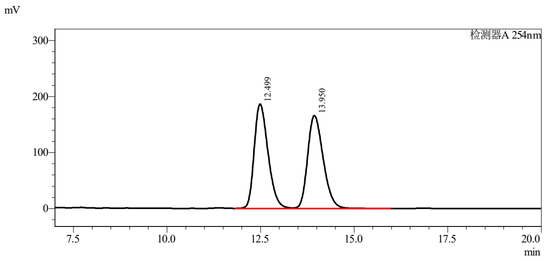

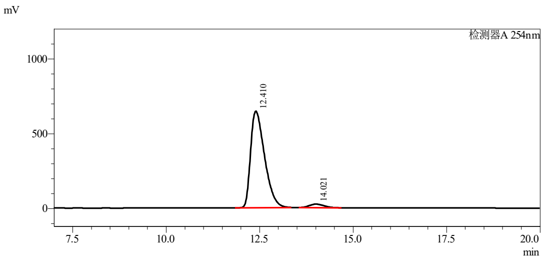

〈峰表〉
检测器A 254nm

峰号	保留时间	面积	高度	浓度
1	12.499	4811959	186181	50.010
2	13.950	4810042	165745	49.990
总计		9622001	351927	

〈峰表〉
检测器A 254nm

峰号	保留时间	面积	高度	浓度
1	12.410	16813246	645360	96.283
2	14.021	649091	23674	3.717
总计		17462337	669034	

(R)-1-(2-((N-((1-((2S,3S,5R)-2-(hydroxymethyl)-5-(5-methyl-2,4-dioxo-3,4-dihydropyrimidin-1(2H)-yl)tetrahydrofuran-3-yl)-1H-1,2,3-triazol-4-yl)methyl)acetamido)methyl)-6-methylphenyl)naphthalen-2-yl acetate (**10**)

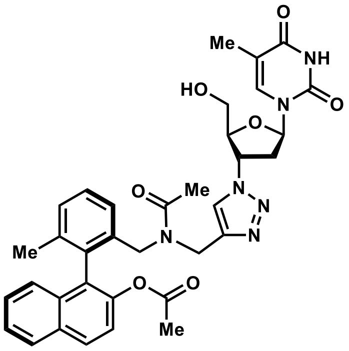

White solid. 81% yield.

^{1}H NMR (500 MHz, CDCl$_3$): δ 10.09 (d, J = 4.4 Hz, 1H), 9.78 – 9.74 (m, 0.22H*), 7.91 – 7.77 (m, 3H), 7.70 – 7.60 (m, 1H), 7.47 (s, 0.22H*), 7.43 (dtd, J = 8.0, 4.0, 1.9 Hz, 1H), 7.37 (dddd, J = 8.2, 6.9, 2.9, 1.3 Hz, 1H), 7.34 – 7.29 (m, 1H), 7.29 – 7.24 (m, 2H), 7.21 (d, J = 8.5 Hz, 2H), 7.06 (d, J = 7.6 Hz, 1H), 6.64 (d, J = 14.1 Hz, 0.22H*), 6.21 (t, J = 5.9 Hz, 1H), 6.13 (td, J = 6.5, 2.4 Hz, 0.22H*), 5.26 – 5.17 (m, 1H), 5.15 (ddt, J = 8.5, 5.7, 2.8 Hz, 0.21H*), 4.53 – 4.36 (m, 2H), 4.33 – 4.05 (m, 3H), 4.05 – 3.80 (m, 2H), 3.70 – 3.60 (m, 1H), 2.92 (dt, J = 13.7, 6.8 Hz, 1H), 2.68 (dtd, J = 14.0, 6.6, 4.2 Hz, 1H), 2.74 (dtd, J = 8.6, 5.7, 2.9 Hz, 0.25H*), 2.07 (d, J = 6.2 Hz, 0.67H*), 1.91 (d, J = 2.0 Hz, 3H), 1.89 (s, 0.68H*), 1.86 (s, 3H), 1.84 – 1.79 (m, 3H), 1.71 (d, J = 1.7 Hz, 3H). *Indicates the peaks of rotamer (5:1).

^{13}C NMR (126 MHz, CDCl$_3$): δ 172.2, 169.5, 164.6, 150.6, 145.8, 144.4, 144.4, 138.8, 137.5, 135.2, 133.1, 132.1, 132.0, 129.6, 129.6, 128.6, 128.6, 126.2, 124.6, 124.1, 123.5, 122.0, 110.7, 87.1, 85.4, 60.9, 58.4, 50.9, 41.6, 37.8, 20.9, 20.6, 19.8, 12.6.

HRMS (ESI+): m/z calcd for $C_{35}H_{37}N_6O_7$ $[M+H]^+$: 653.2724, found 653.2735.

Enantioselectivity: 91% *de*.

Chiral HPLC method: IA column, 254 nm, 20% isopropanol/hexanes, flow rate 1 mL/min, room temperature, t_R (major) = 36.57 min, t_R (minor) = 49.41 min.

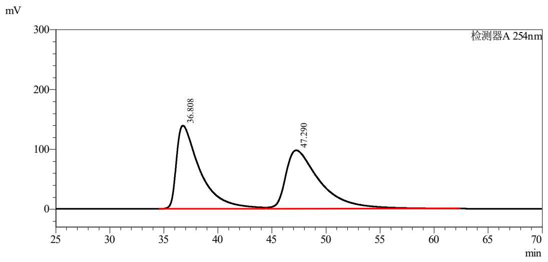

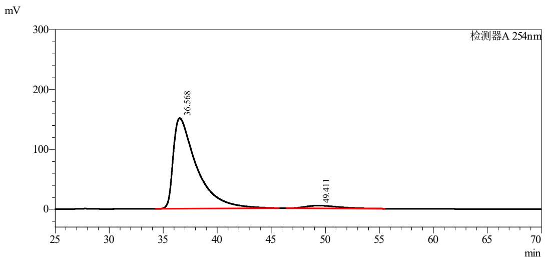

<峰表>

检测器A 254nm

峰号	保留时间	面积	高度	浓度
1	36. 808	21310624	139193	49. 591
2	47. 290	21662455	97727	50. 409
总计		42973080	236920	

<峰表>

检测器A 254nm

峰号	保留时间	面积	高度	浓度
1	36. 568	22949976	151486	95. 883
2	49. 411	985381	4654	4. 117
总计		23935356	156139	

Crystallographic information

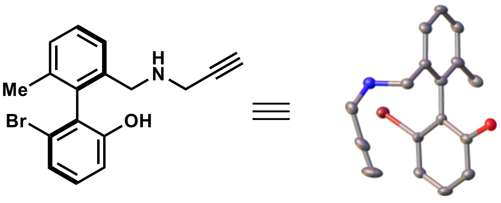

(S)-**3h**

CDCC:2426714

Identification code	exp_9720
Empirical formula	C17H16BrNO
Formula weight	330.22
Temperature / K	116.6(3)
Crystal system	monoclinic
Space group	$P2_1$
a / Å, b / Å, c / Å	9.5166(13), 8.0759(14), 9.8904(17)
α/°, β/°, γ/°	90, 97.671(15), 90
Volume / $Å^3$	753.3(2)
Z	2
ρcalc / mg mm^{-3}	1.456
μ / mm^{-1}	3.668
F(000)	336
Crystal size / mm^3	0.210 × 0.170 × 0.007
2Θ range for data collection	9.378 to 134.098°
Index ranges	$-7 \leq h \leq 11$, $-9 \leq k \leq 9$, $-11 \leq l \leq 11$
Reflections collected	4486
Independent reflections	2566[R(int) = 0.0483 (inf-0.9Å)]
Data/restraints/parameters	2566/1/183
Goodness-of-fit on F^2	1.057
Final R indexes [I>2σ (I) i.e. Fo>4σ (Fo)]	$R_1 = 0.0485$, $wR_2 = 0.1234$
Final R indexes [all data]	$R_1 = 0.0506$, $wR_2 = 0.1269$
Largest diff. peak/hole / e $Å^{-3}$	1.230/-0.619
Flack Parameters	-0.04(3)

SI Table 7. Crystal data and structure refinement for compound (S)-**3h** (CCDC: 2426714).

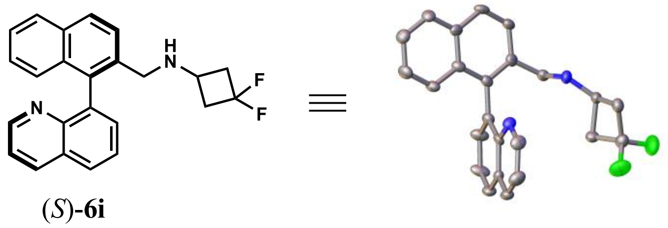

(S)-**6i**

CDCC: 2422607

Identification code	exp_9612
Empirical formula	C24H20F2N2
Formula weight	374.42
Temperature / K	114.8(2)
Crystal system	orthorhombic
Space group	$P2_12_12_1$
a / Å, b / Å, c / Å	8.1055(3), 19.0485(7), 12.0578(7)
α/°, β/°, γ/°	90, 90, 90
Volume / $Å^3$	1861.71(15)
Z	4
ρcalc / mg mm^{-3}	1.336
μ / mm^{-1}	0.755
F(000)	784
Crystal size / mm^3	0.310 × 0.230 × 0.200
2Θ range for data collection	8.68 to 132.946°
Index ranges	$-9 \leq h \leq 3$, $-21 \leq k \leq 22$, $-14 \leq l \leq 12$
Reflections collected	5968
Independent reflections	3190[R(int) = 0.0344 (inf-0.9Å)]
Data/restraints/parameters	3190/298/327
Goodness-of-fit on F^2	1.147
Final R indexes [I>2σ (I) i.e. Fo>4σ (Fo)]	R_1 = 0.0791, wR_2 = 0.1816
Final R indexes [all data]	R_1 = 0.0816, wR_2 = 0.1830
Largest diff. peak/hole / e $Å^{-3}$	0.262/-0.302
Flack Parameters	0.03(18)

SI Table 8. Crystal data and structure refinement for compound (S)-**6i** (CCDC: 2422607).

Density functional theory (DFT) calculations

Computational Details

In our calculations, Gaussian 16 program[11] was used to carry out total density functional theory (DFT) calculations. Geometry optimizations were performed by B3LYP functional[12,13] with dispersion correction of D3(BJ),[14] 6-31+G(d) basis set for the all elements. The vibrational frequencies calculations were conducted at the same level of theory to be sure whether every optimized stationary point is an energy minimum or a transition state and evaluate the zero-point vibrational energy and thermal corrections at 298 K. The single point energies and solvent effects based on the gas-phase optimized structures were calculated by the UM06 functional[15,16] with dispersion correction of D3,[17] and def2-TZVP basis set[18] was used for all elements. In order to adjust the Gibbs free energies from 1 atm to 1 mol/L, a correction of $RT\ln(\text{Csol}/\text{Cgas})$ (1.89 kcal/mol) is added to energies of all species. Csol represents the standard molar concentration in solution (1 mol/L), Cgas represents the standard molar concentration in gas phase (0.0446 mol/L), and *R* represents the gas constant.

SI Figure 9. Rotational barriers of selected substrates and their corresponding imine intermediates.

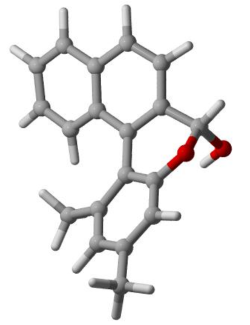

TS-1a

$\Delta G^{\neq}$ = 26.2 kcal/mol

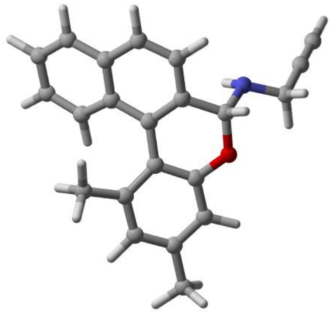

TS-3aI

$\Delta G^{\neq}$ = 13.3 kcal/mol

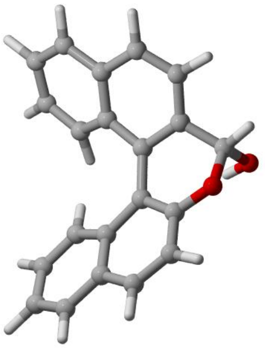

TS-1f

$\Delta G^{\neq}$ = 28.3 kcal/mol

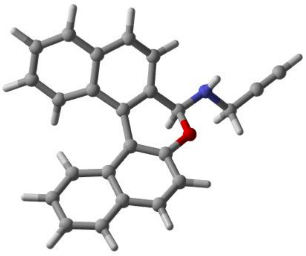

TS-3fI

$\Delta G^{\neq}$ = 26.3 kcal/mol

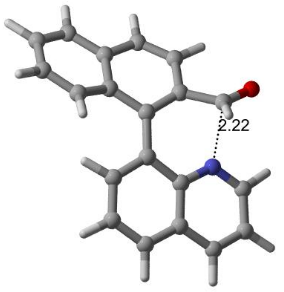

TS-4a
ΔG≠ = 25.9 kcal/mol

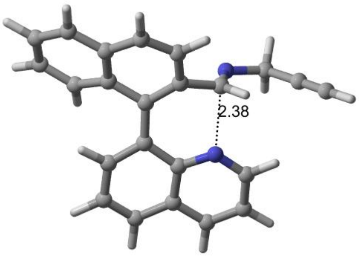

TS-5aI
ΔG≠ = 29.8 kcal/mol

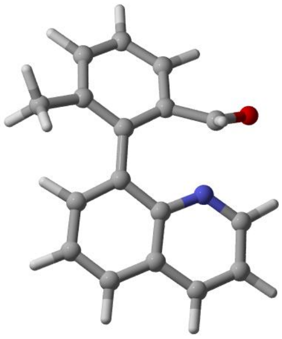

TS-4h
ΔG≠ = 24.9 kcal/mol

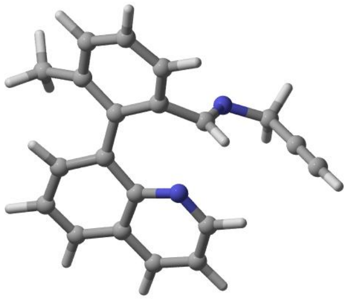

TS-5hI
ΔG≠ = 31.0 kcal/mol

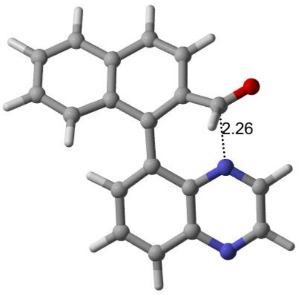

TS-4m
ΔG≠ = 26.1 kcal/mol

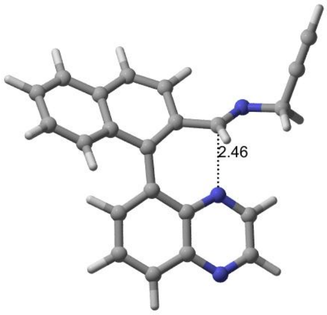

TS-5mI
ΔG≠ = 29.7 kcal/mol

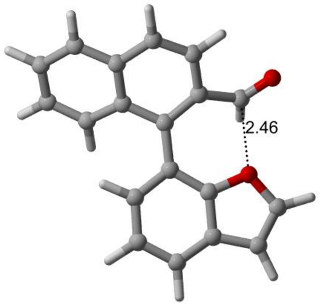

TS-4n
ΔG≠ = 24.3 kcal/mol

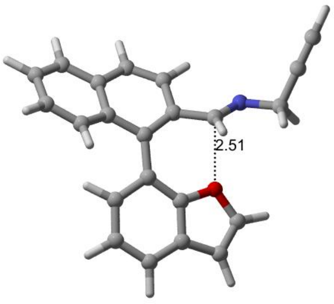

TS-5nI
ΔG≠ = 25.8 kcal/mol

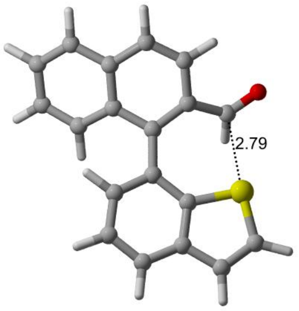

TS-4o
ΔG≠ = 32.6 kcal/mol

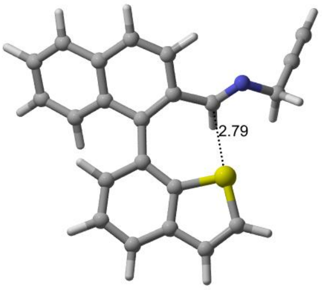

TS-5oI
ΔG≠ = 32.8 kcal/mol

SI Table 9. Zero-point correction (ZPE), thermal correction to enthalpy (TCH), thermal correction to Gibbs free energy (TCG), energies (E), Gibbs free energies(G), and enthalpies (H) (in Hartree) of the structures calculated at the M06-D3/def2-TZVP//B3LYP-D3(BJ)/6-31+G(d) level of theory.

	ZPE	TCH	TCG	E	G	H	Imaginary Frequency
**1a**	0.296935	0.316464	0.24875	-883.806231	-883.557481	-883.489767	-
**TS-1a**	0.301574	0.318486	0.258244	-883.773992	-883.515748	-883.455506	-75.18
**3aI**	0.346735	0.369841	0.292546	-979.319285	-979.026739	-978.949444	-
**TS-3aI**	0.349689	0.369628	0.302952	-979.308515	-979.005563	-978.938887	-38.32
**1f**	0.289109	0.307612	0.242502	-958.781639	-958.539137	-958.474027	-
**TS-1f**	0.291244	0.307723	0.249042	-958.743053	-958.494011	-958.43533	-97.1
**3fI**	0.338688	0.36072	0.288023	-1054.293839	-1054.005816	-1053.933119	-
**TS-3fI**	0.341282	0.361384	0.293668	-1054.257603	-1053.963935	-1053.896219	-91.6
**4a**	0.272815	0.289979	0.22725	-899.599258	-899.372008	-899.309279	-
**TS-4a**	0.27402	0.289685	0.23273	-899.563439	-899.330709	-899.273754	-30.17
**5aI**	0.322371	0.343186	0.270757	-995.108804	-994.838047	-994.765618	-
**TS-5aI**	0.322291	0.341819	0.276018	-995.066654	-994.790636	-994.724835	-29.67
**4h**	0.254418	0.270439	0.211663	-785.32254	-785.110877	-785.052101	-
**TS-4h**	0.255443	0.269783	0.216018	-785.28719	-785.071172	-785.017407	-37.15
**5hI**	0.303989	0.323751	0.255619	-880.834722	-880.579103	-880.510971	-

**TS-5hI**	0.304305	0.322543	0.259892	-880.789532	-880.52964	-880.466989	-41.83
**4m**	0.26081	0.277846	0.215146	-915.639248	-915.424102	-915.361402	-
**TS-4m**	0.261274	0.27688	0.21991	-915.602425	-915.382515	-915.325545	-26.55
**5mI**	0.310399	0.331079	0.258753	-1011.149018	-1010.890265	-1010.817939	-
**TS-5mI**	0.310482	0.329851	0.26358	-1011.106573	-1010.842993	-1010.776722	-30.63
**4n**	0.254669	0.271037	0.210727	-881.376816	-881.166089	-881.105779	-
**TS-In**	0.254877	0.269996	0.214193	-881.341496	-881.127303	-881.0715	-36.46
**5nI**	0.304242	0.324256	0.25456	-976.886802	-976.632242	-976.562546	-
**TS-5nI**	0.304322	0.323133	0.25811	-976.849313	-976.591203	-976.52618	-35.51
**4o**	0.251287	0.268266	0.206065	-1204.35725	-1204.151185	-1204.088984	-
**TS-4o**	0.251266	0.266967	0.209779	-1204.30899	-1204.099211	-1204.042023	-41.73
**5oI**	0.300955	0.321535	0.250429	-1299.867452	-1299.617023	-1299.545917	-
**TS-5oI**	0.300797	0.320158	0.253812	-1299.818505	-1299.564693	-1299.498347	-35.69

Cartesian coordinates of the optimized structure

1a

C	2.89633000	-0.16309100	-0.13937800
C	1.51831800	-0.52850500	-0.28569800
C	0.50117800	0.37631000	0.15481100
C	0.86813800	1.60094900	0.70522900
C	2.23909300	1.95652500	0.84154600
C	3.22514400	1.09773000	0.43341400
C	-0.93235900	-0.02363300	0.02226400
C	-1.55727400	-0.83192500	0.98946300
C	-2.89274900	-1.21143300	0.81065800
C	-3.62870800	-0.80717700	-0.30857600
C	-3.00026900	-0.00802600	-1.26832900
C	-1.66891800	0.37778100	-1.10147600
C	-0.15920900	2.56168200	1.16291900
C	3.89971900	-1.06882000	-0.57168000
C	3.56403200	-2.28479400	-1.12764100
C	2.20326100	-2.64596500	-1.27768500
C	1.20590600	-1.78793300	-0.86728600
H	2.47156700	2.92282700	1.27761900
H	4.27317100	1.36733200	0.53944700
H	-3.54998000	0.31811300	-2.15039400

H	4.94288300	-0.78451400	-0.45642300
H	4.34281100	-2.96859900	-1.45420900
H	1.94601000	-3.60397000	-1.72107400
H	0.16348800	-2.06493800	-0.98682500
H	-1.20909900	2.23917000	1.03714700
O	0.09448500	3.65022200	1.65627000
O	-1.01854700	1.16507200	-2.01697300
H	-1.62169800	1.39174500	-2.74138400
C	-0.79158800	-1.28436100	2.20926200
H	-1.42947100	-1.87409600	2.87411000
H	0.07367200	-1.89825200	1.93220200
H	-0.40358700	-0.43024100	2.77659200
C	-5.07885000	-1.19635100	-0.46720700
H	-5.74085800	-0.39538100	-0.11233600
H	-5.33092400	-1.38787000	-1.51619800
H	-5.31403200	-2.09682800	0.10931900
H	-3.36882500	-1.83513000	1.56349200

TS-1a

C	-2.92738100	0.32957000	0.57755400
C	-1.80814100	-0.34929600	-0.02912200
C	-0.52786900	0.35086600	-0.14194100
C	-0.62428700	1.74714100	-0.02952800
C	-1.76572500	2.42104000	0.45301600
C	-2.86776400	1.71804100	0.84810800
C	0.88578500	-0.20204500	-0.15759000
C	1.89508000	0.74013000	0.22406500
C	3.21308000	0.40817700	0.53813800
C	3.65219300	-0.89744600	0.44934000
C	2.72322500	-1.83518000	-0.00504800
C	1.39057600	-1.54405200	-0.31140400
C	0.56467400	2.58382100	-0.37353100
C	-4.14334600	-0.36296600	0.81832400
C	-4.33696000	-1.65007100	0.37948000
C	-3.31712600	-2.26019700	-0.37555300
C	-2.10891700	-1.62420000	-0.57053900
H	-1.72784700	3.50223400	0.55111300
H	-3.72602900	2.21063400	1.29594900
H	3.05860700	-2.86034200	-0.13320300
H	0.45840200	3.61513500	-0.03710900
H	-4.94151200	0.17556600	1.32275600
H	-5.27763200	-2.16296300	0.55655600
H	-3.48616700	-3.23000300	-0.83522100
H	-1.40592600	-2.08560100	-1.23192900

C	0.65079600	-2.76416400	-0.82103800
H	-0.13506800	-3.10140700	-0.14269500
H	0.21456100	-2.60146900	-1.81172400
H	1.35778700	-3.59010500	-0.92805400
C	5.05997600	-1.29590200	0.80625700
H	5.09568100	-1.76076400	1.80016800
H	5.46127100	-2.02535800	0.09403600
H	5.72835200	-0.42973300	0.82279000
O	1.67205900	2.07988300	0.35042500
H	3.87066100	1.21952700	0.83267400
O	0.82269800	2.65686300	-1.75090500
H	0.70054500	1.76603300	-2.11929400

3aI

C	-3.75969900	0.12693900	-2.98752900
C	-3.86418600	-0.51790300	-1.71138100
C	-2.99431300	-0.16450600	-0.59816700
C	-2.15946400	0.95371700	-0.84949700
C	-1.95828800	1.48308300	-2.15479700
C	-2.76455400	1.11380600	-3.19152000
C	-3.33515000	-0.38660500	0.84802900
C	-4.55344200	-0.97127300	1.31493400
C	-4.71388200	-1.49067700	2.59982900
C	-3.65116200	-1.55933000	3.48833900
C	-2.40341800	-1.14981800	3.01662200
C	-2.21336500	-0.67824100	1.71731600
C	-1.62803600	1.81734800	0.22807700
C	-4.61597500	-0.26306400	-4.04903800
C	-5.48100700	-1.32439300	-3.91804000
C	-5.47382200	-2.07565900	-2.72343400
C	-4.66628700	-1.70045500	-1.67178300
N	-0.65921800	2.62482700	0.03647800
C	-0.30975300	3.49955500	1.15369800
C	-0.49327100	4.90928400	0.79808100
C	-0.65223100	6.07070300	0.51274700
H	-1.22704900	2.27623900	-2.26122100
H	-2.66840200	1.57627200	-4.17017900
H	-1.52988000	-1.29752700	3.64708900
H	-2.16200600	1.77510200	1.18623000
H	-4.54575200	0.28005000	-4.98784300
H	-6.12510600	-1.61494100	-4.74275200
H	-6.08087700	-2.97237500	-2.64177400
H	-4.62102000	-2.33361900	-0.79501700
H	-0.89259200	3.27230800	2.06418600

H	0.74781400	3.32646100	1.39760200
H	-0.79498900	7.09491700	0.25368800
C	-0.75807500	-0.65113200	1.27680100
H	-0.64404800	-0.82518300	0.20548600
H	-0.24992300	0.29137400	1.50426700
H	-0.21615700	-1.44035000	1.80584800
C	-3.83438900	-2.06676000	4.89454100
H	-2.92218500	-2.54335300	5.26848500
H	-4.07551400	-1.24298200	5.57914800
H	-4.65257200	-2.79154700	4.95523800
O	-5.75035200	-0.82132600	0.66437900
H	-5.60564700	-0.44349700	-0.21732800
H	-5.71168300	-1.81199300	2.88160700

TS-3aI

C	1.79533900	-2.51074900	0.26661100
C	1.68139400	-1.13330700	-0.11550700
C	0.56951800	-0.35370900	0.37532500
C	-0.51738200	-1.05066700	0.89469400
C	-0.41477400	-2.41841400	1.24106900
C	0.74758700	-3.11441500	1.01233700
C	0.49250300	1.11721700	0.30061900
C	-0.79877900	1.69535700	0.32875800
C	-1.00895000	3.05181000	0.09728100
C	0.08037800	3.91422000	-0.04657400
C	1.36155700	3.38460300	0.12961300
C	1.58994400	2.01800200	0.34033300
C	-1.89607900	-0.43622000	0.91906000
C	2.91078800	-3.26742300	-0.18343800
C	3.82638200	-2.72829200	-1.06001000
C	3.64267300	-1.41054000	-1.54176100
C	2.60182600	-0.63567800	-1.07579000
N	-2.71288500	-1.14881400	-0.02941900
C	-4.10340100	-0.68345000	-0.11491000
C	-4.86929600	-1.48830300	-1.06645900
C	-5.49830800	-2.13497700	-1.87123800
H	-1.27724800	-2.91096400	1.68036200
H	0.84324700	-4.15297800	1.31807100
H	2.21487800	4.05939100	0.14608100
H	-2.38382700	-0.54056600	1.89594900
H	3.00355600	-4.29950600	0.14633500
H	4.66411000	-3.32355200	-1.41280900
H	4.31896000	-1.01061500	-2.29247200
H	2.45189400	0.36004300	-1.47563900

H	-4.54540500	-0.77593300	0.88641300
H	-4.17587300	0.37853600	-0.39554600
H	-6.05316900	-2.71463900	-2.57477800
C	2.99010800	1.62310500	0.77001500
H	3.01175100	0.65985700	1.28226700
H	3.70046800	1.56561800	-0.06201900
H	3.36706300	2.38263900	1.46411100
C	-0.13583100	5.38713100	-0.29461300
H	0.81368600	5.90530300	-0.46151100
H	-0.77096800	5.55182400	-1.17358100
H	-0.63475500	5.86567900	0.55810800
O	-1.91137200	0.98849700	0.67669600
H	-2.03018900	3.42262700	0.08444000
H	-2.27299600	-1.16841900	-0.94805500

1f

C	2.89542400	0.42131800	-0.36869000
C	1.59856800	-0.16678000	-0.54051600
C	0.44922200	0.45969800	0.04003100
C	0.61596600	1.64323000	0.76398600
C	1.90876000	2.21171700	0.92504200
C	3.01727300	1.62258400	0.37625900
C	-0.88560800	-0.18281200	-0.14177300
C	-1.72700100	0.25842700	-1.14896900
C	-2.97656000	-0.36304000	-1.38305100
C	-3.37526300	-1.41603200	-0.60071300
C	-2.55223800	-1.90364600	0.44864300
C	-1.28584100	-1.27655800	0.68228100
C	-0.50121300	2.36463300	1.42771300
C	4.02551100	-0.21234600	-0.94676000
C	3.88768200	-1.37711700	-1.66528800
C	2.60895000	-1.95910000	-1.83915500
C	1.49319000	-1.36993400	-1.29236300
H	2.00348100	3.12972500	1.50008000
H	4.00178100	2.06429400	0.50455200
H	-0.15521500	3.23562900	2.03000500
H	5.00345400	0.24158700	-0.81007000
H	4.75998000	-1.85374800	-2.10306000
H	2.51033700	-2.87773300	-2.40977800
H	0.51413400	-1.81461500	-1.42556700
O	-1.41097900	1.29820600	-1.97231700
H	-3.59746000	0.02228500	-2.18487100
O	-1.68270300	2.09572600	1.36571700
H	-4.33568700	-1.89320300	-0.77715100

C	-0.46988200	-1.77447100	1.73307600
H	0.49000400	-1.30504900	1.92225400
C	-2.94705200	-2.99284500	1.26579900
C	-2.13326000	-3.45421400	2.27559400
H	-2.44754600	-4.28910400	2.89507100
C	-0.88312400	-2.83531200	2.50791400
H	-0.24360700	-3.19953100	3.30723700
H	-3.91181300	-3.45799500	1.07806100
H	-0.56434100	1.67505200	-1.67939700

TS-1f

C	-2.90549100	0.37054400	-0.60301100
C	-1.67749500	0.59817700	0.11879600
C	-0.72867800	-0.51389900	0.24422000
C	-1.33259600	-1.78200700	0.16326500
C	-2.60477800	-2.00274500	-0.40692300
C	-3.32662000	-0.95095000	-0.90449300
C	0.77899400	-0.51654200	0.18110500
C	1.35123900	-1.76618900	-0.14740400
C	2.64965900	-1.93802800	-0.68201500
C	3.45393100	-0.85977700	-0.88977200
C	3.02351900	0.42753100	-0.47676300
C	1.72247900	0.60796800	0.12012800
C	-0.54703500	-2.96850100	0.61025400
C	-3.77457700	1.45678100	-0.89415200
C	-3.55923600	2.70971200	-0.36711300
C	-2.49473700	2.88090300	0.54049900
C	-1.59846900	1.85554400	0.77294300
H	-2.96716400	-3.02317900	-0.49783900
H	-4.26127200	-1.10462300	-1.43648600
H	-1.01042300	-3.91299800	0.32870200
H	-4.65651200	1.25542400	-1.49733400
H	-4.24110800	3.52769600	-0.58107600
H	-2.39265800	3.81180800	1.09148600
H	-0.89560200	1.98729700	1.57766900
O	0.69523300	-2.95435300	-0.07855500
H	2.94957400	-2.94856600	-0.93818400
O	-0.36141800	-3.03862900	2.00294000
H	-0.18469400	-2.14464200	2.34184100
H	4.43873900	-0.97384500	-1.33443100
C	1.49642600	1.90166600	0.65025000
H	0.61296600	2.09099400	1.21769700
C	3.91980400	1.51673600	-0.62138000
C	3.61338400	2.77394300	-0.15135100

H	4.31035200	3.59910200	-0.26506800
C	2.39052000	2.94844400	0.51993500
H	2.14195700	3.91016300	0.96101600
H	4.87659200	1.32484700	-1.10118500

3fI

C	2.95030500	0.50440100	-0.79702100
C	1.63179400	-0.05762600	-0.74951500
C	0.66204800	0.47160100	0.16397200
C	1.02401400	1.52549800	1.00660800
C	2.34701000	2.04345600	0.97118800
C	3.27909300	1.56099700	0.08888100
C	-0.69601200	-0.14767800	0.21504800
C	-1.62530100	0.17284700	-0.76010000
C	-2.92632500	-0.38307900	-0.74718700
C	-3.29122600	-1.24585500	0.25376000
C	-2.37913300	-1.60389400	1.28073500
C	-1.05807600	-1.05175200	1.25800400
C	0.10383000	2.11816500	1.99598400
C	3.89690200	-0.02049300	-1.71468400
C	3.56566000	-1.06150700	-2.55014700
C	2.26916800	-1.62731300	-2.49800000
C	1.32768400	-1.14028600	-1.62156700
H	2.60760200	2.84854600	1.65359100
H	4.28249900	1.97756700	0.06187300
H	0.59781400	2.57044400	2.87360700
H	4.89182200	0.41645400	-1.74194100
H	4.29800000	-1.45498300	-3.24915600
H	2.01741900	-2.45406900	-3.15576300
H	0.33719800	-1.57834300	-1.57904200
O	-1.35305600	1.03864500	-1.77886300
H	-3.61515700	-0.09375500	-1.53394200
H	-4.29333600	-1.66628800	0.27356400
C	-0.15721000	-1.42207400	2.29121800
H	0.84888600	-1.01595500	2.28123500
C	-2.74329300	-2.49002600	2.32554500
C	-1.84960800	-2.82266600	3.31857600
H	-2.14009000	-3.50339000	4.11389300
C	-0.54348200	-2.28086700	3.29577500
H	0.16245300	-2.54758500	4.07754200
H	-3.74947400	-2.90305800	2.32632700
N	-1.16112000	2.11368100	1.86935700
C	-1.94455800	2.62689900	2.99068000
H	-2.68712700	3.33266400	2.59646200

H	-1.32583800	3.17288900	3.72578000
C	-2.62252800	1.50687400	3.64828900
C	-3.13998500	0.54788800	4.16507000
H	-3.57799300	-0.32180900	4.59734700
H	-0.46672700	1.41053600	-1.63324400

TS-3fI

C	0.46385400	2.96529200	-0.62168800
C	0.95495700	1.82908100	0.11724800
C	0.11805600	0.62890900	0.18460500
C	-1.25889300	0.89214800	0.05593100
C	-1.76589400	2.08065500	-0.51083500
C	-0.90672000	3.04279700	-0.97582700
C	0.51650900	-0.82890400	0.12348700
C	-0.51268000	-1.69669700	-0.30872900
C	-0.30495300	-2.98673900	-0.85098000
C	0.95351200	-3.49631600	-0.94646800
C	2.05036400	-2.75539400	-0.43795800
C	1.84984100	-1.44683200	0.13901200
C	-2.19335300	-0.20805900	0.46130600
C	1.30970700	4.08448600	-0.85472300
C	2.53850200	4.19328400	-0.24516600
C	2.91261600	3.20598100	0.69014300
C	2.13869300	2.07530000	0.86344100
H	-2.83914400	2.20622600	-0.59799900
H	-1.27225700	3.91924700	-1.50388500
H	0.92794400	4.89031400	-1.47704700
H	3.16779200	5.06257800	-0.41344500
H	3.79395400	3.34844400	1.30958100
H	2.37935400	1.43002100	1.69247100
O	-1.82587400	-1.35568000	-0.34425300
H	-1.18055800	-3.52736300	-1.19506800
H	1.12945900	-4.47668600	-1.38090400
C	3.01431400	-0.87993900	0.71359300
H	2.94612000	0.04712200	1.23540200
C	3.33830400	-3.34739500	-0.48707300
C	4.44402000	-2.72234800	0.04272200
H	5.42458800	-3.18751000	0.00036000
C	4.26056300	-1.47671700	0.66834100
H	5.10110800	-0.97230100	1.13784400
H	3.42681900	-4.32780700	-0.94923200
N	-3.57688100	0.07052200	0.29362200
H	-3.79709800	0.23049100	-0.68751000
C	-4.46859600	-0.95887400	0.84893000

H	-4.28210000	-1.01435800	1.92907300
H	-4.26481300	-1.95888100	0.43497400
C	-5.87119700	-0.61884000	0.61056700
C	-7.02980200	-0.35246600	0.39241000
H	-8.05287400	-0.11049400	0.20849900
H	-2.02373400	-0.49414400	1.51061700

4a

C	-2.27722000	0.37122200	-2.81092900
C	-2.43316700	-0.21826700	-1.51410100
C	-3.74633800	-0.36874100	-0.96874800
C	-4.84851400	0.06401900	-1.69952100
C	-4.68168300	0.64399200	-2.98838000
C	-3.43200700	0.79289600	-3.52840900
C	-3.90496300	-1.01037600	0.37057100
C	-3.80985400	-0.21894800	1.55601000
C	-3.93844400	-0.85251600	2.83341700
C	-4.15586500	-2.25318200	2.90213000
C	-4.24250100	-2.99724700	1.74662000
C	-4.11442000	-2.37204200	0.48256400
C	-6.21547600	-0.03841100	-1.14390000
N	-3.60476500	1.12378000	1.42447200
C	-3.52110000	1.84979200	2.52114400
C	-3.63290300	1.32232400	3.83346900
C	-3.84212900	-0.02840600	3.98438300
C	-0.97239500	0.52004200	-3.34827300
C	0.13637500	0.10928300	-2.63956400
C	-0.01517700	-0.47166800	-1.35755200
C	-1.26951700	-0.63230600	-0.80993200
H	-5.57235900	0.96510300	-3.51878500
H	-3.30338600	1.23834300	-4.51189900
H	-4.25367300	-2.72500700	3.87693000
H	-4.41026200	-4.06927200	1.79651700
H	-4.18106200	-2.97171100	-0.42114300
H	-3.35725600	2.91706900	2.37977600
H	-3.55491000	1.98349200	4.69126700
H	-3.93669800	-0.47727900	4.97047400
H	-0.86270700	0.96744900	-4.33318400
H	1.12978600	0.23044400	-3.06286100
H	0.86327400	-0.79066800	-0.80346400
H	-1.37943900	-1.07735600	0.17321700
O	-7.22483600	0.29382200	-1.74627500
H	-6.29036300	-0.43856000	-0.11603700

TS-4a

C	-3.00585000	0.60918300	-0.45954100
C	-2.03585900	-0.27644400	0.12405700
C	-0.65294400	0.16781600	0.24614000
C	-0.46794200	1.56385600	0.24891900
C	-1.45449400	2.43422500	-0.27199600
C	-2.65490500	1.95751100	-0.72313100
C	0.49730700	-0.78816400	0.19838700
C	1.86633900	-0.34961400	-0.01229700
C	2.93969400	-1.29304400	-0.14356400
C	2.67646800	-2.68044600	-0.09852900
C	1.37362800	-3.09827200	0.00399200
C	0.31909200	-2.17192700	0.12494200
C	0.67548400	2.26621900	0.92157100
N	2.13530100	0.96889000	-0.14174200
C	3.35214600	1.41906800	-0.34326500
C	4.47093500	0.56411400	-0.43966500
C	4.25081900	-0.78891000	-0.34047100
C	-4.33397800	0.16220600	-0.68270000
C	-4.75561500	-1.07247500	-0.24887800
C	-3.85902000	-1.88078400	0.47930000
C	-2.54663800	-1.49401100	0.65713500
H	-1.19888100	3.48792200	-0.31052000
H	-3.38394800	2.61438900	-1.18998200
H	3.49843700	-3.38654500	-0.17743500
H	1.13246200	-4.15704000	-0.01544700
H	-0.67304600	-2.58672000	0.11544700
H	1.09555300	1.71176500	1.78235900
H	3.44803800	2.50108200	-0.42152300
H	5.46422900	0.97181600	-0.59562100
H	5.07205500	-1.49642000	-0.42205300
H	-5.02774100	0.84463000	-1.16703500
H	-5.77957700	-1.39615100	-0.41002900
H	-4.20821000	-2.80434400	0.93229200
H	-1.92596700	-2.09438400	1.30783800
O	0.93509400	3.44824300	0.75264700

5aI

C	2.89799600	-1.38655000	-0.58418400
C	2.30417300	-0.27325800	0.09675700
C	0.86997500	-0.02487000	-0.02829600
C	0.09398300	-1.16181200	-0.35253900
C	0.70347500	-2.26782900	-1.00861000
C	2.05379900	-2.33076300	-1.22437600

C	0.35883400	1.37857100	0.06159800
C	-1.03178700	1.77951700	-0.06013100
C	-1.41075500	3.16140800	0.06990900
C	-0.42627100	4.16477500	0.22750300
C	0.89703300	3.80008800	0.16968300
C	1.26712200	2.44466600	0.06600000
C	-1.29010700	-1.41390100	0.11434200
N	-1.97180700	0.86013800	-0.38124800
C	-3.23670800	1.19807900	-0.51312100
C	-3.71386300	2.51245200	-0.31138700
C	-2.78766700	3.48832300	-0.02657400
C	4.30210700	-1.59058800	-0.52510400
C	5.10420300	-0.80367000	0.27088600
C	4.50751100	0.19554700	1.07105500
C	3.15413300	0.45430900	0.97915500
N	-2.03796500	-2.30053500	-0.42173800
C	-3.28976400	-2.59445800	0.27036000
C	-3.33536300	-3.98534600	0.73533400
C	-3.37258700	-5.12924200	1.12558300
H	0.04135500	-3.06682600	-1.32470300
H	2.49507100	-3.15672000	-1.77624700
H	-0.72874800	5.20337100	0.33417400
H	1.67974300	4.55301100	0.19949300
H	2.32115300	2.24438800	-0.04403700
H	-1.55572500	-0.94416800	1.06787800
H	-3.91788200	0.39780800	-0.80079500
H	-4.77180300	2.73673300	-0.40671600
H	-3.08764200	4.52586000	0.10056100
H	4.72466600	-2.41964200	-1.08769200
H	6.17445900	-0.98218200	0.32494300
H	5.11110800	0.75266800	1.78257800
H	2.71977100	1.18579100	1.65001200
H	-3.46092600	-1.92229300	1.13089500
H	-4.11812400	-2.43327700	-0.43380600
H	-3.39726000	-6.14027200	1.46570500

TS-5aI

C	3.01095000	-1.60143200	0.42851500
C	2.37427300	-0.40695800	-0.04643800
C	0.94010000	-0.21696900	0.15036200
C	0.20168300	-1.39867600	0.34092800
C	0.84806600	-2.57842400	0.79654000
C	2.20994200	-2.65489200	0.93609300
C	0.38658400	1.16776700	0.23112500

C	-1.01842300	1.51567300	0.31731400
C	-1.43029300	2.89224000	0.26440200
C	-0.46783200	3.93021000	0.27041200
C	0.85835600	3.59646900	0.40167000
C	1.26228900	2.24572700	0.39774100
C	-1.21729000	-1.64265500	-0.04431100
N	-1.94294100	0.55032400	0.53223200
C	-3.22688100	0.84312800	0.58585500
C	-3.73045500	2.15299800	0.42334300
C	-2.81997300	3.17394700	0.28028500
C	4.41767200	-1.75782700	0.30186200
C	5.17942800	-0.83261100	-0.37481100
C	4.53886300	0.26915000	-0.98489700
C	3.18423000	0.47556400	-0.81883100
N	-1.50086400	-1.70196500	-1.28673400
C	-2.86114800	-2.06061900	-1.69154000
C	-3.79227000	-2.52458200	-0.65234800
C	-4.53379000	-2.89912900	0.22852600
H	0.22598900	-3.43871900	1.03122100
H	2.68506300	-3.54840400	1.33260000
H	-0.79505400	4.96614000	0.23209300
H	1.61620800	4.36849000	0.50211400
H	2.31417400	2.05450200	0.54782000
H	-1.90448700	-1.96999400	0.74268200
H	-3.90110800	0.00769400	0.76892000
H	-4.80000600	2.33812100	0.44741600
H	-3.14436700	4.20935900	0.20589200
H	4.87350800	-2.65606300	0.71171500
H	6.25108000	-0.97318100	-0.48492700
H	5.11053000	0.95059300	-1.60928300
H	2.71598200	1.29740000	-1.34651400
H	-3.29146500	-1.17772800	-2.18620600
H	-2.78181800	-2.82946300	-2.47087600
H	-5.19597200	-3.24439500	0.99119300

4h

C	2.83065400	1.83001600	0.62453000
C	1.52004400	1.38498100	0.81668800
C	1.14966300	0.12829200	0.29672900
C	2.09712500	-0.63770300	-0.41172500
C	3.40624700	-0.16561900	-0.58742300
C	3.77315800	1.06602600	-0.06881000
C	-0.21687200	-0.41148100	0.55823800
C	-1.35574800	0.12088200	-0.12124400

C	-2.65350600	-0.41231900	0.16121400
C	-2.79230300	-1.45621900	1.11025800
C	-1.68405800	-1.95547000	1.75328900
C	-0.40050600	-1.43211600	1.47339000
C	1.73744100	-1.93225900	-1.03882900
N	-1.16224500	1.12172200	-1.02807700
C	-2.21602200	1.60299100	-1.65526800
C	-3.54153000	1.14562800	-1.45101100
C	-3.75472500	0.13855300	-0.54157200
H	4.10319700	-0.78664300	-1.14036500
H	4.78481100	1.43941500	-0.19928600
H	-3.78302700	-1.85315100	1.31609900
H	-1.78602000	-2.75635200	2.47945900
H	0.46579900	-1.83739000	1.98821300
H	0.66662700	-2.20746300	-0.99122400
H	-2.02650700	2.40164300	-2.37132100
H	-4.36038300	1.59009300	-2.00779300
H	-4.75332600	-0.24640100	-0.35022000
O	2.53847200	-2.66369600	-1.59441800
C	0.53198300	2.24071500	1.56837000
H	-0.21505900	2.65372800	0.88162500
H	-0.00918900	1.66202800	2.32469600
H	1.03587600	3.07282700	2.06870800
H	3.11887200	2.79580200	1.03234800

TS-4h

C	3.66844900	0.58390400	-0.18793200
C	2.42172400	1.12073300	0.16056000
C	1.25475200	0.29823500	0.10311900
C	1.48471800	-1.10914700	-0.02467700
C	2.73471600	-1.59484200	-0.41220300
C	3.82450800	-0.74464500	-0.55508500
C	-0.11761800	0.90241000	0.04807300
C	-1.33584400	0.12829400	0.05803200
C	-2.62431900	0.74707000	-0.05847000
C	-2.72094500	2.14974600	-0.20534900
C	-1.56387900	2.88048900	-0.30457000
C	-0.30007200	2.26120700	-0.20582900
C	0.59733900	-2.21779300	0.49991200
N	-1.25831300	-1.21734300	0.11307300
C	-2.32021100	-1.98758200	0.07192900
C	-3.62725900	-1.46066700	0.00389600
C	-3.76627000	-0.09348400	-0.05778600
H	2.83240400	-2.66693700	-0.54670000

H	4.79352100	-1.12608500	-0.86372200
H	-3.69903300	2.61759400	-0.27434800
H	-1.60421100	3.95226500	-0.47562000
H	0.55160400	2.89548300	-0.37618200
H	0.31005900	-2.04147600	1.55652400
H	-2.12080000	-3.05840300	0.09058000
H	-4.48686600	-2.12240200	-0.01148900
H	-4.74954600	0.36467600	-0.12787000
O	0.54596400	-3.34154100	0.01700300
C	2.47773600	2.54453400	0.68966200
H	2.43905600	3.30867700	-0.09770400
H	1.68063100	2.75780000	1.40545400
H	3.43262400	2.68321100	1.20706300
H	4.53588500	1.23855400	-0.15321400

5hI

C	3.87494600	-0.60271700	0.51055900
C	2.65962100	-1.22301700	0.20570000
C	1.54850200	-0.41892000	-0.11706400
C	1.67251800	0.98694500	-0.10584000
C	2.90416000	1.57901100	0.20211900
C	4.00322500	0.78706000	0.50313200
C	0.25341600	-1.06248900	-0.48831400
C	-0.89367700	-0.95697500	0.36099700
C	-2.12980700	-1.54987400	-0.05106100
C	-2.19494800	-2.26058100	-1.27543200
C	-1.07832000	-2.36353200	-2.07143800
C	0.13680800	-1.75809900	-1.67771200
C	0.50091000	1.82735600	-0.40092500
N	-0.77607400	-0.26362700	1.52930200
C	-1.84669500	-0.12601300	2.28217900
C	-3.11870100	-0.66283800	1.96202000
C	-3.25271400	-1.37935500	0.79799100
N	0.52582900	3.09823800	-0.29169900
C	-0.70613100	3.84359500	-0.56397200
C	-1.93714800	3.06295100	-0.75190400
C	-2.91150000	2.35915400	-0.87308600
H	2.96614600	2.66189400	0.20299000
H	4.95866600	1.24534100	0.74294900
H	-3.13775400	-2.71182400	-1.57433000
H	-1.12565200	-2.89967900	-3.01475200
H	1.00101700	-1.82564500	-2.33210100
H	-0.40439500	1.30030000	-0.70603600
H	-1.71780600	0.44025800	3.20368400

H	-3.95805200	-0.50637100	2.63230800
H	-4.20615200	-1.81672100	0.51111700
H	-0.84810400	4.54263500	0.27115900
H	-0.52507500	4.47377600	-1.44623200
H	-3.77799900	1.74693800	-0.97603900
C	2.55264600	-2.72983400	0.25837100
H	2.48009200	-3.17460500	-0.74103400
H	3.43079100	-3.16041800	0.74887300
H	1.66190000	-3.04721900	0.81055800
H	4.73142700	-1.22191900	0.76575400

TS-5hI

C	-3.31155400	-2.16612700	-0.22791600
C	-2.86124000	-0.89580100	0.14717700
C	-1.49661600	-0.52126200	-0.08638400
C	-0.60578100	-1.60167700	-0.36622300
C	-1.09967500	-2.84761200	-0.76364600
C	-2.46033300	-3.12262600	-0.76253400
C	-1.10342400	0.92580500	-0.08780300
C	0.24611100	1.44842500	-0.15294200
C	0.49035400	2.85618700	0.00460400
C	-0.58831600	3.76622300	0.09273200
C	-1.86414000	3.28517800	-0.05233900
C	-2.09996100	1.89965000	-0.15877600
C	0.85980400	-1.66619300	-0.07060800
N	1.27642100	0.62408200	-0.44965700
C	2.51369300	1.07347400	-0.49194500
C	2.85716700	2.41851400	-0.23700900
C	1.83418700	3.30558900	-0.00563700
N	1.21413400	-1.82103600	1.14425100
C	2.63296800	-2.01451700	1.44488200
C	3.56044500	-2.22383100	0.32309600
C	4.29189800	-2.38712500	-0.62558300
H	-0.38584300	-3.62710800	-1.01692900
H	-2.83564300	-4.09148600	-1.07858000
H	-0.38667700	4.82742000	0.21103600
H	-2.71224200	3.96313900	-0.08003100
H	-3.12085500	1.60119500	-0.32276300
H	1.53803100	-1.80404800	-0.91840600
H	3.27935200	0.34048900	-0.74096200
H	3.89517600	2.73464200	-0.25934800
H	2.03099800	4.36417400	0.14526300
H	2.96460400	-1.13540000	2.01686900
H	2.71273900	-2.86397600	2.13649800

H	4.95290500	-2.55243900	-1.44572200
C	-3.88267800	-0.08190000	0.92943700
H	-4.59706000	0.46190400	0.29787100
H	-4.47289900	-0.77315100	1.54027300
H	-3.41477100	0.63911100	1.60267400
H	-4.35914800	-2.40803300	-0.06540500

4m

C	2.58503500	-1.86536400	-0.44993200
C	2.10043500	-0.54902600	-0.15552500
C	0.78267700	-0.39426100	0.37709600
C	-0.00915600	-1.51750400	0.59495000
C	0.48468200	-2.81966400	0.30280900
C	1.74556800	-2.98822100	-0.20465700
C	0.29101600	0.97827100	0.70130100
C	-0.34587600	1.76531100	-0.30422700
C	-0.78929000	3.08986900	0.00670000
C	-0.59086400	3.61035300	1.31006400
C	0.02757800	2.83515500	2.26582900
C	0.46877600	1.52362700	1.95880000
C	-1.39036400	-1.38609200	1.10898700
N	-0.52989700	1.23890700	-1.54885400
C	-1.12593900	2.00843100	-2.43383800
C	-1.56433300	3.32656300	-2.12607300
C	3.89254900	-2.01622300	-0.98003500
C	4.69236000	-0.91837700	-1.21506900
C	4.21531300	0.38288900	-0.92671900
C	2.95102500	0.56205900	-0.40856600
H	-0.17034100	-3.66419300	0.49087500
H	2.12269000	-3.98264000	-0.43048500
H	-0.93867400	4.61738000	1.51807100
H	0.18274700	3.22481200	3.26779800
H	0.95828800	0.93034900	2.72619500
H	-1.74828600	-0.35323900	1.27597500
H	-1.27890900	1.59854300	-3.43057500
H	-2.05367800	3.93208500	-2.88680600
H	4.25298100	-3.01820100	-1.19967900
H	5.69123600	-1.04857400	-1.62232600
H	4.85040100	1.24373000	-1.11626300
H	2.59237400	1.56206200	-0.18891800
N	-1.40430700	3.86344000	-0.93554200
O	-2.12502400	-2.33302900	1.34219700

TS-4m

C	3.04377400	0.56667000	0.38526100
C	2.02633400	-0.29184200	-0.15462000
C	0.65283600	0.18995300	-0.22856900
C	0.50329000	1.59266900	-0.23827200
C	1.53372300	2.43500100	0.24880000
C	2.73807500	1.92507700	0.65832000
C	-0.50592700	-0.74894600	-0.13358600
C	-1.87916300	-0.30419000	0.00661900
C	-2.96246400	-1.23864400	0.14658500
C	-2.70351400	-2.62575200	0.21155700
C	-1.39345700	-3.04138500	0.20266800
C	-0.33139100	-2.12396700	0.05496500
C	-0.61967300	2.32464600	-0.91175500
N	-2.16794000	1.01379200	0.08408700
C	-3.40979200	1.41704600	0.21817700
C	-4.47410200	0.48345700	0.27128300
C	4.36881800	0.08195200	0.55052800
C	4.73456900	-1.16521700	0.09707000
C	3.78192500	-1.95119700	-0.58745700
C	2.47246400	-1.52833800	-0.70406900
H	1.31941800	3.49820200	0.29014000
H	3.50012500	2.56559700	1.09468600
H	-3.54091900	-3.30949700	0.30344000
H	-1.15224400	-4.09449100	0.31687900
H	0.66159400	-2.53263300	0.13772500
H	-1.04423700	1.79525600	-1.78434200
H	-3.57631400	2.49107300	0.28217600
H	-5.50627700	0.81902800	0.34824000
H	5.10407300	0.74286200	1.00299900
H	5.75554900	-1.51787300	0.21162400
H	4.08450600	-2.88546500	-1.05264200
H	1.79689400	-2.11765000	-1.31052600
N	-4.25540200	-0.81412000	0.24623200
O	-0.86813200	3.50799000	-0.72802900

5mI

C	2.63496700	-1.83310300	-0.61282800
C	2.11790100	-0.55632200	-0.21968100
C	0.72924000	-0.43422000	0.10106000
C	-0.10559500	-1.54377900	0.01620300
C	0.42346600	-2.80721000	-0.37402100
C	1.75191900	-2.94670900	-0.67695000
C	0.20369800	0.89530500	0.53170100
C	-0.35246900	1.79460500	-0.42712000

C	-0.83781600	3.07342200	-0.00435800
C	-0.76083100	3.43836100	1.36299500
C	-0.21871600	2.55752900	2.27257700
C	0.26303600	1.29177700	1.85470100
C	-1.53982200	-1.42391500	0.31760700
N	-0.42036100	1.41926000	-1.73671400
C	-0.94540200	2.28681800	-2.57470600
C	-1.42609500	3.55826700	-2.15497800
C	4.01252800	-1.95359800	-0.93022000
C	4.85192600	-0.86146900	-0.87095600
C	4.34250900	0.40278200	-0.48984400
C	3.00929400	0.55061800	-0.17139500
N	-2.34061200	-2.41546600	0.24072700
C	-3.74249600	-2.15332800	0.55842300
C	-4.17962000	-2.92530600	1.72582400
C	-4.54222800	-3.55294800	2.69326500
H	-0.25922000	-3.64850900	-0.42261200
H	2.15023900	-3.91404800	-0.97360300
H	-1.13731000	4.41374700	1.65511300
H	-0.15632400	2.82750500	3.32294900
H	0.68920800	0.61262200	2.58789200
H	-1.89487600	-0.42730100	0.61347200
H	-1.00367700	1.99743200	-3.62258200
H	-1.85449700	4.24823900	-2.87984100
H	4.39530100	-2.92787000	-1.22479500
H	5.90468800	-0.96752900	-1.11810800
H	5.00693500	1.26160000	-0.44971400
H	2.62759700	1.52376500	0.11915600
H	-3.93661600	-1.08074600	0.73714500
H	-4.34443300	-2.45166000	-0.31075600
H	-4.85661500	-4.11321900	3.54526600
N	-1.37844800	3.95108400	-0.90007700

TS-5mI

C	2.85463400	-1.48378600	-0.54700400
C	2.28773200	-0.32852500	0.08467300
C	0.86205800	-0.04611800	-0.06112300
C	0.05541600	-1.16986800	-0.35458400
C	0.63889300	-2.31658600	-0.96305400
C	1.98900400	-2.42686100	-1.15960300
C	0.39341100	1.37366400	-0.02127700
C	-0.98228500	1.82018500	-0.14050400
C	-1.31874100	3.21711900	-0.04062600
C	-0.30569900	4.19660500	0.06241400

C	1.00256600	3.78255400	-0.00732800
C	1.33147600	2.41247900	-0.06670000
C	-1.34127900	-1.36412400	0.10024000
N	-1.96957900	0.94730800	-0.44559200
C	-3.20685400	1.37793900	-0.55254300
C	-3.54091600	2.73534500	-0.32958200
C	4.25172400	-1.72529200	-0.46731000
C	5.06950900	-0.92972100	0.30360600
C	4.49586800	0.11831100	1.05683300
C	3.15131600	0.41147300	0.94302900
N	-2.11058200	-2.24032600	-0.41995200
C	-3.38586800	-2.46581500	0.25482600
C	-3.49389200	-3.83981500	0.75701400
C	-3.58240900	-4.96961100	1.17838000
H	-0.04259300	-3.10732600	-1.25782900
H	2.41114300	-3.28578700	-1.67479300
H	-0.59494900	5.23967300	0.13719800
H	1.81005300	4.50932100	-0.01855300
H	2.37770400	2.17649400	-0.18211900
H	-1.60937500	-0.84816000	1.02915700
H	-3.96581900	0.64842800	-0.83126900
H	-4.57546500	3.07111100	-0.36931500
H	4.65486800	-2.58759900	-0.99279400
H	6.13374100	-1.13590200	0.37429000
H	5.11018100	0.68744200	1.74931300
H	2.73364400	1.18361200	1.57799500
H	-3.54694200	-1.76267300	1.09214800
H	-4.19210500	-2.29295700	-0.47183700
H	-3.65357800	-5.96870800	1.54626300
N	-2.61459100	3.63973200	-0.09458500

4n

C	-2.27442000	-2.12132400	0.33396900
C	-2.03468500	-0.72509500	0.10925000
C	-0.70838600	-0.28749600	-0.20974400
C	0.32609300	-1.21895300	-0.27340100
C	0.07569900	-2.59846100	-0.03516800
C	-1.18924400	-3.03776400	0.25306000
C	-0.45928600	1.16148500	-0.45372000
C	0.22932300	1.95998300	0.45850100
C	0.47670100	3.33565500	0.28123500
C	0.00376800	3.96487900	-0.87997100
C	-0.69078400	3.19360600	-1.81000700
C	-0.91991800	1.82158700	-1.60046200

C	1.70759000	-0.80973700	-0.61815100
C	-3.58941200	-2.55634100	0.64258300
C	-4.62856600	-1.65635200	0.74096800
C	-4.39178000	-0.27555100	0.53937500
C	-3.12714800	0.17836800	0.23224200
H	0.91490700	-3.28350500	-0.09900000
H	-1.38203700	-4.09346300	0.42752700
H	0.17684100	5.02327100	-1.05183100
H	-1.06353400	3.65588100	-2.71956200
H	-1.46379200	1.24734700	-2.34501700
H	1.84962800	0.25457000	-0.87810200
H	-3.76141800	-3.61748800	0.80538200
H	-5.63013300	-2.00333300	0.97979400
H	-5.21213600	0.43080100	0.63124500
H	-2.95336900	1.23844400	0.08499800
C	1.36424200	2.63379000	2.21642400
C	1.22112900	3.74565700	1.45080000
H	1.59631500	4.73357300	1.67961600
H	1.84319500	2.44997700	3.16636600
O	0.77241400	1.53176600	1.64225100
O	2.65747600	-1.57666500	-0.63703200

TS-4n

C	-2.88242200	0.47211100	-0.40714500
C	-1.81275200	-0.30832300	0.14773100
C	-0.46436900	0.23538300	0.16521500
C	-0.37341300	1.64260200	0.09238900
C	-1.45022600	2.40413500	-0.43767900
C	-2.64114500	1.82087100	-0.77924000
C	0.72031100	-0.66810700	0.08893100
C	2.07502100	-0.26181400	0.02347400
C	3.17397600	-1.13356500	-0.15052600
C	2.96280500	-2.50846800	-0.29808100
C	1.64388000	-2.94470200	-0.32530600
C	0.57395500	-2.05519000	-0.16165400
C	0.67925100	2.46698700	0.75322300
C	-4.18780300	-0.08058500	-0.49535800
C	-4.47639600	-1.31548000	0.04054500
C	-3.46412400	-2.02515300	0.72455600
C	-2.17323800	-1.53905800	0.76873400
H	-1.28781900	3.47166100	-0.54755000
H	-3.44112700	2.40123900	-1.23119100
H	3.79349400	-3.19537600	-0.42956400
H	1.42194400	-3.99335600	-0.50144000

H	-0.41547400	-2.46255500	-0.29361300
H	1.17944700	1.98887800	1.61093900
H	-4.96944400	0.51761400	-0.95708800
H	-5.48351700	-1.71882900	-0.01561200
H	-3.70544900	-2.95062200	1.24016900
H	-1.43693900	-2.07179900	1.35735900
C	3.91025200	0.97865500	-0.08889800
C	4.35005700	-0.29707500	-0.20089400
H	5.37491900	-0.61825600	-0.32412700
H	4.39771800	1.94171900	-0.08947600
O	2.54371000	1.02414600	0.04763900
O	0.87895800	3.64424300	0.50462700

5nI

C	-2.38202600	-2.10123900	0.51481000
C	-2.07574900	-0.74005300	0.18683900
C	-0.71182200	-0.36770200	-0.04735300
C	0.29817600	-1.32066700	0.06713200
C	-0.02315600	-2.66634200	0.40347100
C	-1.32249300	-3.04489700	0.61434200
C	-0.39509900	1.04470700	-0.40102000
C	0.23314500	1.91265000	0.49160800
C	0.55090900	3.25529000	0.20516000
C	0.21623300	3.77741100	-1.05333600
C	-0.41631400	2.93616600	-1.96667300
C	-0.71667600	1.59939300	-1.64674700
C	1.70343500	-0.96080800	-0.18035200
C	-3.73254900	-2.47333700	0.74034500
C	-4.74701400	-1.54341700	0.66092500
C	-4.44648000	-0.19466400	0.35590100
C	-3.14459400	0.19690000	0.12573000
N	2.66148800	-1.78086400	0.01965500
C	4.00941400	-1.29733700	-0.27124600
C	4.63993500	-2.08137400	-1.33774700
C	5.16065300	-2.71983100	-2.22239600
H	0.79239500	-3.37746900	0.47679600
H	-1.56194200	-4.07606400	0.86289600
H	0.44572600	4.80759400	-1.31000100
H	-0.68316900	3.31433600	-2.94944900
H	-1.20808700	0.96806100	-2.38150100
H	1.89056700	0.05421200	-0.55474200
H	-3.95254100	-3.51033000	0.98268900
H	-5.77683300	-1.84149500	0.83778400
H	-5.24741000	0.53795600	0.30637800

H	-2.92438300	1.23404400	-0.10190300
H	4.01873100	-0.22863400	-0.54956100
H	4.60657300	-1.39957000	0.64517800
H	5.61457400	-3.28950100	-3.00217700
C	1.21902800	2.72913700	2.27929600
C	1.19466500	3.76086000	1.39695500
H	1.58265900	4.75621600	1.56421600
H	1.59307500	2.63113600	3.28739500
O	0.64318200	1.59234900	1.76086300

TS-5nI

C	2.42590200	-1.89818700	-0.59051300
C	2.10009000	-0.69160400	0.11355100
C	0.75708000	-0.13609500	0.00312100
C	-0.25223500	-1.05971300	-0.34509600
C	0.08814500	-2.26894400	-1.01562100
C	1.39009500	-2.62737300	-1.23348800
C	0.53667400	1.33818100	0.10983600
C	-0.68728000	2.03147200	-0.07577900
C	-0.83004300	3.43874300	-0.03923100
C	0.28548700	4.25941700	0.15572600
C	1.51923400	3.62874300	0.24436500
C	1.63235500	2.23331000	0.19899500
C	-1.65392700	-0.95863300	0.10507300
C	3.75118200	-2.40552500	-0.55268100
C	4.71471400	-1.82503500	0.24211400
C	4.36047400	-0.73341700	1.06457500
C	3.09570200	-0.18300900	0.99627100
N	-2.59260800	-1.64555100	-0.41974800
C	-3.90184400	-1.55220100	0.22036400
C	-4.34398600	-2.85276000	0.73358600
C	-4.70785200	-3.92190500	1.16492200
H	-0.73542500	-2.89647300	-1.33892200
H	1.63765400	-3.52562300	-1.79308200
H	0.19148200	5.34079400	0.18666600
H	2.42784900	4.21830600	0.32770500
H	2.63367800	1.83602900	0.17840400
H	-1.82615600	-0.35064200	0.99997000
H	3.97636400	-3.29834800	-1.13098200
H	5.72039500	-2.23427900	0.27846400
H	5.07894400	-0.33495900	1.77579900
H	2.83657600	0.60591900	1.69113300
H	-3.91390800	-0.81452100	1.04259600
H	-4.62121800	-1.20758200	-0.53575700

H	-5.02264300	-4.86928200	1.54177800
C	-2.80645300	2.51389200	-0.52479600
C	-2.21985600	3.71537200	-0.31405700
H	-2.69409000	4.68550000	-0.36663300
H	-3.80991800	2.20934200	-0.78118100
O	-1.90665900	1.48578800	-0.38814300

4o

C	2.35929400	-2.04537600	-0.50828900
C	2.04125500	-0.68781900	-0.17587900
C	0.73649500	-0.37934800	0.32516700
C	-0.20120500	-1.39750100	0.48003200
C	0.12990300	-2.74238700	0.15426500
C	1.37365800	-3.05596400	-0.32715300
C	0.40782000	1.03622900	0.66603000
C	0.00881900	1.93943600	-0.33071900
C	-0.29014200	3.29632600	-0.04417200
C	-0.17839400	3.74899500	1.28324900
C	0.21850300	2.86213400	2.27631300
C	0.50957200	1.51999000	1.97101200
C	-1.57311200	-1.10381600	0.95693400
C	3.65279100	-2.34809000	-1.00737800
C	4.59635300	-1.35712800	-1.17317400
C	4.28514700	-0.01606500	-0.84213300
C	3.03854400	0.31043900	-0.35460100
H	-0.63397100	-3.50054400	0.29344400
H	1.62521700	-4.08289300	-0.58071900
H	-0.40360900	4.78453900	1.52384900
H	0.30572200	3.20403900	3.30360000
H	0.82142600	0.83983100	2.75875100
H	-1.80675900	-0.03838200	1.13326200
H	3.88647000	-3.38001400	-1.25755000
H	5.58276500	-1.60256000	-1.55696800
H	5.03470000	0.75903700	-0.97469700
H	2.80449000	1.33910900	-0.10198700
S	-0.20144300	1.58118800	-2.03507200
C	-0.68062200	3.23674900	-2.34406100
C	-0.68371000	4.01639600	-1.22902500
H	-0.95527200	5.06663600	-1.23051600
H	-0.93567800	3.53058100	-3.35423700
O	-2.42265100	-1.96000600	1.14683600

TS-4o

C	-3.05496400	0.41910500	-0.45205800

C	-1.97101300	-0.30696600	0.14487100
C	-0.62294400	0.24647500	0.10909800
C	-0.57244900	1.65578200	-0.00845400
C	-1.65569500	2.36311000	-0.60319400
C	-2.83048800	1.74053500	-0.92473600
C	0.55440500	-0.67439100	0.03593200
C	1.95238300	-0.34757700	0.03110700
C	2.95179500	-1.35982900	-0.11865800
C	2.59908900	-2.70602800	-0.28910800
C	1.25808900	-3.02459900	-0.39650400
C	0.28553100	-2.03063300	-0.26551400
C	0.38122400	2.52456600	0.71974400
C	-4.35570900	-0.14944100	-0.47587500
C	-4.62483200	-1.33880900	0.16421300
C	-3.59541700	-1.98734400	0.88246400
C	-2.30779900	-1.49146400	0.86195000
H	-1.51545200	3.42884700	-0.75372800
H	-3.63656200	2.27792100	-1.41685500
H	3.37377100	-3.46003100	-0.39752000
H	0.94542100	-4.04209300	-0.61242500
H	-0.73309800	-2.32726500	-0.45666000
H	0.90770700	2.03835700	1.55870000
H	-5.15007500	0.40344300	-0.97098800
H	-5.62972300	-1.75150500	0.15854600
H	-3.81889500	-2.87391100	1.46932500
H	-1.54601300	-1.98357700	1.45503400
S	2.75461900	1.22328200	0.04880500
C	4.33434200	0.51241100	-0.11689900
C	4.29248900	-0.84105500	-0.16970400
H	5.16702000	-1.47516900	-0.26721200
H	5.19976600	1.16155100	-0.15272100
O	0.47752400	3.73184900	0.55989100

5oI

C	2.33489200	-2.21190400	-0.25048100
C	1.99446600	-0.89934400	0.21242900
C	0.61596400	-0.51518600	0.27146900
C	-0.37392200	-1.40809500	-0.13361100
C	-0.01820000	-2.70696100	-0.59662100
C	1.29419200	-3.09646400	-0.64853800
C	0.26387500	0.85494900	0.74586200
C	-0.06524600	1.86894500	-0.16644400
C	-0.39325900	3.18522200	0.24931000
C	-0.38411600	3.48292700	1.62423800

C	-0.05661800	2.48662700	2.53599000
C	0.26552800	1.18807500	2.10147600
C	-1.79463800	-1.02608900	-0.07844400
C	3.70028600	-2.59370400	-0.30640500
C	4.69661300	-1.71741500	0.06767700
C	4.36403100	-0.41540300	0.51199800
C	3.04626900	-0.01625400	0.58248600
N	-2.71974700	-1.78570500	-0.52241300
C	-4.08822000	-1.28514300	-0.41021100
C	-4.90622800	-2.15317300	0.44235400
C	-5.58236700	-2.86112200	1.15160400
H	-0.81889400	-3.37335900	-0.89829900
H	1.55892500	-4.09153900	-0.99807400
H	-0.63264700	4.48480700	1.96421000
H	-0.04896700	2.70824300	3.59943400
H	0.52056900	0.42094300	2.82729700
H	-2.02086200	-0.04866700	0.36733600
H	3.94727300	-3.59366900	-0.65498800
H	5.73850700	-2.02180100	0.01840900
H	5.15363300	0.27453300	0.79677300
H	2.79941000	0.98451300	0.92021600
H	-4.12432600	-0.25211000	-0.02185100
H	-4.52105400	-1.26789400	-1.41974200
H	-6.17398300	-3.49213300	1.77647300
S	-0.14100700	1.70921200	-1.91266400
C	-0.60373300	3.39119400	-2.06572300
C	-0.69792300	4.03786600	-0.87225100
H	-0.97349500	5.08258000	-0.77439600
H	-0.77962100	3.80081300	-3.05225300

TS-5oI

C	2.75136000	-1.66118600	-0.59621600
C	2.24853300	-0.51347300	0.10073900
C	0.84765900	-0.12840100	-0.04983800
C	-0.01399500	-1.18982600	-0.40416600
C	0.49885400	-2.32633500	-1.09385300
C	1.84073000	-2.50493900	-1.28742900
C	0.46394800	1.31842500	0.04437500
C	-0.83773300	1.92571800	-0.01632300
C	-0.99901400	3.34383900	0.08695800
C	0.10852800	4.19599900	0.20187500
C	1.37082900	3.64018600	0.11828400
C	1.52366500	2.25563700	0.01598400
C	-1.38941500	-1.32776800	0.09789100

C	4.12666600	-2.00083200	-0.50264300
C	4.97392400	-1.31675300	0.34060600
C	4.45359700	-0.28245500	1.14959100
C	3.13626100	0.11083100	1.02423700
N	-2.23183800	-2.14897600	-0.40002800
C	-3.50916700	-2.27075800	0.29591100
C	-3.75184500	-3.64795800	0.73572600
C	-3.95224700	-4.78058000	1.10756000
H	-0.22700100	-3.05735900	-1.43395400
H	2.21848000	-3.35363500	-1.85136500
H	-0.03904000	5.26942200	0.28157900
H	2.25583400	4.26991700	0.10848100
H	2.52818300	1.89393900	-0.13191800
H	-1.60869300	-0.78262900	1.02431800
H	4.48635100	-2.85116900	-1.07684800
H	6.01950900	-1.60052800	0.42093200
H	5.08732800	0.19873100	1.88951300
H	2.75425100	0.87717300	1.68821300
H	-3.58057200	-1.59209600	1.16362000
H	-4.29956000	-1.97703600	-0.40983200
H	-4.12353700	-5.78270100	1.43172400
S	-2.42300500	1.22050700	-0.36886300
C	-3.21773300	2.76824100	-0.31385800
C	-2.36210100	3.78391800	-0.04854800
H	-2.65372500	4.82622600	0.02312700
H	-4.28554500	2.81601100	-0.48569600

Molecular dynamics (MD) simulations

The dimeric structure of IR-09 was constructed based on its crystal structure (PDB: 8QHE), which is in complex with NADPH and the small molecule N-methylcyclohexanamine (VCU).[19] VCU was removed from the active site in order to model the imine intermediates of **3a** and **5h** (i.e., **3aI**, **5hI**, SI Figure 10) into the active site. The protonation states of residues were determined by propka3.0[20] and the ff14SB force field[21] was employed for the protein. Parameters for the small molecules were generated within the Antechamber module in AMBER package using the general AMBER force field[22] (GAFF2), with partial charges set to fit the electrostatic potential generated at the HF/6-31G(d) level by the RESP model. The complex was solvated in a periodic rectangular prism box of TIP3P[23] water with the surface at least 8 Å away from the complex and neutralized with Na^+ counterions. Molecular dynamics (MD) simulations were carried out using the AMBER19 package with the PMEMD code.[24] First, the solvent was relaxed and the system was optimized by energy minimization via the steepest descent and conjugate gradient methods. Then, the system was heated from 0 to 310 K under the NVT ensemble for 100 ps, followed by another 100 ps of NPT ensemble MD simulations at 310K and 1.0 atm. Finally, NVT MD simulation was performed under the periodic boundary condition. The SHAKE algorithm[25] was applied to constrain all hydrogen-containing bonds during MD simulation. Both van der Waals (vdW) and electrostatic interactions were truncated at a cutoff of 10 Å. The generated trajectories were analyzed using the Cpptraj module implemented in the AMBER19 package.[26]

We initially attempted to dock the imine intermediates into the active site using AutoDock Vina[27] but failed due to the tight pocket induced by the small volume of the co-crystallized substrate (VCU). Consequently, we retained the molecular skeleton of **3aI** and **5hI**, and designed smaller molecules without chirality, namely **3aD** and **5hD** (SI Figure 10). Both **3aD** and **5hD** were successfully docked into the active site. Subsequently, 100 ns MD simulations were performed on the resulting complexes. The generated trajectories were clustered, and representative conformations were obtained. After removing **3aD** and **5hD** from the binding pocket, the resulting structures were used for docking **3aI** and **5hI** (SI Figure 10). The generated enzyme complexes with either the (*S*)- or (*R*)-atropisomer were proceeded with 200-250 ns MD simulations, with the exact duration determined based on RMSD stabilization. The last 100 ns of the MD trajectories were used

for further analysis (SI Figure 11).

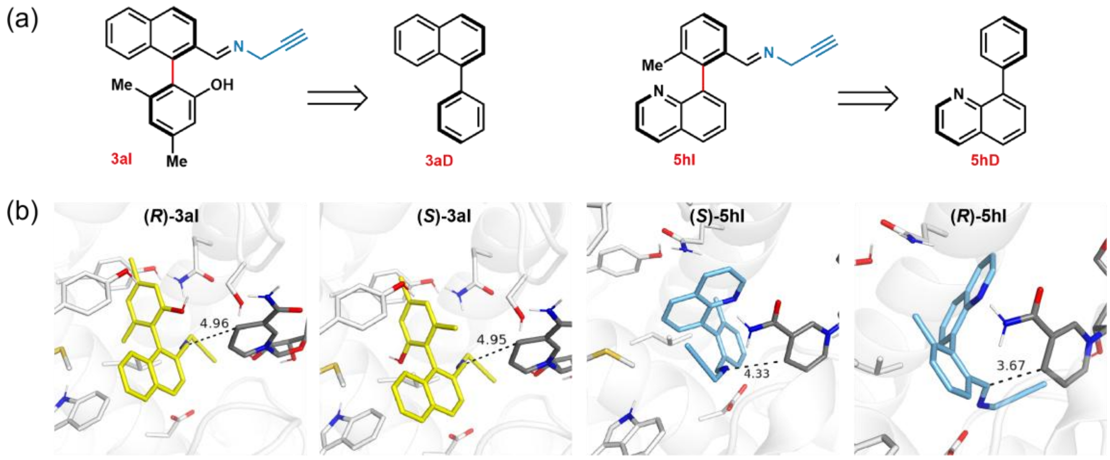

SI Figure 10. Molecular docking. (a) Chemical structures of **3aI**, **3aD**, **5hI** and **5hD**. (b) Docking poses of the (*S*)- and (*R*)-atropisomers of **3aI** and **5hI** within the IR-09 complex.

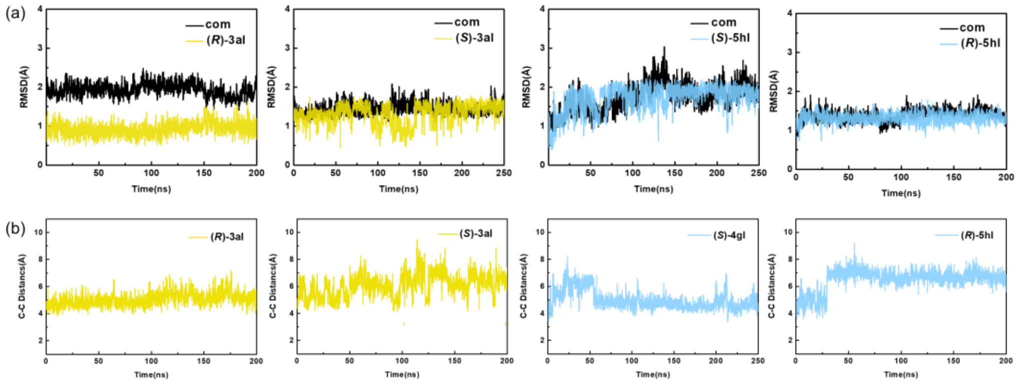

SI Figure 11. Evolution of RMSD values and catalytic distance during MD simulations. The distances measured are between the carbon atom of the C=N group of the imine intermediate and the C4 atom of the nicotinamide ring of NADPH.

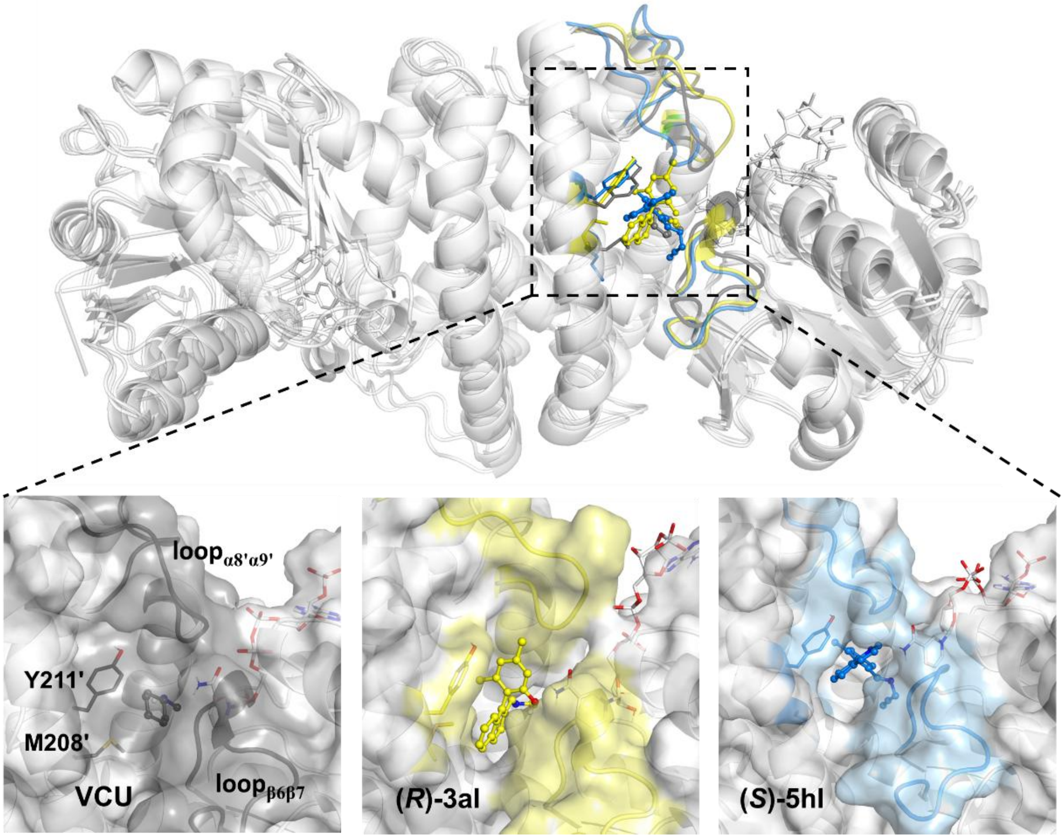

SI Figure 12. Comparison of the IR-09 active pocket with different substrates bound. The loop$_{\alpha8'\alpha8'}$ and loop$_{\beta6'\beta7'}$ are colored in gray, yellow and blue for the IR-09 complex with VCU, (*R*)-**3aI** and (*S*)-**5hI**, respectively.

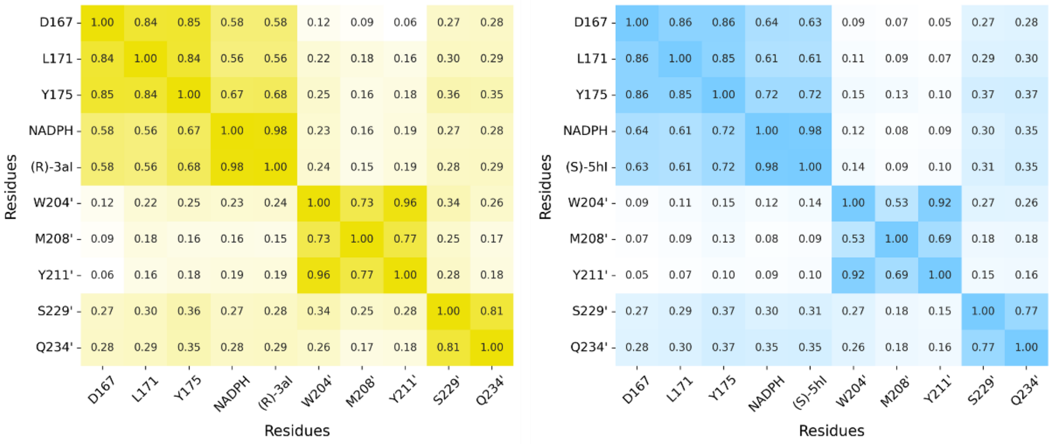

SI Figure 13. Dynamic cross-correlation matrix (DCCM). The values were calculated based on the positional fluctuations of heavy atoms throughout the MD simulation.

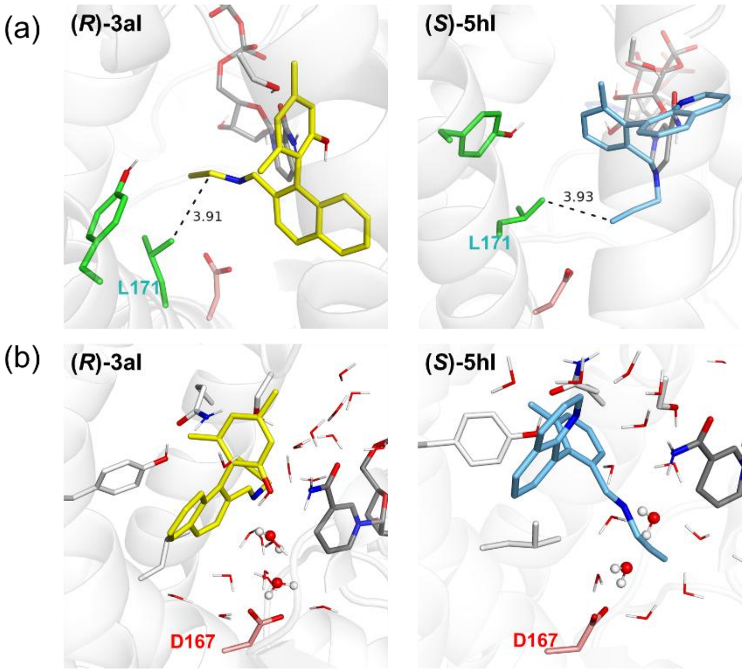

SI Figure 14. Detailed view of the IR-09 active site.

3.NMR Spectra

^{1}H NMR (400 MHz, $CDCl_3$) of **4b**

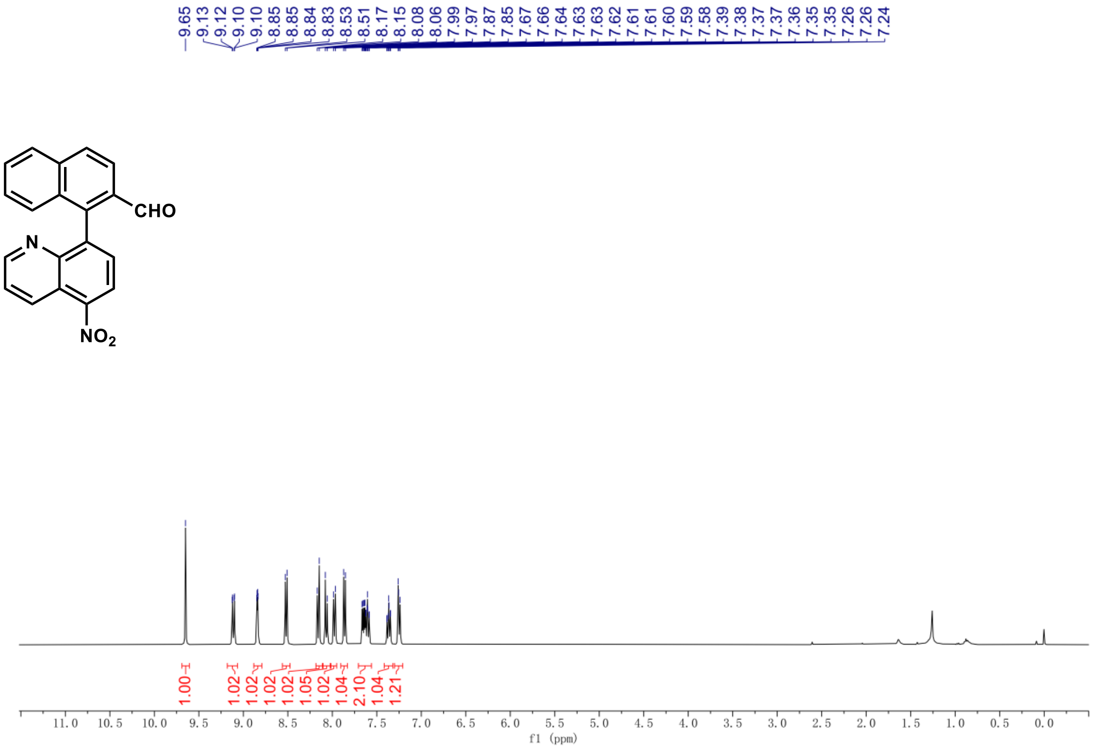

^{13}C NMR (101 MHz, $CDCl_3$) of **4b**

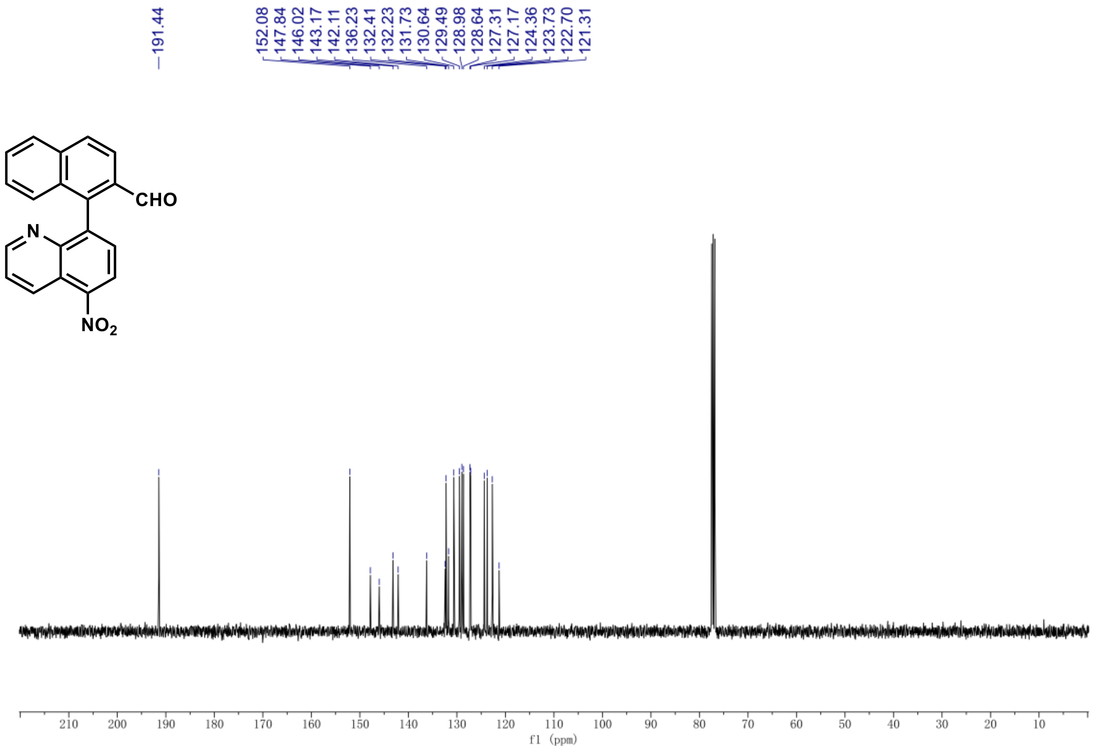

^{1}H NMR (400 MHz, $CDCl_3$) of **4d**

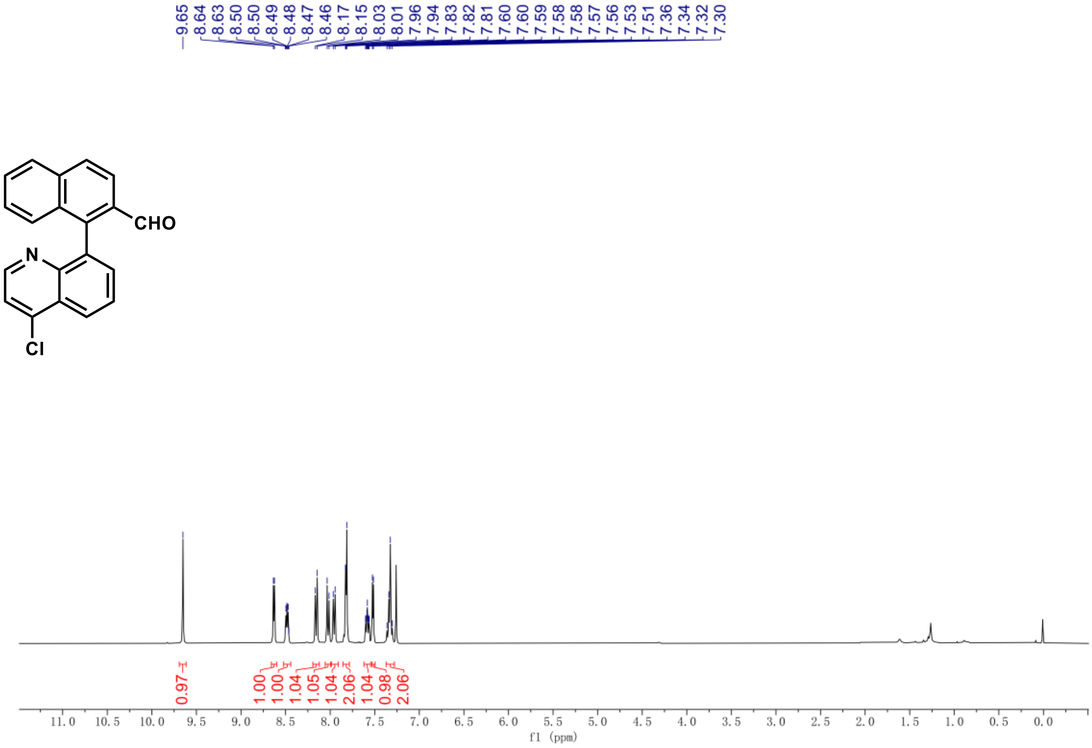

^{13}C NMR (101 MHz, $CDCl_3$) of **4d**

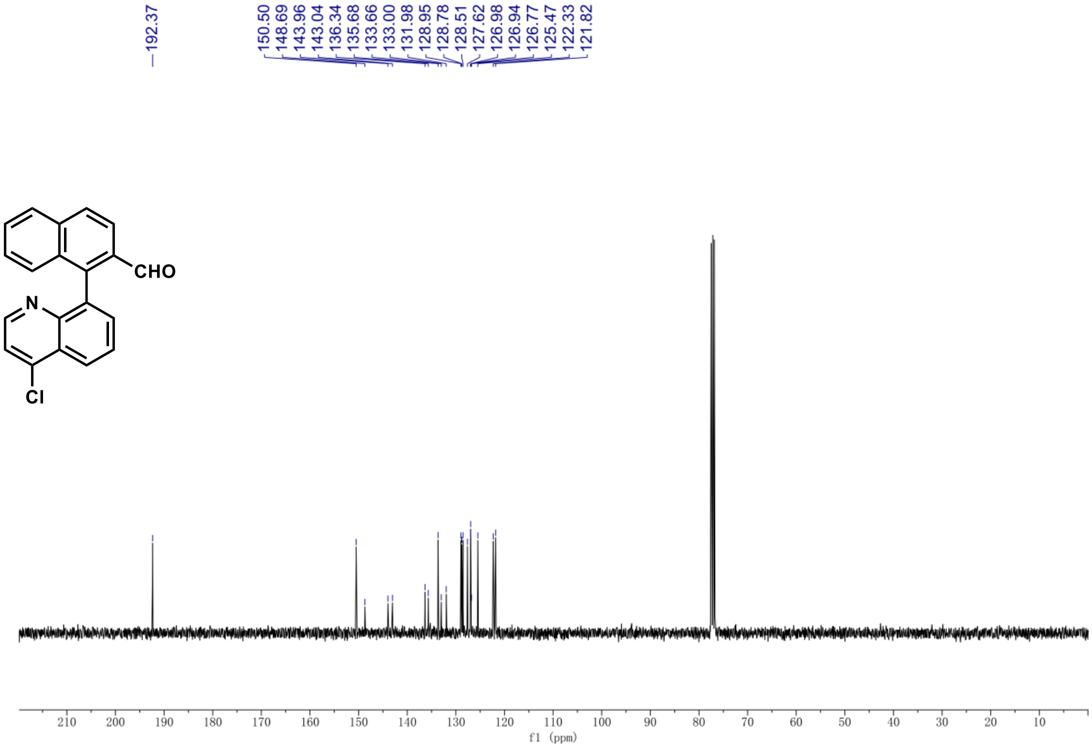

^{1}H NMR (400 MHz, $CDCl_3$) of **4f**

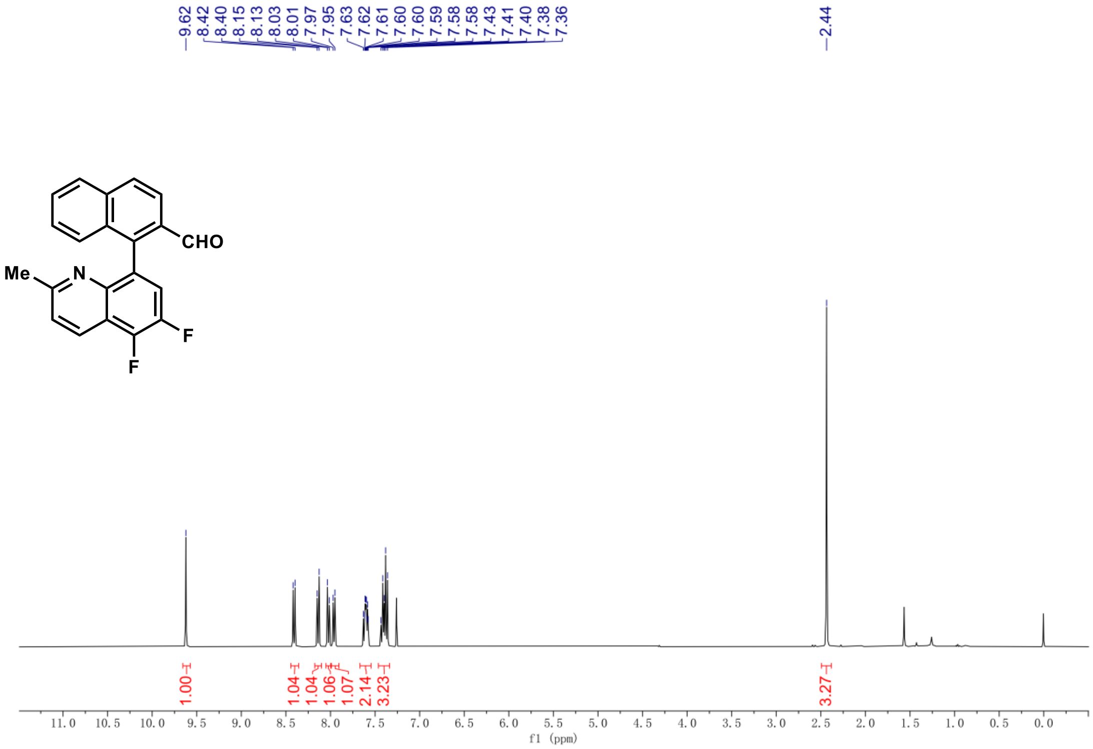

^{13}C NMR (126 MHz, $CDCl_3$) of **4f**

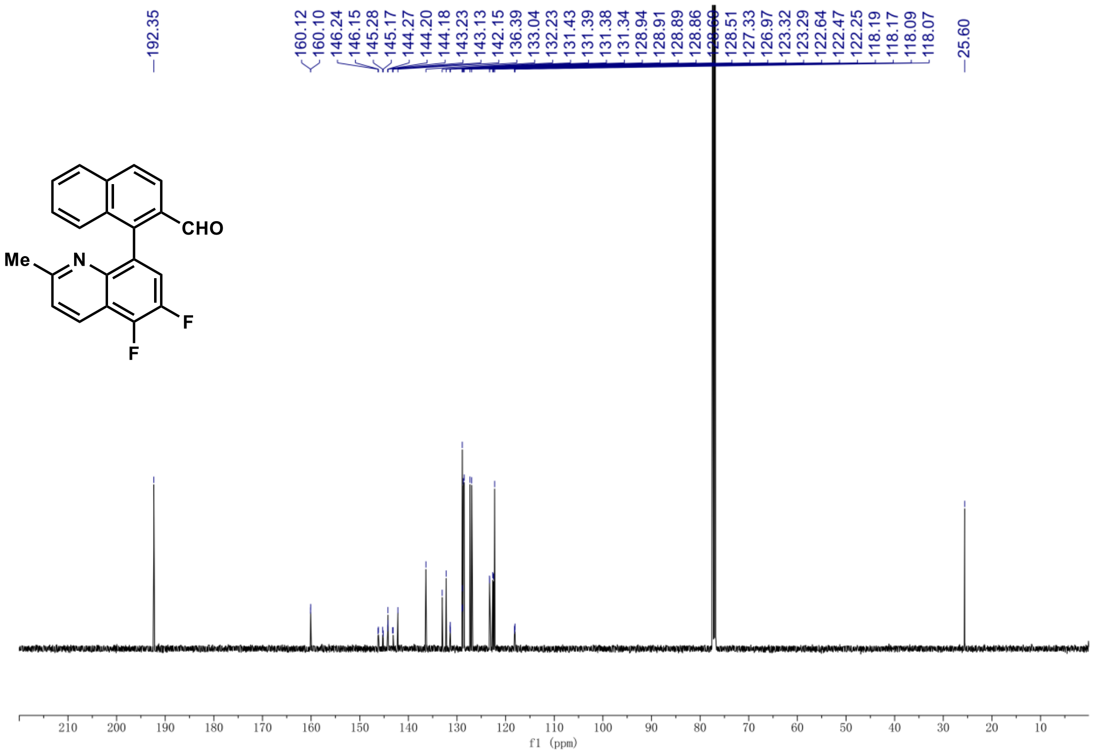

^{19}F NMR (470 MHz, $CDCl_3$) of **4f**

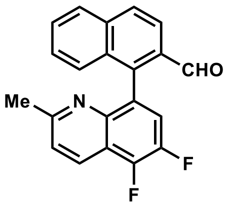

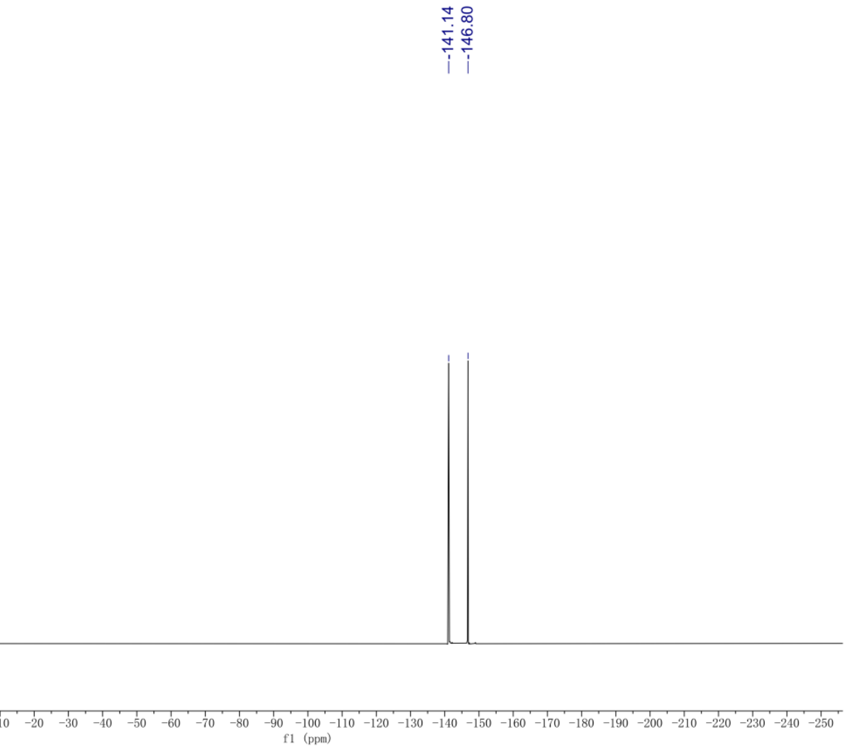

^{1}H NMR (400 MHz, $CDCl_3$) of **4g**

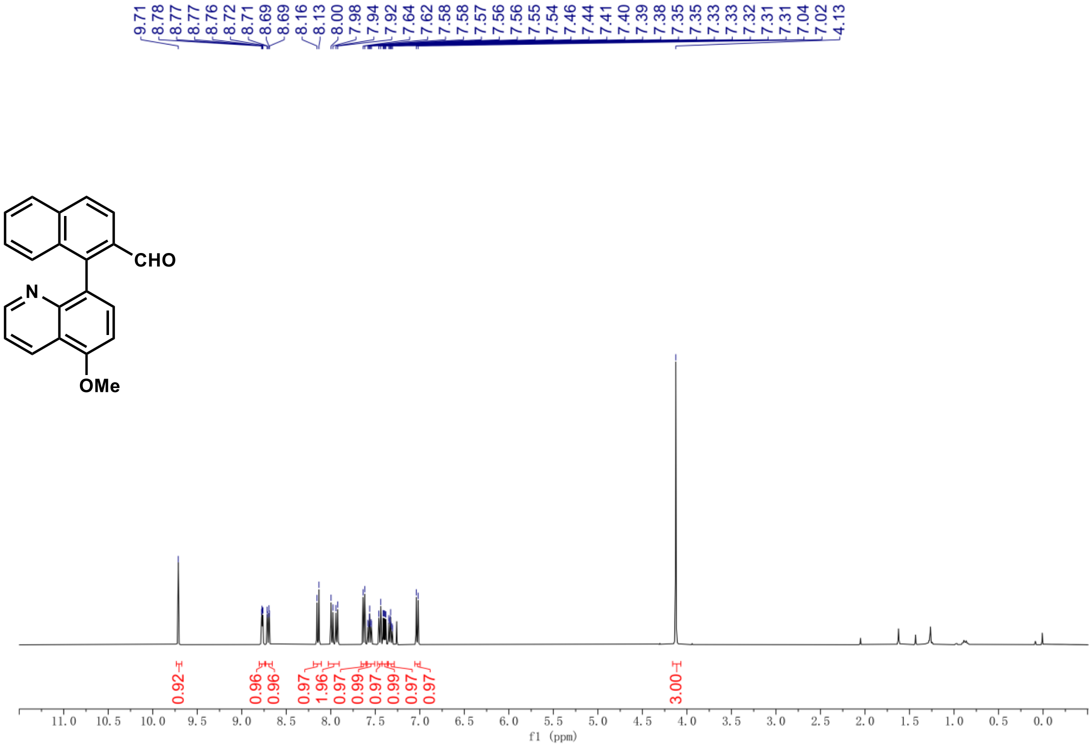

^{13}C NMR (101 MHz, $CDCl_3$) of **4g**

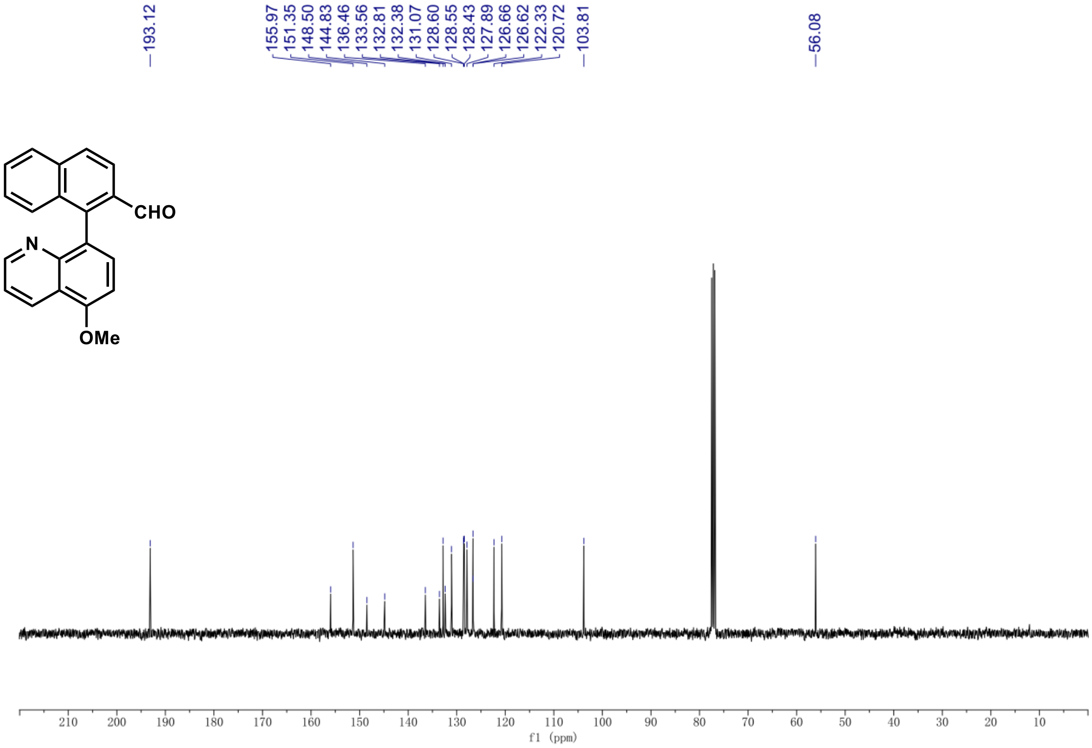

^{1}H NMR (400 MHz, CDCl$_3$) of **4k**

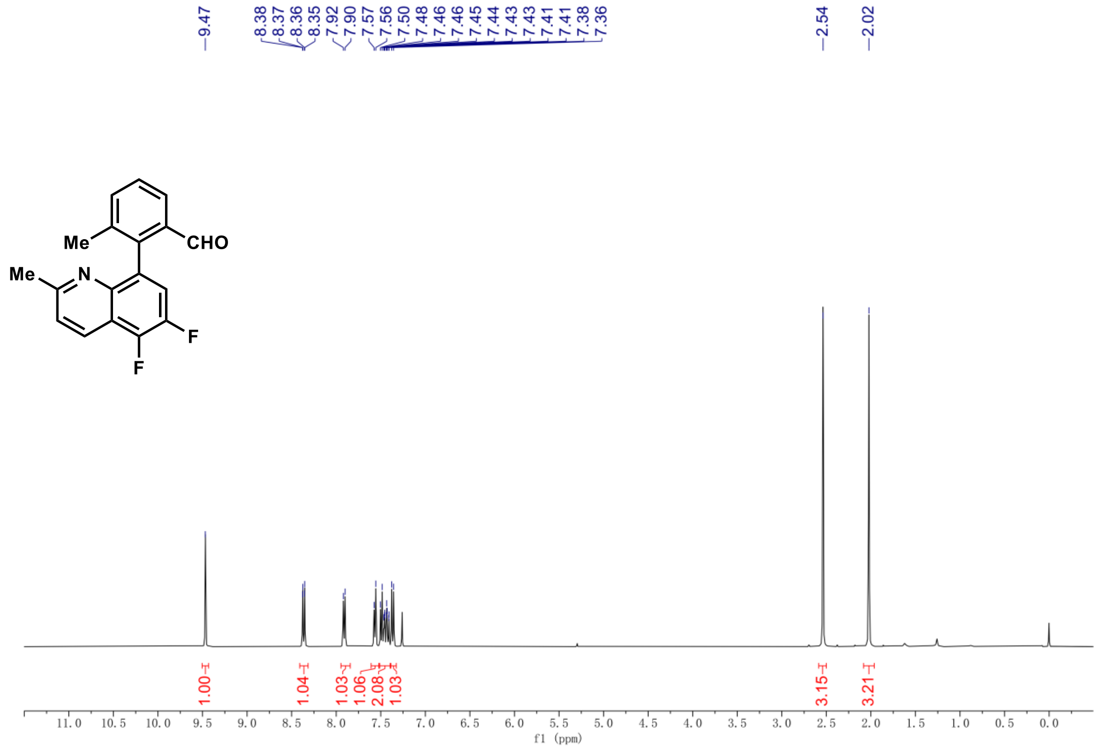

^{13}C NMR (126 MHz, CDCl$_3$) of **4k**

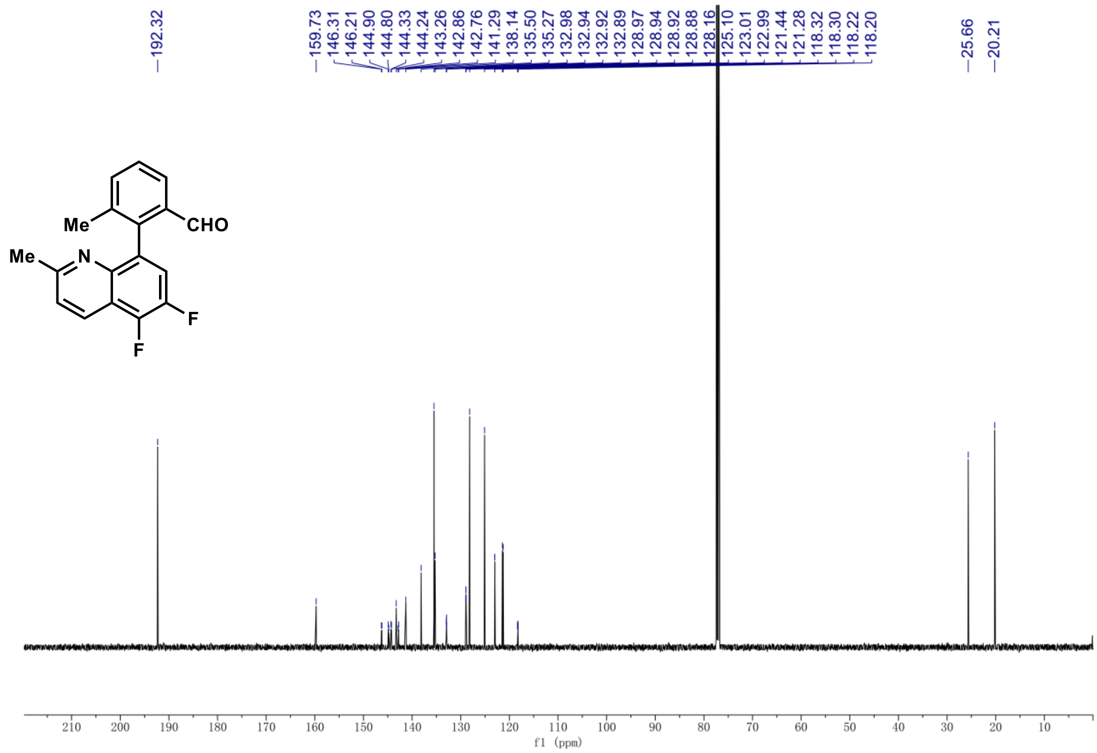

^{19}F NMR (470 MHz, $CDCl_3$) of **4k**

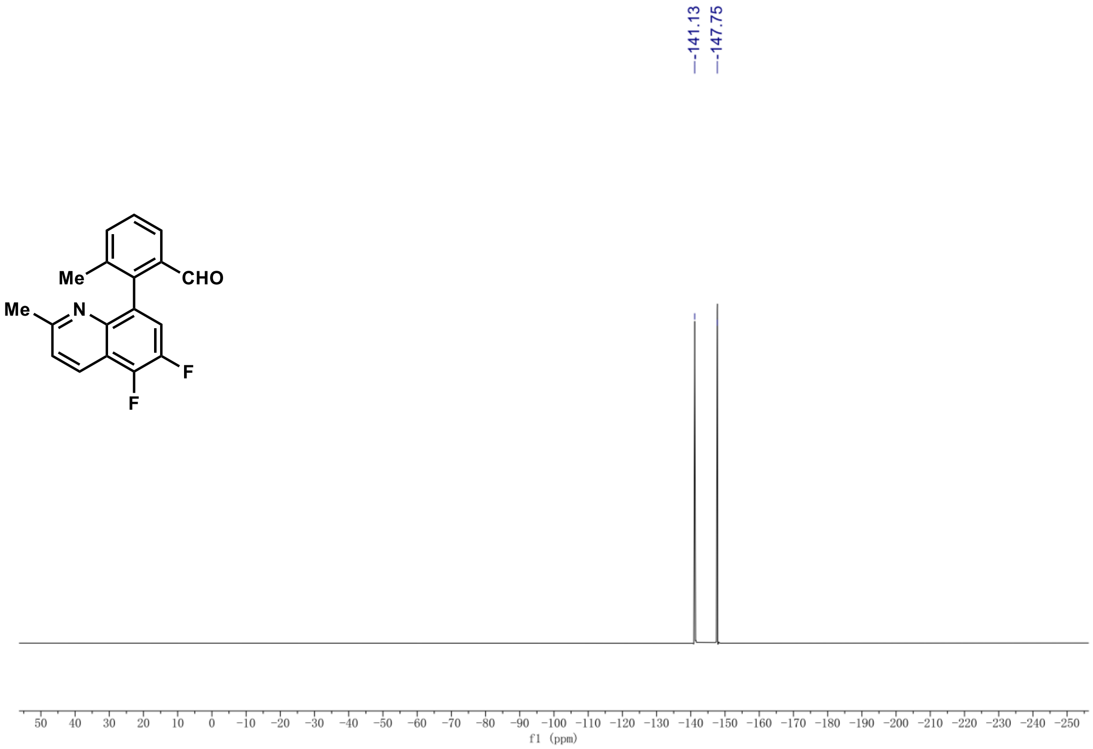

^{1}H NMR (400 MHz, $CDCl_3$) of **4m**

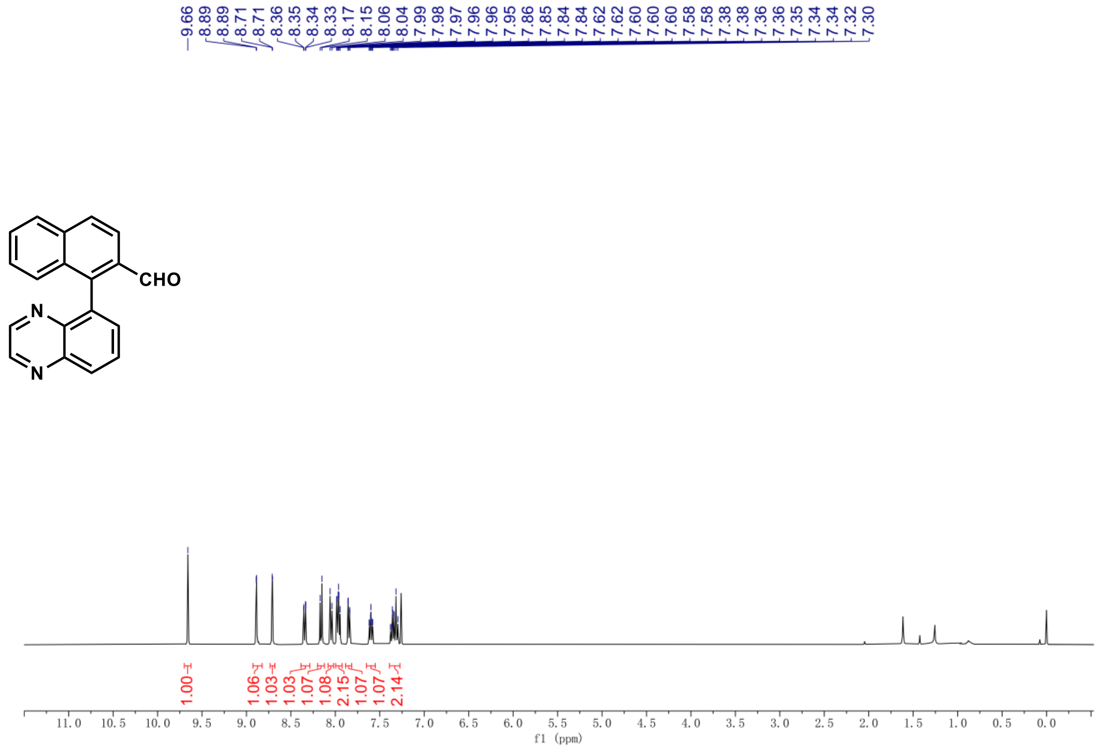

^{13}C NMR (101 MHz, $CDCl_3$) of **4m**

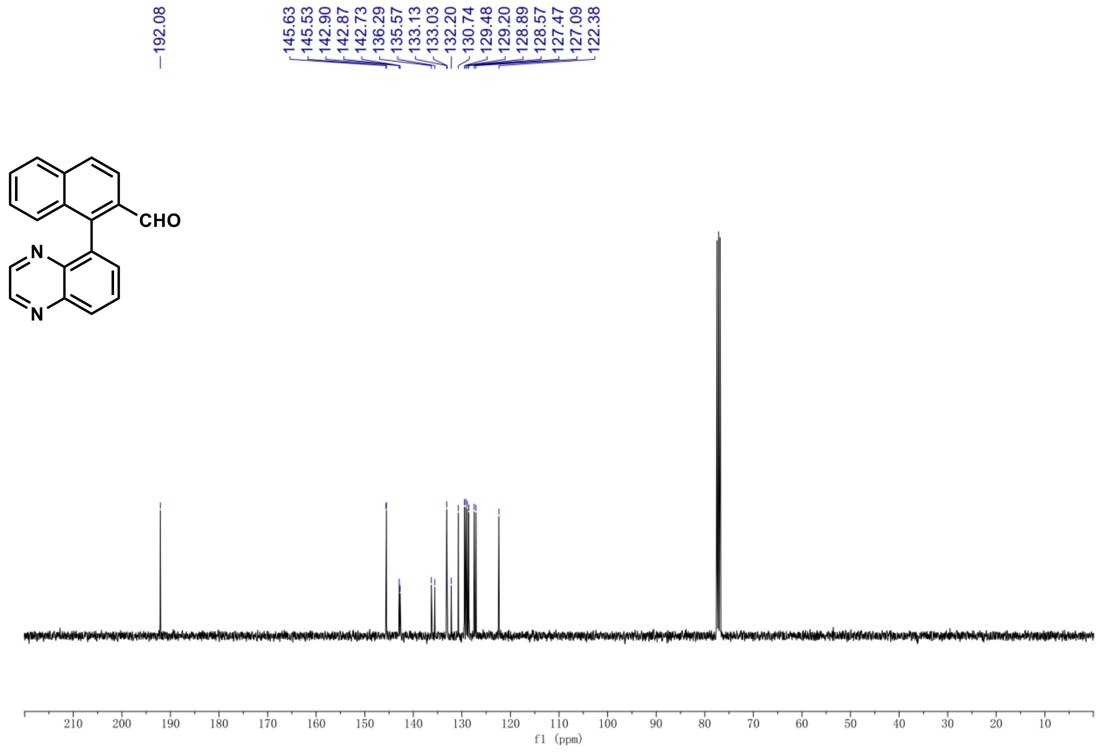

^{1}H NMR (400 MHz, $CDCl_3$) of **4n**

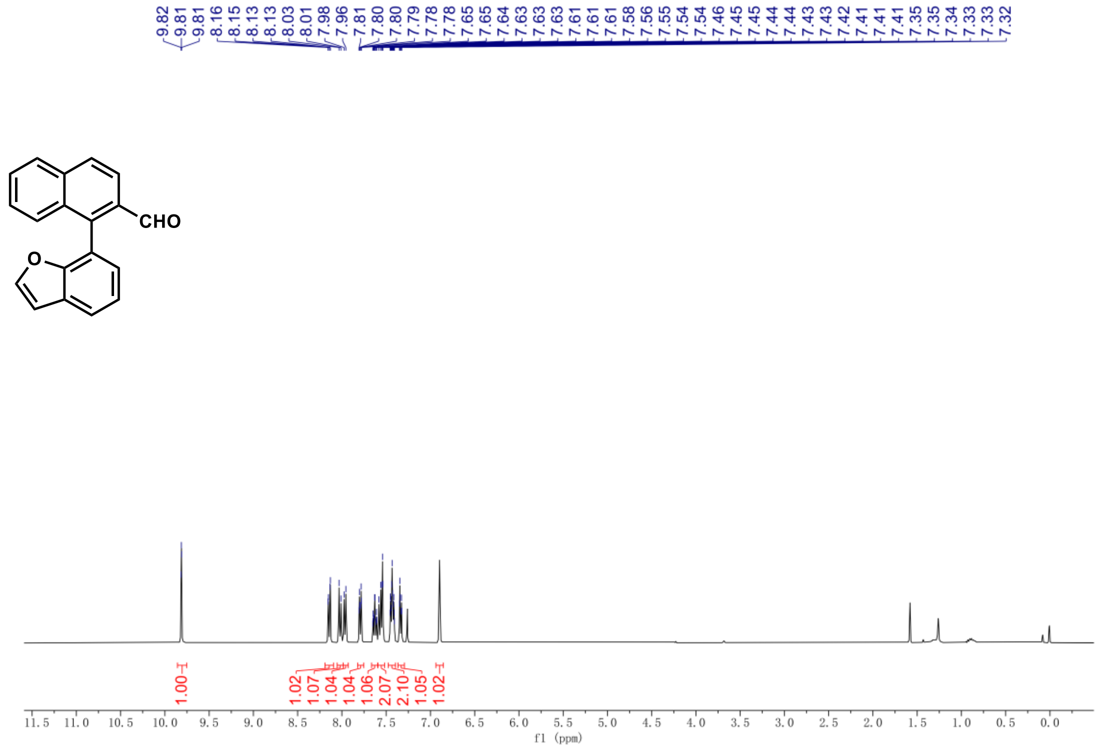

^{13}C NMR (101 MHz, $CDCl_3$) of **4n**

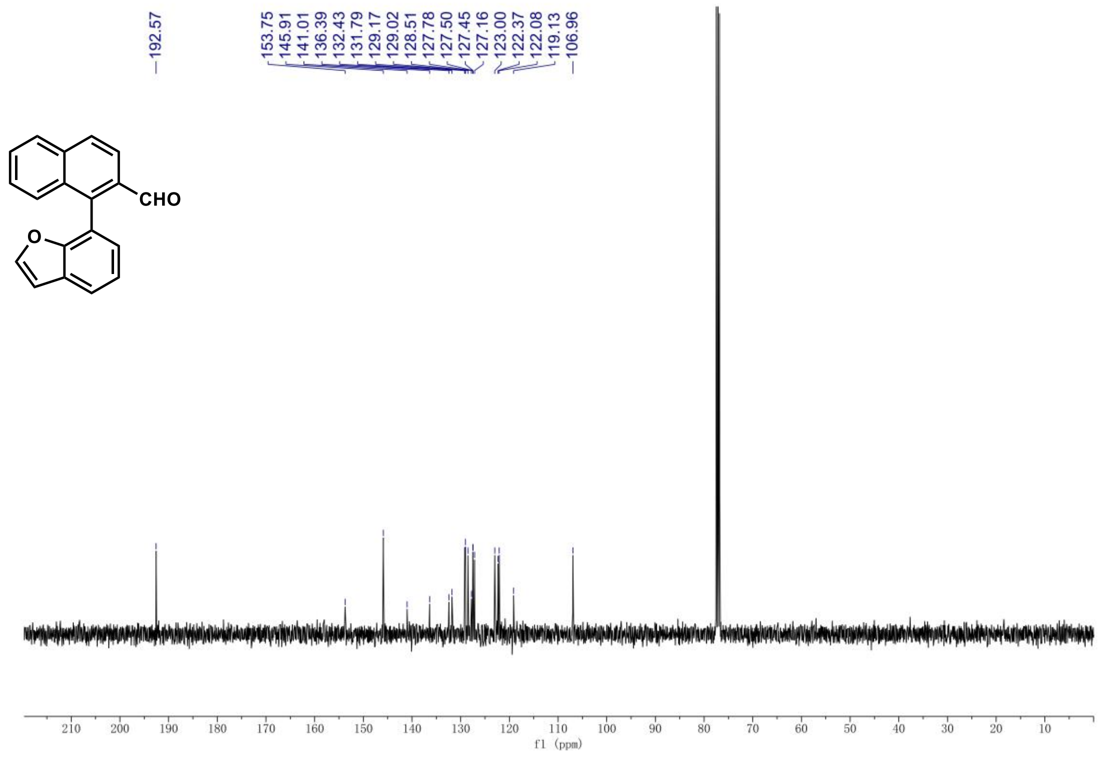

^{1}H NMR (400 MHz, $CDCl_3$) of **4o**

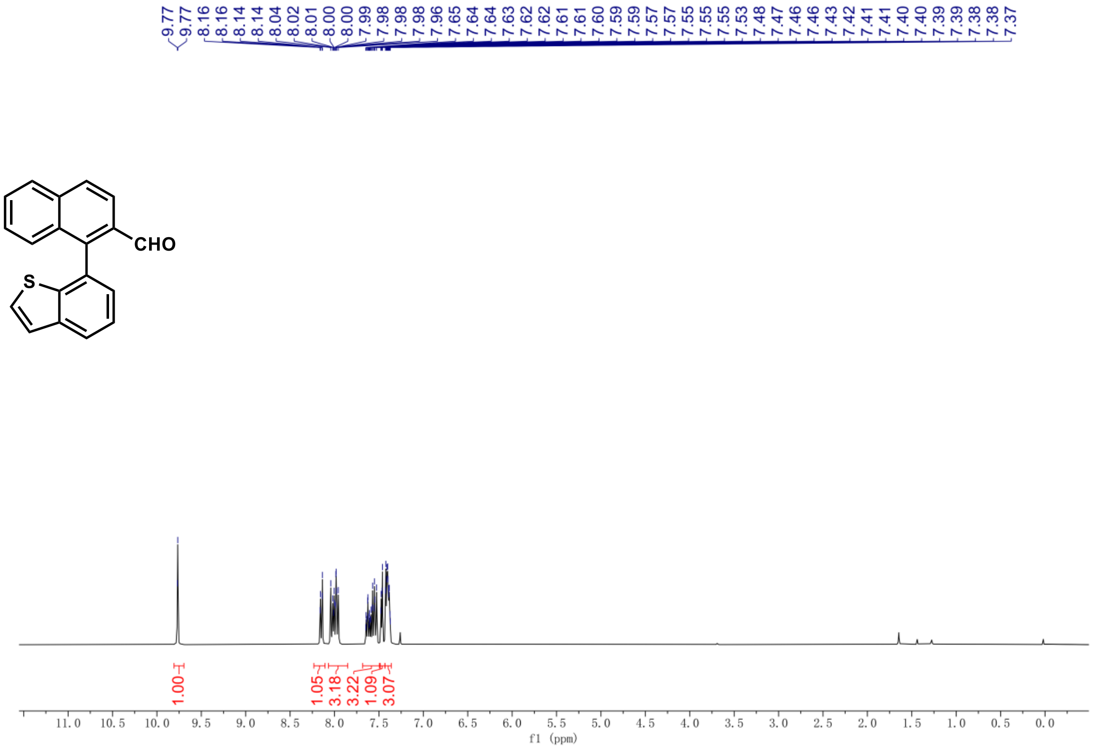

^{13}C NMR (101 MHz, $CDCl_3$) of **4o**

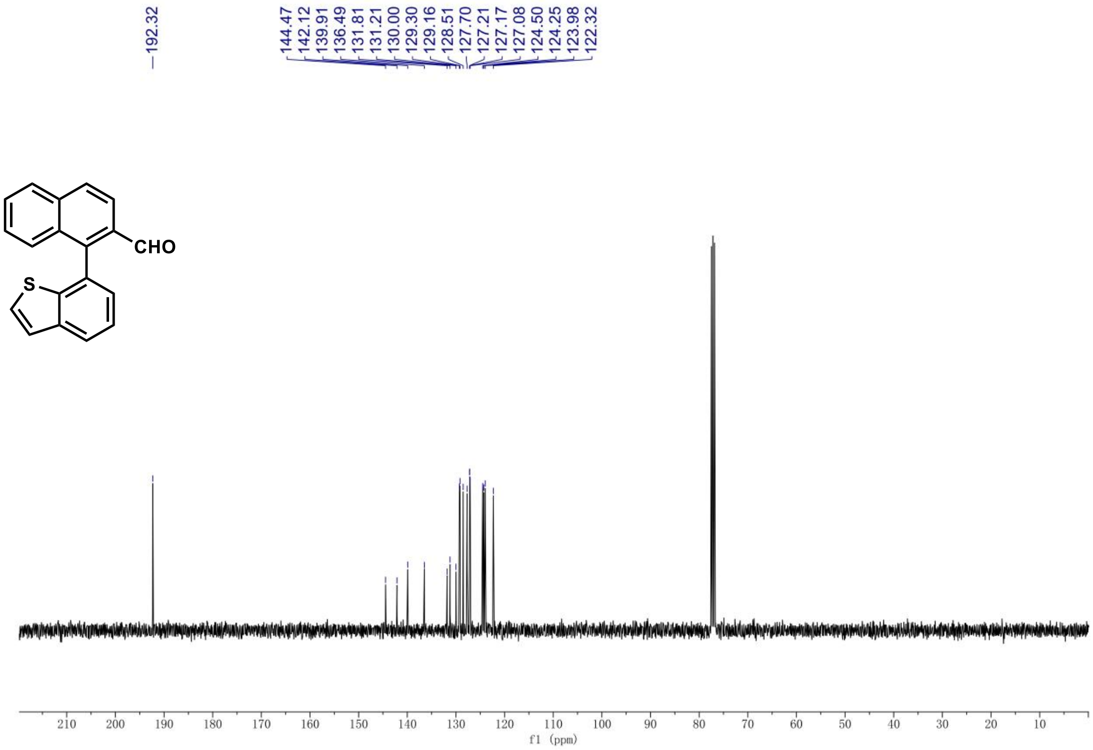

^1H NMR (400 MHz, $CDCl_3$) of **4p**

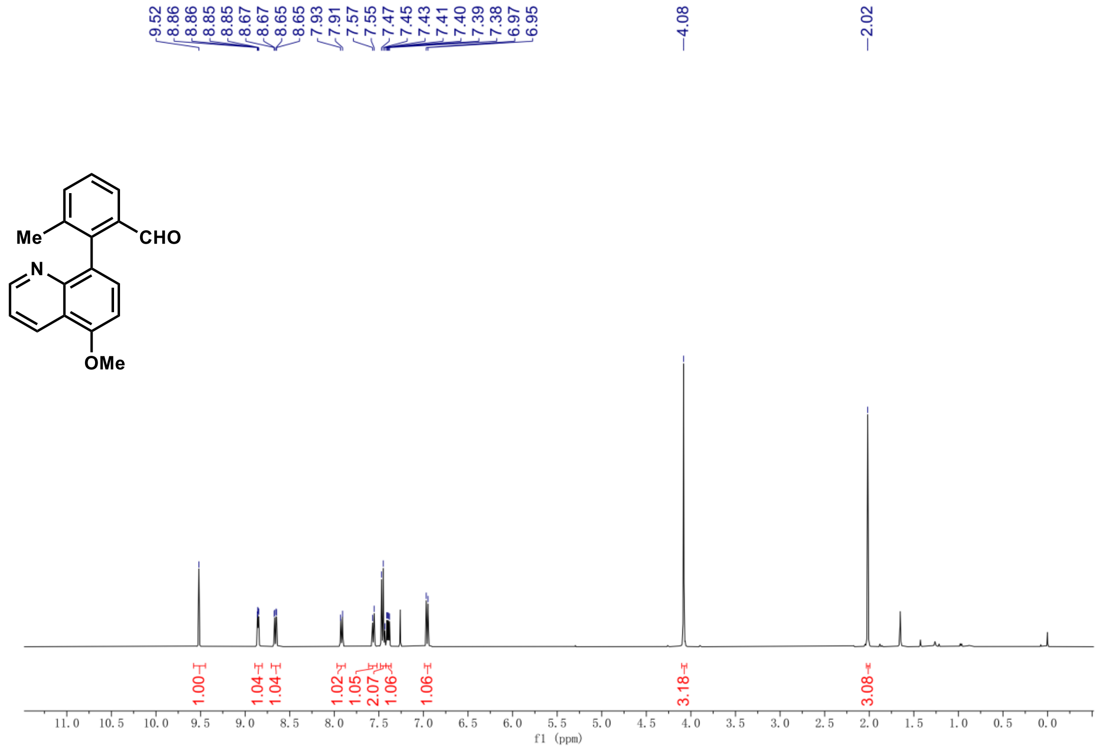

^{13}C NMR (101 MHz, $CDCl_3$) of **4p**

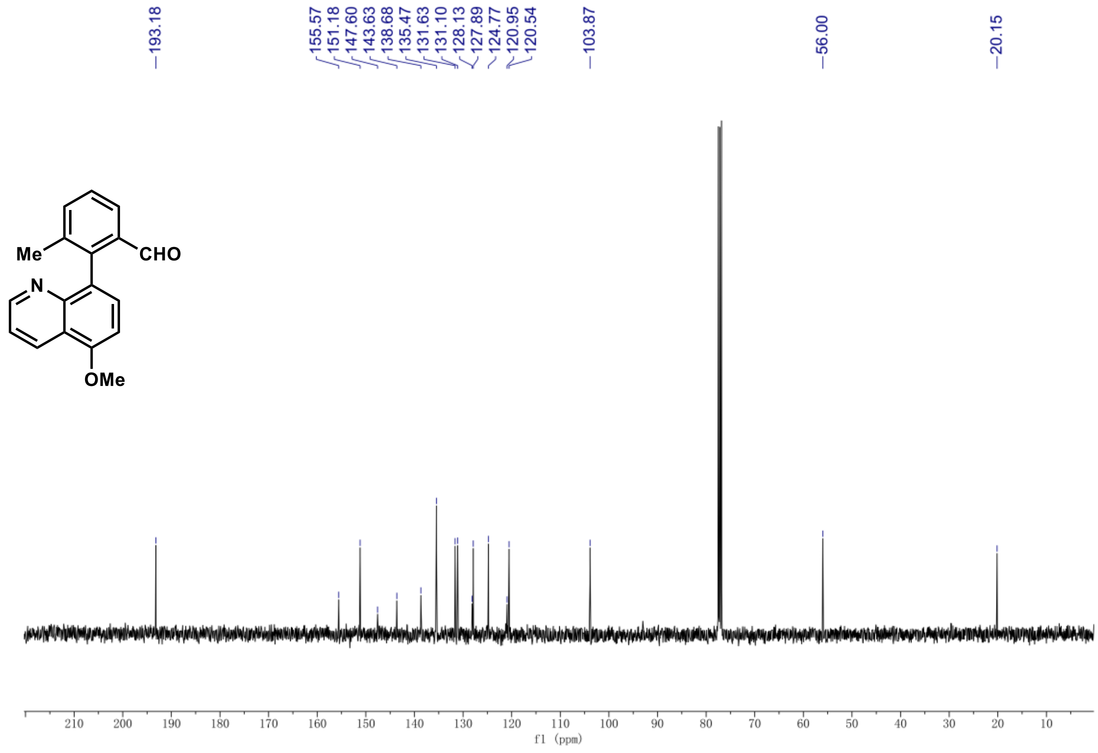

^{1}H NMR (400 MHz, $CDCl_3$) of **3a**

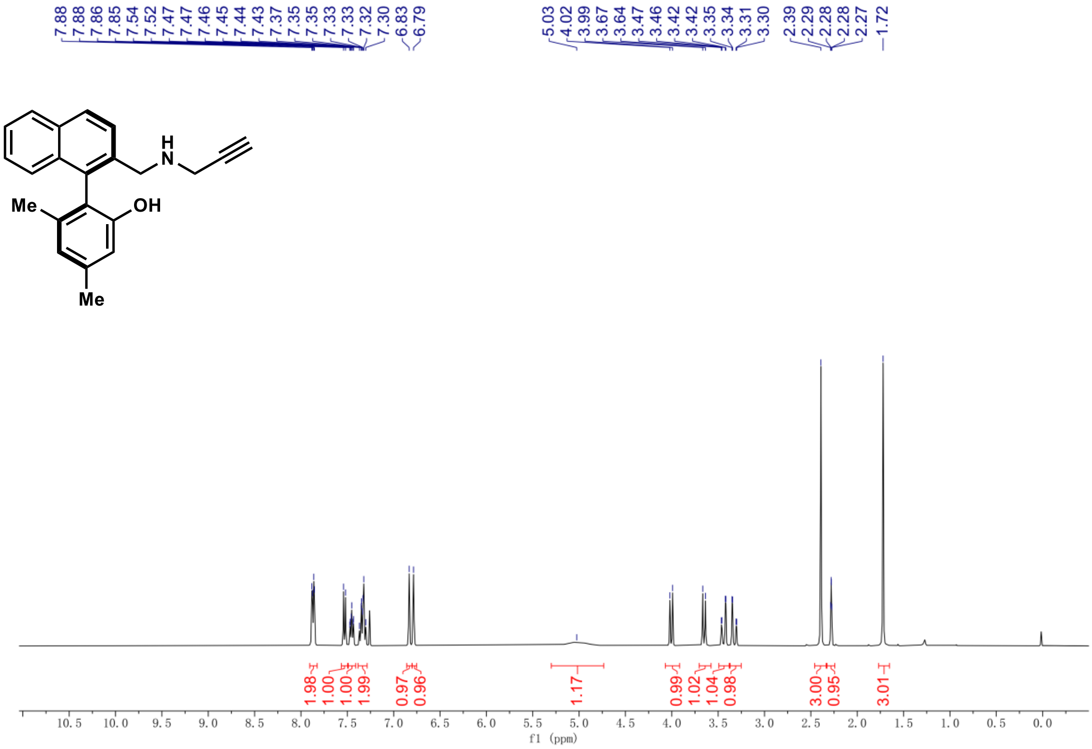

^{13}C NMR (101 MHz, $CDCl_3$) of **3a**

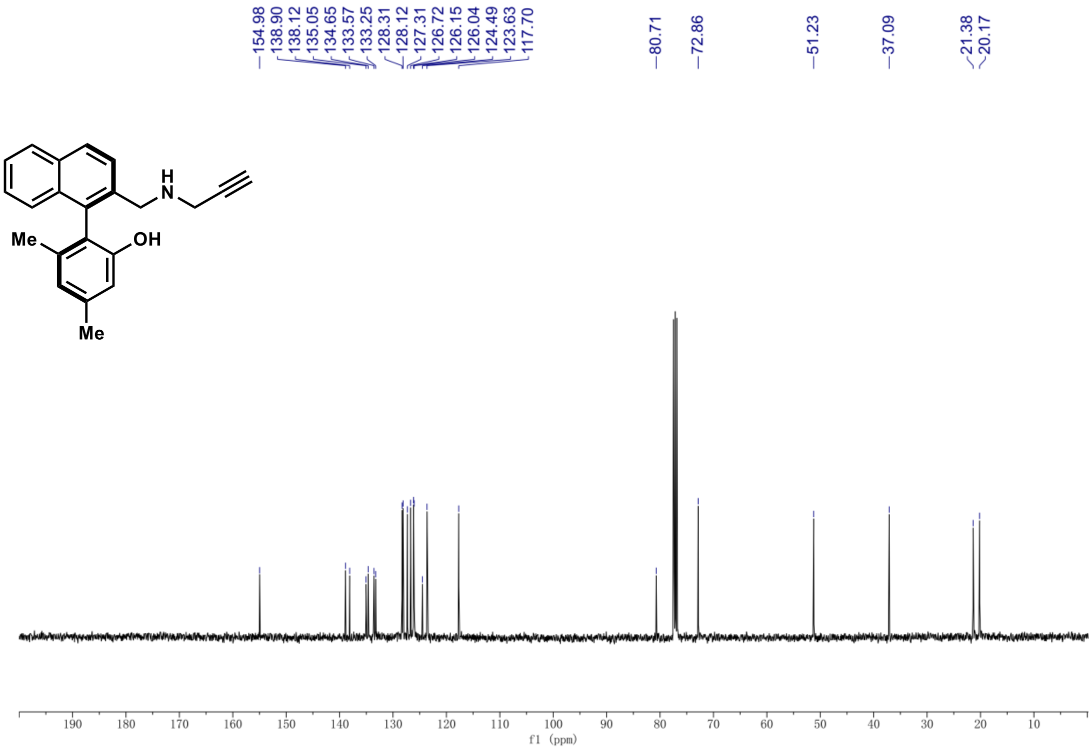

^{1}H NMR (400 MHz, $CDCl_3$) of **3b**

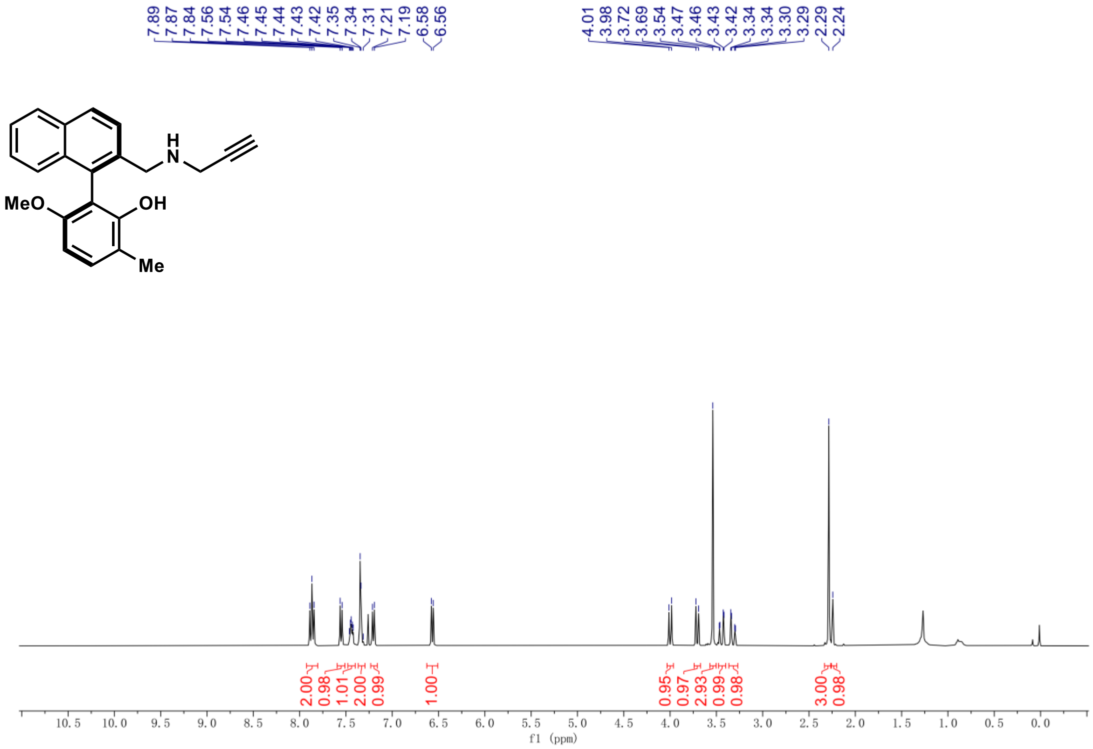

^{13}C NMR (101 MHz, $CDCl_3$) of **3b**

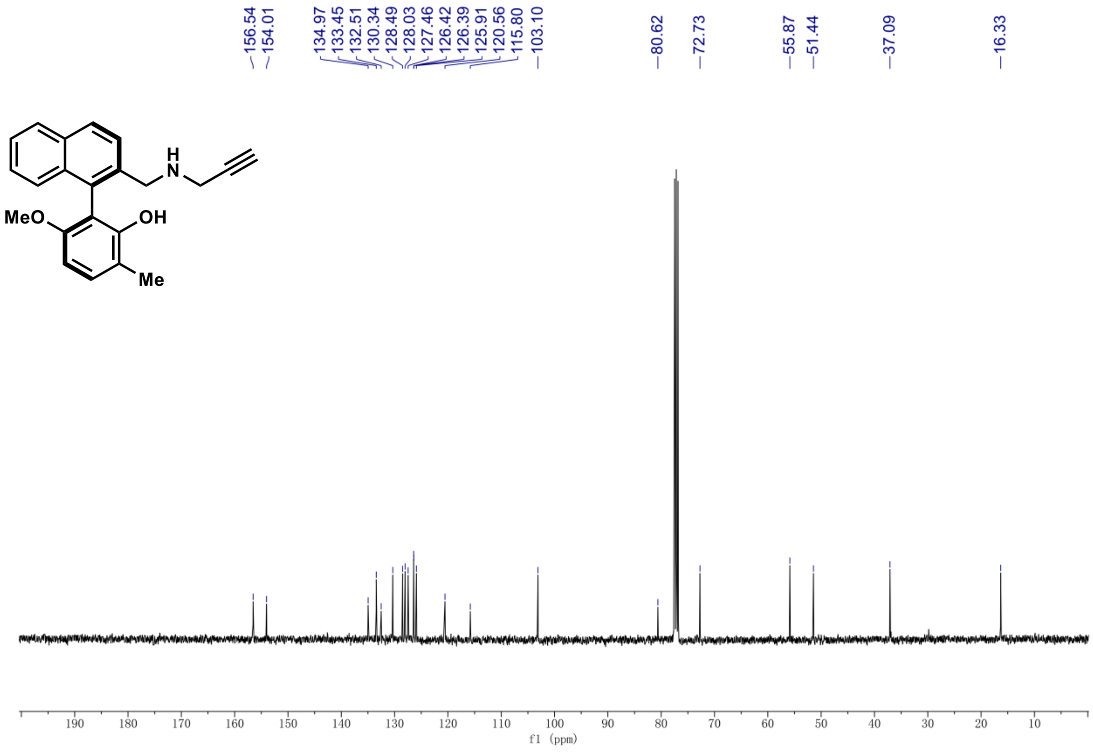

^{1}H NMR (400 MHz, $CDCl_3$) of **3c**

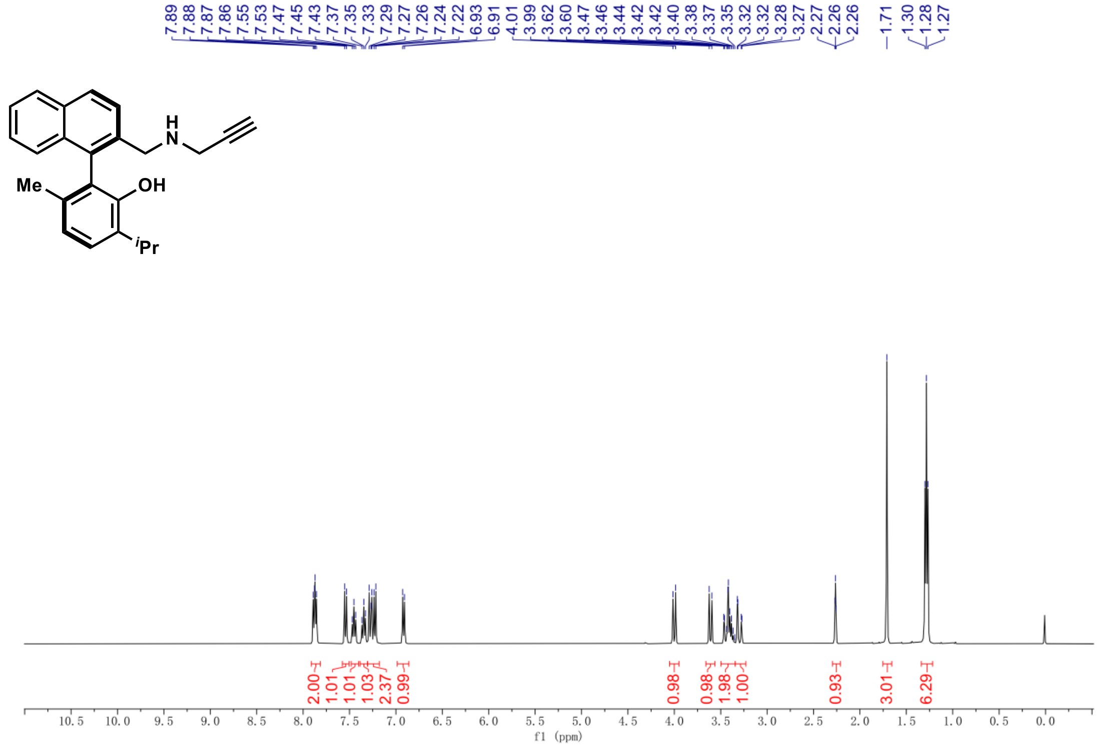

^{13}C NMR (101 MHz, $CDCl_3$) of **3c**

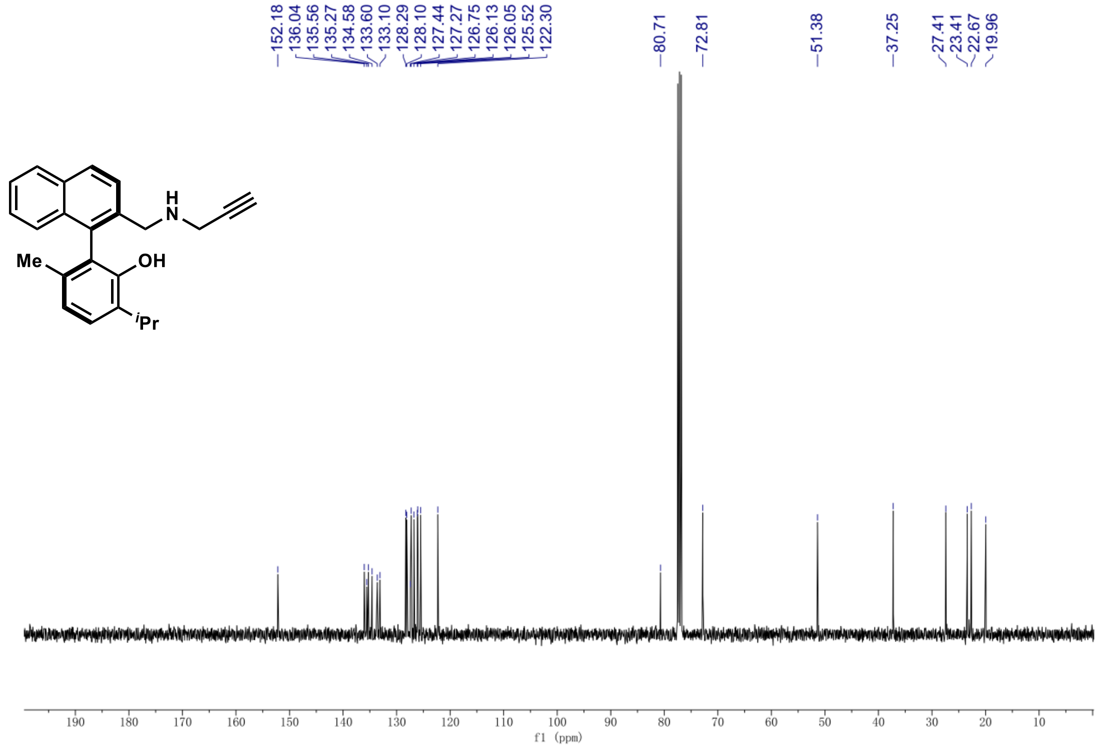

^{1}H NMR (400 MHz, $CDCl_3$) of **3d**

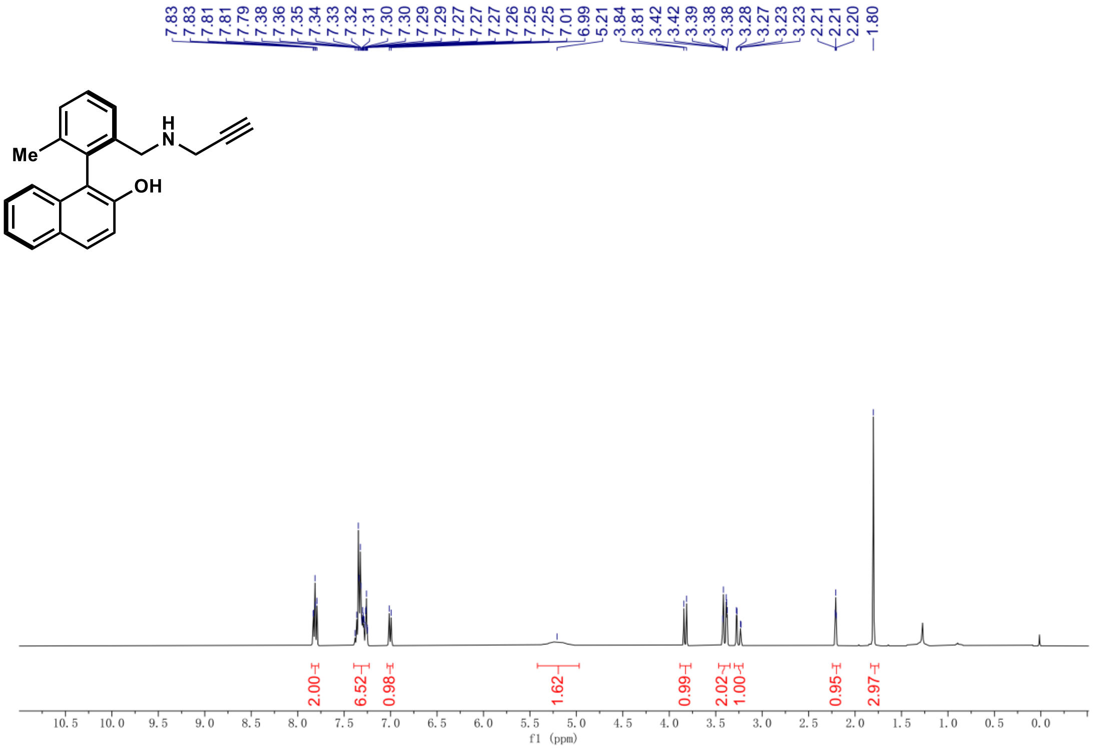

^{13}C NMR (101 MHz, $CDCl_3$) of **3d**

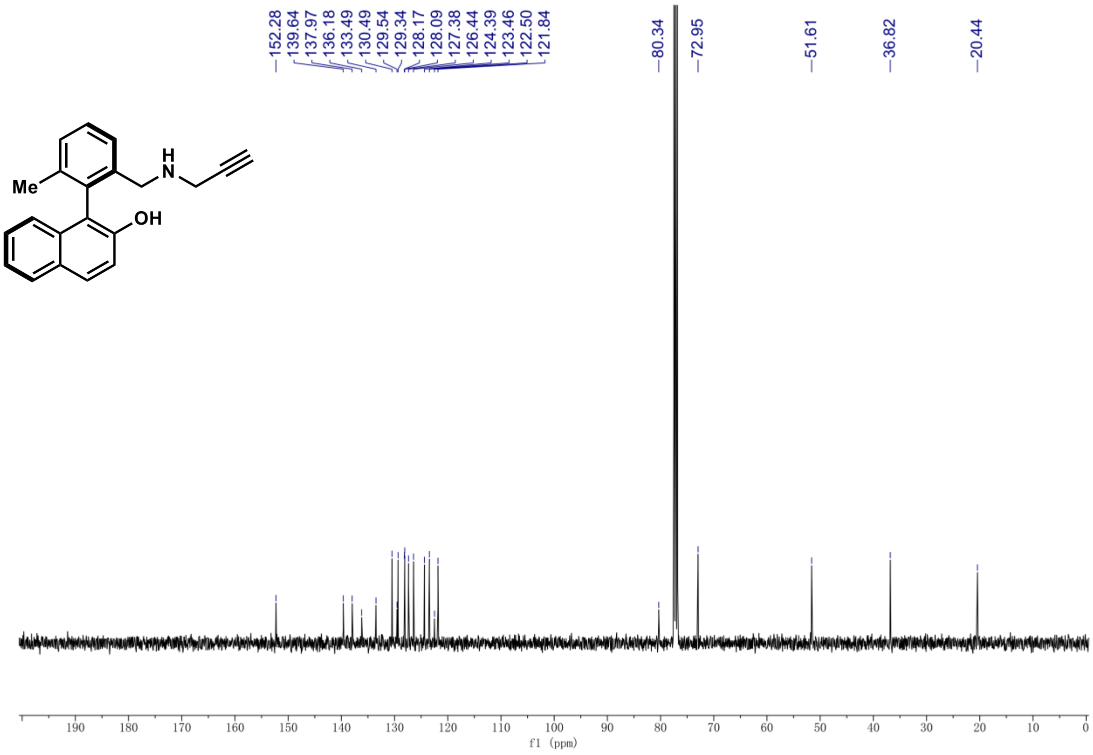

^{1}H NMR (400 MHz, $CDCl_3$) of **3e**

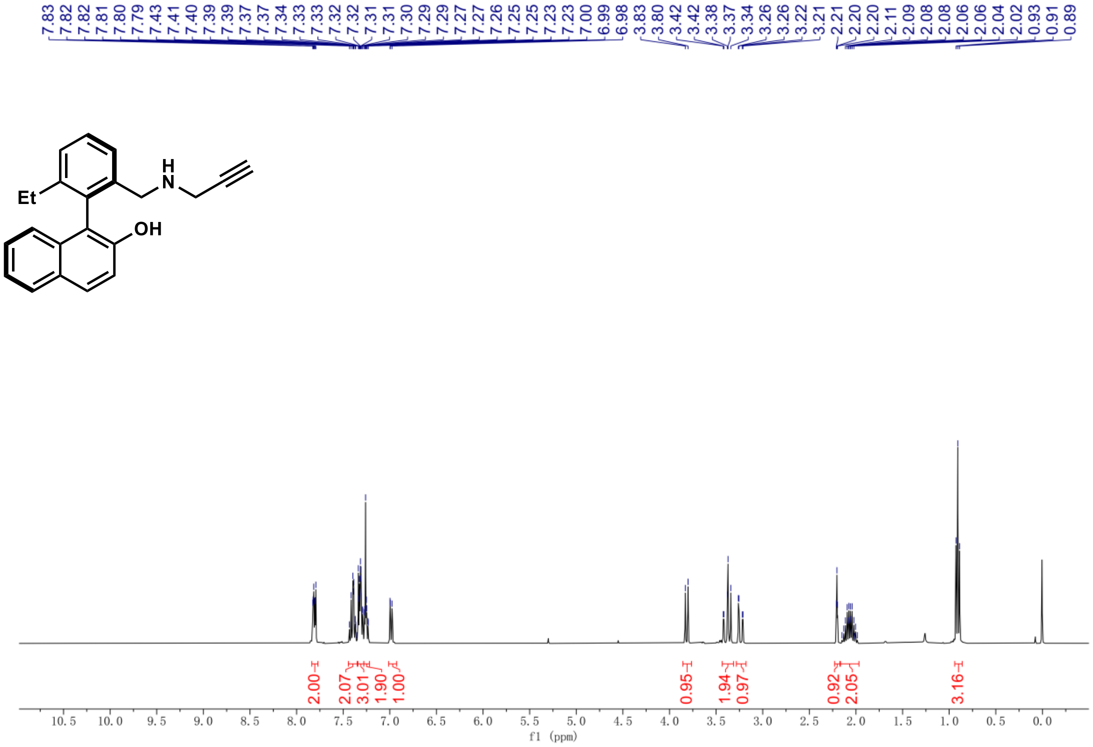

^{13}C NMR (101 MHz, $CDCl_3$) of **3e**

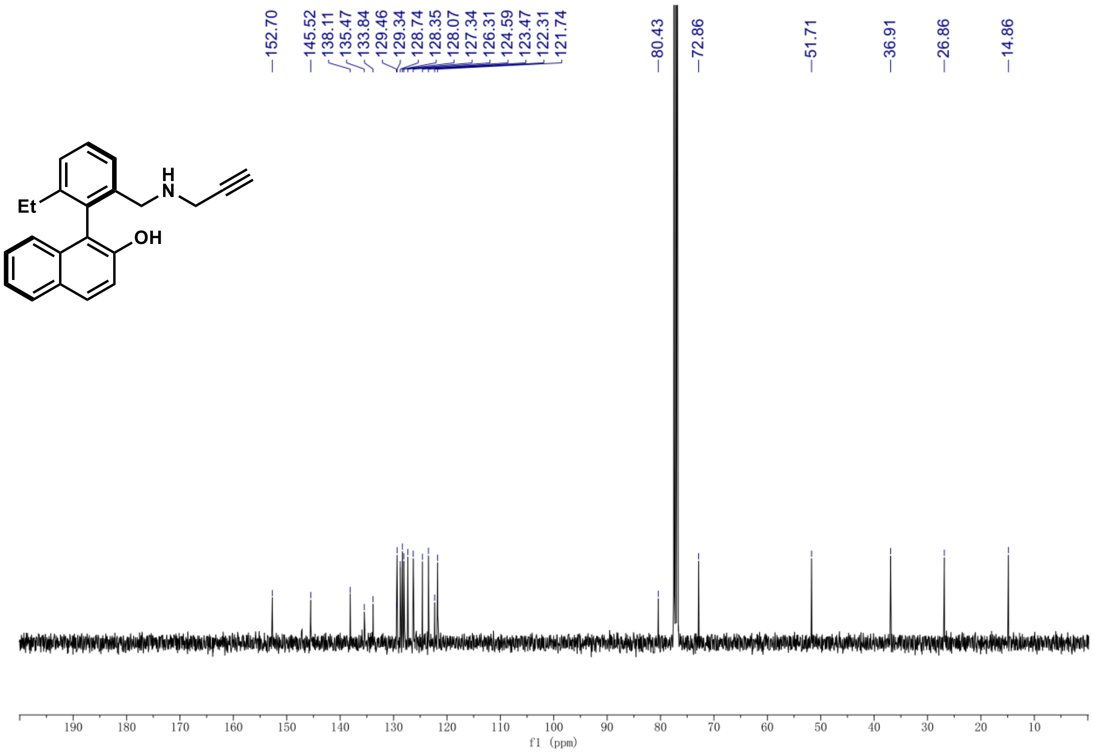

^{1}H NMR (400 MHz, $CDCl_3$) of **3f**

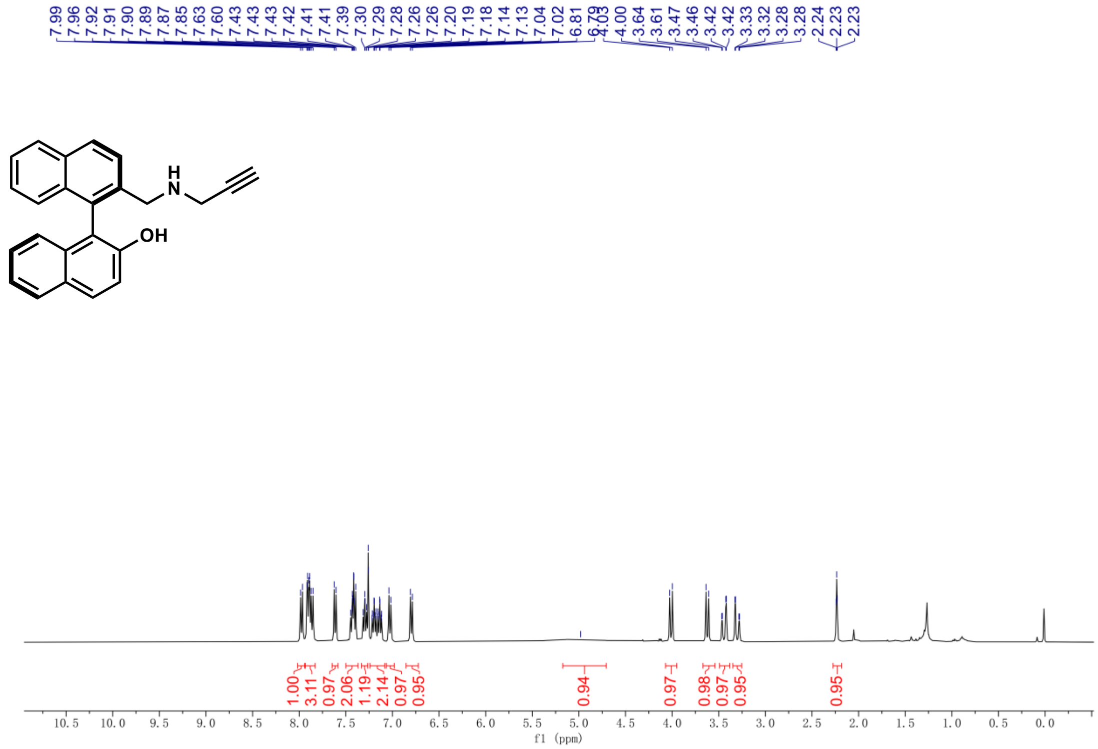

^{13}C NMR (101 MHz, $CDCl_3$) of **3f**

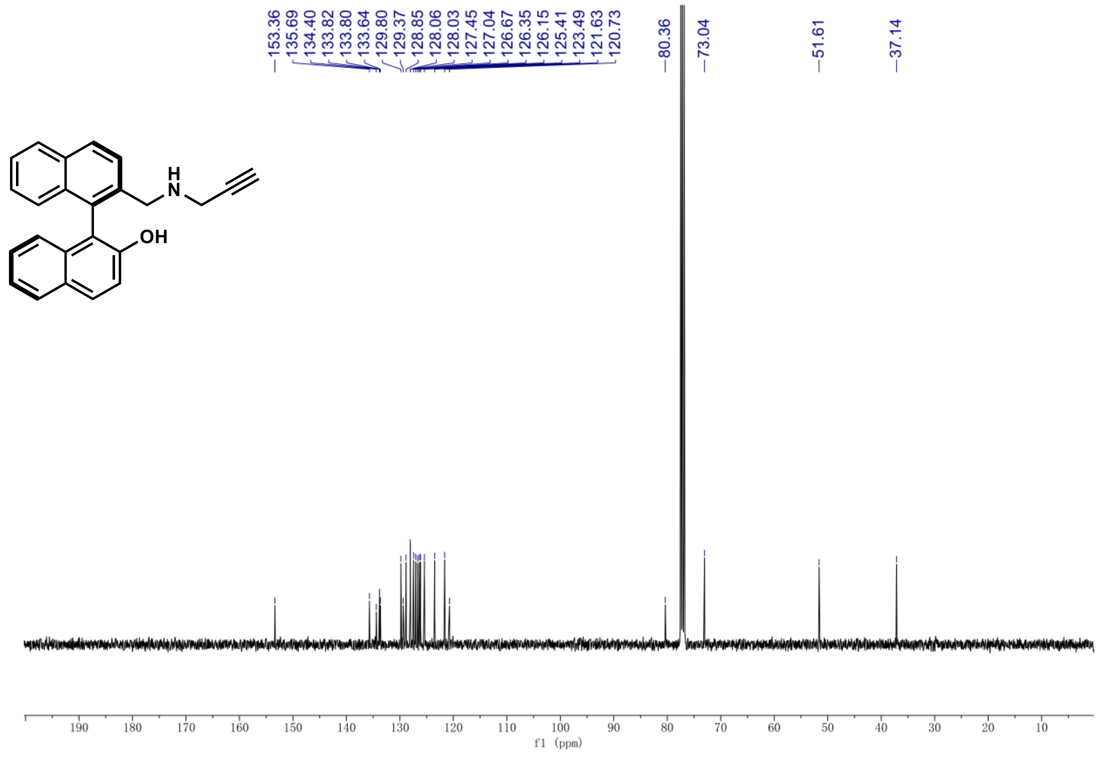

^{1}H NMR (400 MHz, $CDCl_3$) of **3g**

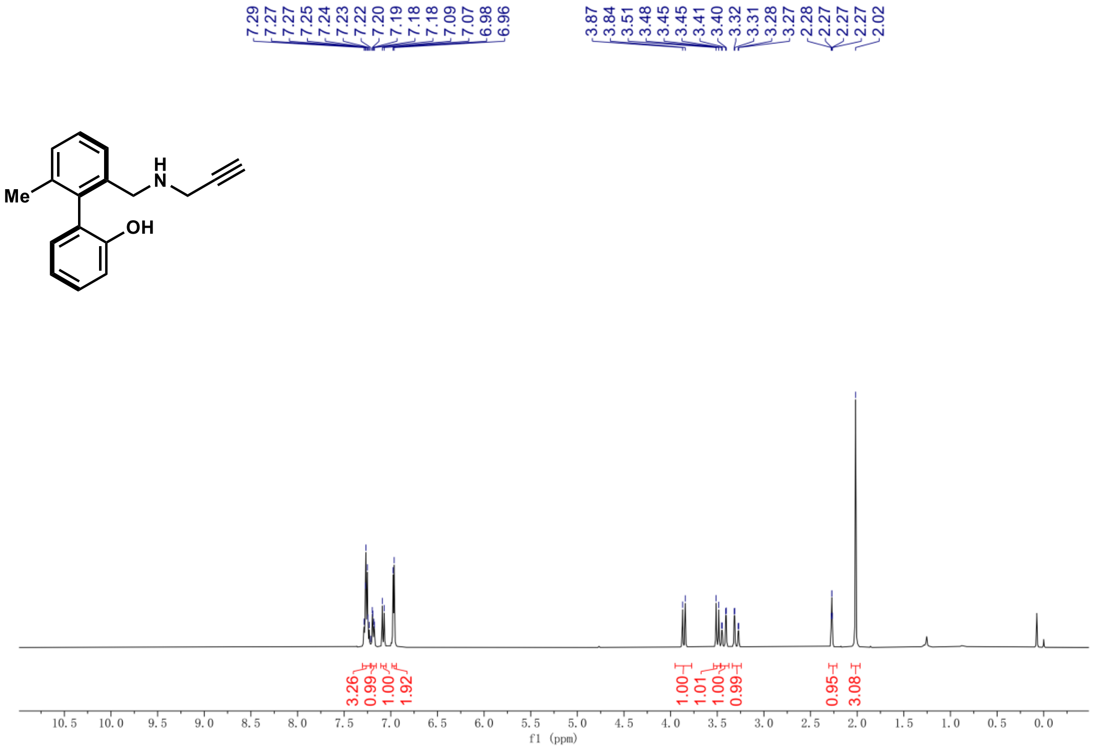

^{13}C NMR (101 MHz, $CDCl_3$) of **3g**

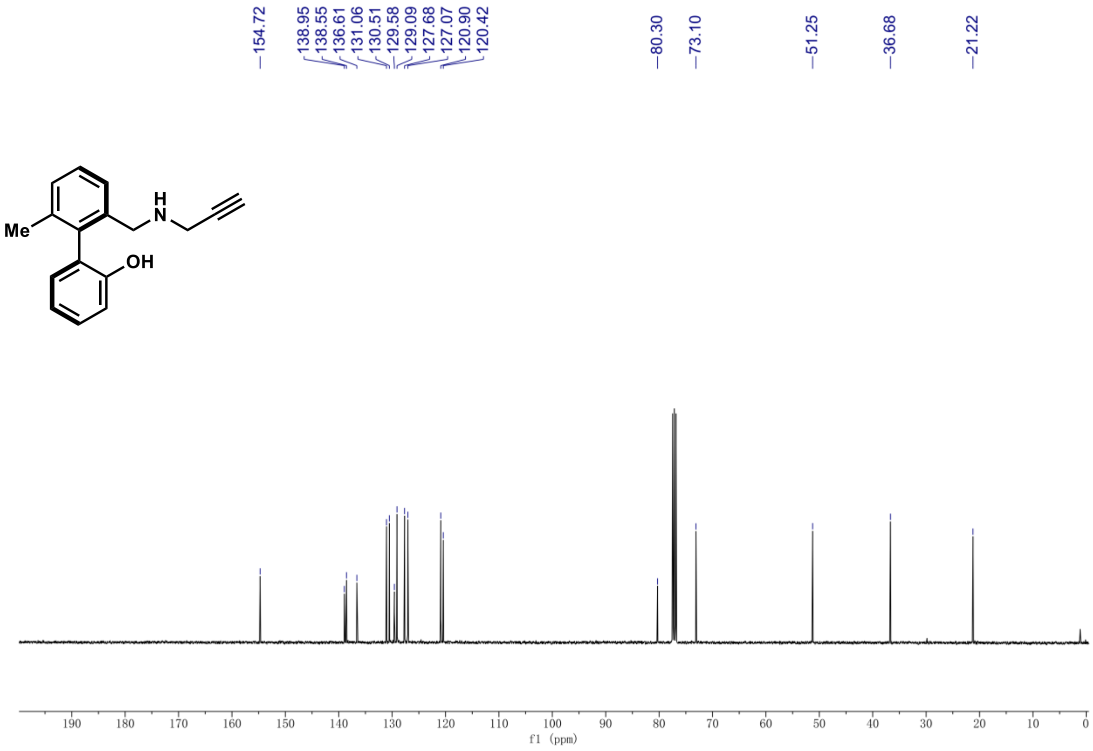

^{1}H NMR (400 MHz, $CDCl_3$) of **3h**

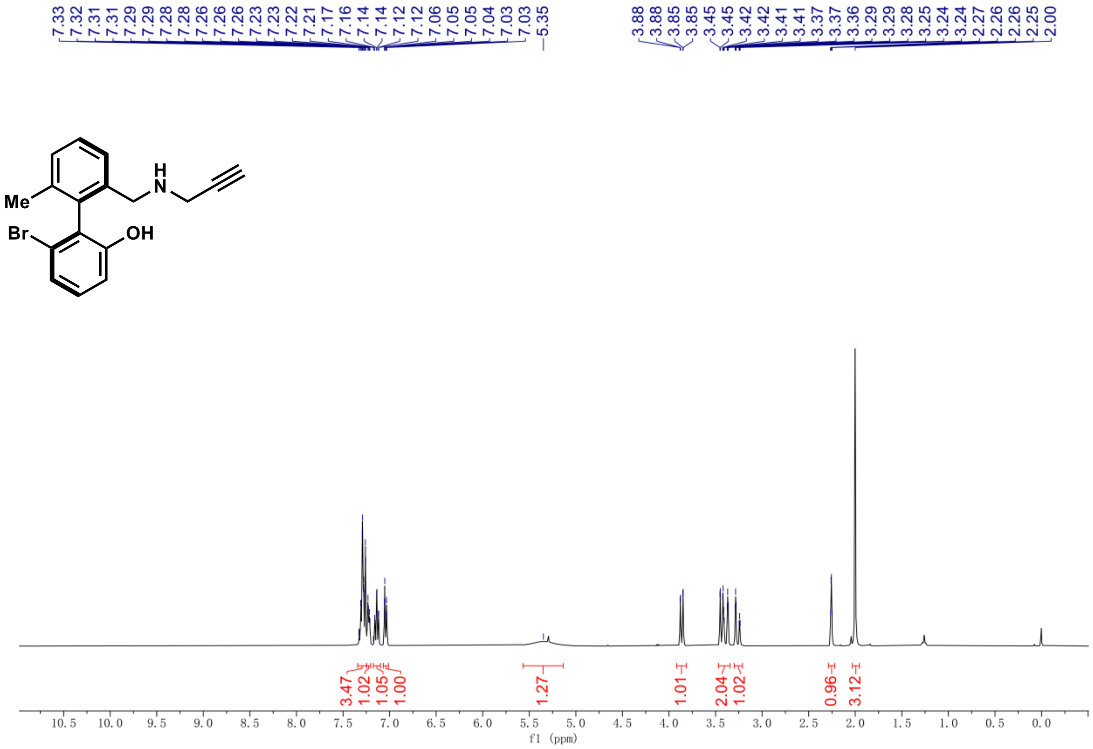

^{13}C NMR (101 MHz, $CDCl_3$) of **3h**

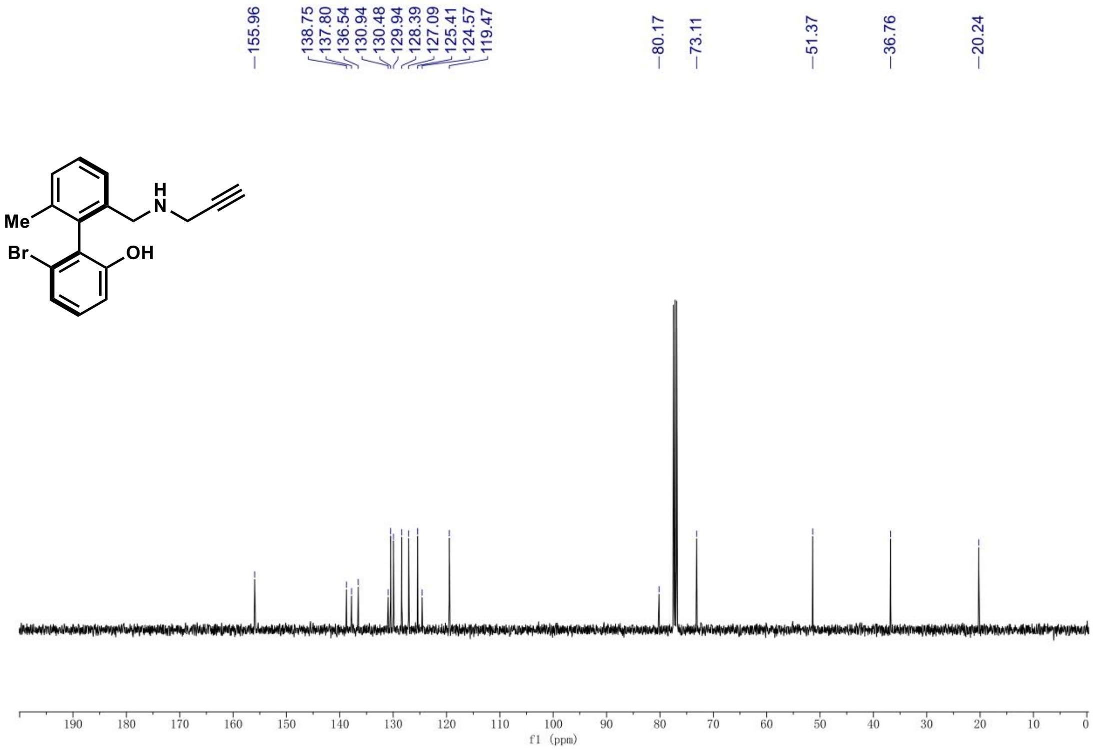

^{1}H NMR (400 MHz, $CDCl_3$) of **3i**

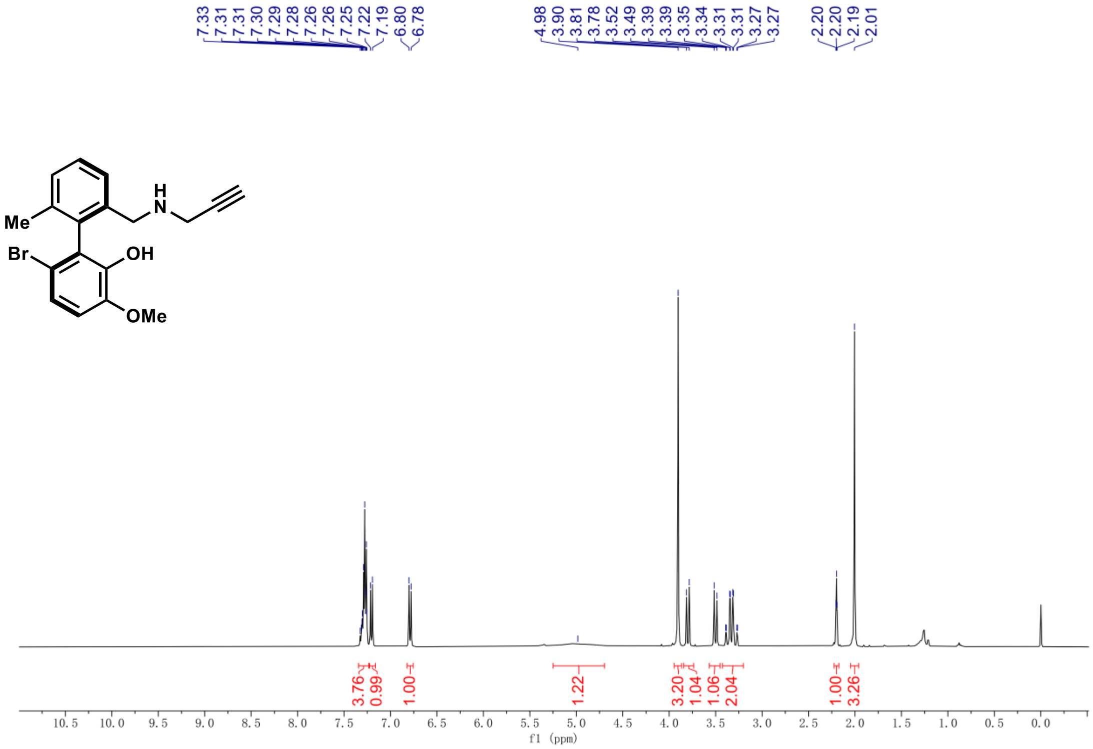

^{13}C NMR (101 MHz, $CDCl_3$) of **3i**

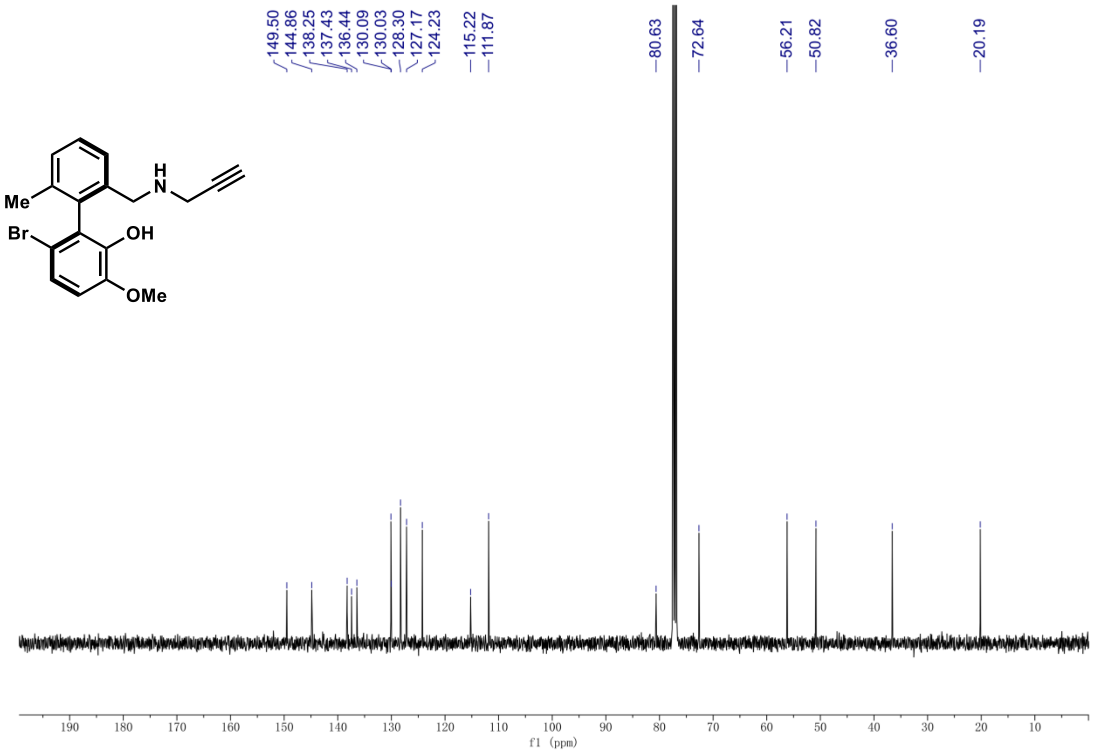

^{1}H NMR (400 MHz, $CDCl_3$) of **3j**

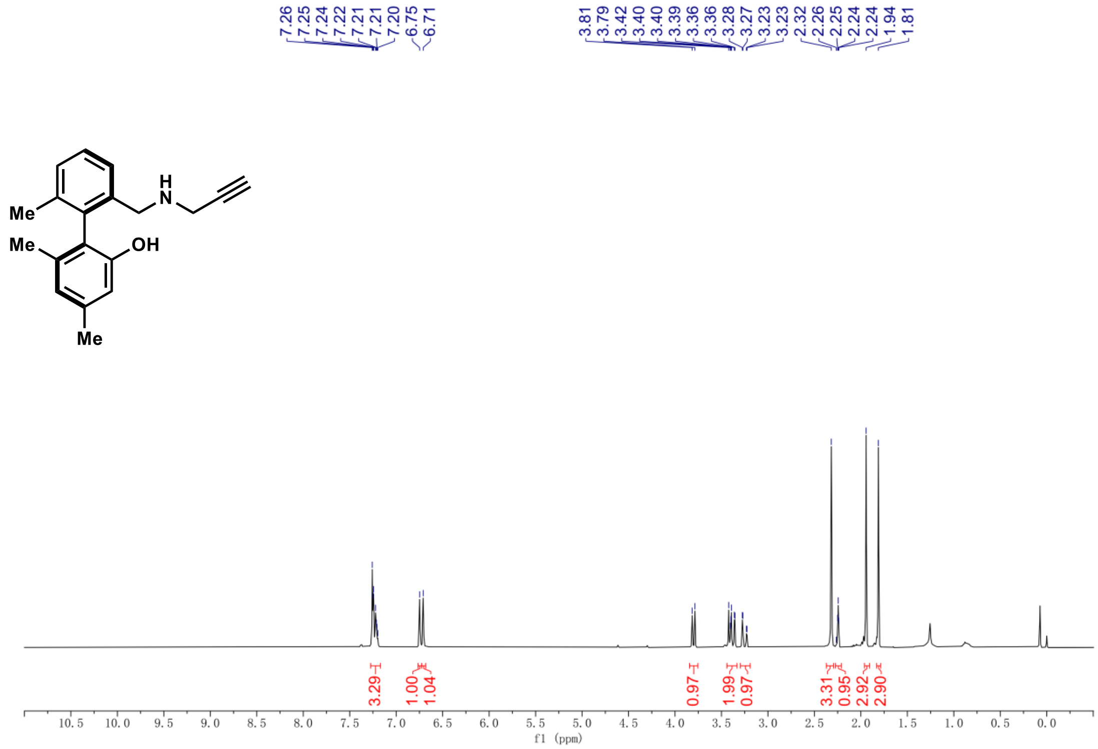

^{13}C NMR (101 MHz, $CDCl_3$) of **3j**

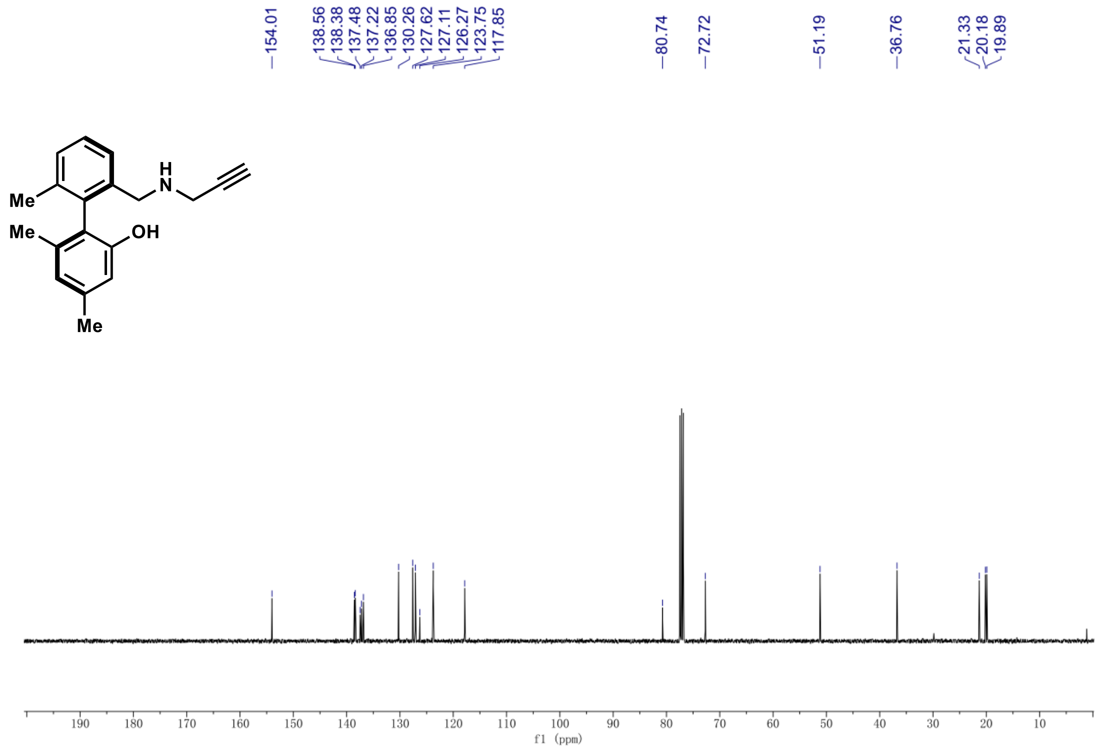

^{1}H NMR (400 MHz, $CDCl_3$) of **3k**

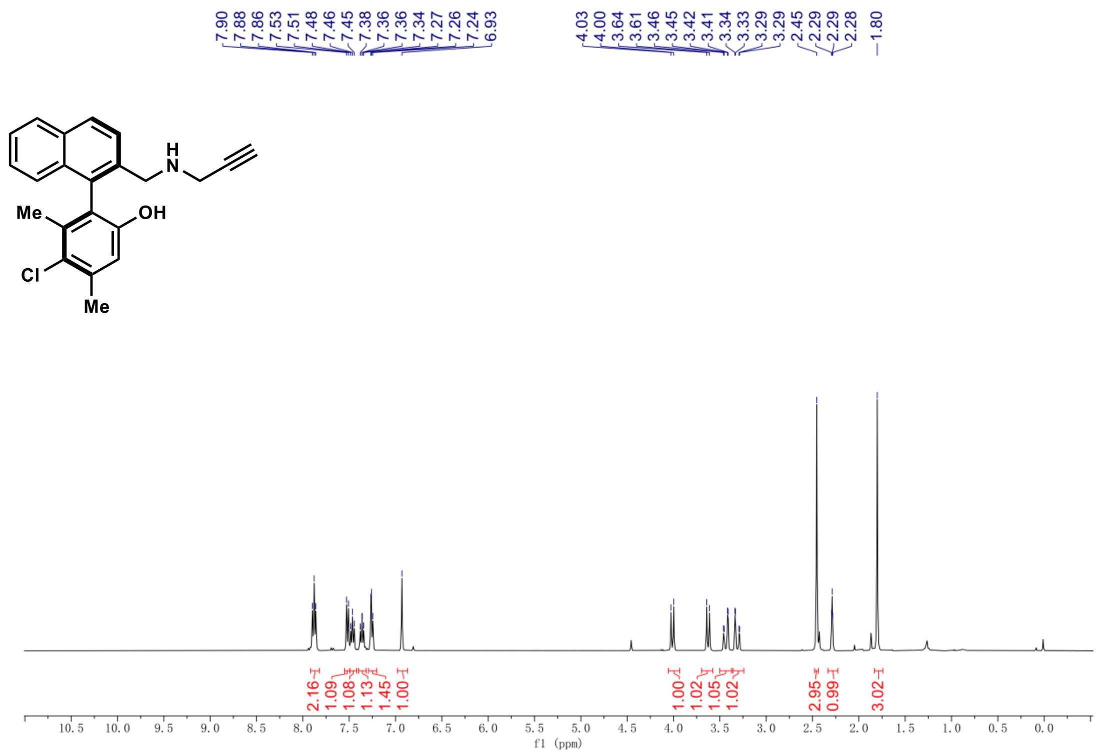

^{13}C NMR (101 MHz, $CDCl_3$) of **3k**

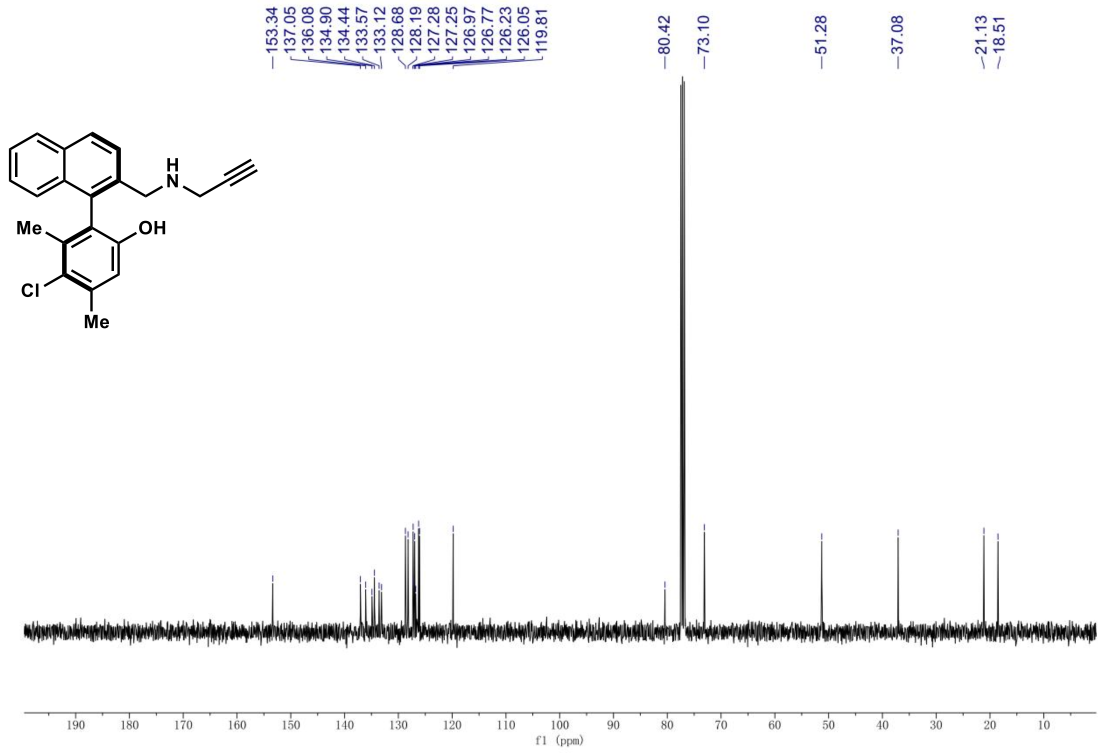

^{1}H NMR (400 MHz, $CDCl_3$) of **5a**

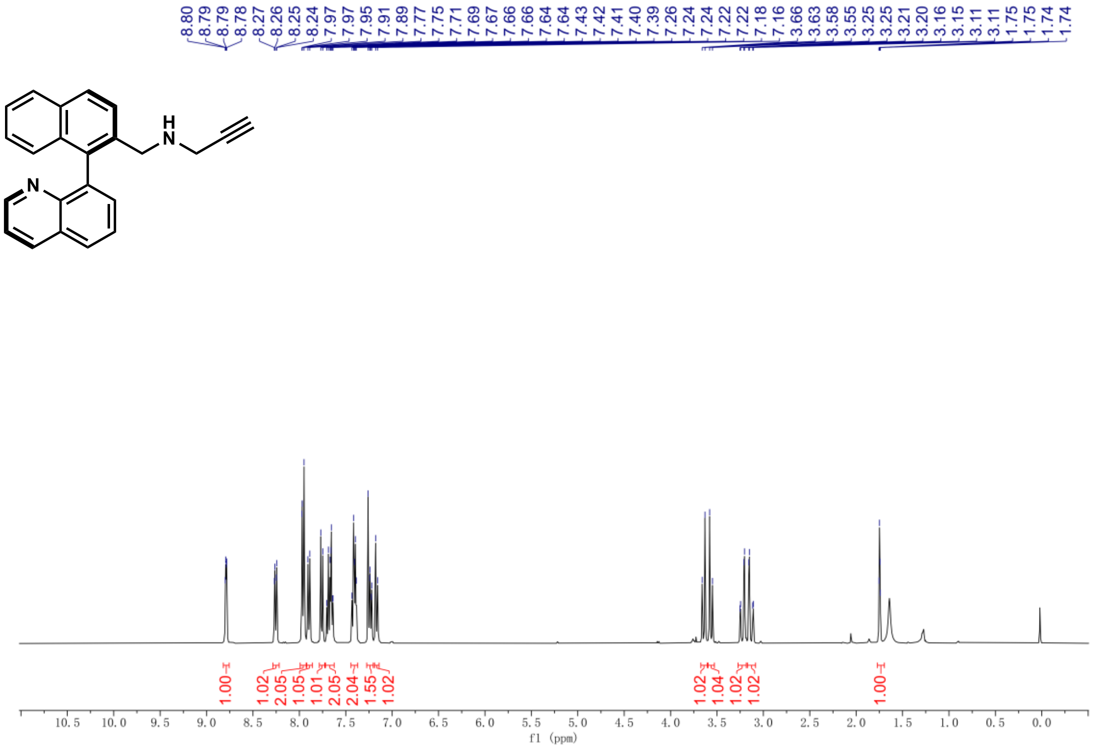

^{13}C NMR (101 MHz, $CDCl_3$) of **5a**

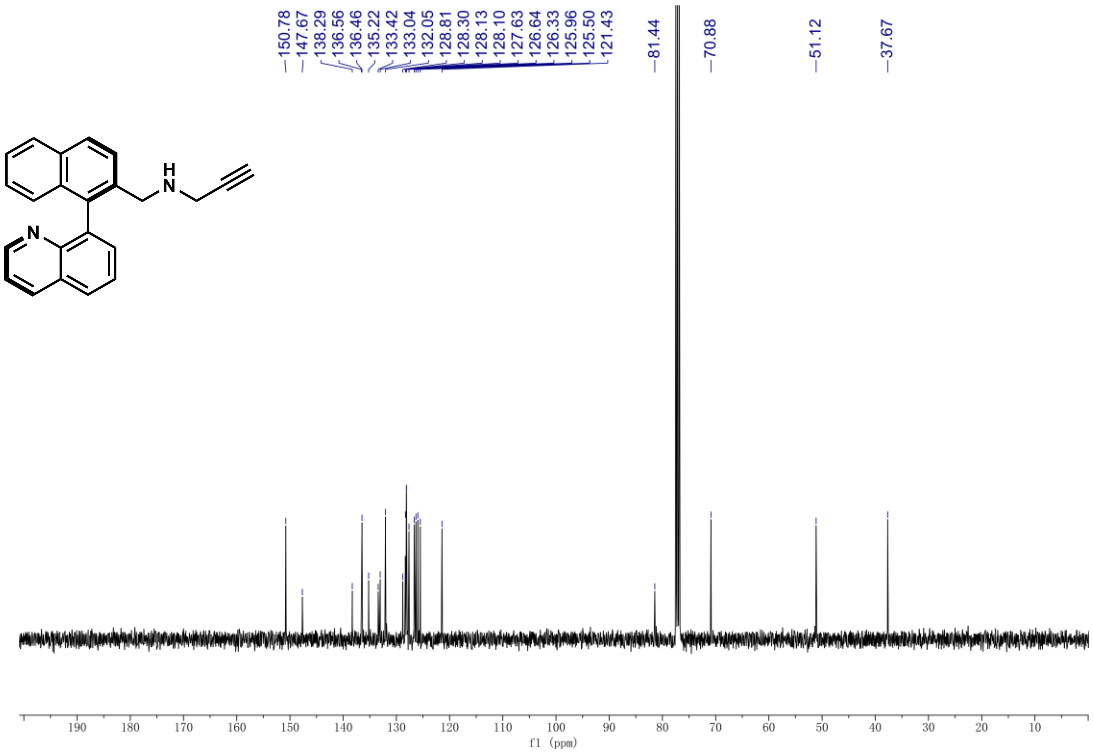

^{1}H NMR (400 MHz, $CDCl_3$) of **5b**

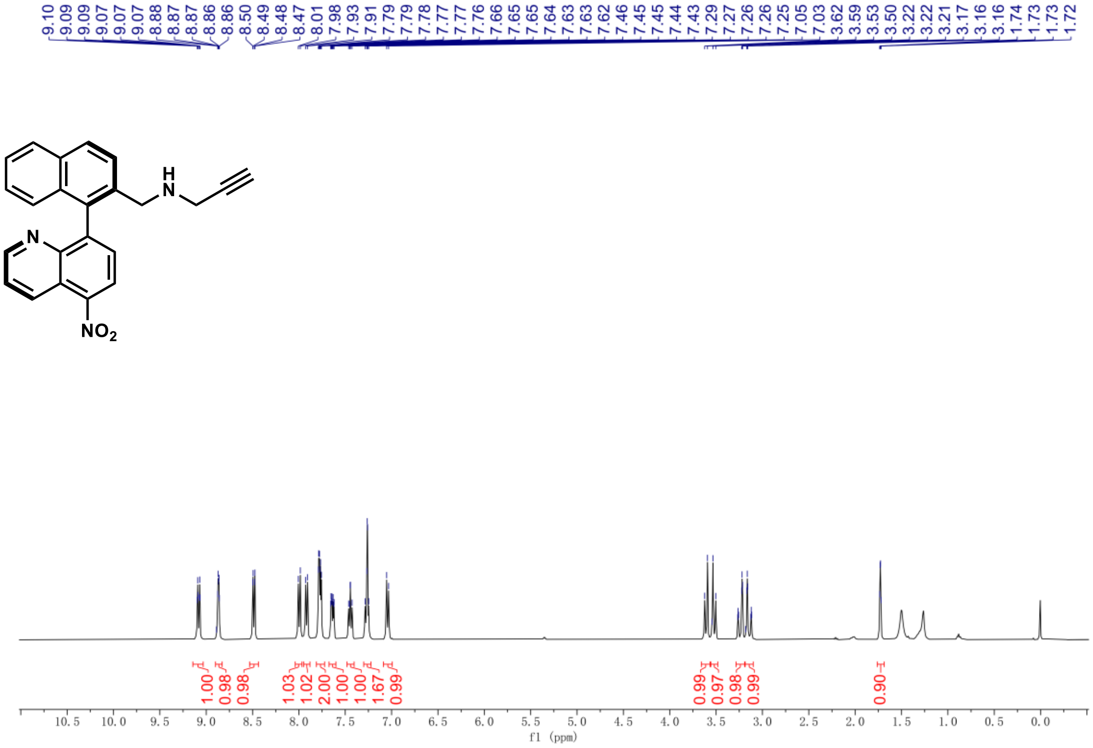

^{13}C NMR (101 MHz, $CDCl_3$) of **5b**

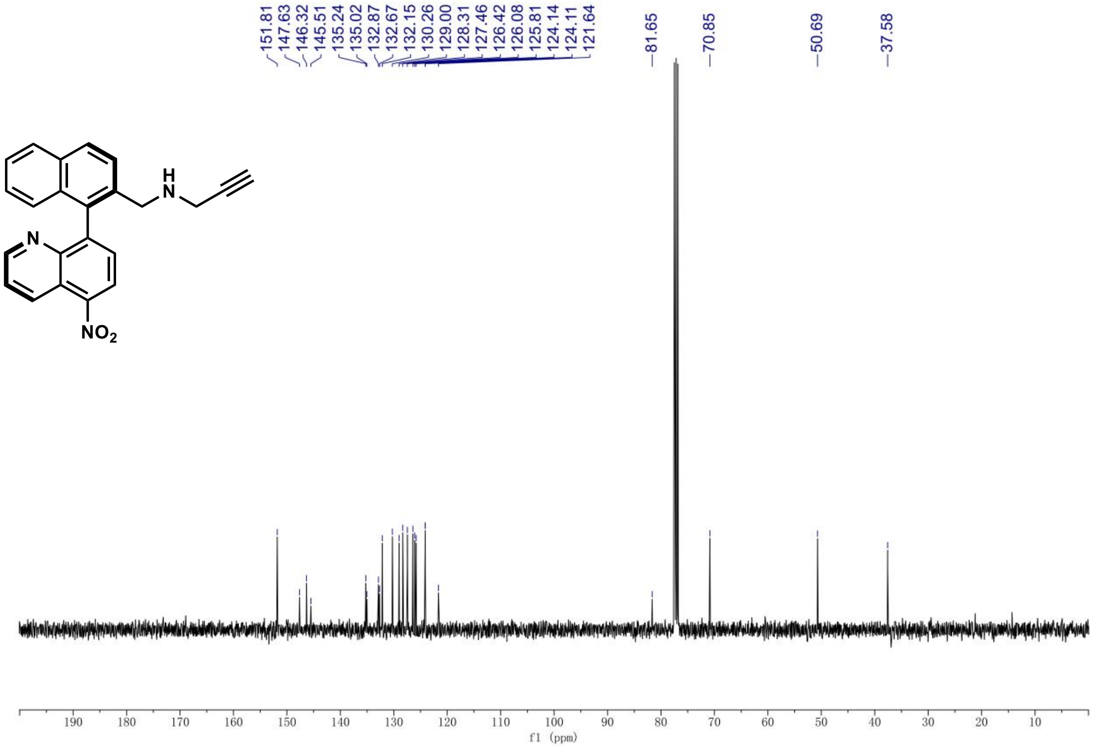

^{1}H NMR (400 MHz, $CDCl_3$) of **5c**

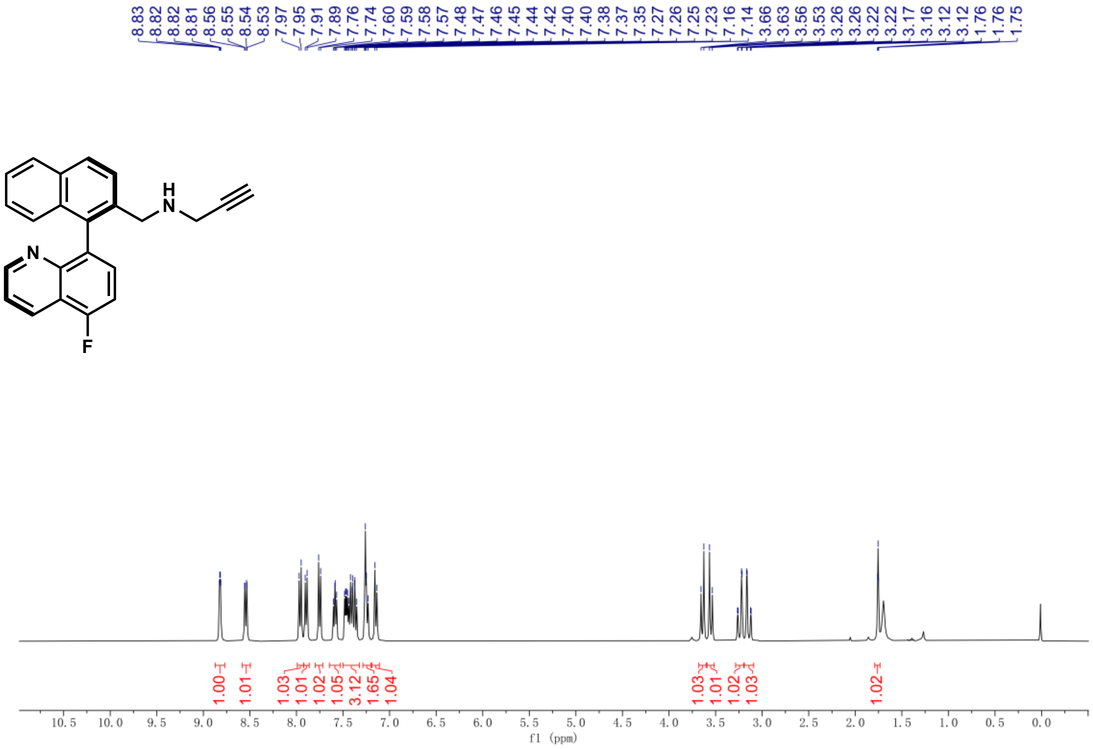

^{13}C NMR (126 MHz, $CDCl_3$) of **5c**

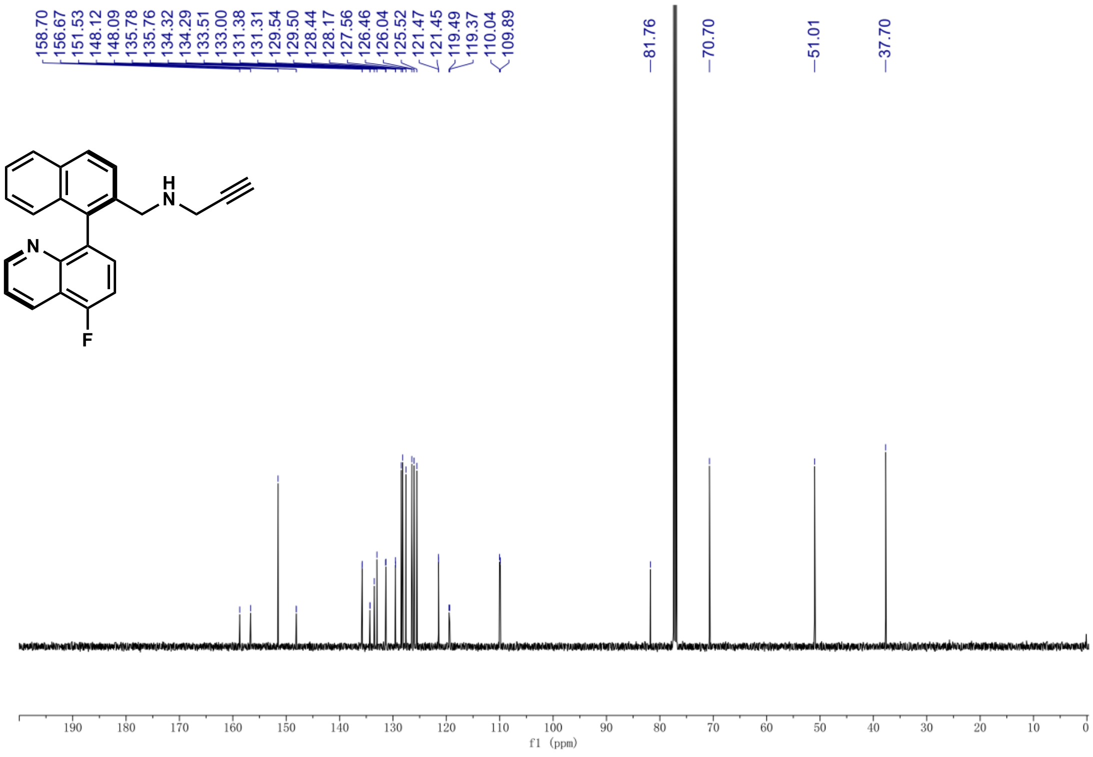

^{19}F NMR (470 MHz, $CDCl_3$) of **5c**

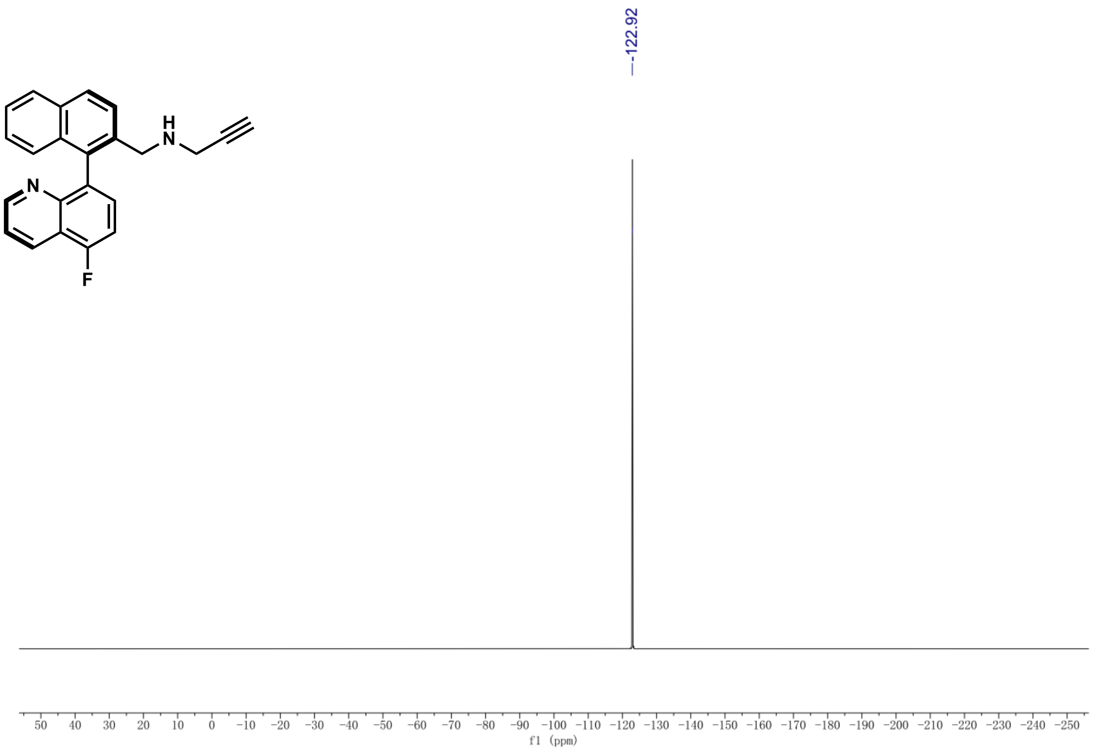

^{1}H NMR (400 MHz, $CDCl_3$) of **5d**

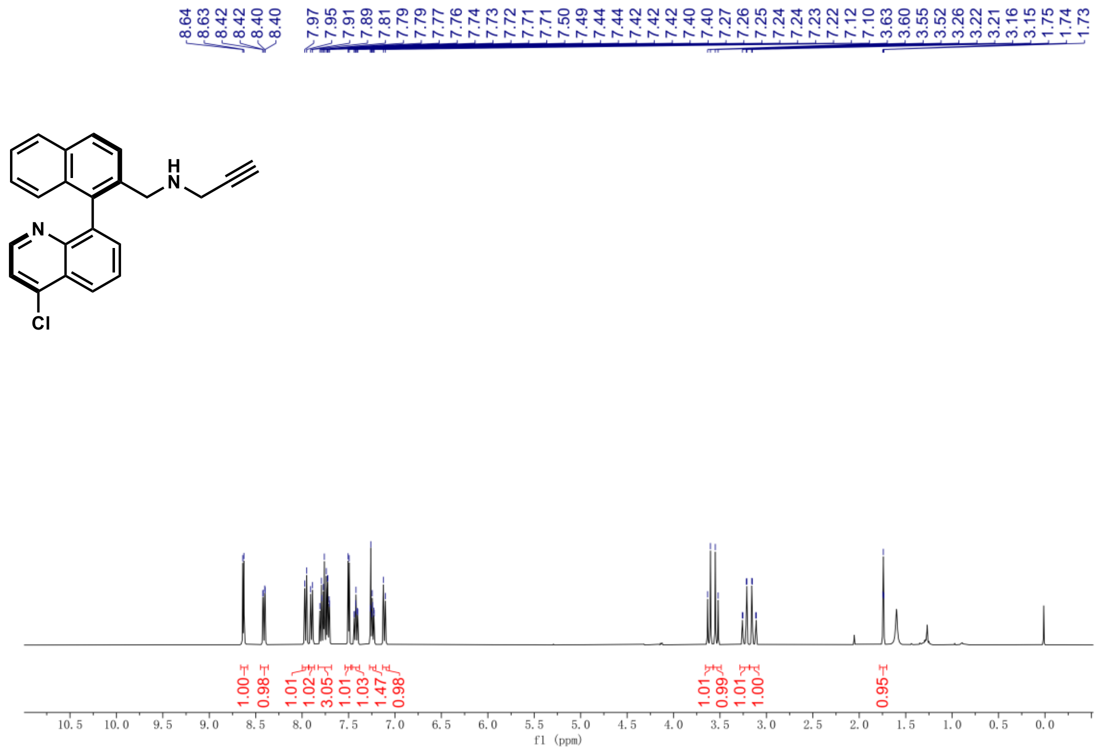

^{13}C NMR (101 MHz, $CDCl_3$) of **5d**

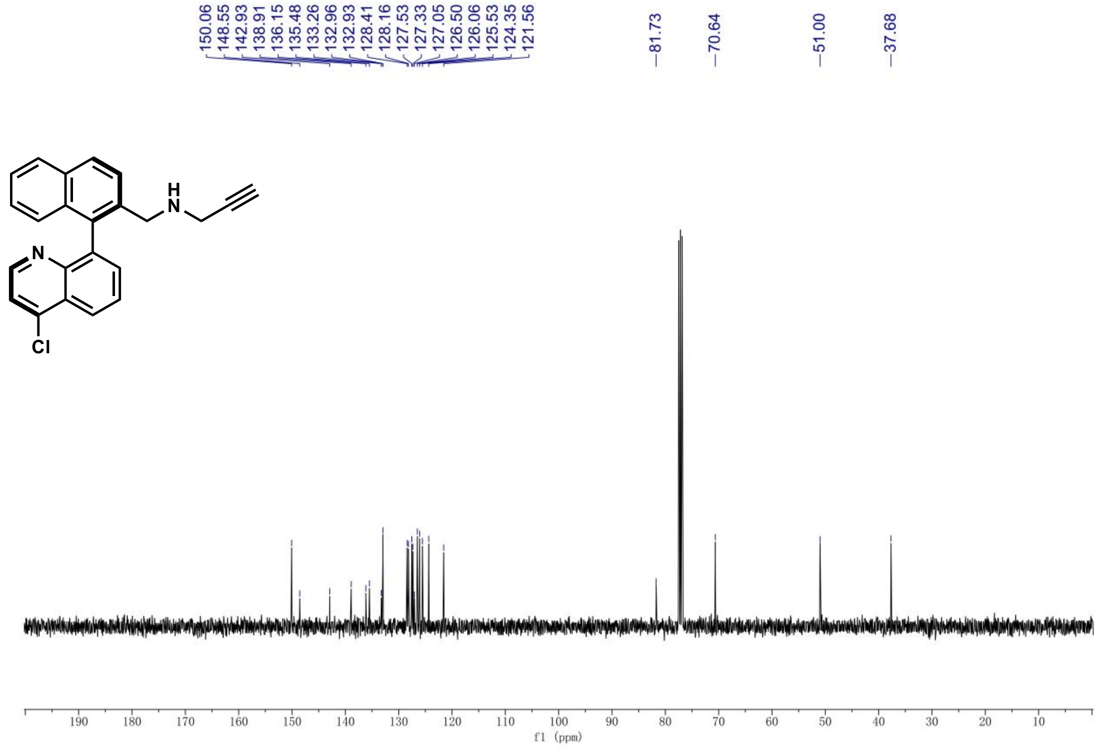

^{1}H NMR (400 MHz, $CDCl_3$) of **5e**

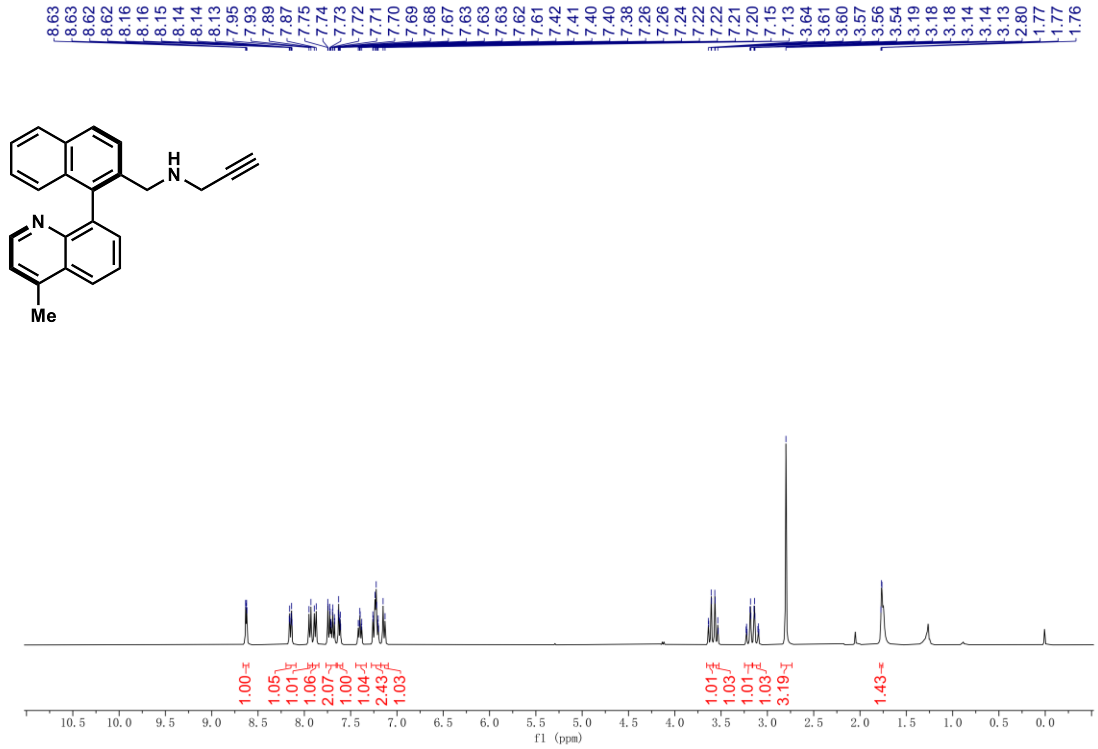

^{13}C NMR (101 MHz, $CDCl_3$) of **5e**

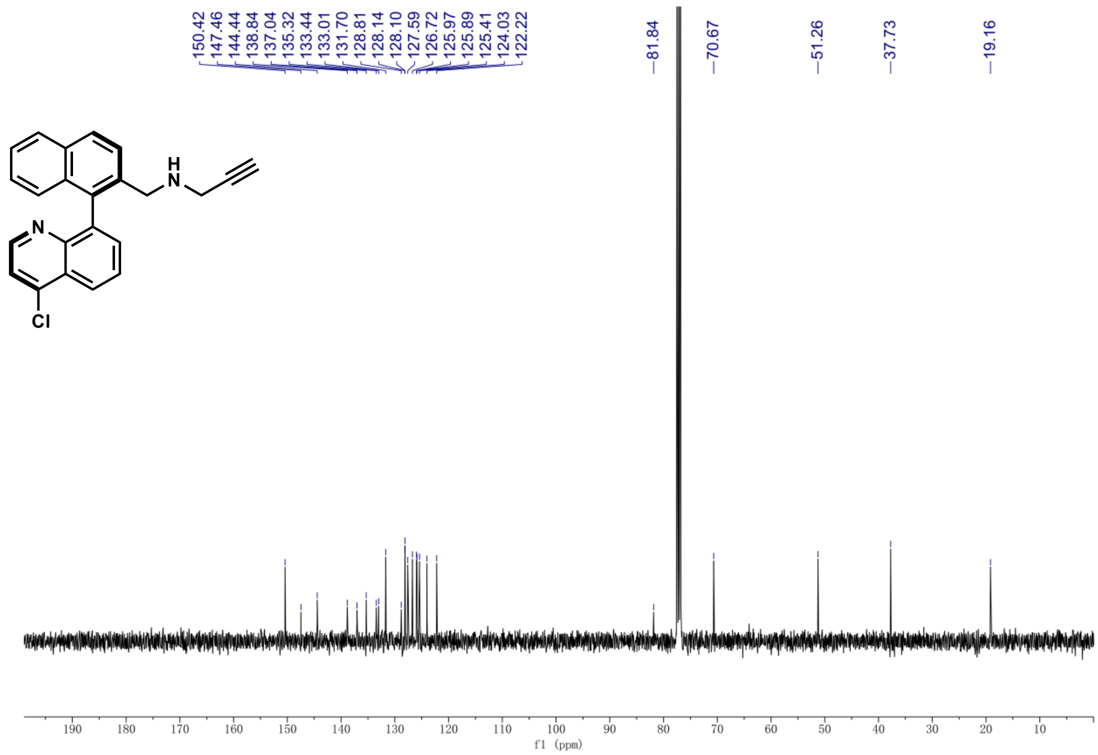

^{1}H NMR (400 MHz, $CDCl_3$) of **5f**

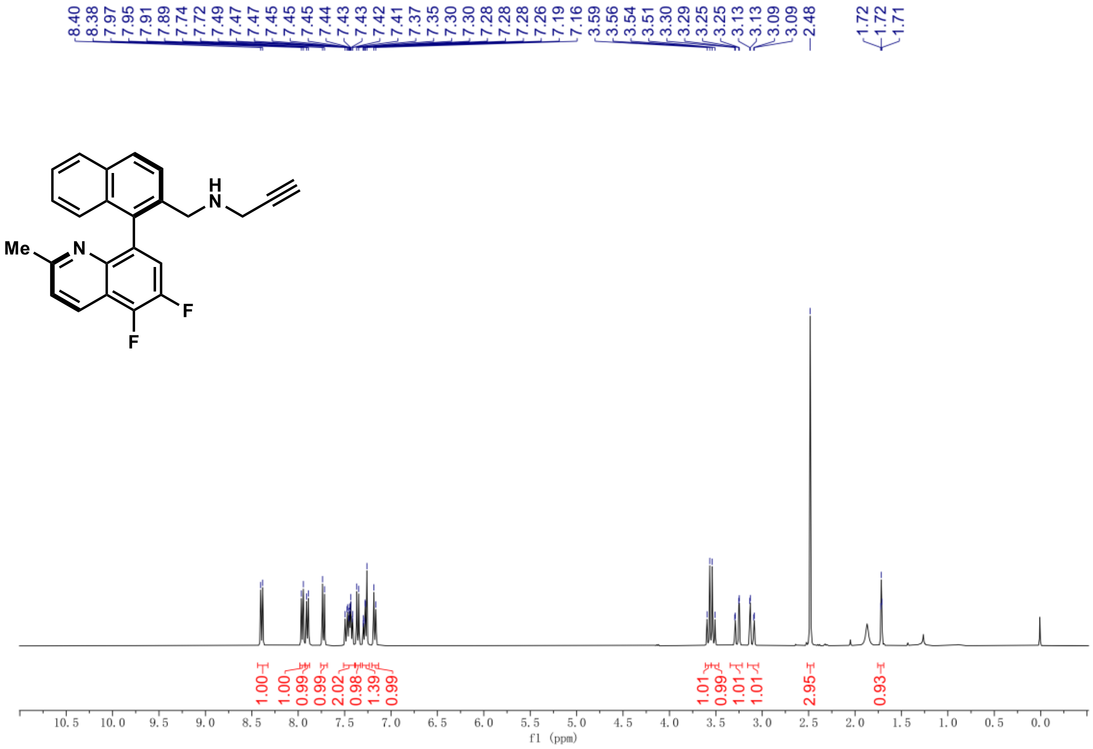

^{13}C NMR (126 MHz, $CDCl_3$) of **5f**

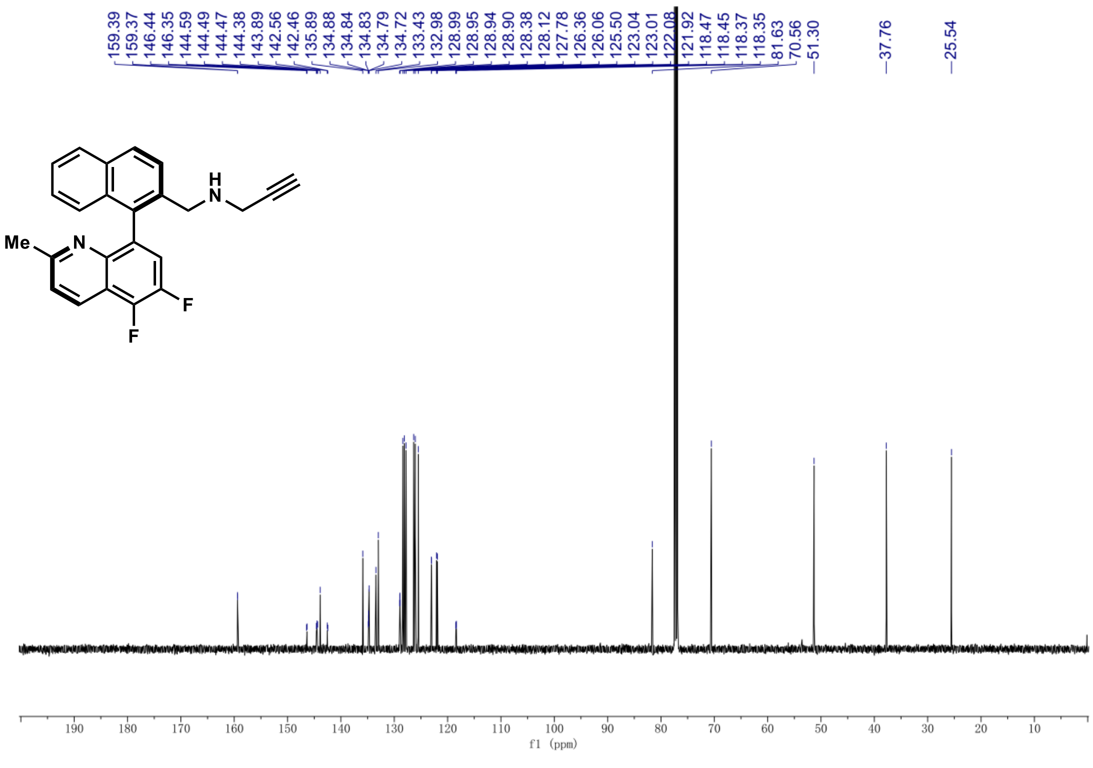

^{19}F NMR (470 MHz, CDCl$_3$) of **5f**

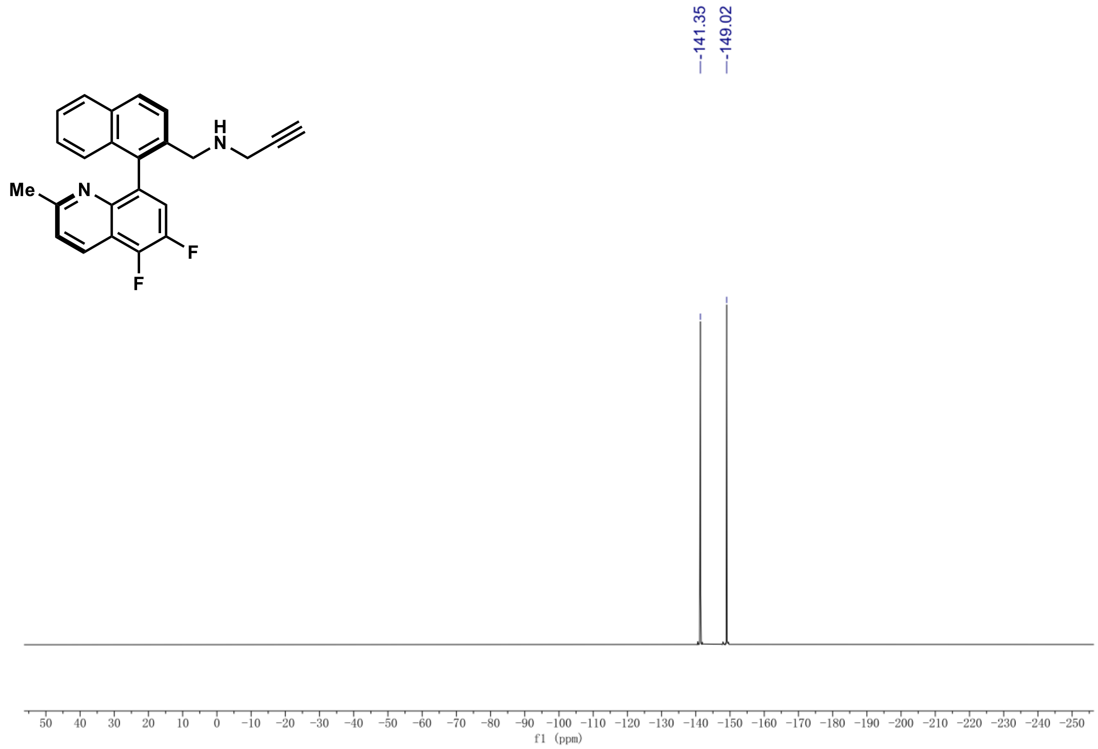

^{1}H NMR (400 MHz, $CDCl_3$) of **5g**

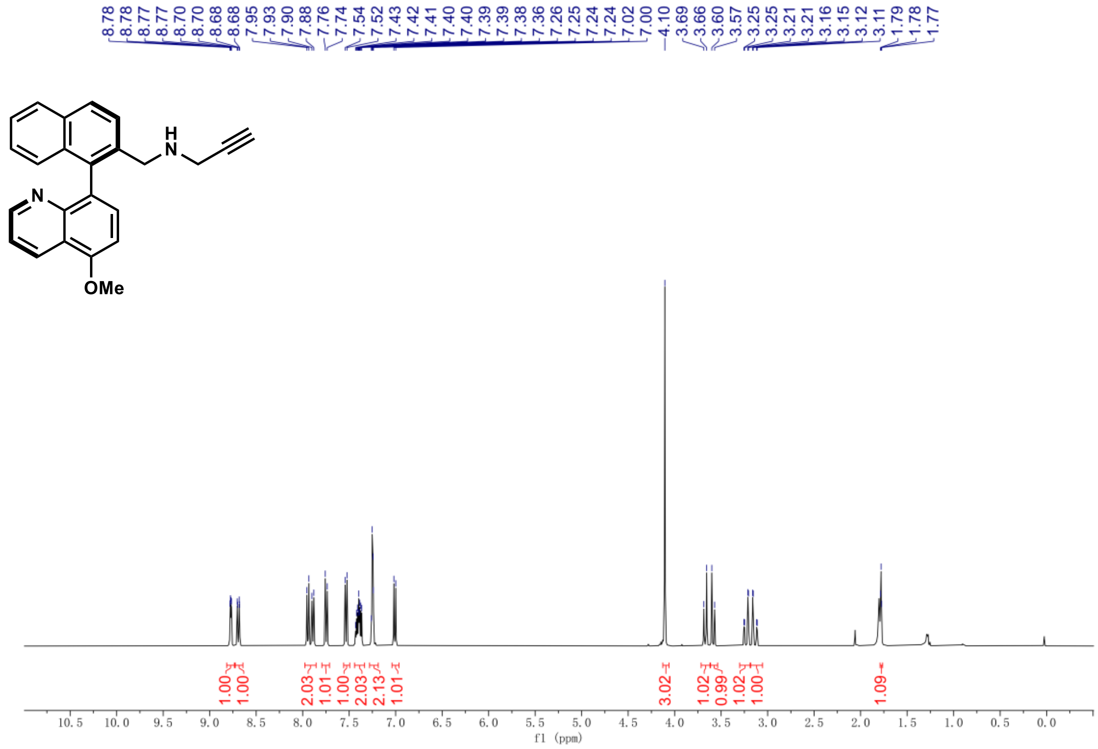

^{13}C NMR (101 MHz, $CDCl_3$) of **5g**

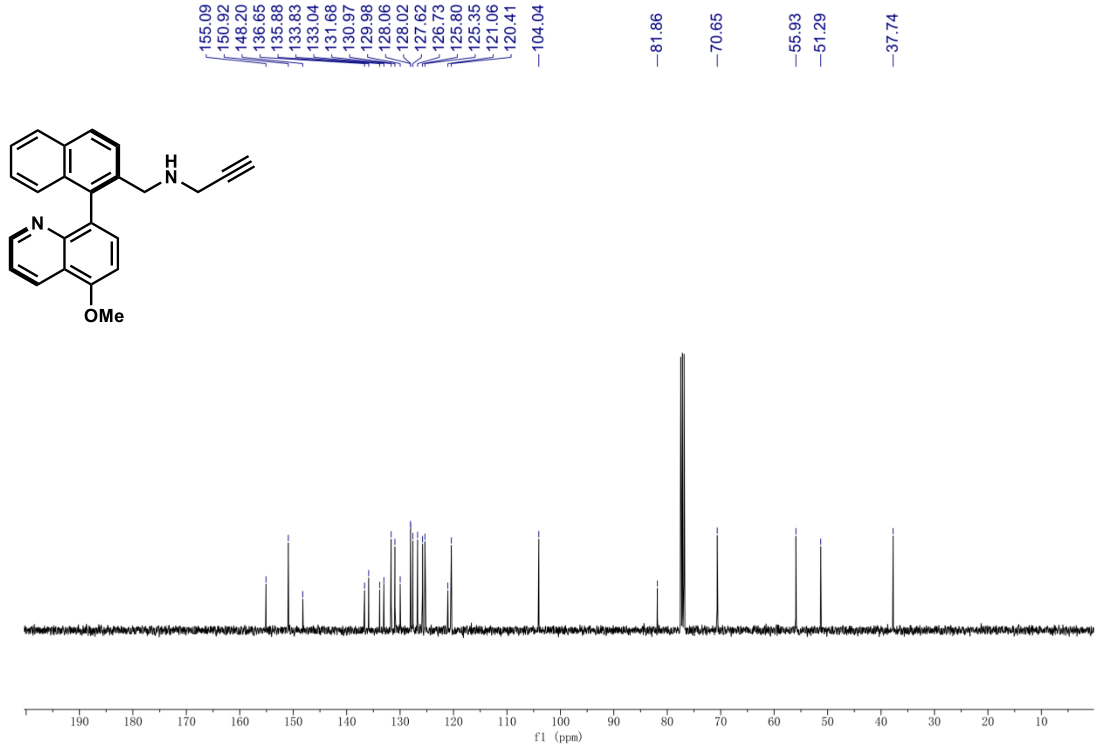

^{1}H NMR (400 MHz, $CDCl_3$) of **5h**

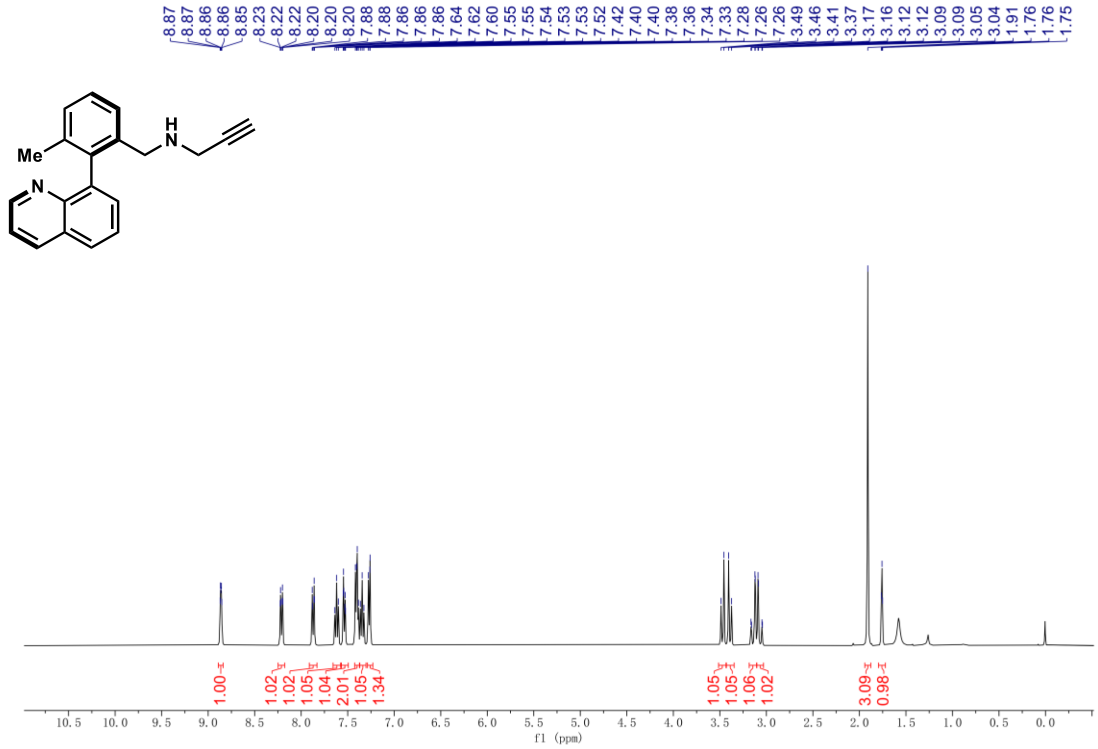

^{13}C NMR (101 MHz, $CDCl_3$) of **5h**

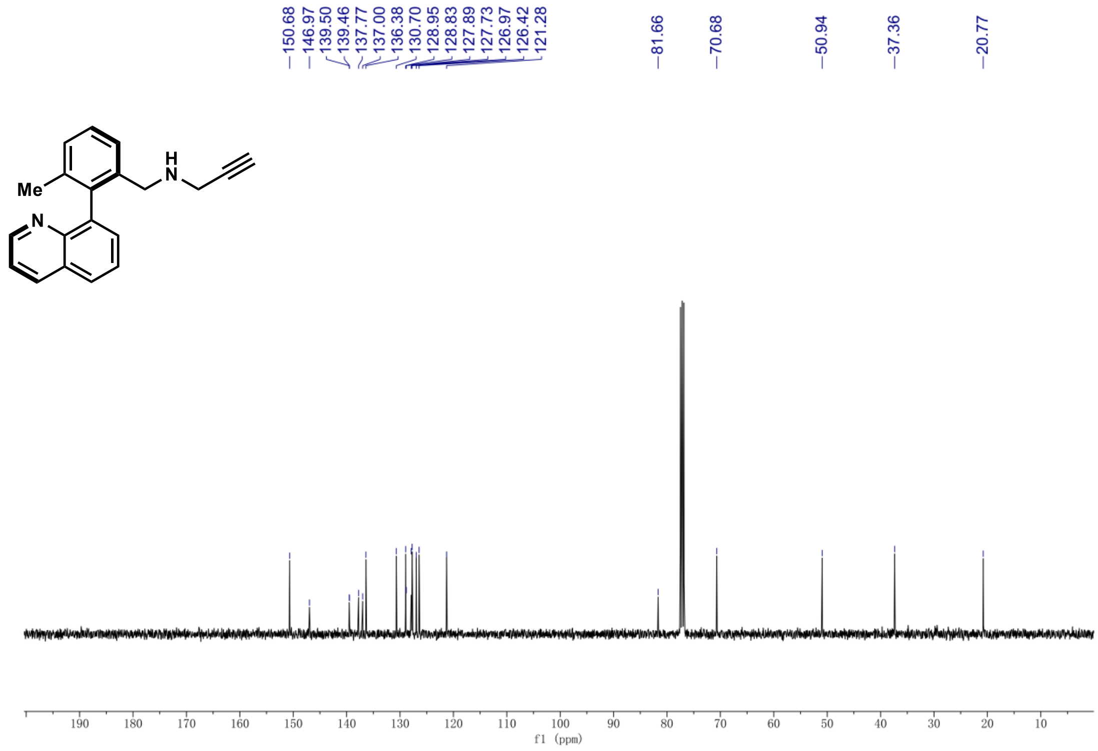

^{1}H NMR (400 MHz, $CDCl_3$) of **5i**

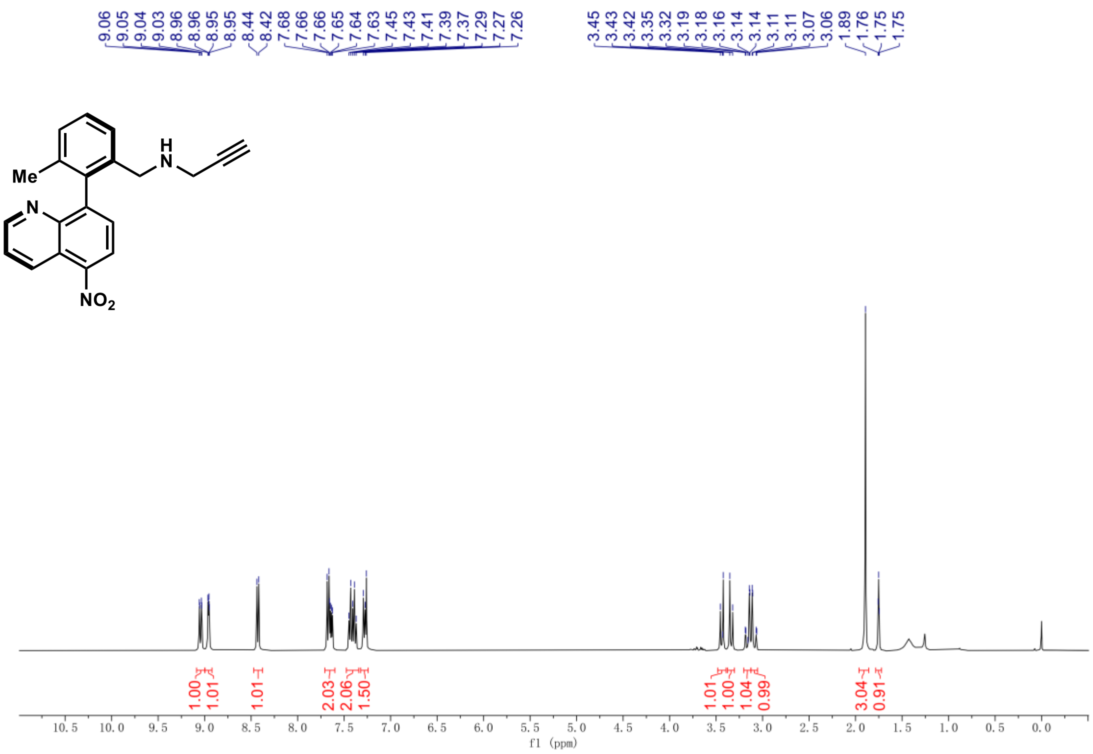

^{13}C NMR (101 MHz, $CDCl_3$) of **5i**

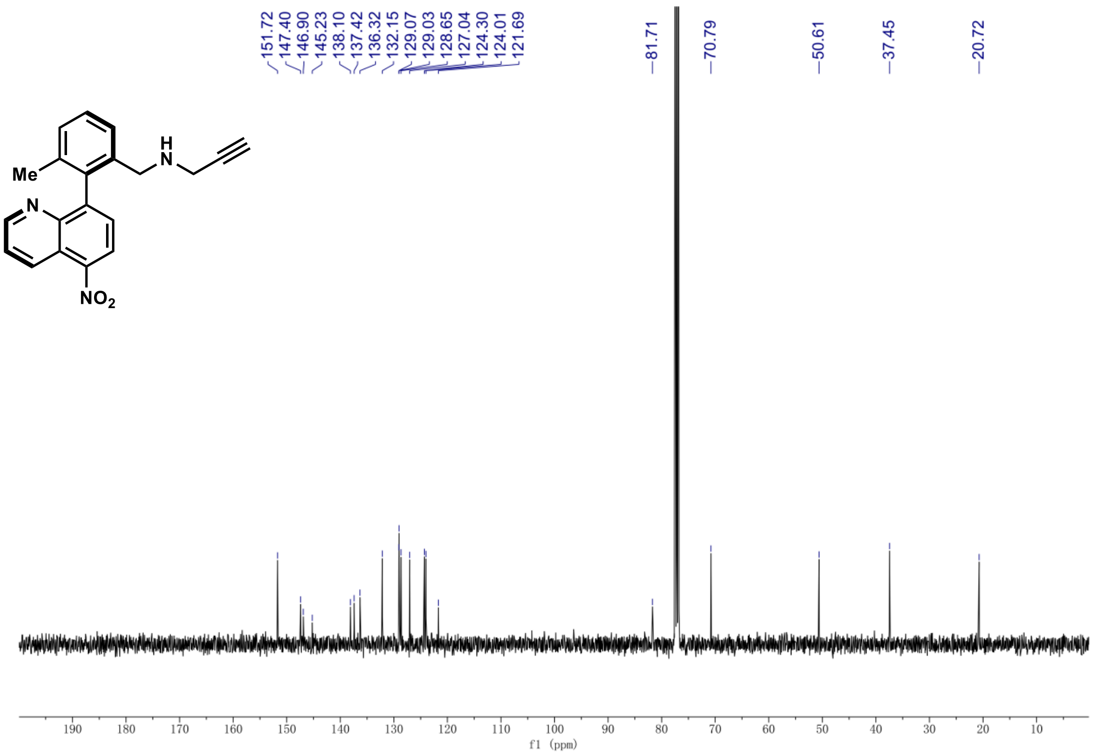

^{1}H NMR (400 MHz, $CDCl_3$) of **5j**

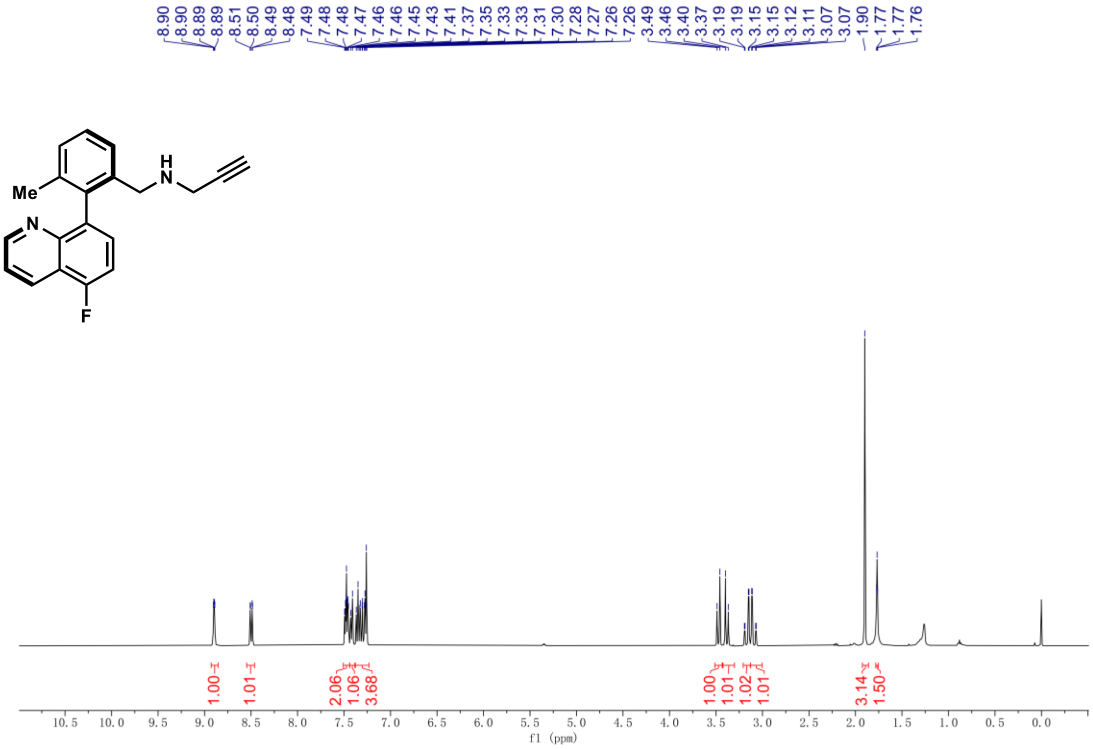

^{13}C NMR (126 MHz, $CDCl_3$) of **5j**

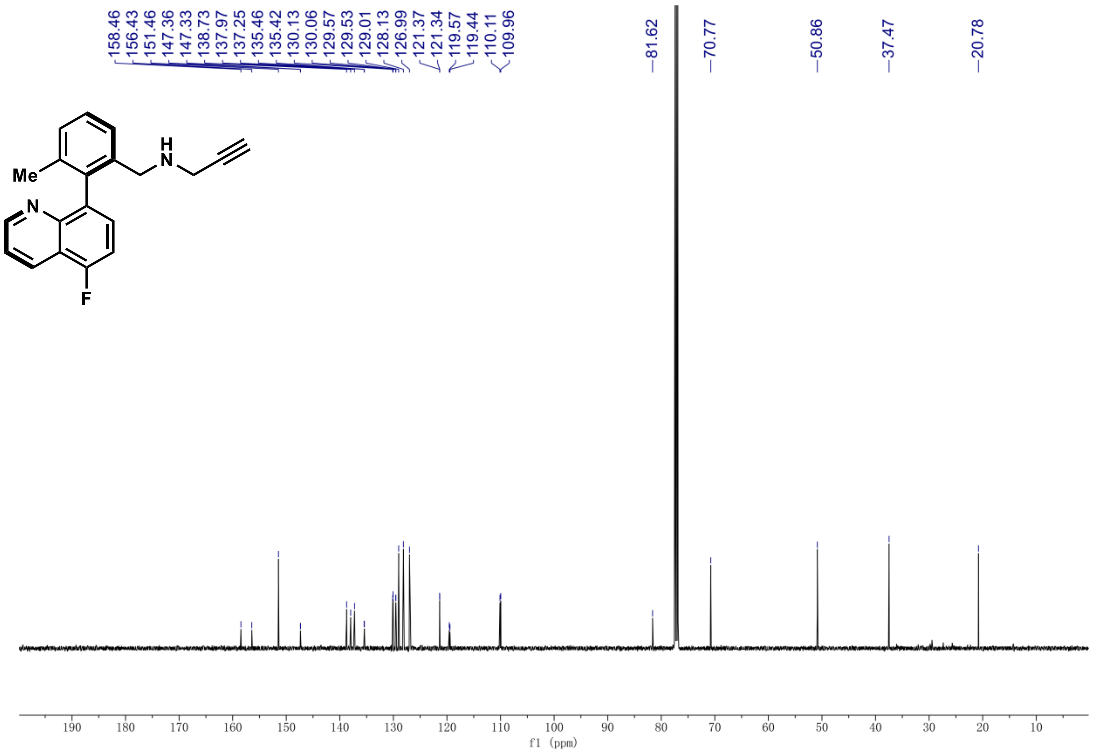

^{19}F NMR (470 MHz, $CDCl_3$) of **5j**

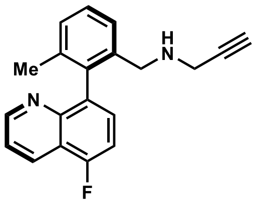

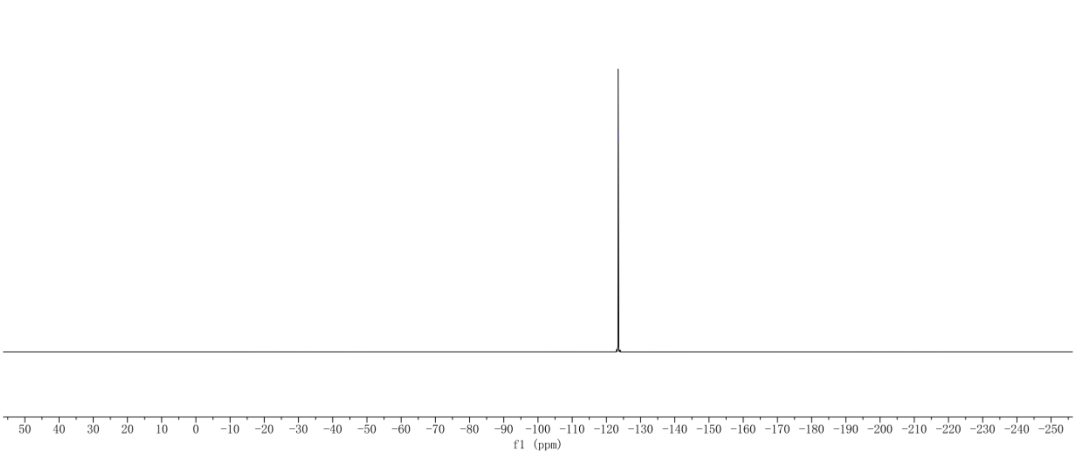

^{1}H NMR (400 MHz, $CDCl_3$) of **5k**

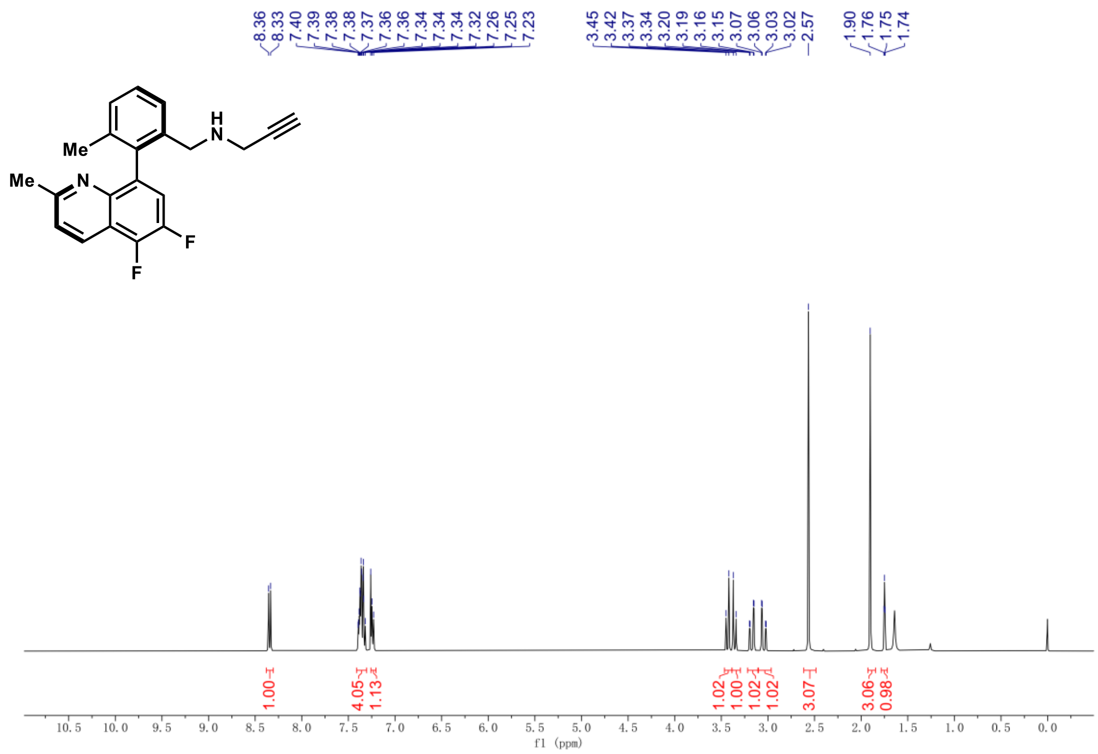

^{13}C NMR (126 MHz, $CDCl_3$) of **5k**

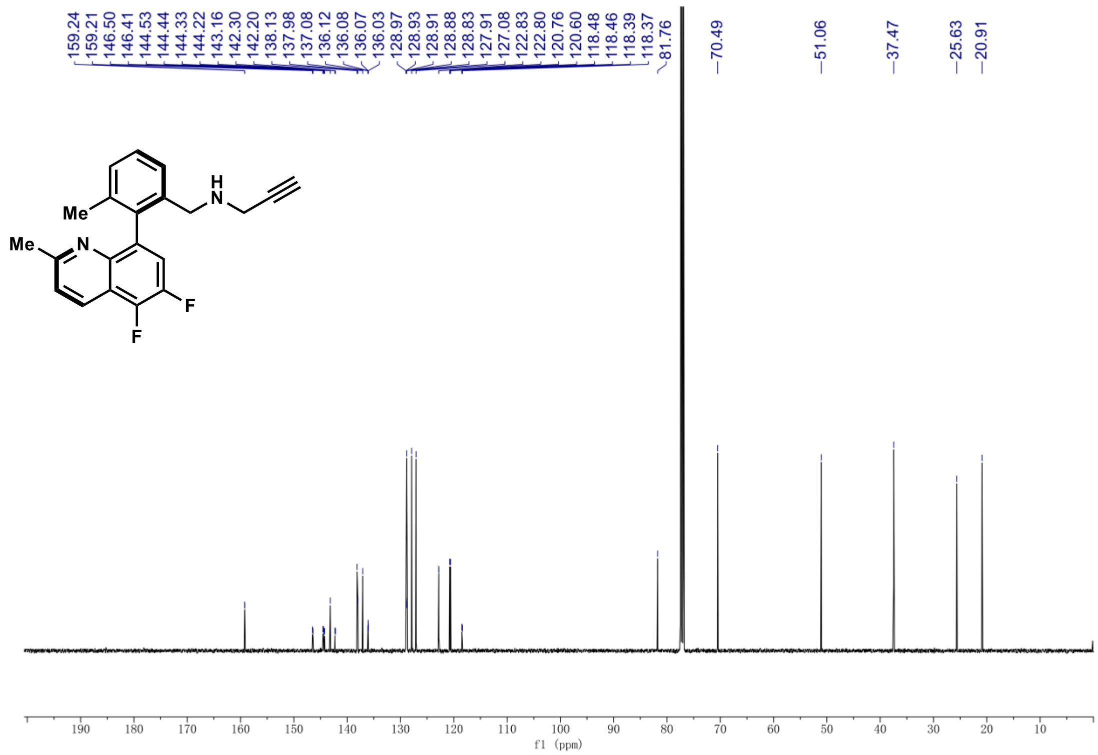

^{19}F NMR (470 MHz, $CDCl_3$) of **5k**

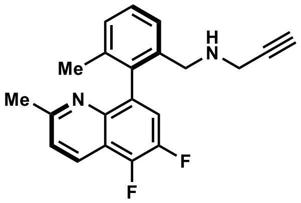

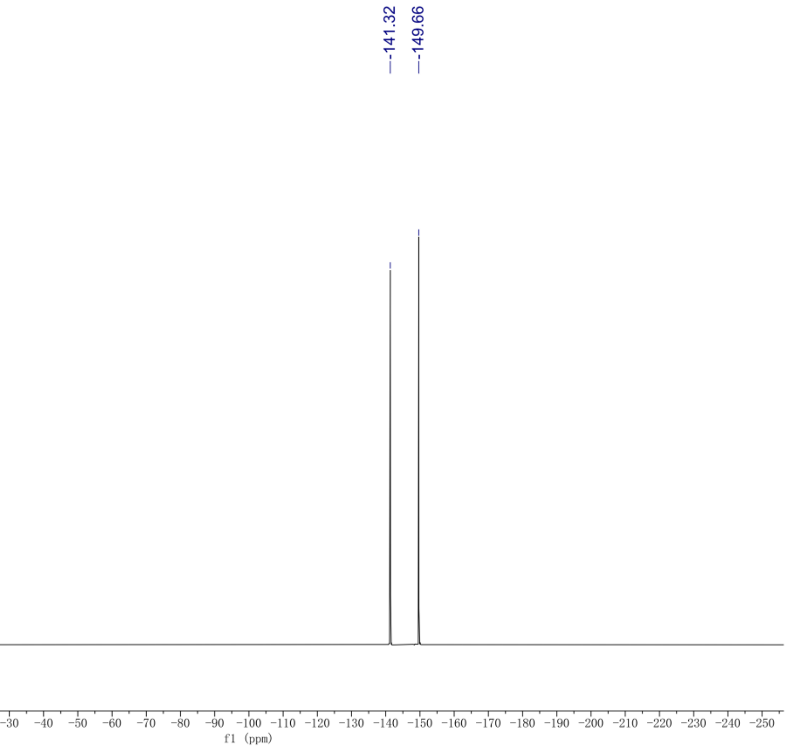

^{1}H NMR (400 MHz, $CDCl_3$) of **5l**

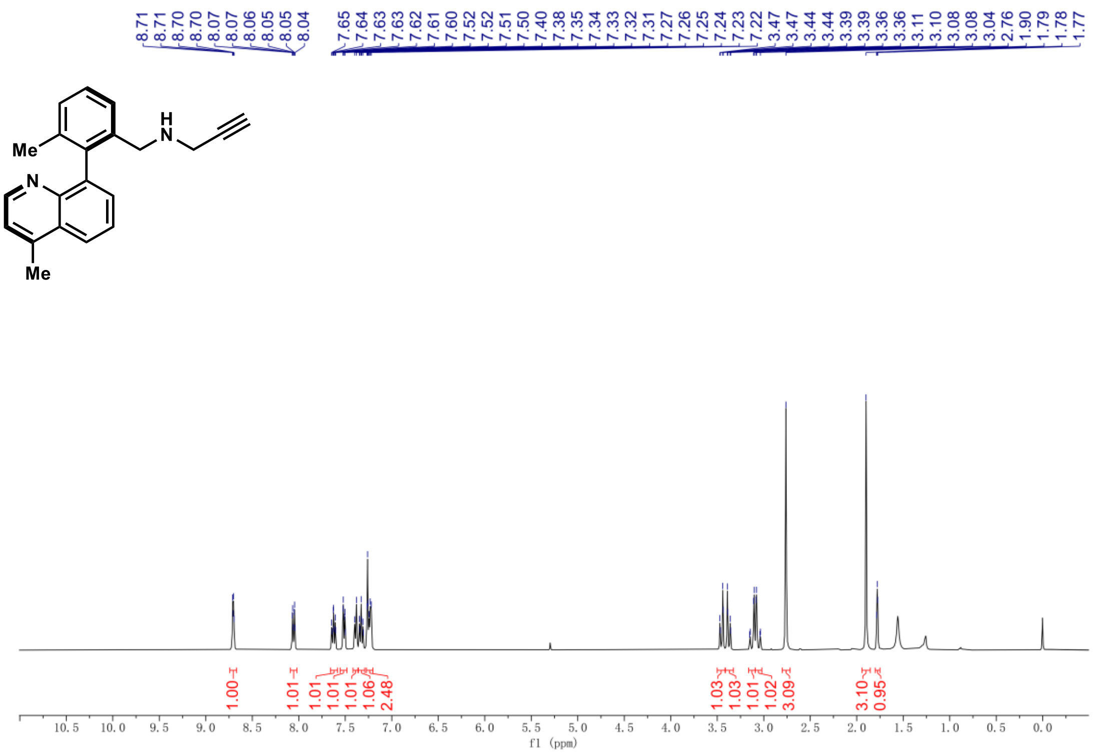

^{13}C NMR (101 MHz, $CDCl_3$) of **5l**

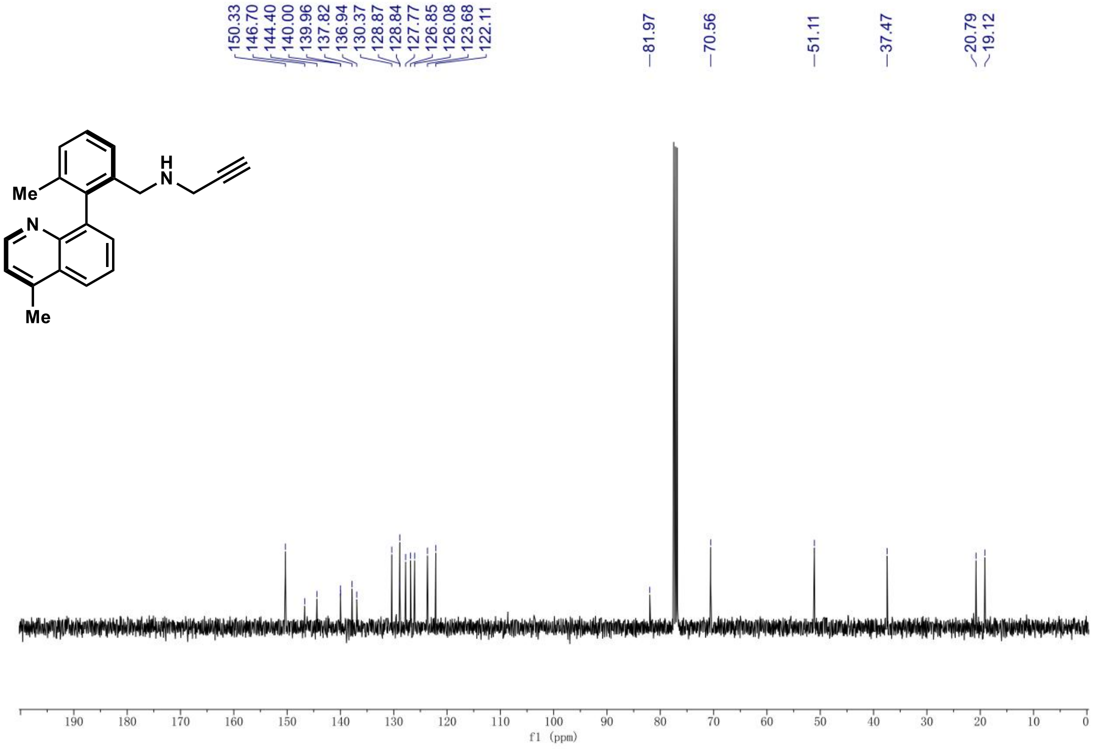

^{1}H NMR (400 MHz, $CDCl_3$) of **5m**

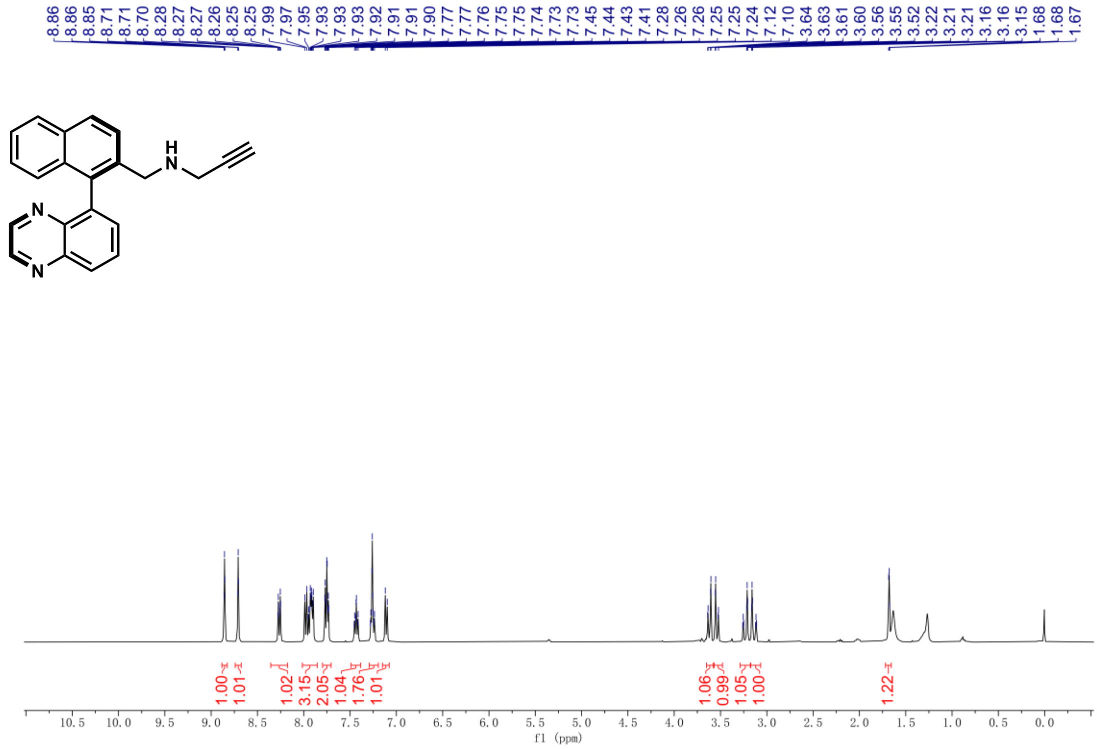

^{13}C NMR (101 MHz, $CDCl_3$) of **5m**

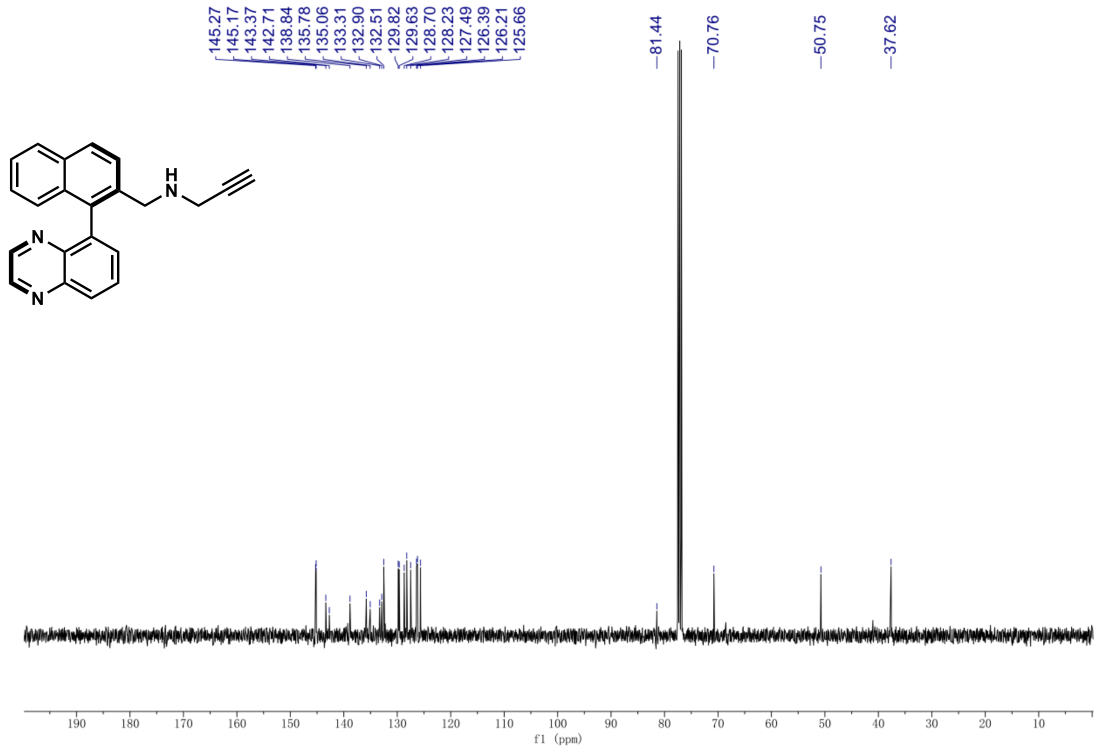

^{1}H NMR (400 MHz, $CDCl_3$) of **5n**

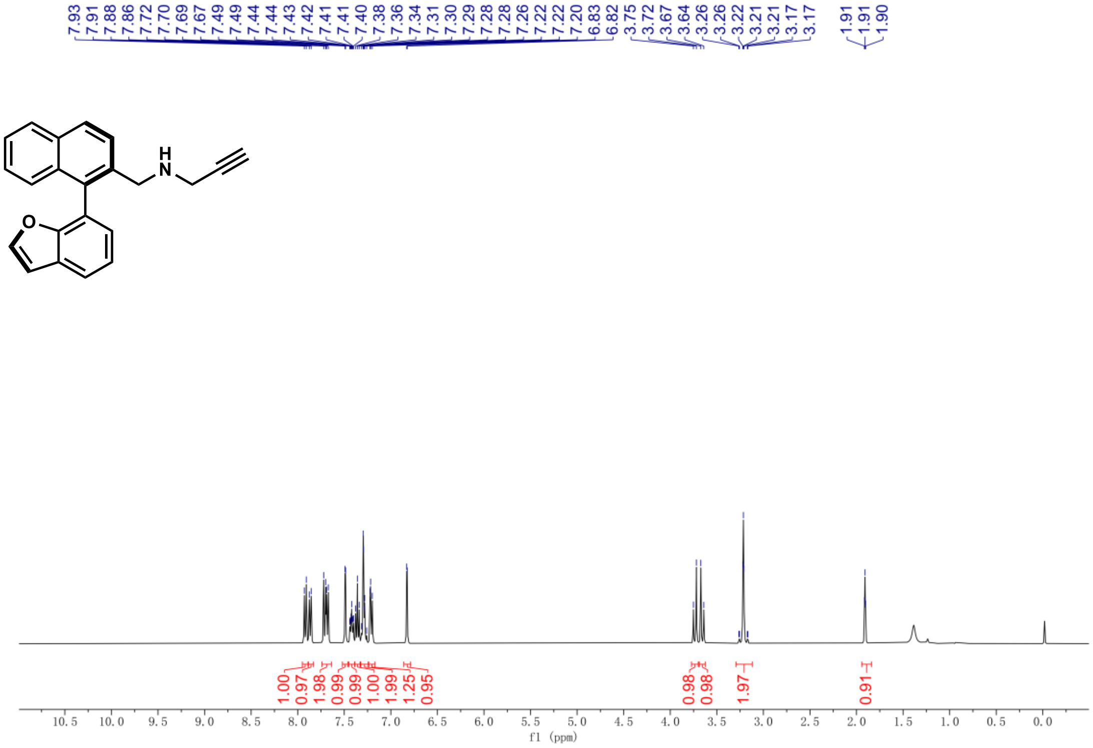

^{13}C NMR (101 MHz, $CDCl_3$) of **5n**

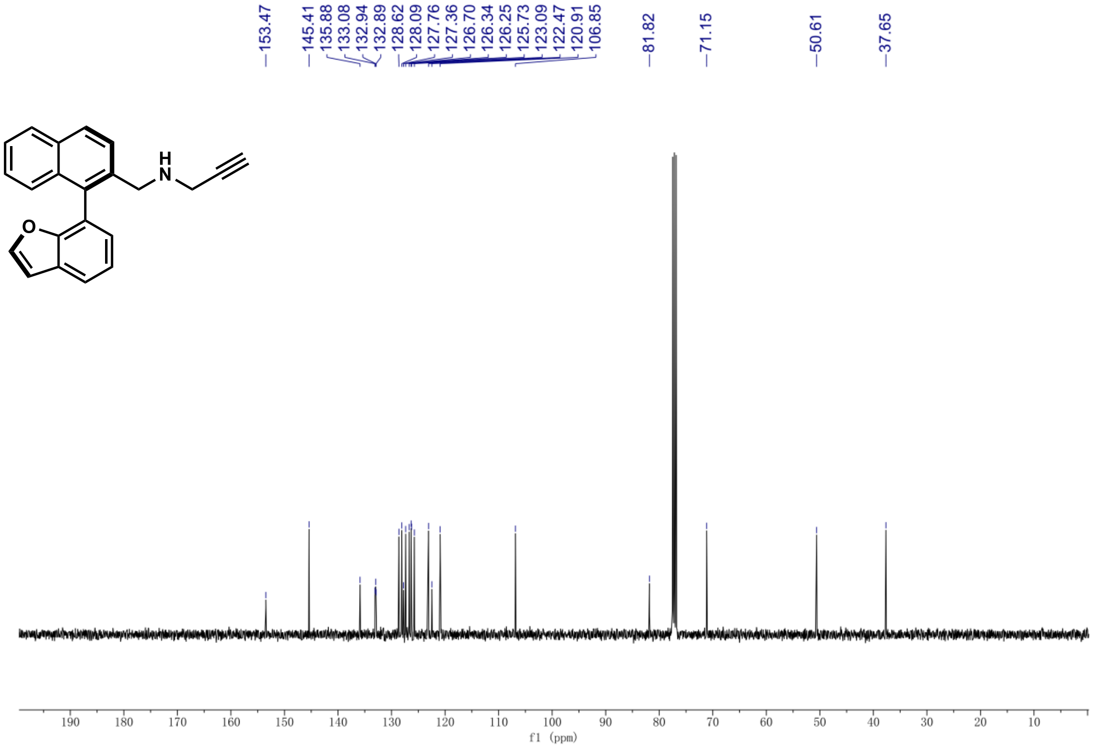

^{1}H NMR (400 MHz, $CDCl_3$) of **5o**

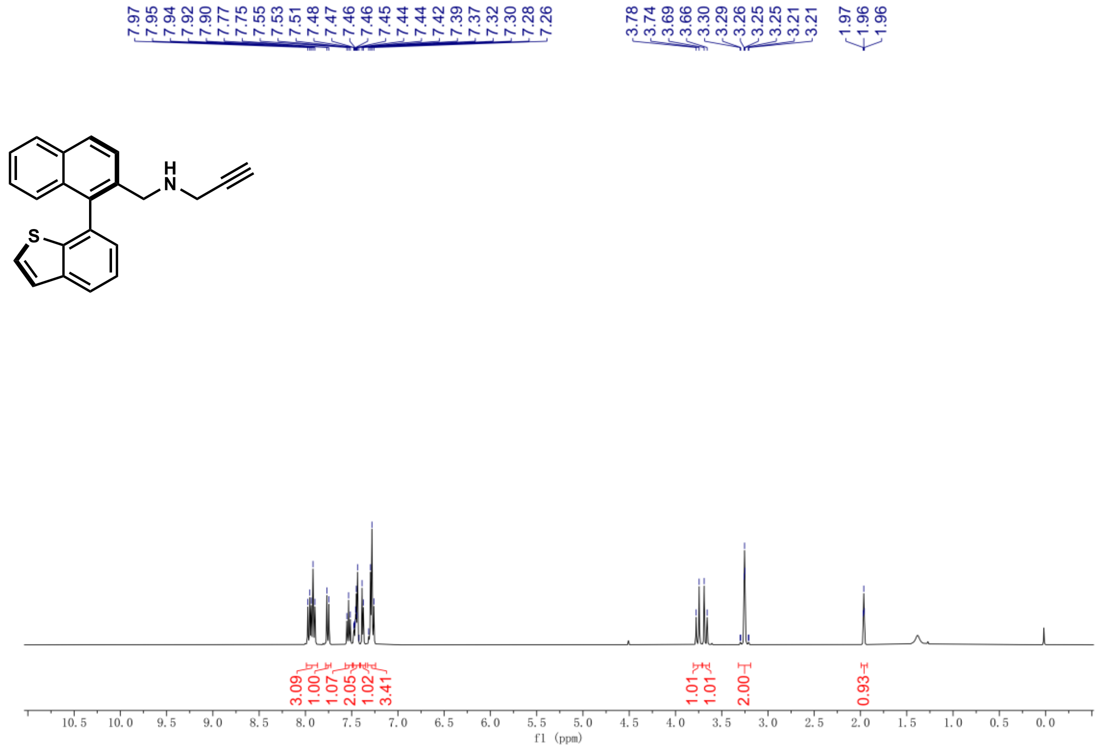

^{13}C NMR (101 MHz, $CDCl_3$) of **5o**

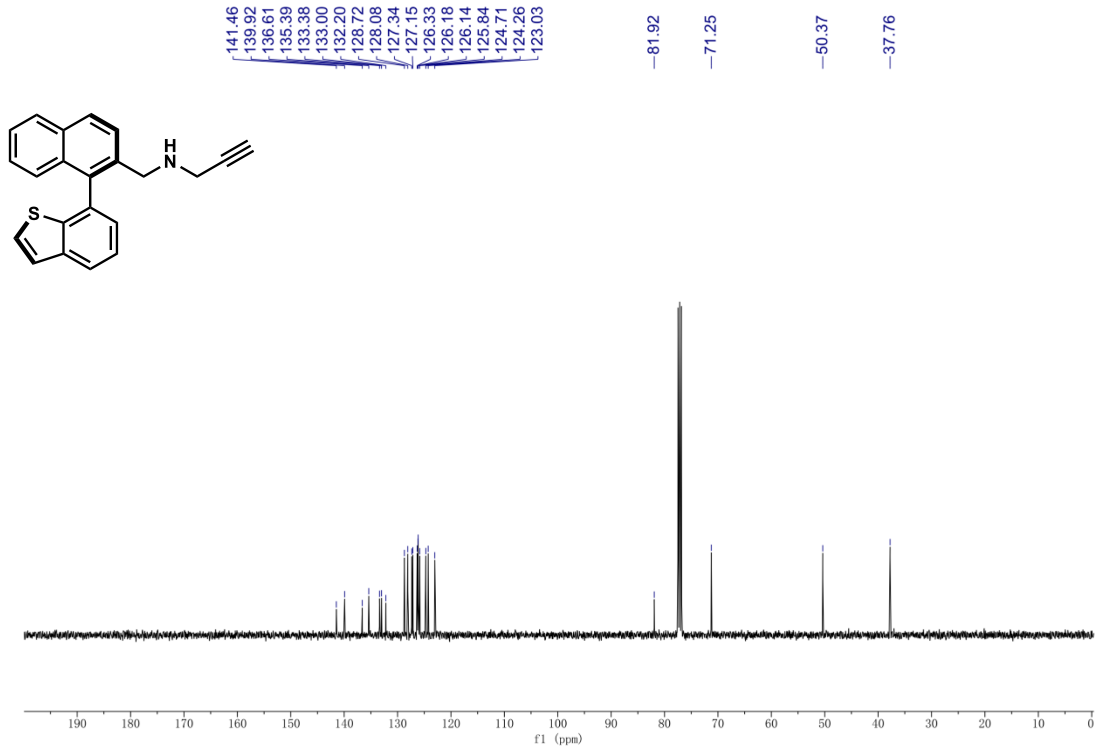

^{1}H NMR (400 MHz, $CDCl_3$) of **5p**

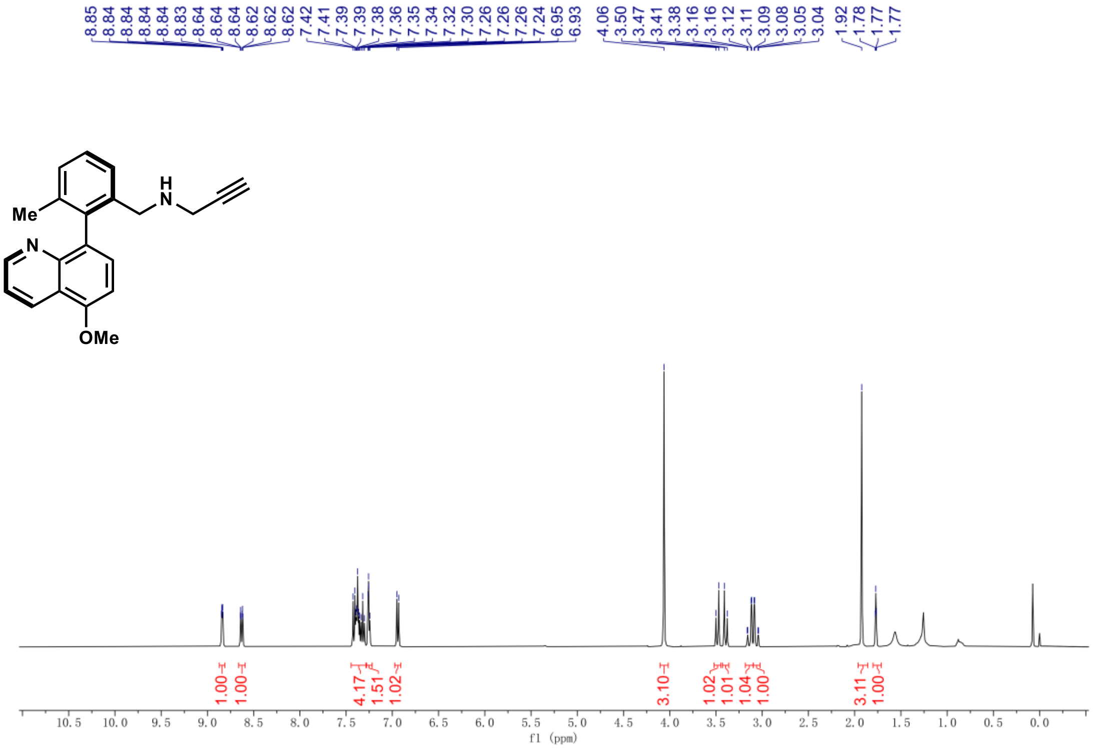

^{13}C NMR (101 MHz, $CDCl_3$) of **5p**

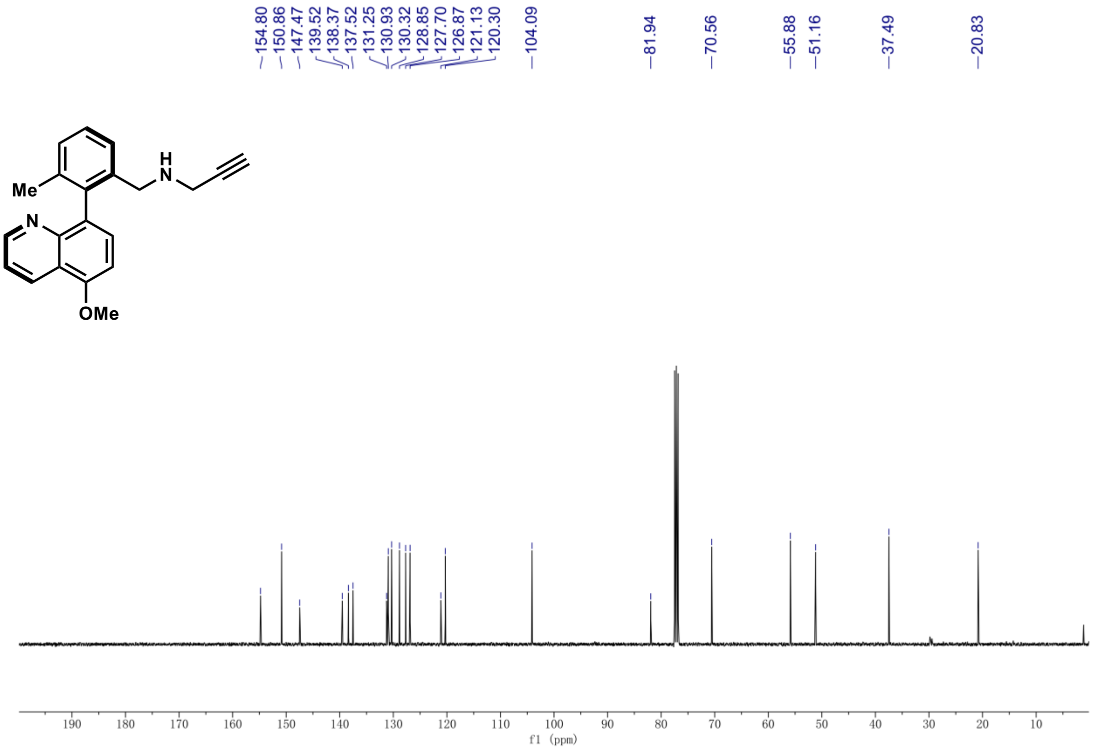

^{1}H NMR (400 MHz, $CDCl_3$) of **6a**

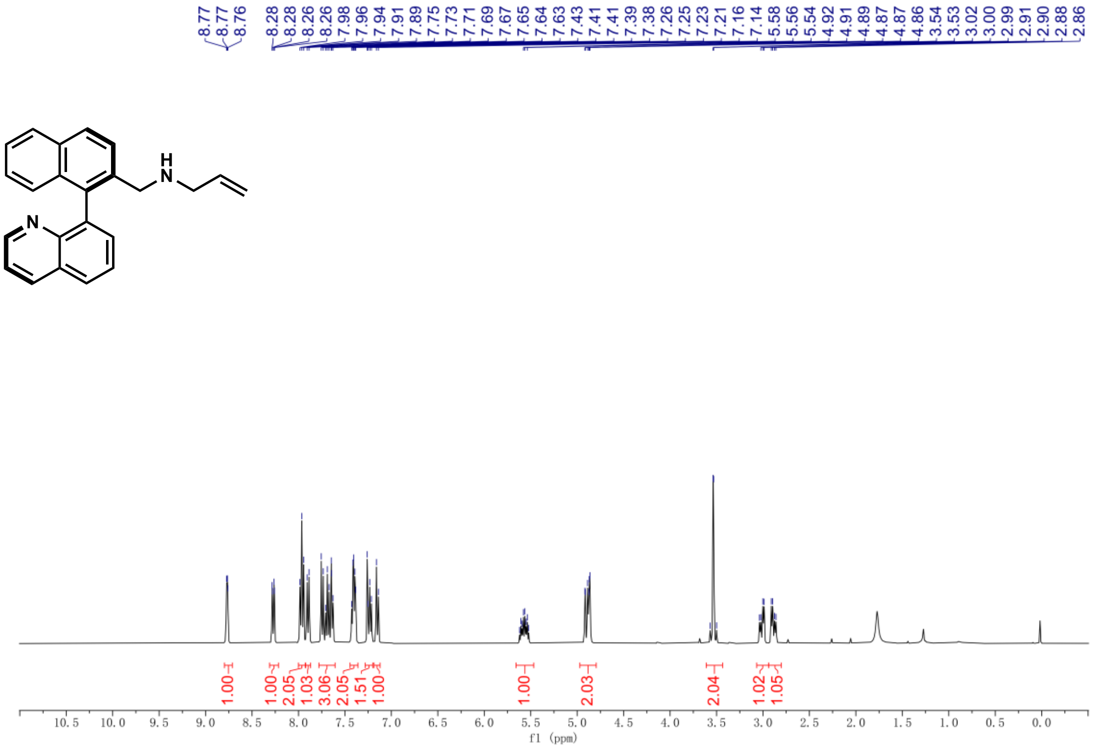

^{13}C NMR (126 MHz, $CDCl_3$) of **6a**

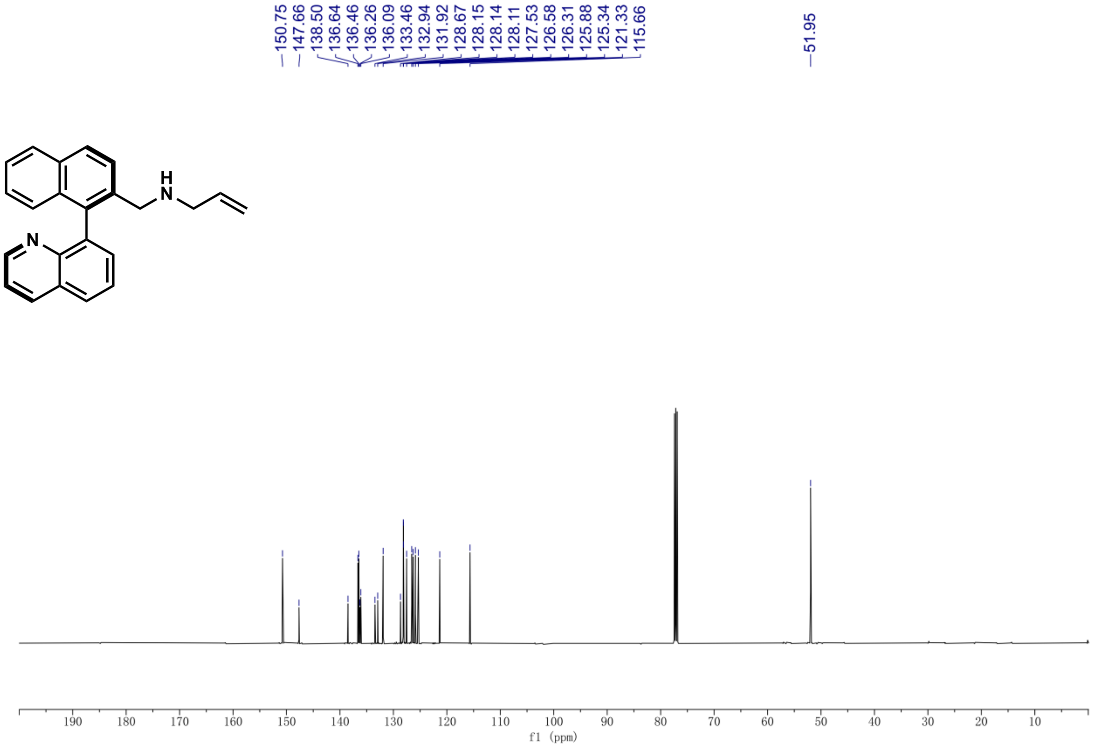

^{1}H NMR (400 MHz, $CDCl_3$) of **6b**

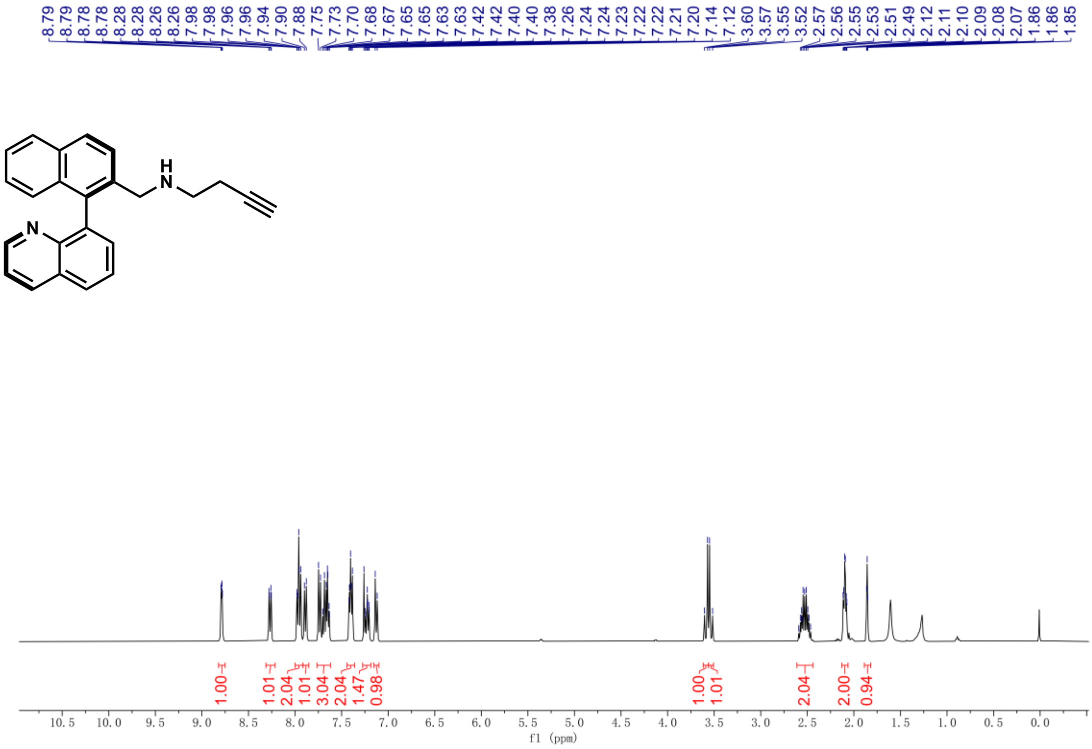

^{13}C NMR (101 MHz, $CDCl_3$) of **6b**

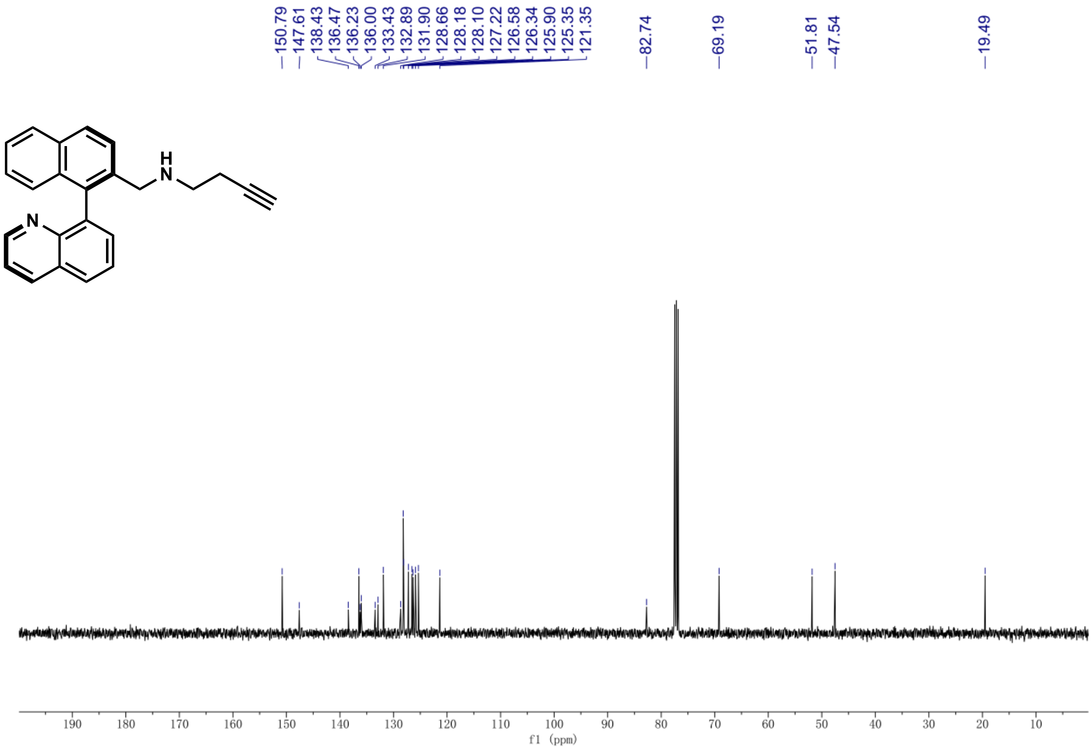

^{1}H NMR (500 MHz, CDCl$_3$) of **6c**

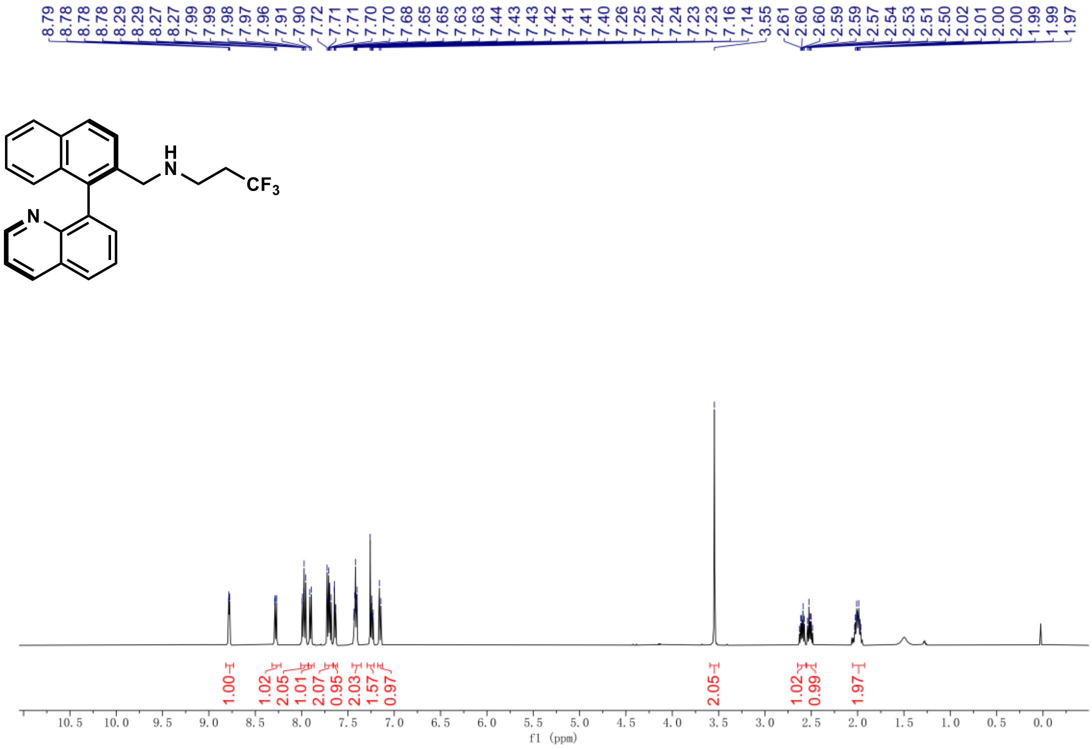

^{13}C NMR (126 MHz, CDCl$_3$) of **6c**

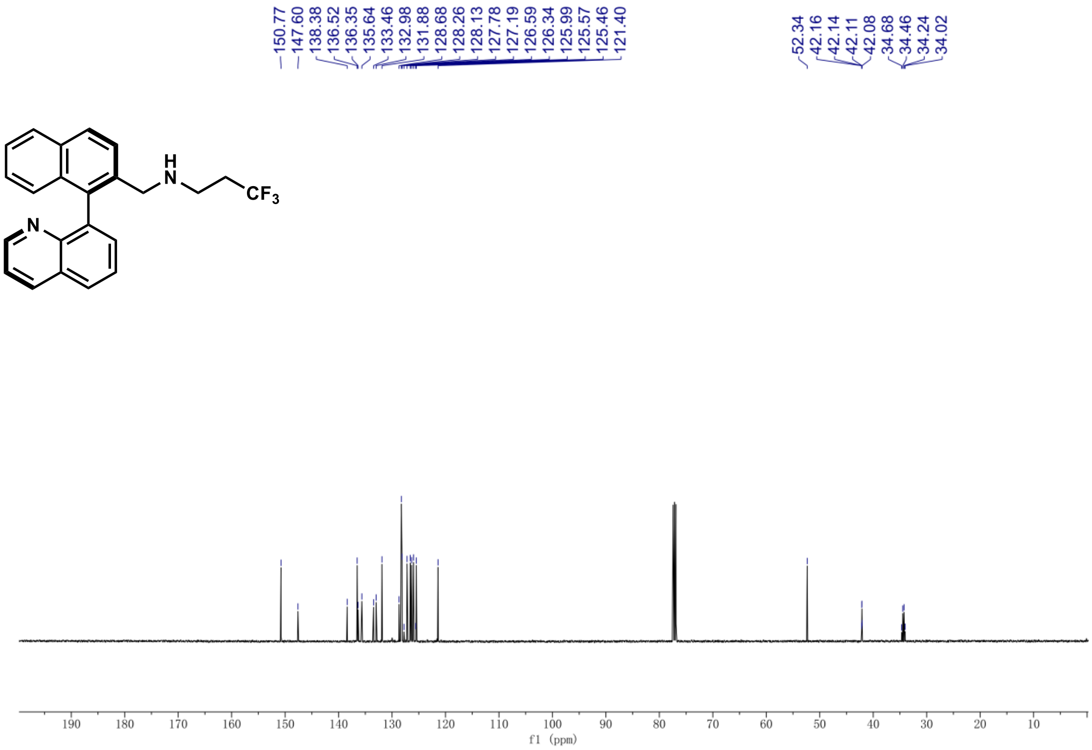

^{19}F NMR (470 MHz, $CDCl_3$) of **6c**

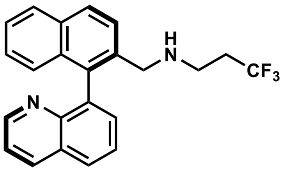

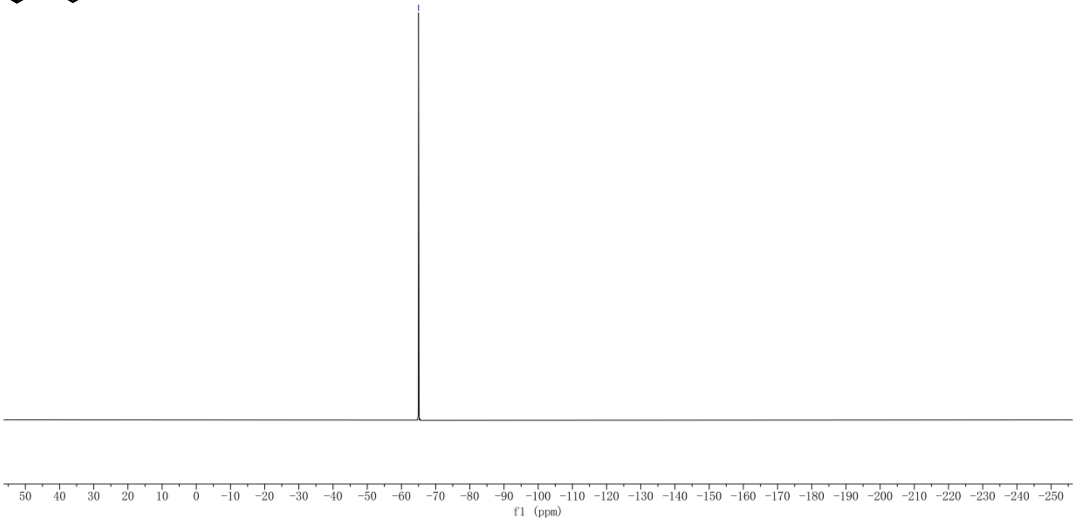

^{1}H NMR (400 MHz, $CDCl_3$) of **6d**

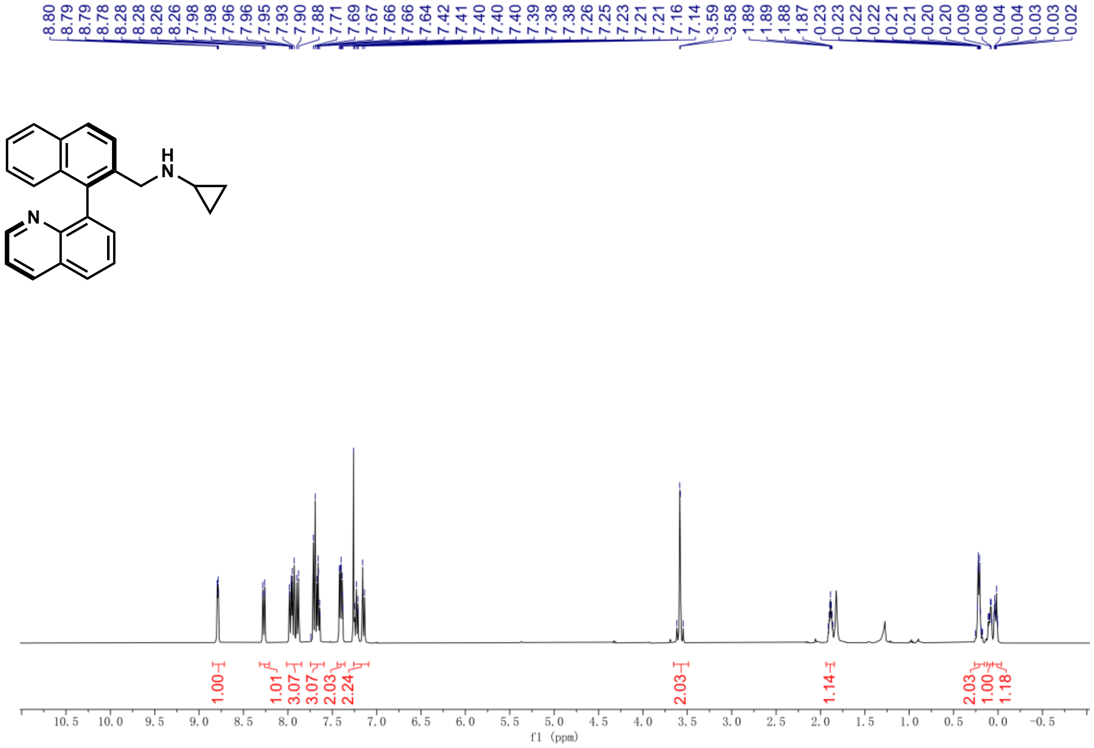

^{13}C NMR (101 MHz, $CDCl_3$) of **6d**

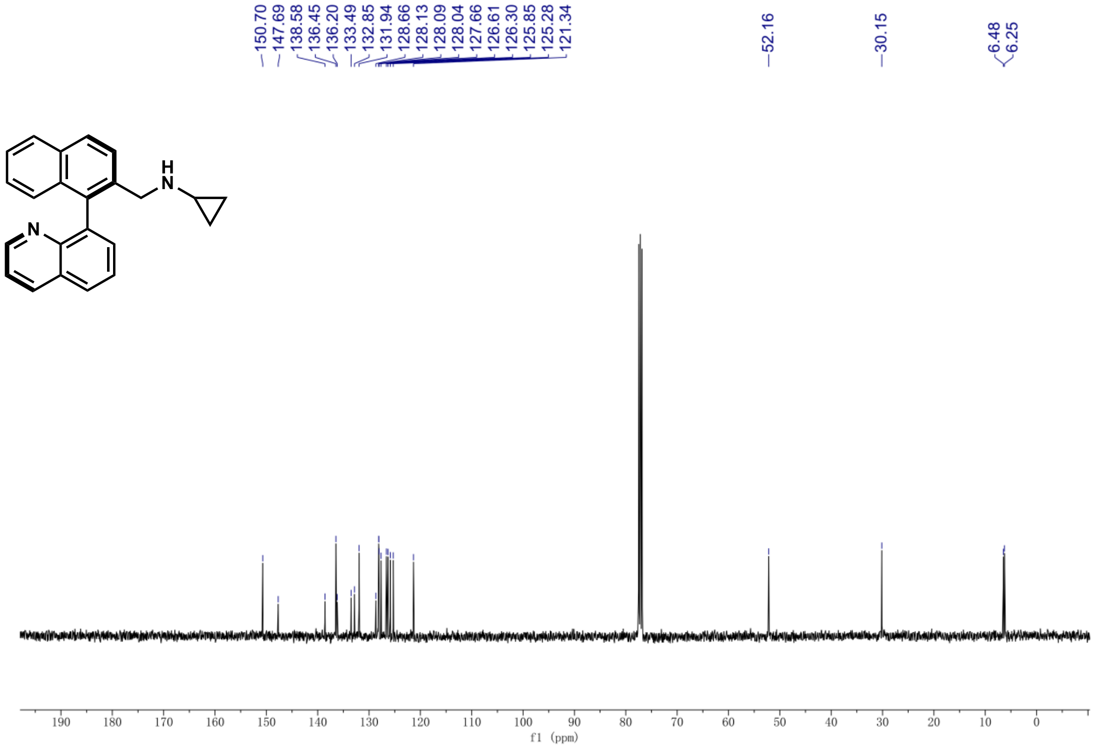

^{1}H NMR (400 MHz, $CDCl_3$) of **6e**

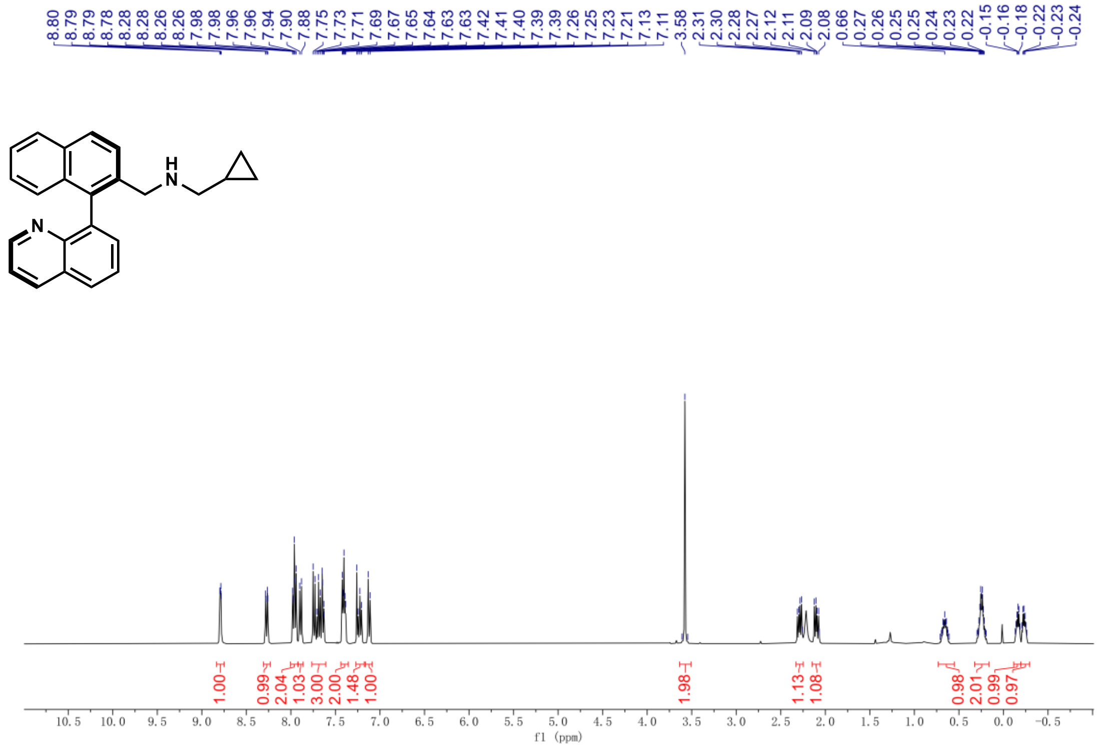

^{13}C NMR (101 MHz, $CDCl_3$) of **6e**

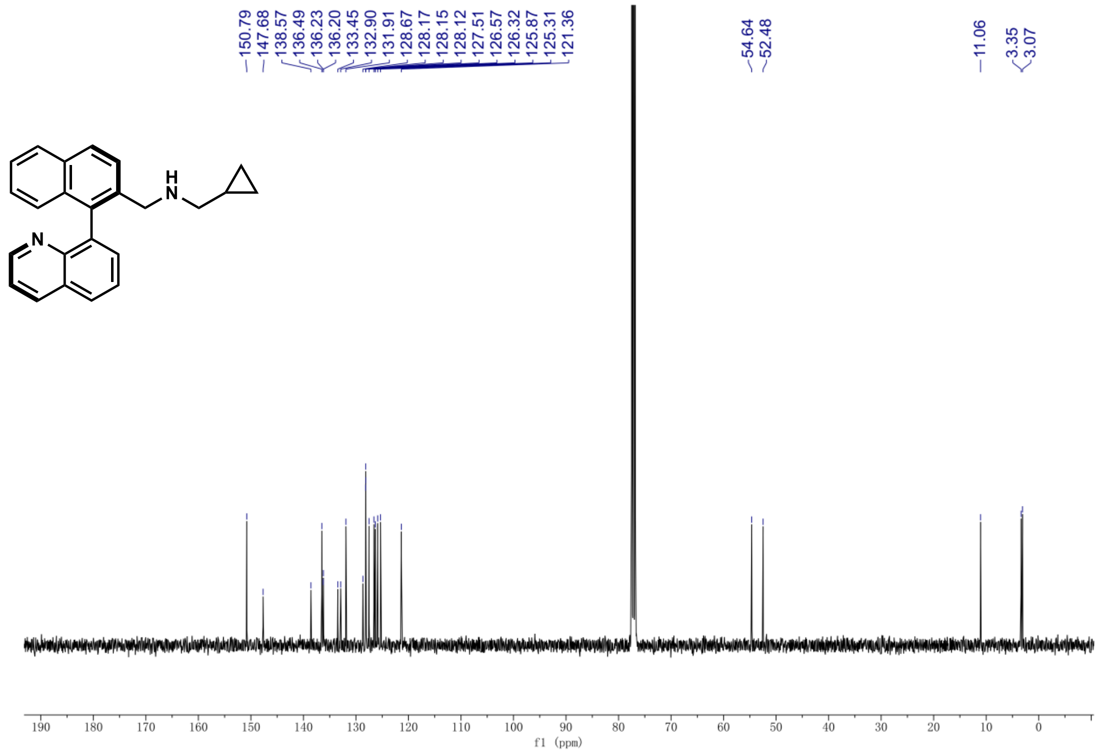

^{1}H NMR (400 MHz, CDCl$_3$) of **6f**

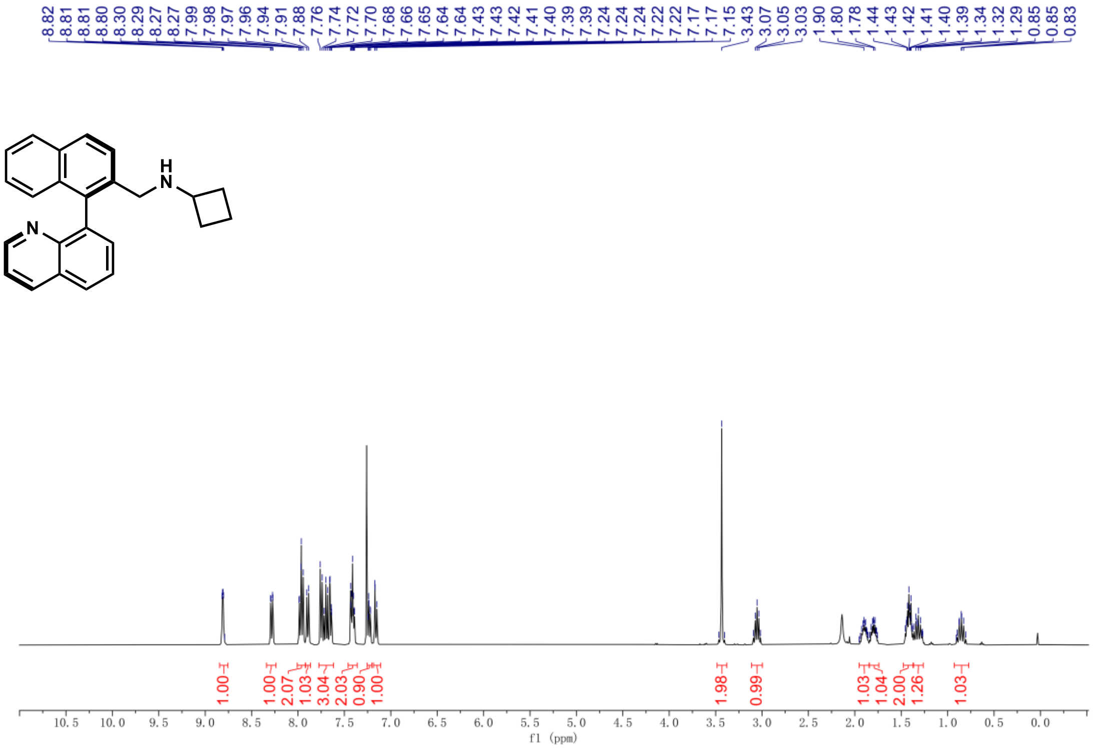

^{13}C NMR (101 MHz, CDCl$_3$) of **6f**

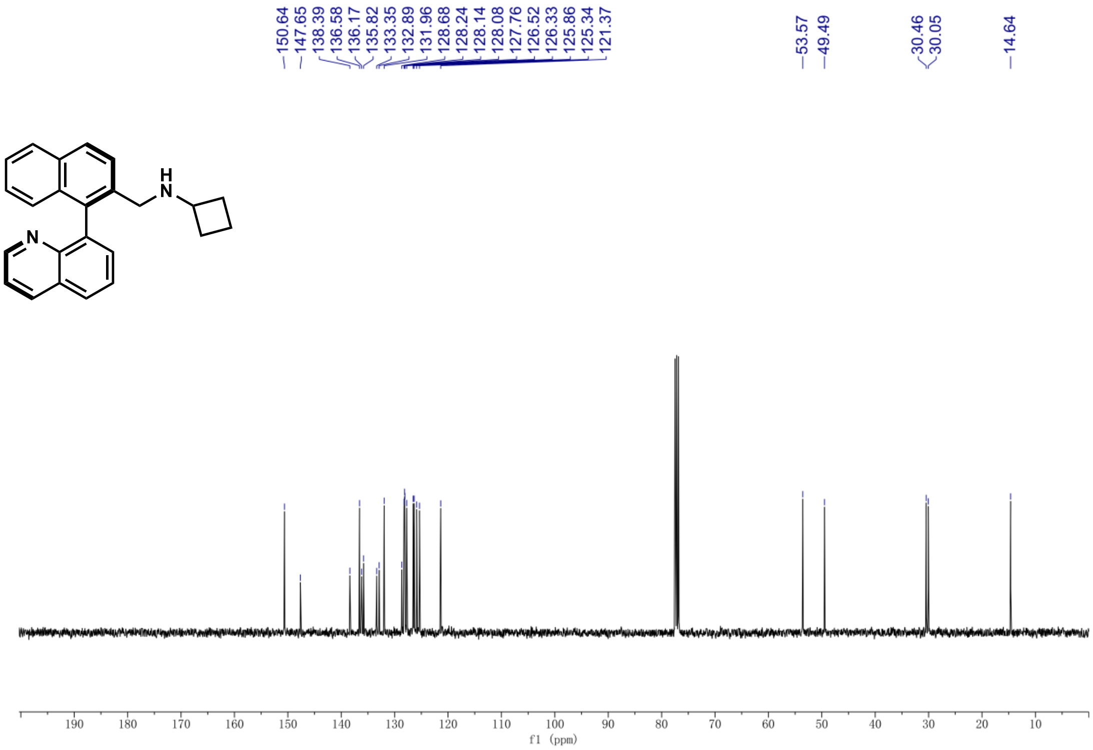

^{1}H NMR (400 MHz, $CDCl_3$) of **6g**

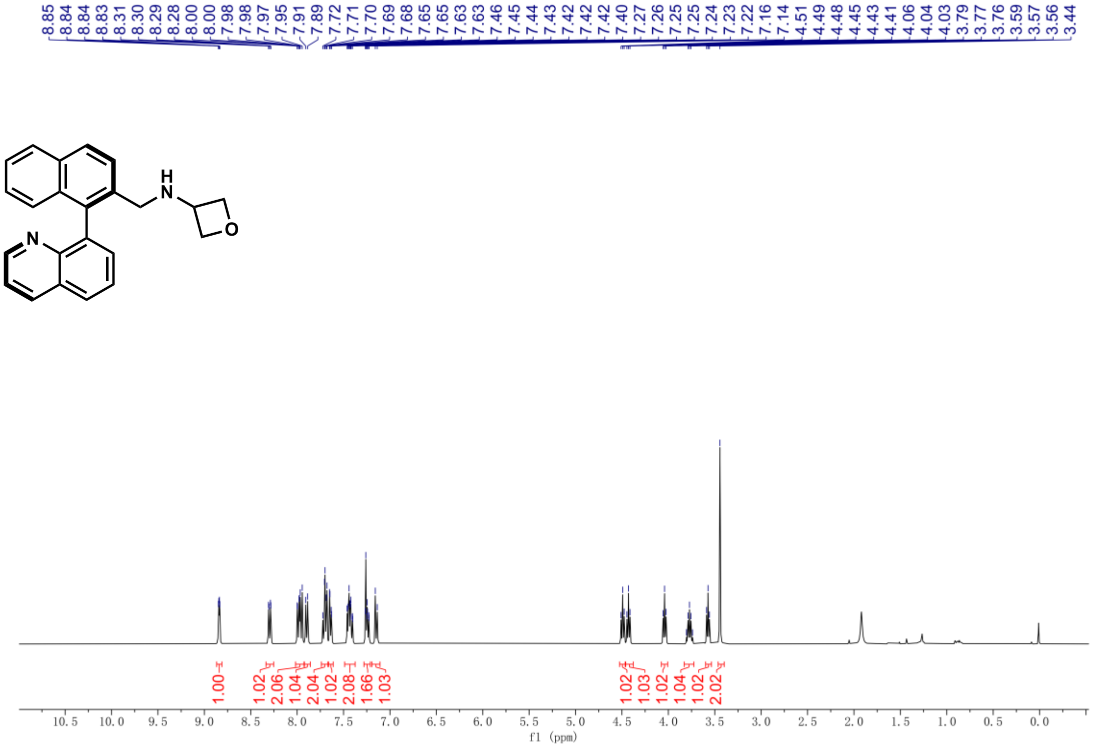

^{13}C NMR (101 MHz, $CDCl_3$) of **6g**

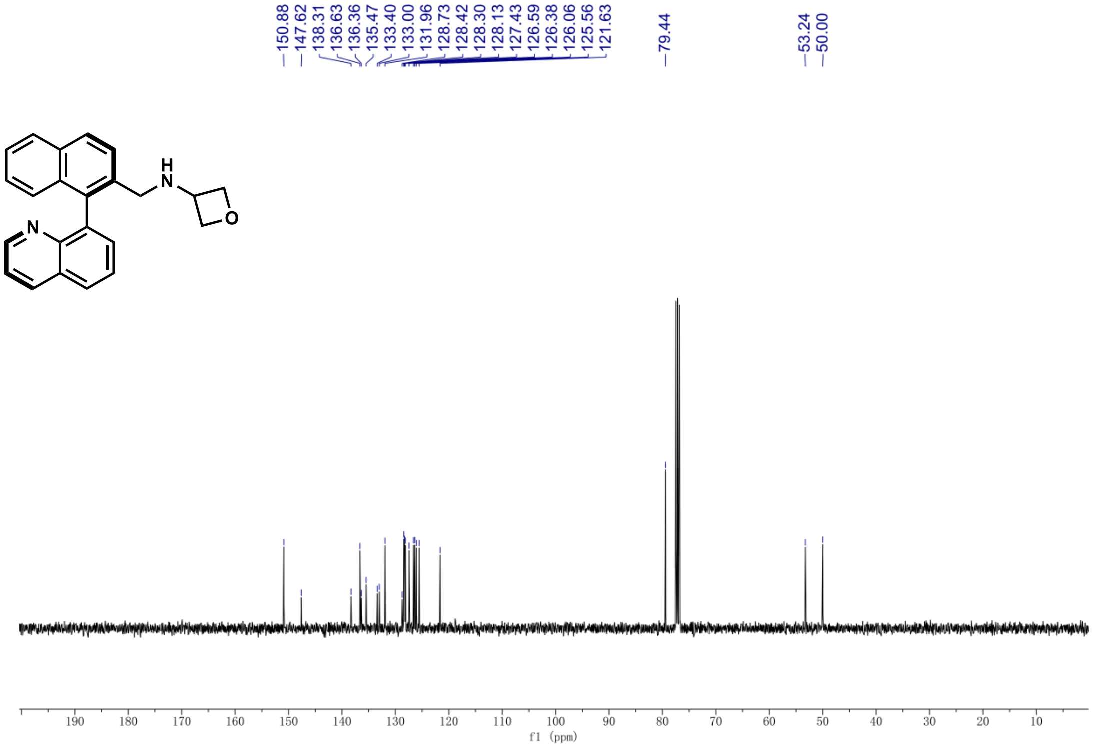

^{1}H NMR (400 MHz, $CDCl_3$) of **6h**

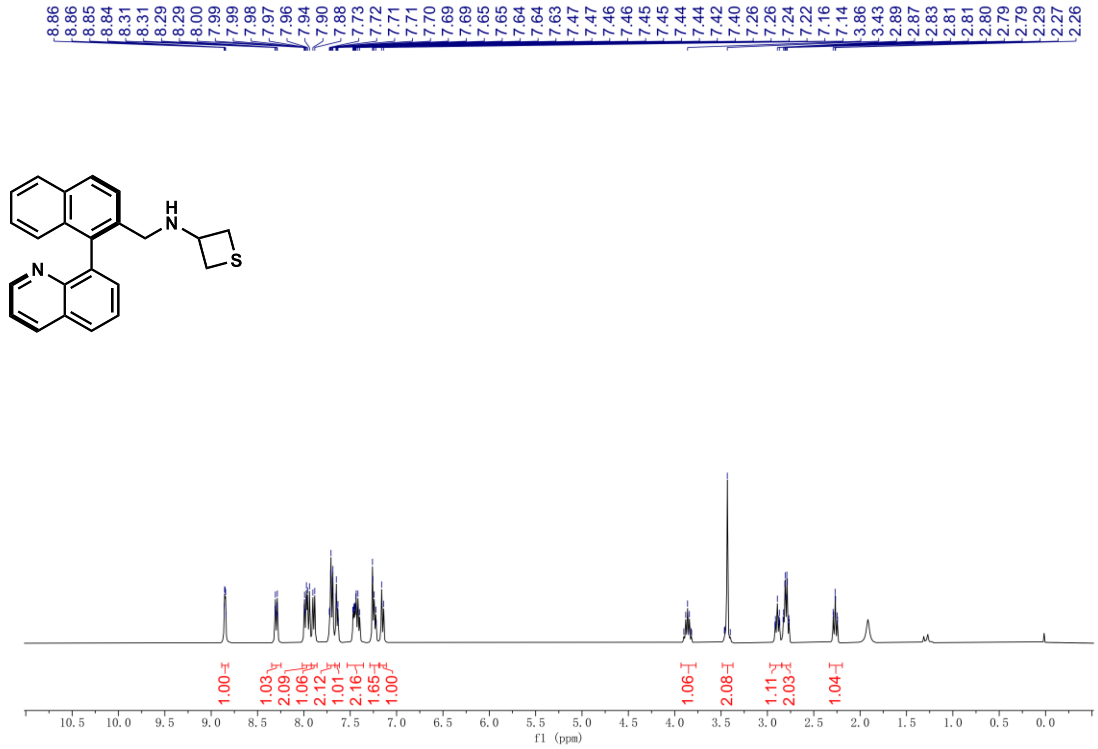

^{13}C NMR (101 MHz, $CDCl_3$) of **6h**

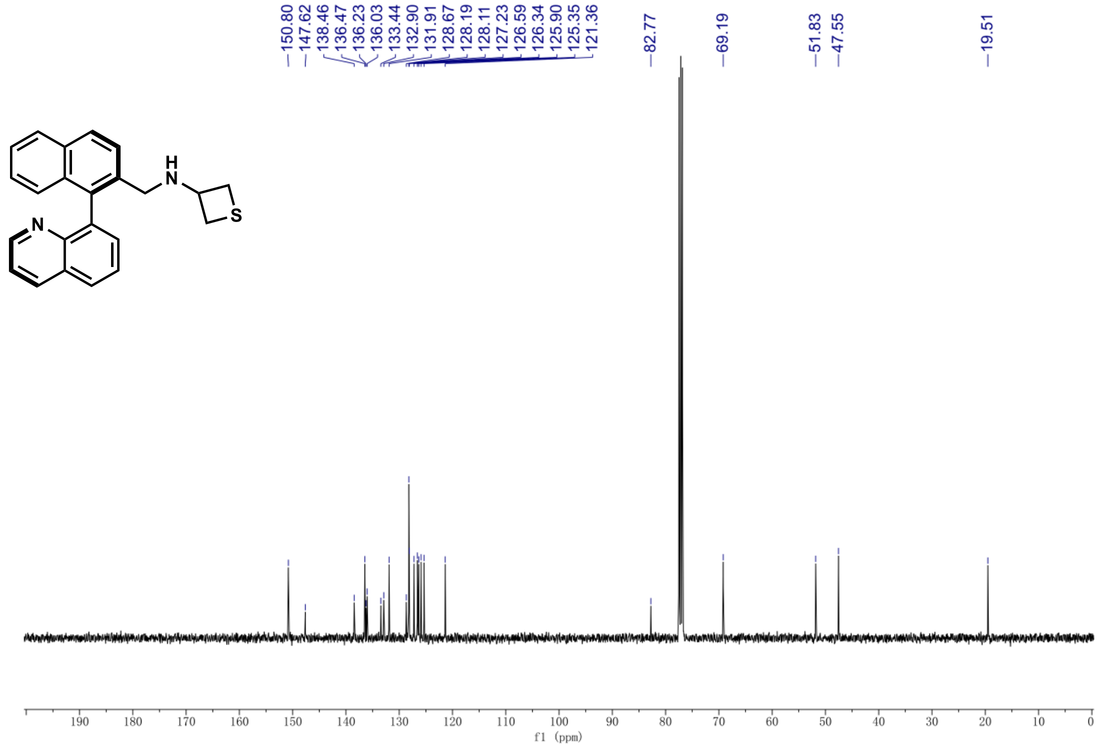

^{1}H NMR (400 MHz, $CDCl_3$) of **6i**

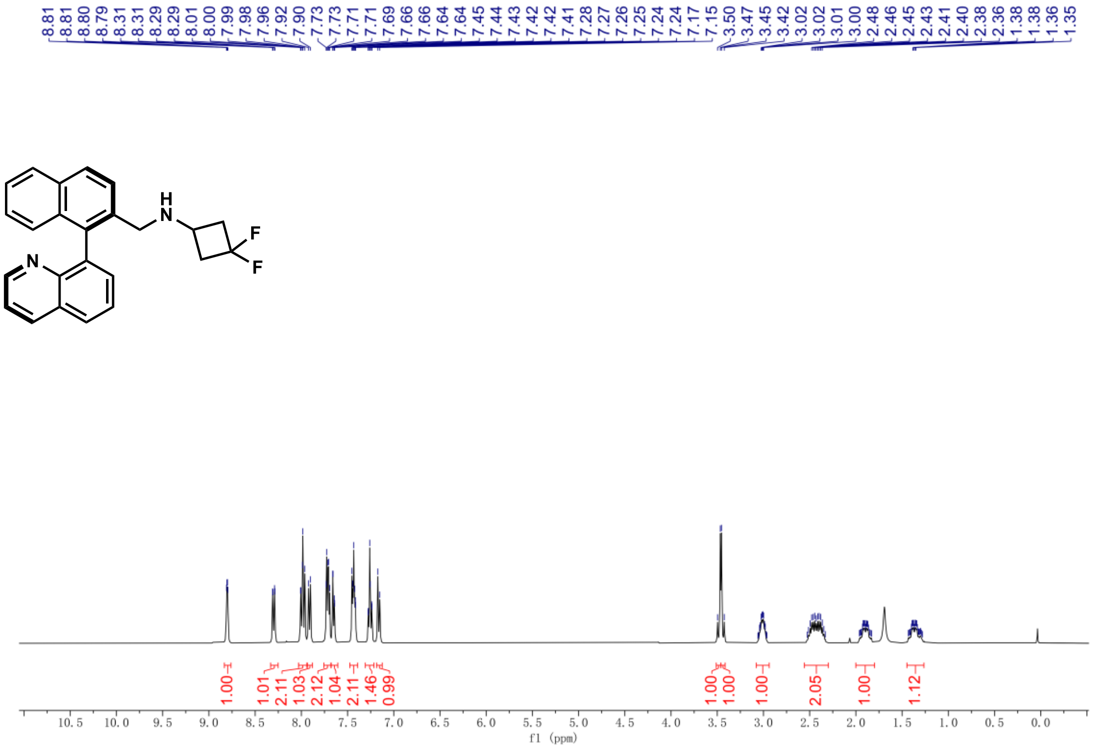

^{13}C NMR (126 MHz, $CDCl_3$) of **6i**

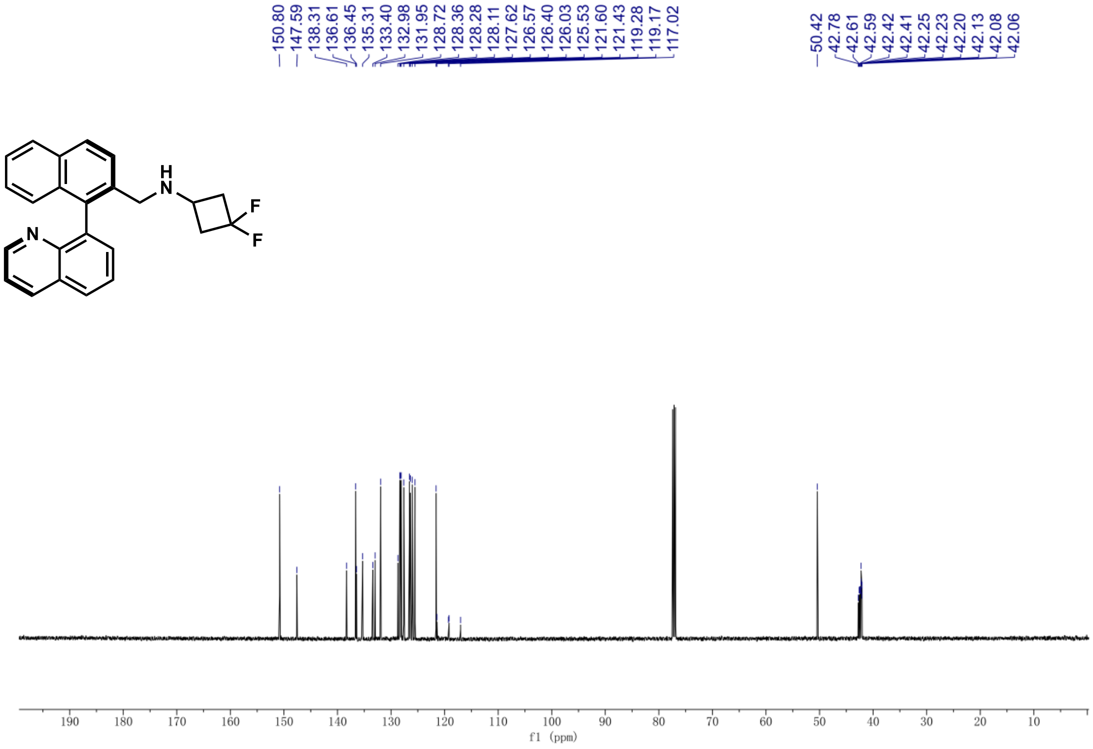

^{19}F NMR (470 MHz, $CDCl_3$) of **6i**

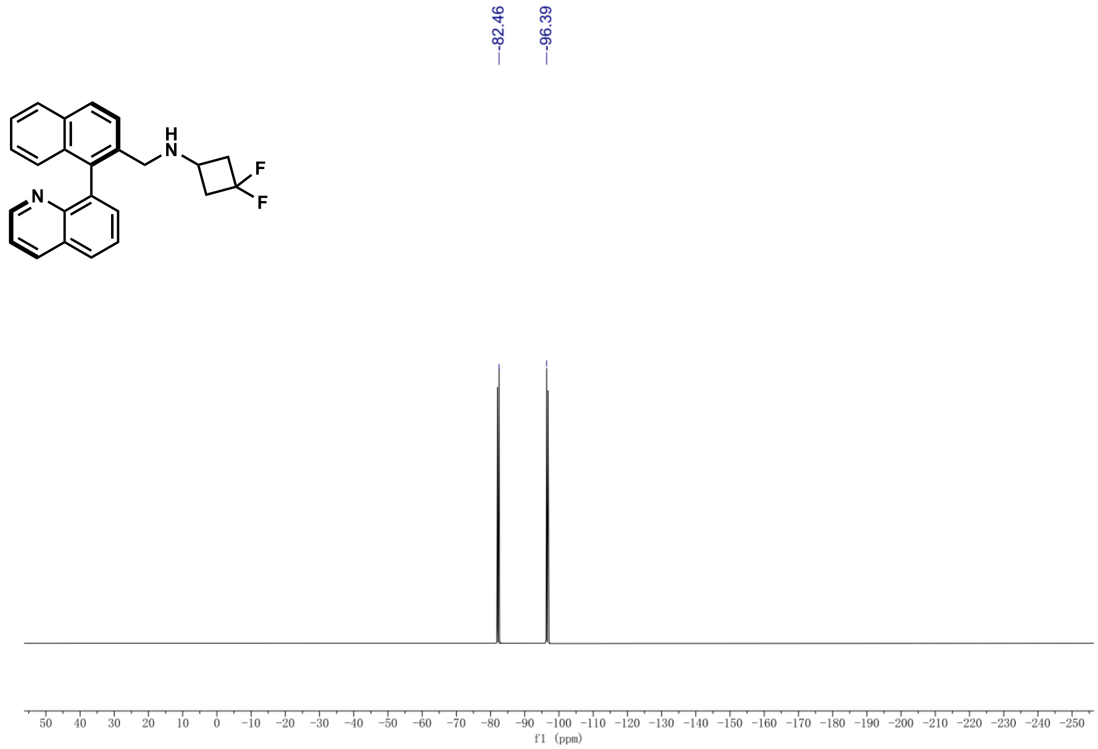

^{1}H NMR (400 MHz, $CDCl_3$) of **7a**

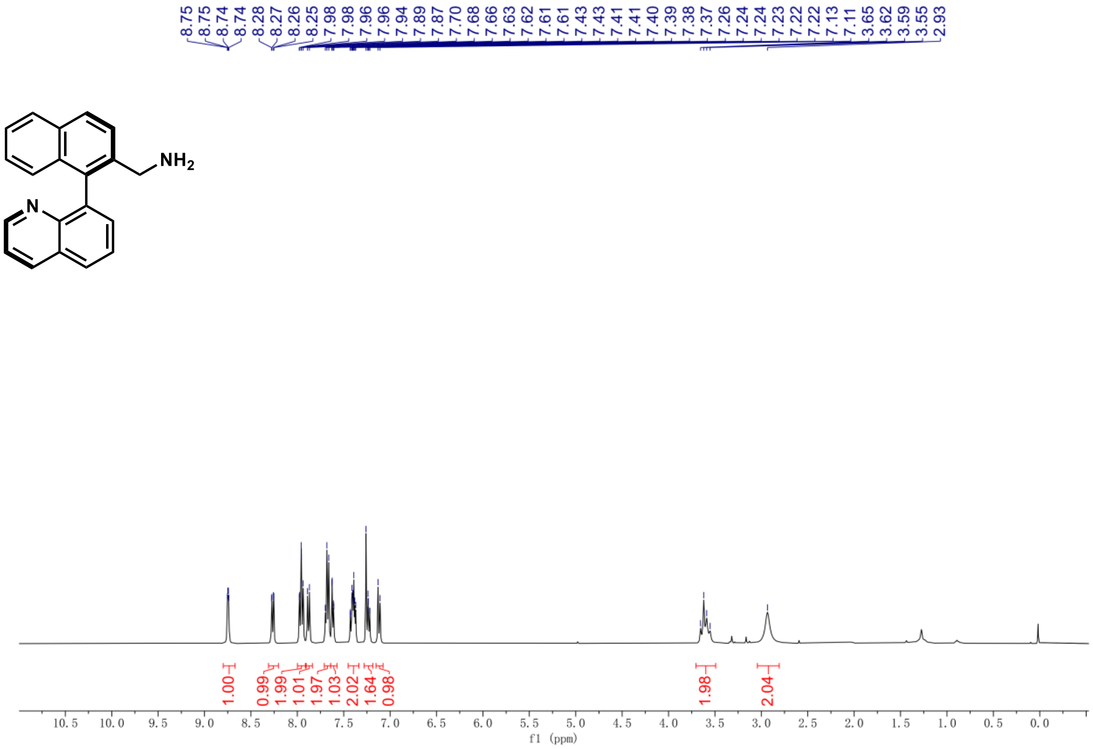

^{13}C NMR (101 MHz, $CDCl_3$) of **7a**

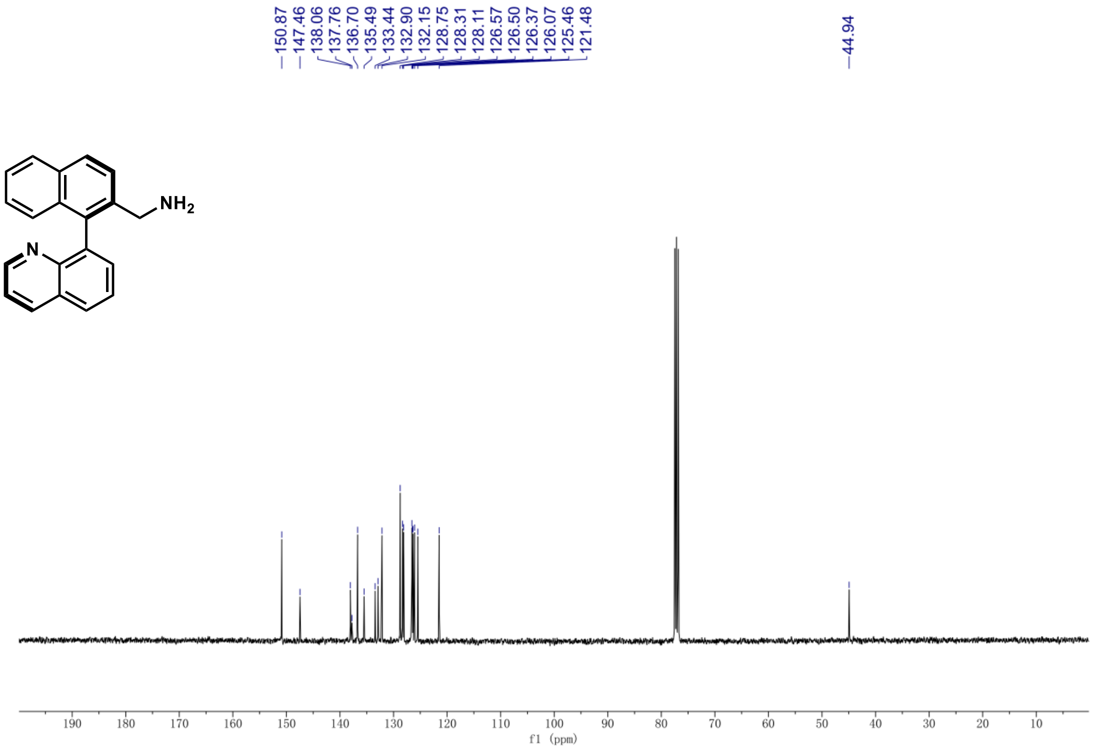

^{1}H NMR (400 MHz, $CDCl_3$) of **8a**

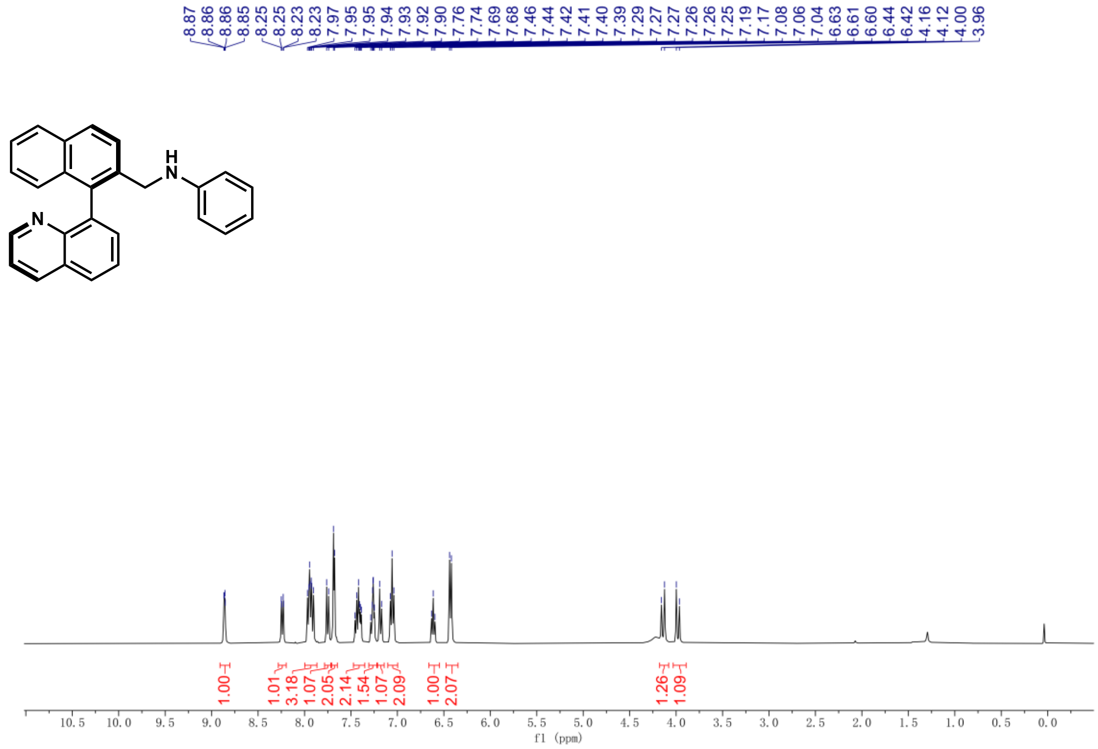

^{13}C NMR (101 MHz, $CDCl_3$) of **8a**

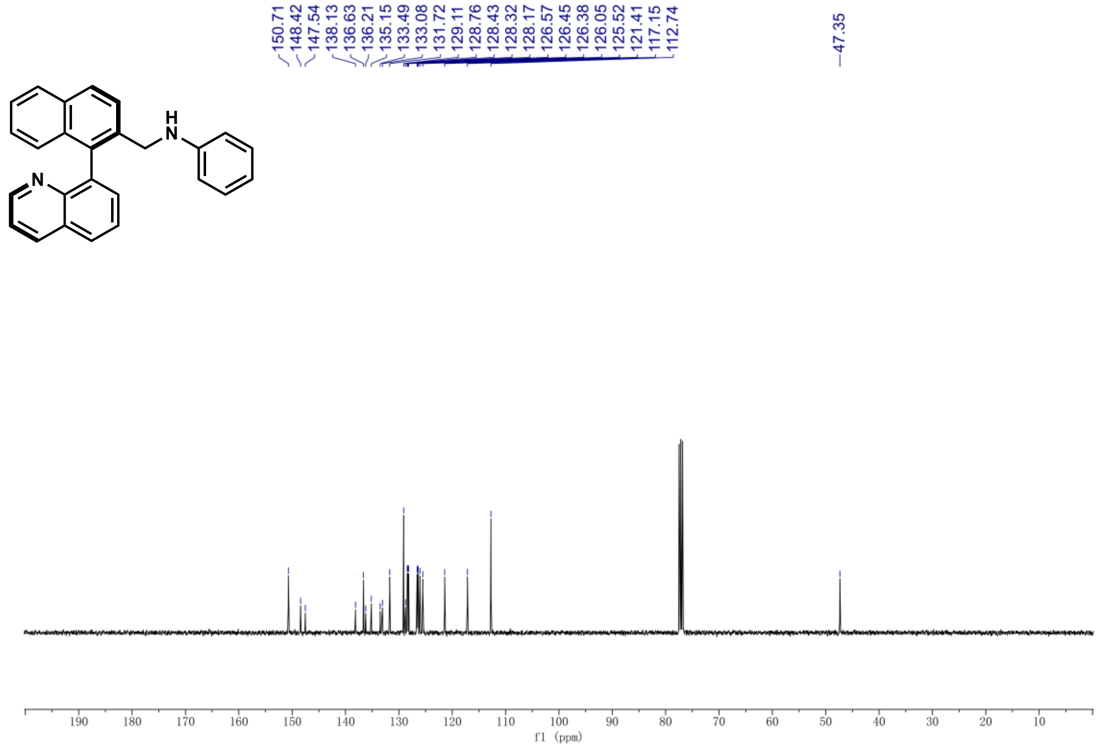

^{1}H NMR (400 MHz, $CDCl_3$) of **8b**

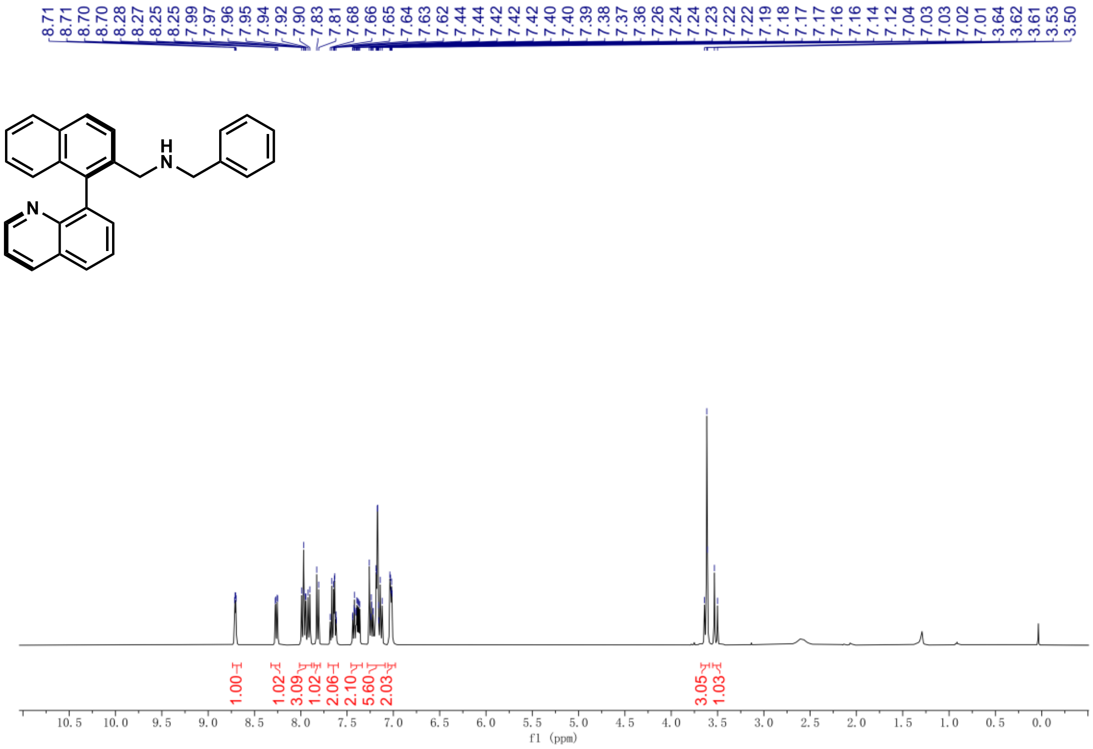

^{13}C NMR (101 MHz, $CDCl_3$) of **8b**

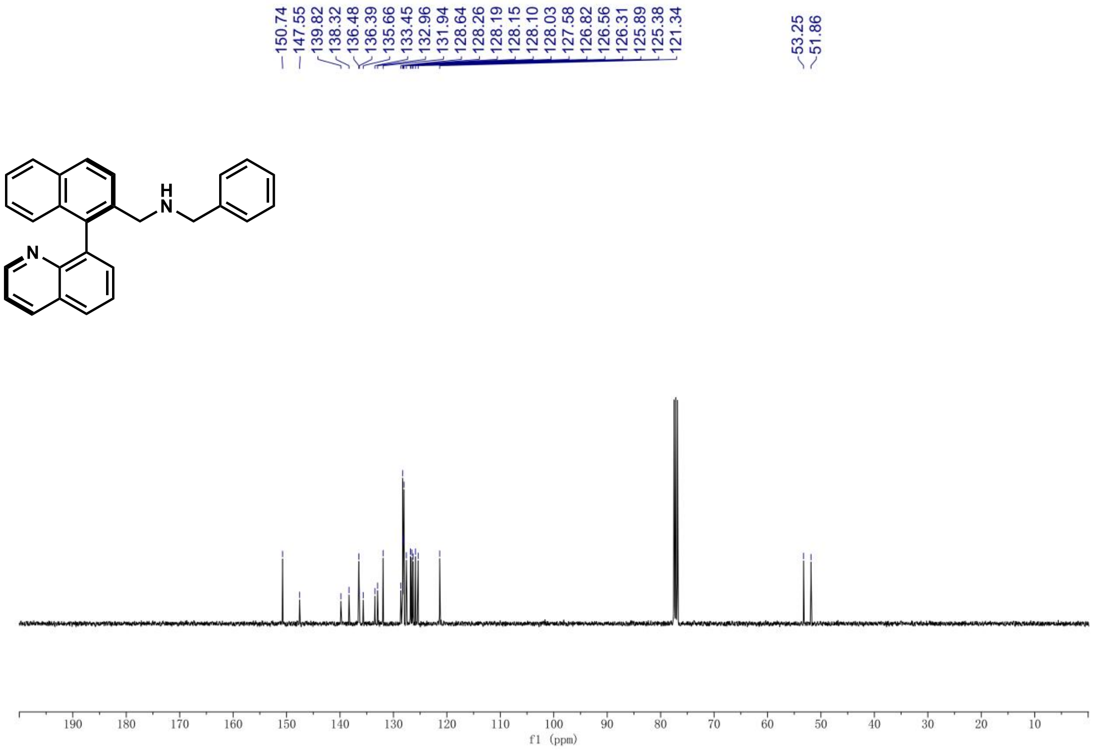

^{1}H NMR (400 MHz, $CDCl_3$) of **8c**

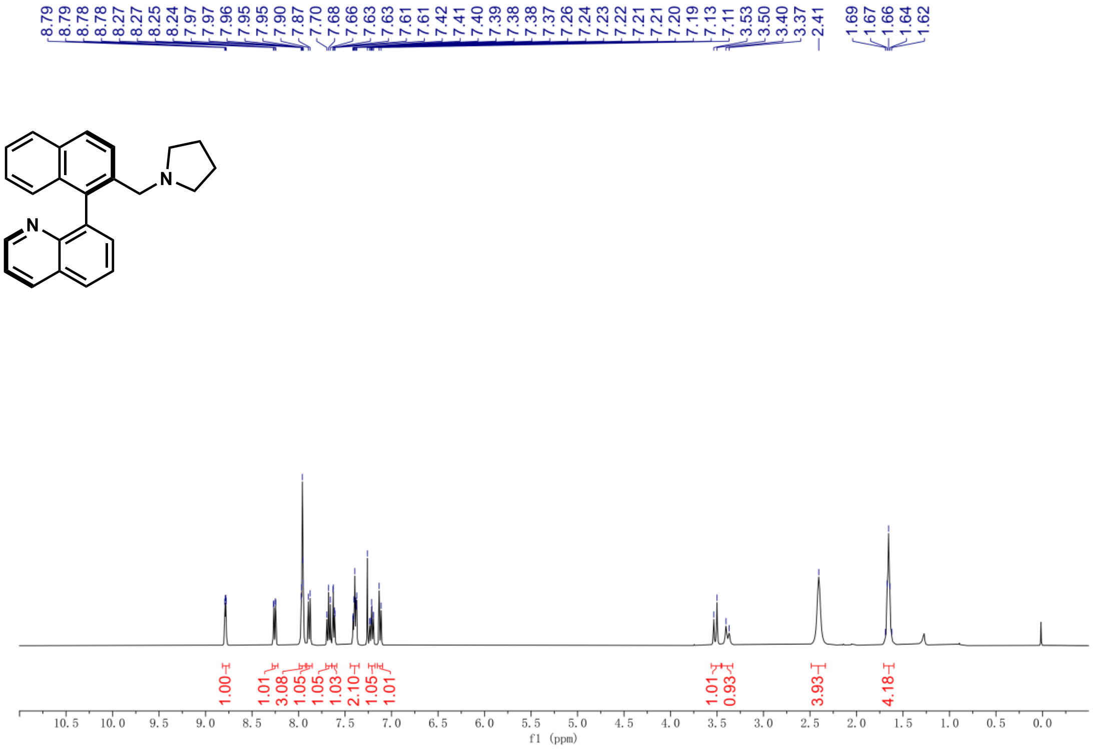

^{13}C NMR (101 MHz, $CDCl_3$) of **8c**

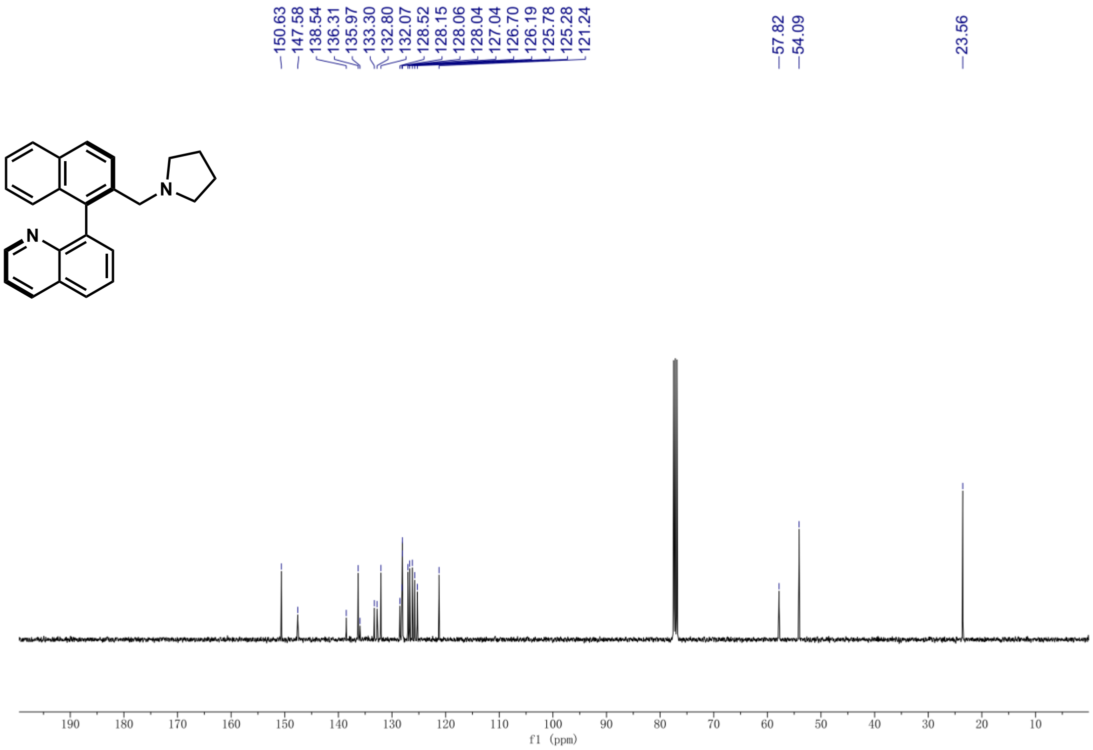

^{1}H NMR (500 MHz, DMSO-d_6) of **8d**

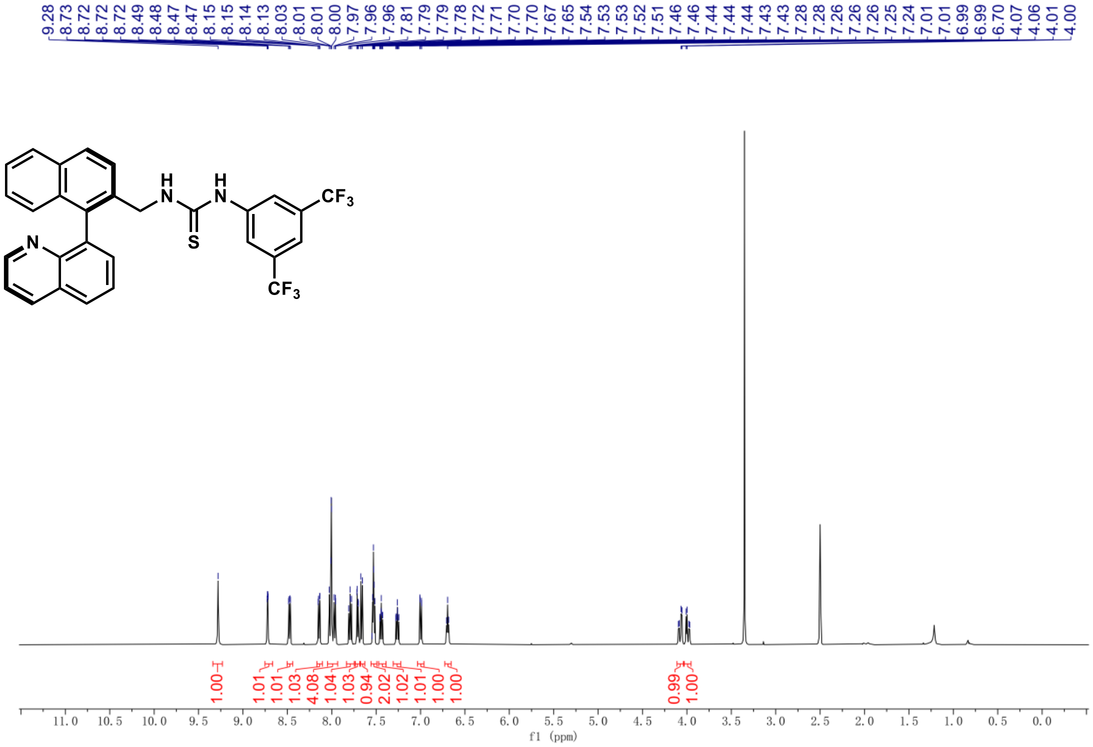

^{13}C NMR (126 MHz, DMSO-d_6) of **8d**

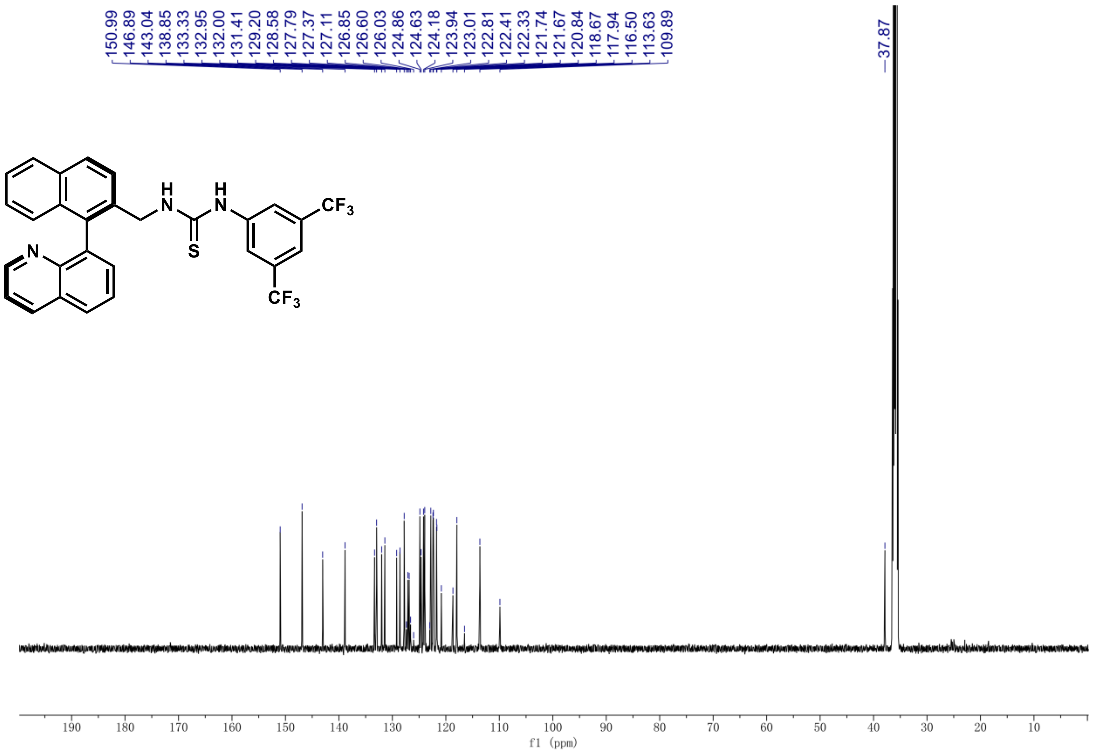

^{19}F NMR (470 MHz, DMSO-d_6) of **8d**

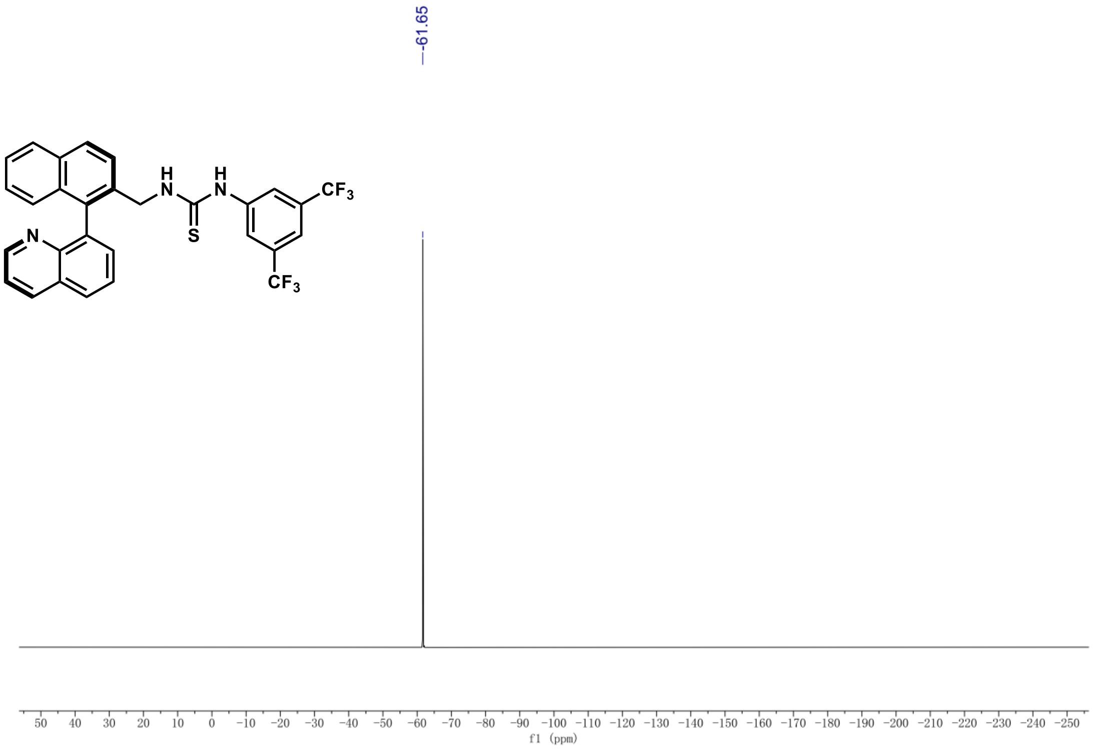

^{1}H NMR (400 MHz, $CDCl_3$) of **8e**

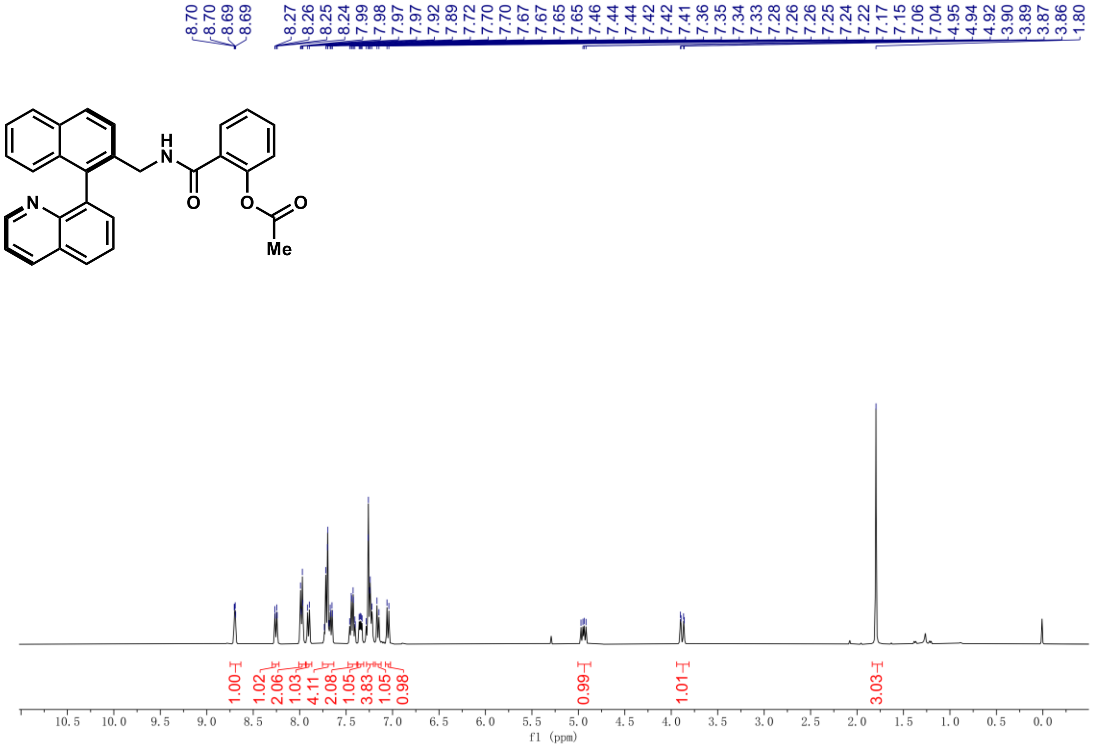

^{13}C NMR (101 MHz, $CDCl_3$) of **8e**

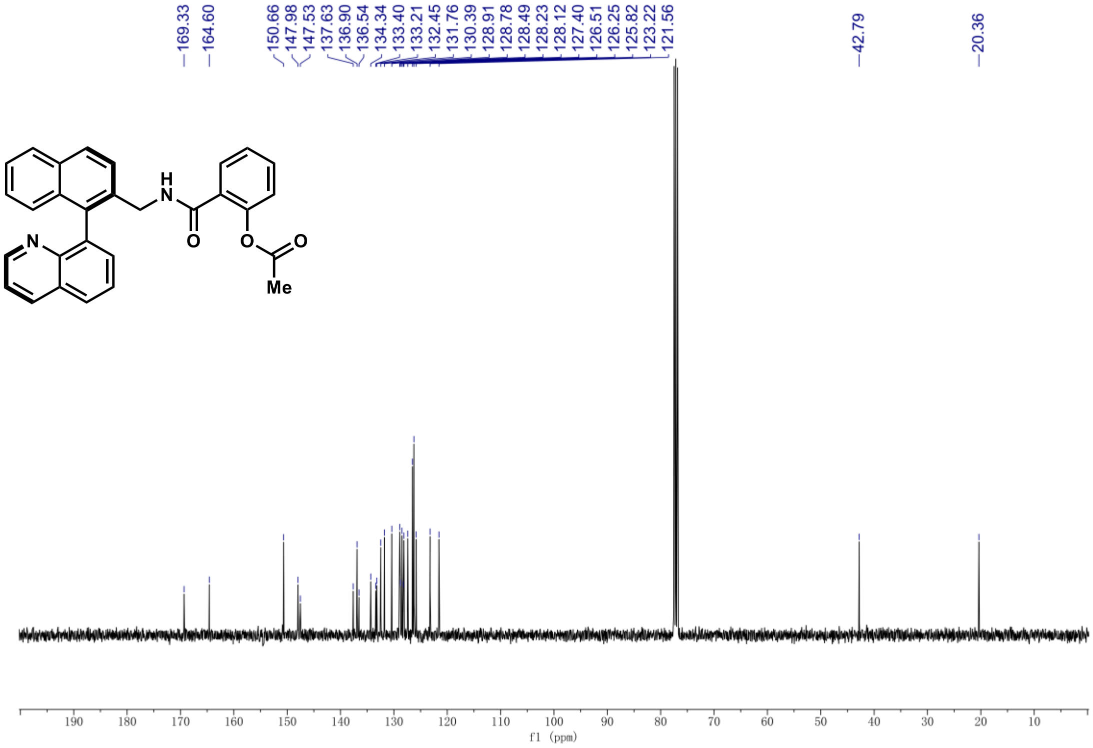

^{1}H NMR (400 MHz, $CDCl_3$) of **8f**

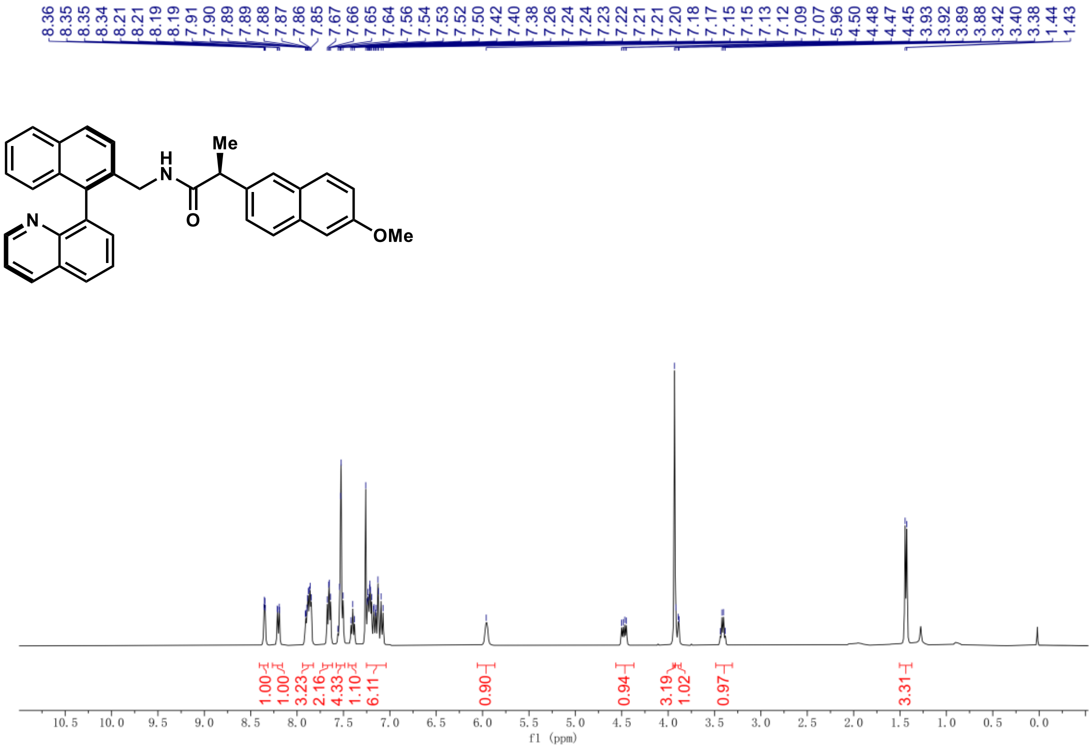

^{13}C NMR (101 MHz, $CDCl_3$) of **8f**

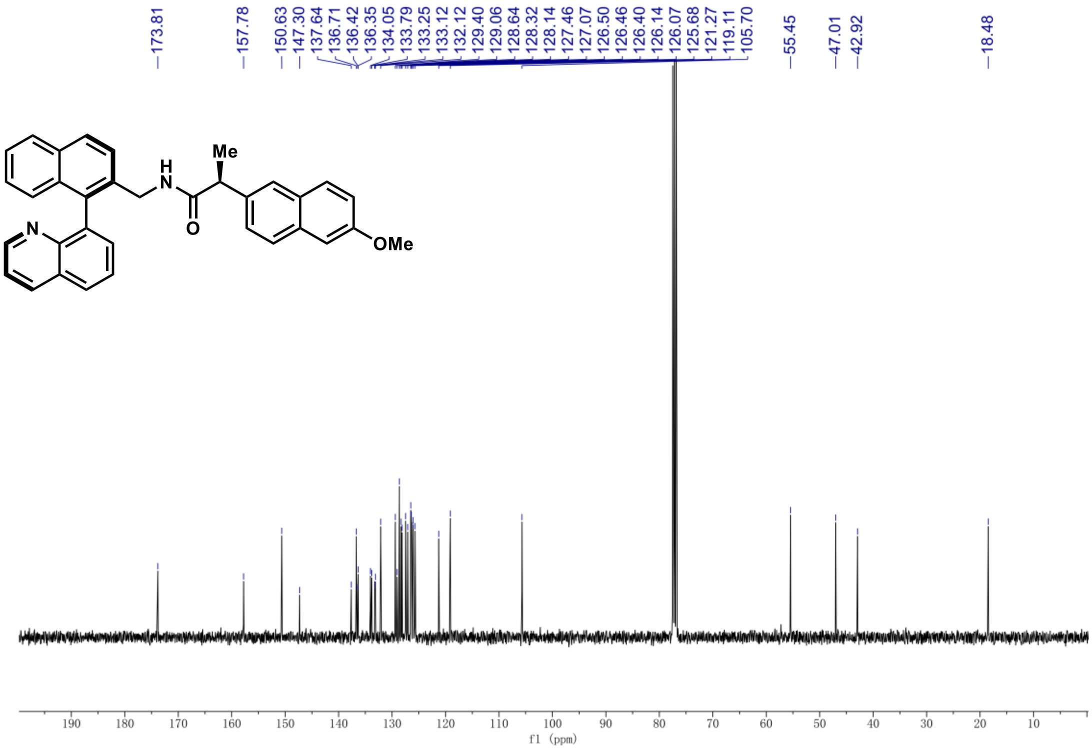

^{1}H NMR (400 MHz, $CDCl_3$) of **9**

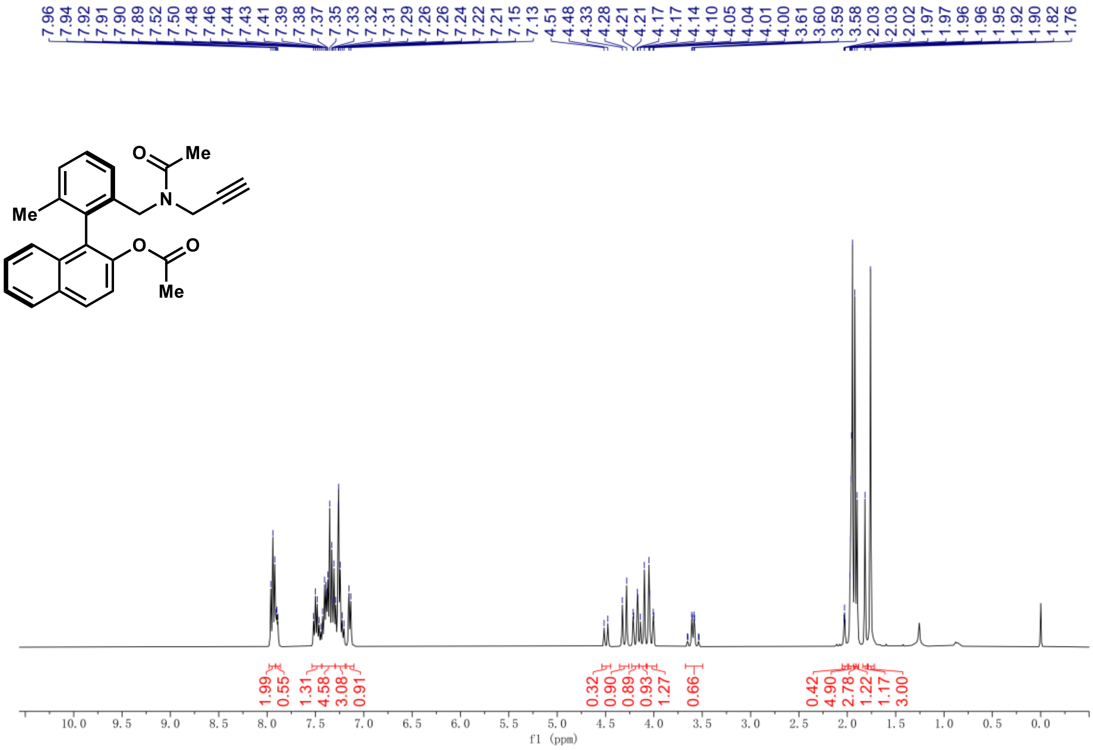

^{13}C NMR (101 MHz, $CDCl_3$) of **9**

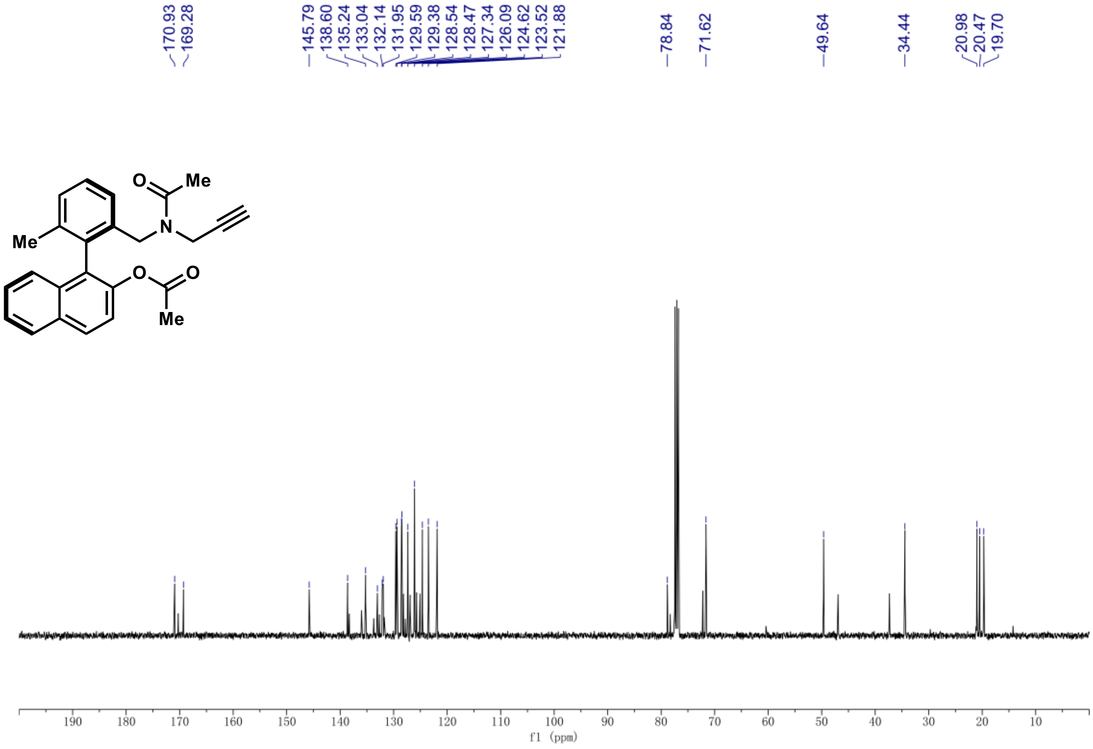

^{1}H NMR (500 MHz, $CDCl_3$) of **10**

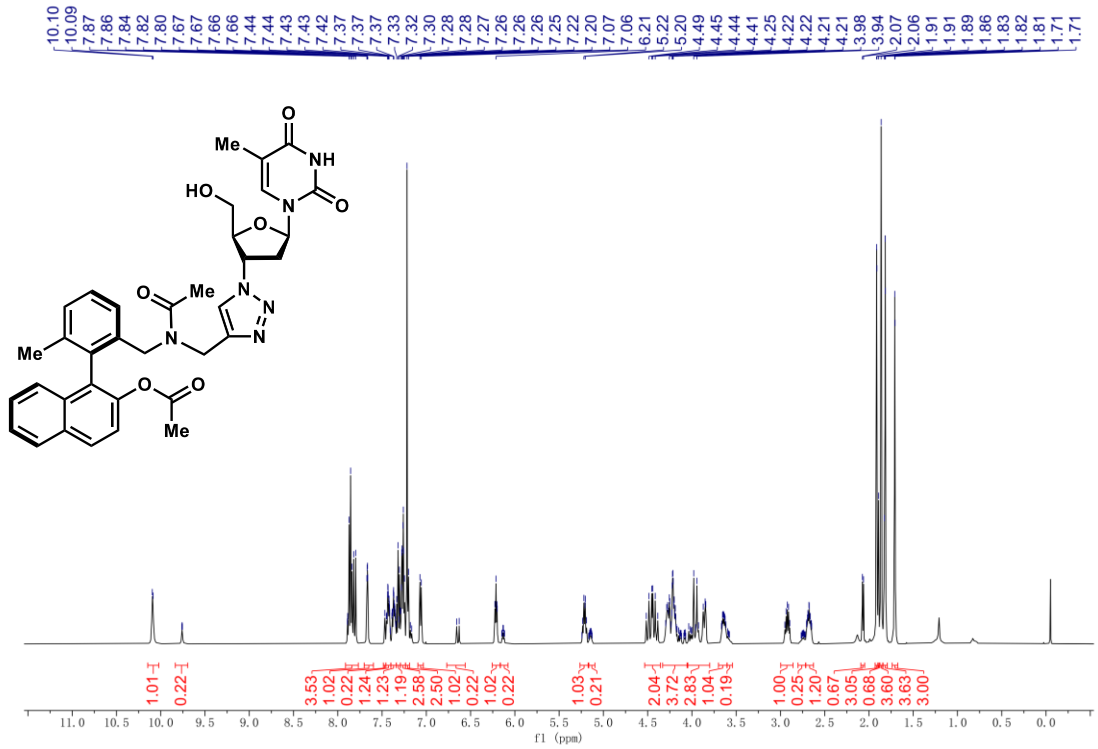

^{13}C NMR (126 MHz, $CDCl_3$) of **10**

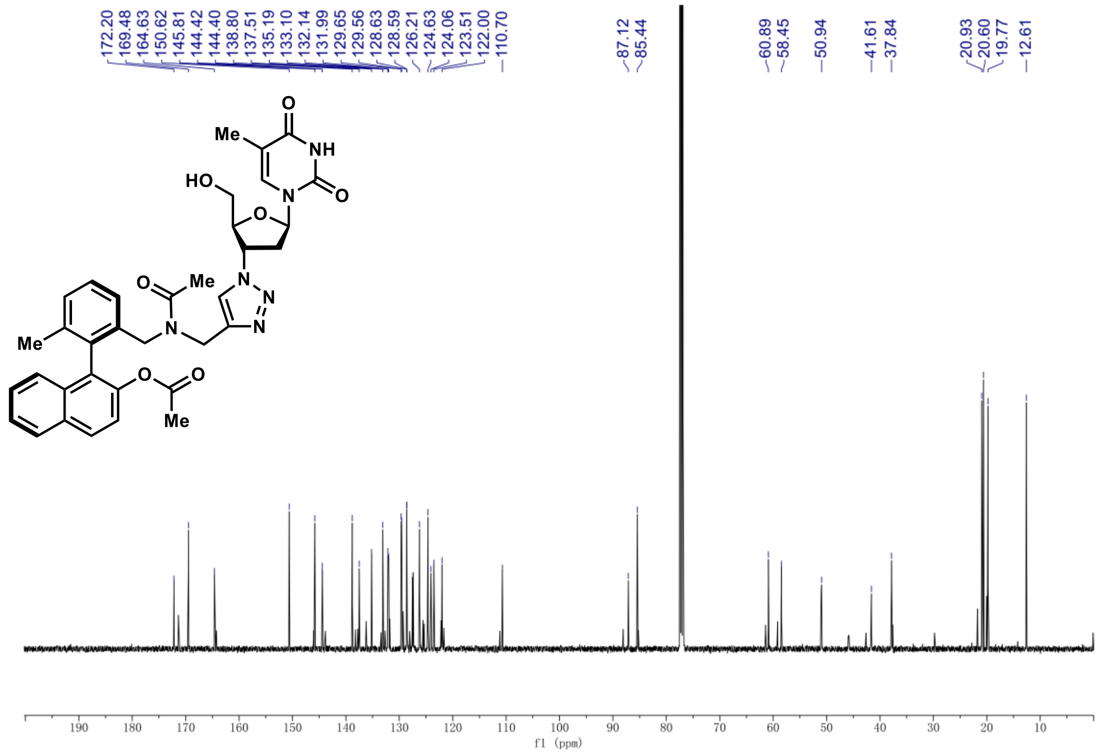

4.References

[1] G.-Q. Chen, B.-J. Lin, J.-M. Huang, L.-Y. Zhao, Q.-S. Chen, S.-P. Jia, Q. Yin, X.-M. Zhang, *J. Am. Chem. Soc.* **2018**, *140*, 8064−8068.

[2] D. Guo, J. Zhang, B. Zhang, J. Wang, *Org. Lett.* **2018**, *20*, 6284−6288.

[3] K. Mori, T. Itakura, T. Akiyama, *Angew. Chem. Int. Ed.* **2016**, *55*, 11642−11646.

[4] X. Hao, Z. Tian, Z. Yao, T. Zang, S. Song, L. Lin, T. Qiao, L. Huang, H. Fu, *Angew. Chem. Int. Ed.* **2024**, *63*, e202410112.

[5] J. A. Carmona, P. Rodríguez-Salamanca, R. Fernández, J. Lassaletta, V. Hornillos, *Angew. Chem. Int. Ed.* **2023**, *62*, e202306981.

[6] J. M. Coto-Cid, G. de Gonzalo, J. A. Carmona, J. Iglesias-Sigüenza, P. Rodríguez-Salamanca, R. Fernández, V. Hornillos, J. M. Lassaletta, *Adv. Synth. Catal.* **2024**, *366*, 909−915.

[7] J. A. Carmona, C. Rodríguez-Franco, J. López-Serrano, A. Ros, J. Iglesias-Sigüenza, R. Fernández, J. M. Lassaletta, V. Hornillos, *ACS Catal.* **2021**, *11*, 4117−4124.

[8] H. Molaei, M. M. Ghanbari, *Chin. Chem. Lett.* **2012**, *23*, 301−304.

[9] X. Hao, B. Wang, Z. Tian, Z. Yao, T. Qiao, L. Huang, H. Fu, *Org. Chem. Front.* **2025**, *12*, 2658−2669.

[10] V. R. Sirivolu, S. K. V. Vernekar, T. Ilina, N. S. Myshakina, M. A. Parniak, Z. Wang, *J. Med. Chem.* **2013**, *56*, 8765−8780.

[11] M. J. Frisch, G. W. Trucks, H. B. Schlegel, G. E. Scuseria, M. A. Robb, J. R. Cheeseman, G. Scalmani, V. Barone, G. A. Petersson, H. Nakatsuji, X. Li, M. Caricato, A. V. Marenich, J. Bloino, B. G. Janesko, R. Gomperts, B. Mennucci, H. P. Hratchian, J. V. Ortiz, A. F. Izmaylov, J. L. Sonnenberg, D. Williams-Young, F. Ding, F. Lipparini, F. Egidi, J. Goings, B. Peng, A. Petrone, T. Henderson, D. Ranasinghe, V. G. Zakrzewski, J. Gao, N. Rega, G. Zheng, W. Liang, M. Hada, M. Ehara, K. Toyota, R. Fukuda, J. Hasegawa, M. Ishida, T. Nakajima, Y. Honda, O. Kitao, H. Nakai, T. Vreven, K. Throssell, J. A. Montgomery, Jr., J. E. Peralta, F. Ogliaro, M. J. Bearpark, J. J. Heyd, E. N. Brothers, K. N. Kudin, V. N. Staroverov, T. A. Keith, R. Kobayashi, J. Normand, K. Raghavachari, A. P. Rendell, J. C. Burant, S. S. Iyengar, J. Tomasi, M. Cossi, J. M. Millam, M. Klene, C. Adamo, R. Cammi, J. W. Ochterski, R. L. Martin, K. Morokuma, O. Farkas, J. B. Foresman, D. J. Fox, Gaussian, Inc., Wallingford CT, *Gaussian 16, Revision B.01*, **2016**.

[12] A. D. Becke, *J. Chem. Phys.* **1993***, 98*, 5648−5652.

[13] C. Lee, W. Yang, R. G. Parr, *Phys. Rev. B: Condens. Matter Mater. Phys.* **1988**, *37*, 785.

[14] S. Grimme, S. Ehrlich, L. Goerigk, *J. Comput. Chem*. **2011**, *32*, 1456−1465.

[15] Y. Zhao, D. G. Truhlar, *Theor. Chem. Acc.* **2008**, *120*, 215−241.

[16] Y. Zhao, D. G. Truhlar, *Acc. Chem. Res.* **2008**, *41*, 157−167.

[17] S. Grimme, J. Antony, S. Ehrlich, H. Krieg, *J. Chem. Phys.* **2010**, *132*, 154104.

[18] F. Weigend, R. Ahlrichs, *Phys. Chem. Chem. Phys.* **2005**, *7*, 3297−3305.

[19] A. R. Casamajo, Y. Yu, C. Schnepel, C. Morrill, R. Barker, C. W. Levy, J. Finnigan, V. Spelling, K. Westerlund, M. Petchey, R. J. Sheppard, R. J. Lewis, F. Falcioni, M. A. Hayes, N. J. Turner, *J. Am. Chem. Soc.* **2023**, *145*, 22041−22046.

[20] M. H. Olsson, C. R. Sondergaard, M. Rostkowski, J. H. Jensen, *J. Chem. Theory Comput.* **2011**, *7*, 525−537.

[21] J. A. Maier, C. Martinez, K. Kasavajhala, L. Wickstrom, K. E. Hauser, C. Simmerling, *J. Chem. Theory Comput.* **2015**, *11*, 3696−3713.

[22] J. Wang, R. M. Wolf, J. W. Caldwell, P. A. Kollman, D. A. Case, *J. Comput. Chem.* **2004**, *25*, 1157−1174.
[23] W. L. Jorgensen, J. Chandrasekhar, J. D. Madura, R. W. Impey, M. L. Klein, *J. Chem. Phys.* **1983**, *79*, 926−935.
[24] D.A. Case, I.Y. Ben-Shalom, S.R. Brozell, D.S. Cerutti, T.E. Cheatham, III, V.W.D. Cruzeiro, T.A. Darden, R.E. Duke, D. Ghoreishi, G. Giambasu, T. Giese, M.K. Gilson, H. Gohlke, A.W. Goetz, D. Greene, R Harris, N. Homeyer, Y. Huang, S. Izadi, A. Kovalenko, R. Krasny, T. Kurtzman, T.S. Lee, S. LeGrand, P. Li, C. Lin, J. Liu, T. Luchko, R. Luo, V. Man, D.J. Mermelstein, K.M. Merz, Y. Miao, G. Monard, C. Nguyen, H. Nguyen, A. Onufriev, F. Pan, R. Qi, D.R. Roe, A. Roitberg, C. Sagui, S. Schott-Verdugo, J. Shen, C.L. Simmerling, J. Smith, J. Swails, R.C. Walker, J. Wang, H. Wei, L. Wilson, R.M. Wolf, X. Wu, L. Xiao, Y. Xiong, D.M. York, P.A. Kollman, *Amber 2019*, **2019**.
[25] J.-P. Ryckaert, G. Ciccotti, H. J. C. Berendsen, *J. Comput. Phys.* **1977**, *23*, 327−341.
[26] D. R. Roe, T. E. Cheatham, J. Chem. *J. Chem. Theory Comput.* **2013**, 9, 3084−3095.
[27] O. Trott, A. J. Olson, *J. Comput. Chem.* **2010**, *31*, 455−461.
